# Development of a reference and proficiency chemical list for human steatosis endpoints *in vitro*

**DOI:** 10.3389/fendo.2023.1126880

**Published:** 2023-04-24

**Authors:** Barbara Kubickova, Miriam N. Jacobs

**Affiliations:** Radiation, Chemical and Environmental Hazards (RCE), Department of Toxicology, UK Health Security Agency (UKHSA), Harwell Science and Innovation Campus, Chilton, United Kingdom

**Keywords:** HepaRG, human hazard, lipid accumulation, triglyceride, drug-induced liver injury, validation, alternative method, new approach methodology

## Abstract

The most prevalent liver disease in humans is non-alcoholic fatty liver disease, characterised by excessive hepatic fat accumulation, or steatosis. The western diet and a sedentary lifestyle are considered to be major influences, but chemical exposure may also play a role. Suspected environmental chemicals of concern include pesticides, plasticizers, metals, and perfluorinated compounds. Here we present a detailed literature analysis of chemicals that may (or may not) be implicated in lipid accumulation in the liver, to provide a basis for developing and optimizing human steatosis-relevant *in vitro* test methods. Independently collated and reviewed reference and proficiency chemicals are needed to assist in the test method development where an assay is intended to ultimately be taken forward for OECD Test Guideline development purposes. The selection criteria and considerations required for acceptance of proficiency chemical selection for OECD Test Guideline development. (i.e., structural diversity, range of activity including negatives, relevant chemical sectors, global restrictions, etc.) is described herein. Of 160 chemicals initially screened for inclusion, 36 were prioritized for detailed review. Based on the selection criteria and a weight-of-evidence basis, 18 chemicals (9 steatosis inducers, 9 negatives), including some environmental chemicals of concern, were ranked as high priority chemicals to assist *in vitro* human steatosis test method optimisation and proficiency testing, and inform potential subsequent test method (pre-)validation.

## 1 Introduction

The global increase in metabolic disorders is not only due to diet, lifestyle and genetic factors; that environmental factors also play a role is being increasingly acknowledged. Exposure to endocrine disrupting chemicals (EDCs) which disrupt metabolic functions – chemicals collectively referred to as ‘metabolic disrupting chemicals’ (MDCs) – is an environmental risk factor of concern that requires investigation to support public health protection via regulatory and policy action.

Within European chemical regulations, criteria to identify EDCs have been proposed that require information on a chemicals’ endocrine mode of action (MoA) and related adverse effects relevant for human health (1). This involves the screening and testing of EDCs according to the EU Test Methods Regulation, which mainly incorporates internationally accepted test methods developed under the Organisation for Economic Cooperation and Development (OECD). Currently, test methods to identify EDCs are based upon well-studied endocrine pathways in the oestrogen, androgen, steroidogenesis, and thyroid systems, although the need for additional endocrine modality test methods, including metabolic disruption, was recognised by OECD member countries a decade ago (2). The need for test development in the field of metabolic disorders has also been highlighted in expert surveys on identification of gaps in available test methods for EDC evaluation (3, 4) and in work on temporal aspects of EDCs (5). Currently, introduction and definition of hazard classes for the classification, labeling, and packaging of EDCs is being discussed in the EU^1^, particularly in relation to the established oestrogen, androgen, steroidogenesis and thyroid modalities, but for other endocrine modalities, such as MDCs, the potential test method tools are insufficiently characterized for regulatory purposes as yet.

MDCs can be endogenous, natural and anthropogenic chemicals that have the ability to promote metabolic changes that can ultimately result in obesity, diabetes and non-alcoholic fatty liver disease in humans (6). Whilst there are no standardised test methods adopted as regulatory chemical hazard assessment tools as yet, work is underway internationally, including as part of the EU-funded Horizon 2020 GOLIATH project (7) (https://beatinggoliath.eu/; https://cordis.europa.eu/project/id/825489). GOLIATH is developing and pre-validating *in vitro* test methods for chemical hazard testing in relation to metabolic disruption, including steatosis as a key event (KE).

**Figure 1:**
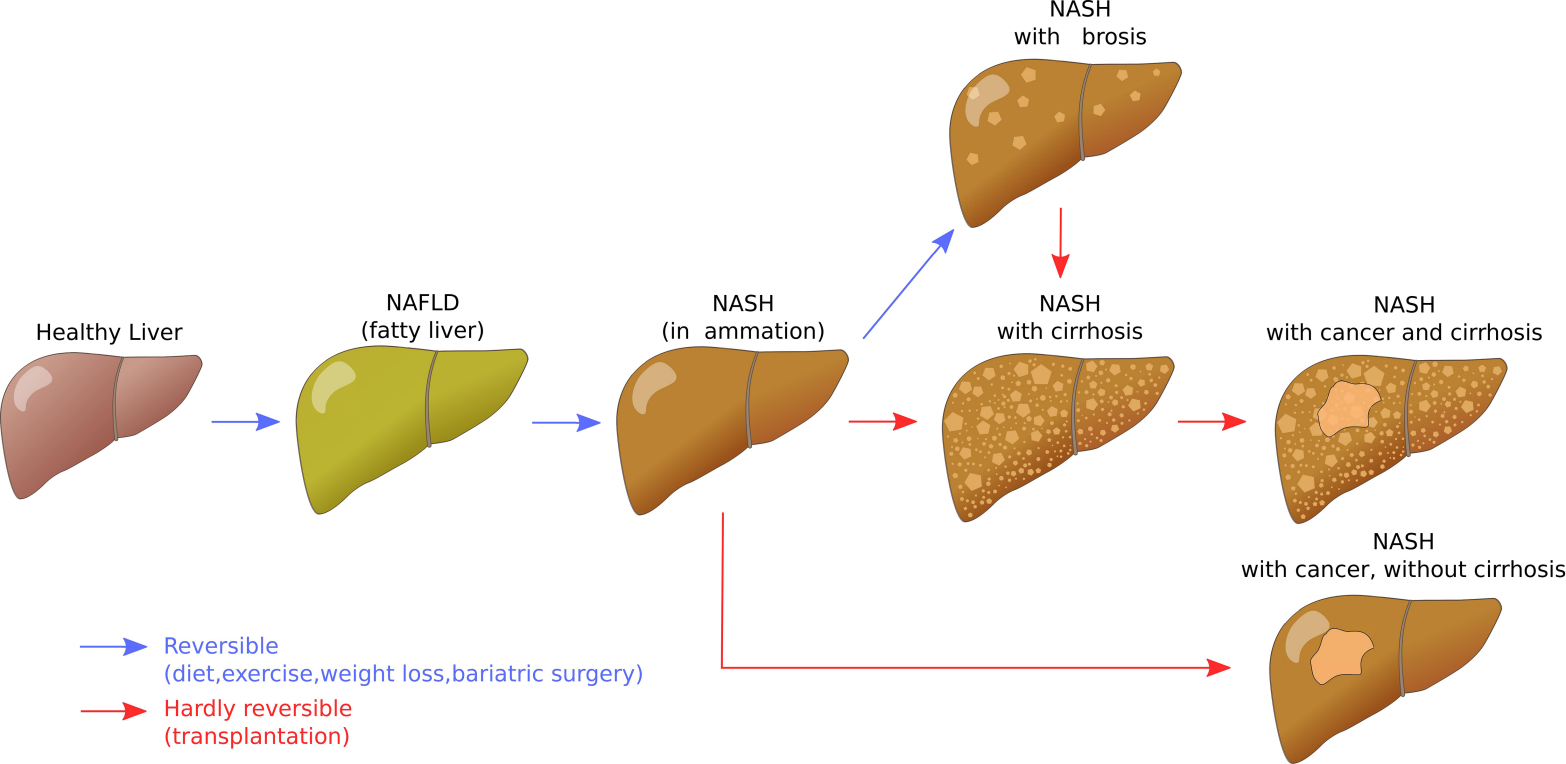
Liver disease progression for non-alcoholic fatty liver disease. Author: Signimu Figure version from 7 October 2019, 20:06, accessed on 4 January 2023. https://web.archive.org/web/20230104123719/; https://commons.wikimedia.org/wiki/File:NAFLD_liver_progression.svg (Wikimedia Commons, CC BY-SA 3.0).

Non-alcoholic fatty liver disease (NAFLD) is ‘characterised by excessive hepatic fat accumulation, defined by the presence of steatosis in >5% of hepatocytes’ (8). It is the most prevalent liver disease in humans and linked to sedentary lifestyle, western diet, but also exposure to chemicals. It can progress from (reversible) steatosis to steatohepatitis, fibrosis, or cirrhosis and cancer (**Figure 1**) (9).

Hepatic steatosis is well-known in the fields of pharmacology, medicine, and nutrition with respect to the development of NAFLD, but the significance of chemical perturbation leading to steatosis and down-stream adverse events, in response to xenobiotics is less well understood, and has been identified as a key gap in the safety assessment of chemicals at international and European levels (2, 7). The fact that it is a disease characterised by the ‘presence of steatosis in >5% of hepatocytes’, means that the occurrence and progression is measurable in cells such as hepatocytes, and *in vitro* tests can be developed on this basis. Appropriate tests need to be developed and shown to be relevant, reliable, and reproducible, before they can reasonably be expected to be utilised in Integrated Approaches to Testing and Assessment (IATA), and ultimately be suitable tool kits to be incorporated into chemical legislation.

**Figure 2:**
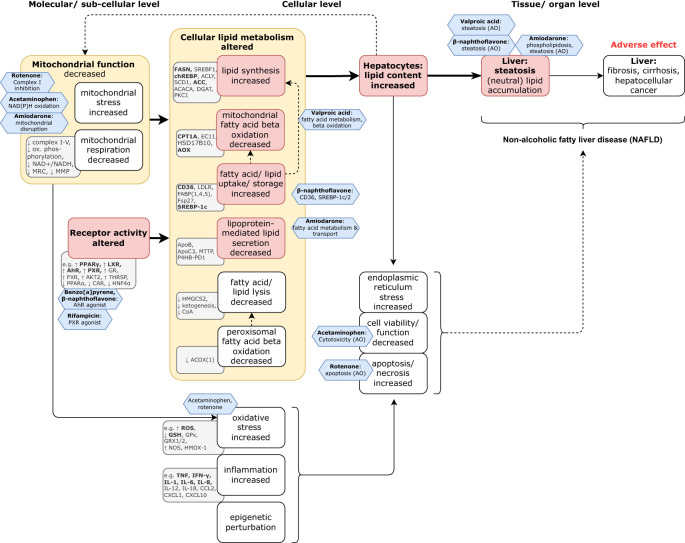
Model of the natural history of the progression towards steatosis. Steatosis is the first adaptive response to liver stress, and first indicator of NAFLD, but is (potentially) reversible. However, if left untreated, it can progress to more severe and progressively less reversible (maladaptive) disease states of fibrosis, cirrhosis, and ultimately, hepatocellular cancer. **Key**: On the basis of the mechanistic literature reviewed herein (including the **Supplementary Table 1**), the grey rectangles identify indicative biomarkers (unless otherwise indicated, the upwards or downwards arrows indicate the trend of the biomarker that corresponds to that of the associated key event); the red boxes indicate pivotal key events and the yellow boxes specify the Mode of Action. The blue hexagons capture the prototypical chemical effects/ stressors (modified from (15, 16), and with data from **Supplementary Table 2**).

Assessment of chemical hazards towards the endpoint of steatosis, and informing upon the adverse human health endpoint of NAFLD for chemical regulatory purposes, are currently based mainly upon rodent *in vivo* liver histochemistry and blood biomarkers for liver function. These parameters are reported when conducting OECD *in vivo* Test Guidelines (TGs) for acute, sub-chronic, and chronic studies, as for example under the European Union’s Registration, Evaluation, Authorisation and Restriction of Chemicals (REACH, (10)), and Plant Protection Product legislation (11). However, as steatosis is of high interest to nutrition research and the pharmacological industry, many potential candidate *in vitro* models that inform upon both molecular mechanisms and biomarkers, and also on the more apical lipid accumulation in hepatocytes relevant towards human hepatic steatosis, are being developed. They have also been mapped to a varying extent in (preliminary) Adverse Outcome Pathway models (12–14) and are also indicated in **Figure 2**.

For regulatory purposes, test methods need to demonstrate that they are able to address the intended chemical applicability domain and to classify chemicals correctly (17). To date, safety assessment of chemicals in the European Union still heavily relies on animal testing. However, substantial efforts over the last 20 years to develop, validate, and demonstrate regulatory acceptability of New Approach Methodologies (NAMs) are evident (18, 19) in an international consensus-driven approach to reduce and replace animal testing, whilst increasing human health protection relevance when assessing chemical hazard (20–23). *In vitro* test method tools can provide mechanistic understanding of apical adverse outcomes earlier, thereby contributing to avoiding redundant *in vivo* studies (19).

The objectives of the study presented here are to a) propose a list of reference and proficiency chemicals to facilitate the development, refinement, and (pre-)validation of human-health relevant steatosis *in vitro* test methods, and b) to prepare a chemical evidence basis for the integration of suitable steatosis *in vitro* test method(s), as part of a battery of suitable tests for an IATA for metabolic disruption (7, 24, 25).

Here we present a detailed analysis of chemicals, including putative negatives, as a basis for developing and optimizing human steatosis-relevant *in vitro* test methods with a measurable endpoint of hepatic lipid accumulation, for regulatory applications.

## 2 Methods

Critical considerations for the selection of reference and proficiency chemicals for test method development and validation were reported in detail earlier (17). Briefly, the chemicals selected need to be structurally diverse and representative of the chemical universe that is intended to be tested, for the respective endpoint. This includes chemicals from different industrial sectors, such as pharmaceuticals, industrial manufacturing chemicals, food additive and packaging materials, industrial by-products and contaminants, pesticides, but also (endogenous) metabolites, and naturally occurring chemicals. Chemicals subject to international agreements limiting their use and transport (e.g., the Stockholm Convention on Persistent Organic Pollutants) and undefined chemical mixtures, including isomeric mixtures, were avoided where possible, such that one can have increased confidence that the effects being reported are due to the specific chemical being investigated, and are not confounded by the interactions of other/additional chemical(s). Chemicals of limited availability or excessive cost (e.g., experimental pharmaceutical chemicals) were not prioritised in the selection, despite such chemicals often exerting specific molecular effects that are of scientific interest and can target e.g., specific nuclear receptors and signalling pathways. This is because for the intended OECD test method guideline purposes, chemicals need to be reasonably available globally in the foreseeable future, for ease of access by TG end-users. Finally, proficiency chemicals proposed for method optimisation and (pre-)validation testing should cover a range of activity (weak, moderate, strong activity), including chemicals that are negative on the respective endpoint (i.e., lipid accumulation in hepatocytes, steatosis). Ideally, the share of negative chemicals should be 25-50%, in order to reliably inform as to whether a test method can discriminate between positive and negative chemicals, and ultimately be suitable to predict the chemical activity and ideally potency in relation to the target endpoint.

### 2.1 Data sources and critical evaluation

**Figure 3:**
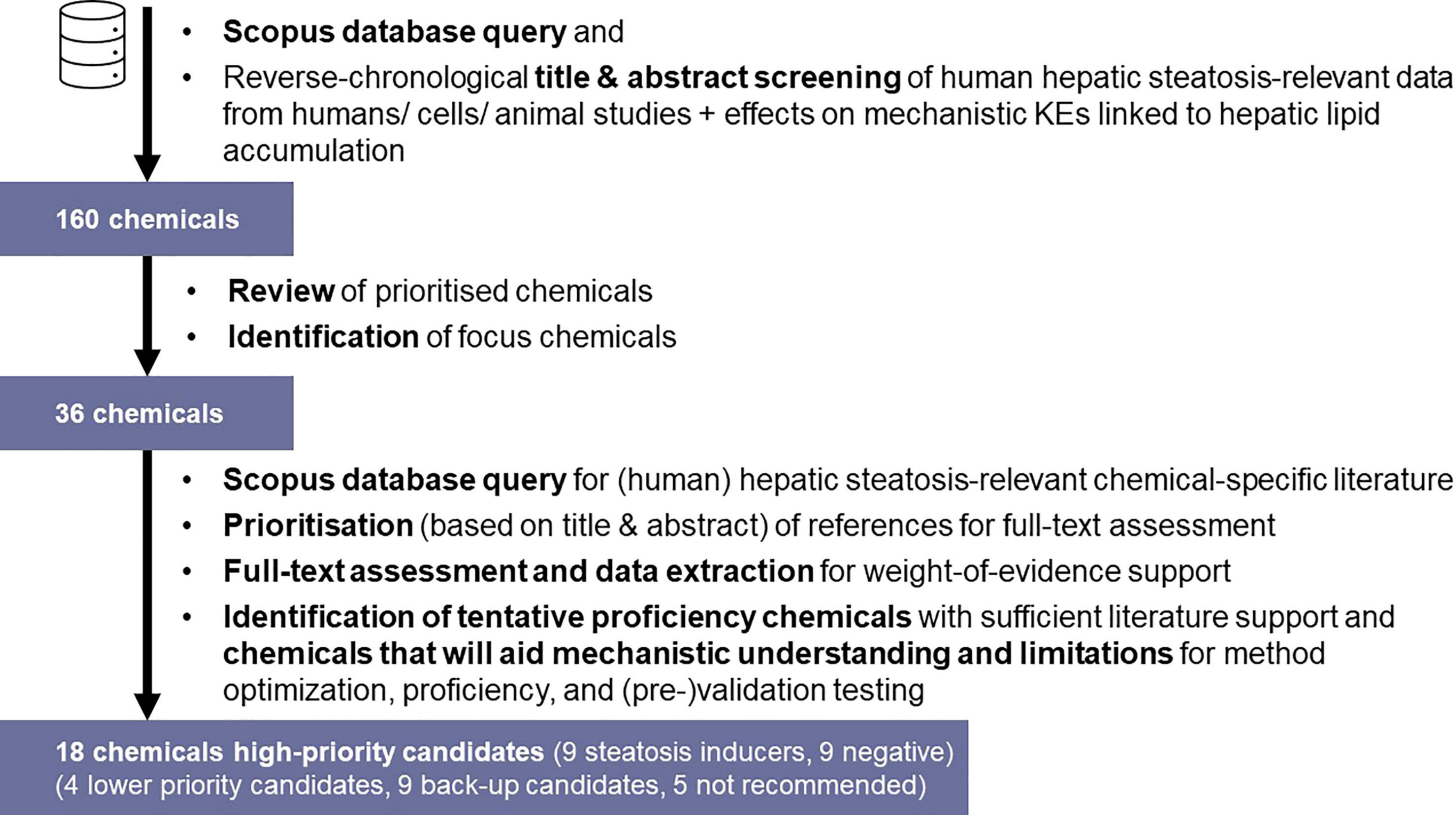
Tiered search strategy for the identification and prioritisation of preliminary proposed proficiency chemicals for *in vitro* hepatic steatosis/lipid accumulation test method optimisation, proficiency, and (pre-)validation testing.

A schematic workflow on the identification and prioritisation of chemicals for detailed steatosis-specific literature review is depicted in **Figure 3**. To retrieve relevant publications, the Scopus database (https://www.scopus.com/) was initially queried to obtain publications relevant towards (human) hepatic steatosis (see **Supplementary Material 1**, “Broad Search”). The retrieved publications were screened based on title and abstract to identify chemicals that were tested in relation to hepatic steatosis in reverse chronological order (i.e., starting from the most recent publications), with application of the above-mentioned inclusion/exclusion criteria (e.g., exclusion of studies reporting on experiments with undefined chemical mixtures/undefined extracts, co-exposure experiments, etc.). Upon identification of 160 chemicals (data not shown), this selection underwent expert review to identify focus chemicals for chemical-specific database queries in Scopus; 36 chemicals were identified for chemical-specific searches.

For chemical-specific sub-searches (see **Supplementary Material 1**, “Chemical-specific sub-searches”), the initial search strings were complemented with chemical-specific identifiers (such as chemical name, CAS number, IUPAC-compliant chemical name, name of formulation (if applicable)). If the search returned unfeasibly many hits (> 1000 per chemical) to conduct the review, the search was refined to include articles only in English, focused on original research, but including systematic reviews and Open Access articles (including hybrid/green Open Access modalities) as far as possible; this is denoted in the chemical-specific search strings provided in Annex 1, where applicable. In addition, WHO/FAO Joint Meeting on Pesticide Residues (JMPR) monographs and EFSA pesticide summaries were checked for any relevant metabolic disruption and lipid dysregulation information for the pesticides considered here, and EMA and US FDA evaluations were consulted for specified pharmaceuticals.

The chemical-specific searches were filtered for relevance towards (human) steatosis/hepatic lipid accumulation; prioritisation of references for full-text assessment was based on title and abstract screening.

Highest priority was given to human-health relevant publications (human *in vivo* epidemiological studies, clinical trials, adverse event case reports, meta-analyses, genome/ transcriptome/ metabolome-wide association studies, and *in vitro* studies with human liver-relevant models, such as primary human hepatocytes, HepaRG, or HepG2 cells). However, where only epidemiological studies with no defined exposure scenario (i.e., quantification and duration/timing of exposure) were available, the utility for conclusion on chemical hazard assessment was considered limited and utilised as supportive, rather than primary evidence. Mechanistic information from *in chemico* and *in silico* studies was considered for weight-of-evidence support, followed by rodent *in vivo* and *in vitro* studies, and studies with other well-characterised species frequently used for chemical hazard risk assessment, such as *Danio rerio* (zebrafish) or *Xenopus laevis* (African clawfrog).

The literature searches and search iterations were conducted between 6-8^th^ January 2021, and 15^th^ February 2022 (details are listed in Annex 1 for each chemical and database query).

Where available, we utilised recommendations for chemical testing related to hepatotoxicity, including steatosis, developed previously in the EU funded projects SEURAT-1 (16) and LIINTOP (15). Due to lower confidence in the predictive capacity of some high throughput data sources such as ToxCast/Tox21 projects for nuclear receptor transactivation assays and triglyceride lipid accumulation in murine pre-adipocytes (3T3-L1) (26, 27), screening of this database was not considered reliable for the chemical selection, and we did not have the resources to conduct adequate manual data cleaning, curation and repeat testing, as for example reported by (28).

Full-text assessment of prioritised references and data-extraction was conducted by two team members and tabulated during October 2021-April 2022; each reviewing the others’ results, and an additional third team member reviewed for scientific correctness and consistency (April-July 2022).

Where feasible, preliminary expected potency activity profiles for the chemicals with respect to human steatosis induction were postulated on the basis of the literature evaluation for each (prioritised) chemical. Chemicals where the evidence basis was considered supported by a reasonable number of independent literature reports were proposed as tentative proficiency chemicals.

The focus of the chemical review was to address the induction or increase of hepatic steatosis/ hepatic lipid accumulation, and not a decrease in lipid accumulation. It is noted that some chemicals, particularly pharmaceuticals and essential nutrients included in the list of proposed proficiency chemicals are reported to decrease steatosis/ hepatic lipid content *in vivo* and/or *in vitro* (**Table 1** and **Supplementary Material 2**). Reduction of lipid accumulation in the liver is an important area for therapeutic drug development (12). Human biomarkers have been identified, and there are many animal models (including genetically modified) that have been developed for studying disease mechanisms and in relation to drug discovery of hepatic lipid-lowering steatosis drugs. Additionally, there are *in vitro* steatosis models including with fatty acid pre-loading, or animal models on high fat diets (59–61). Data from these assays are included as part of the weight-of-evidence evaluation in **Supplementary Material 2**. All chemicals that do either not induce steatosis, or reduce steatosis were categorised as “negative”.

**Table 1:** Summary of the evaluation of chemicals to be proposed as preliminary reference and proficiency chemicals for (pre-)validation of *in vitro* human steatosis test method(s).

Chemical	CAS No.	Structure	Use	Triglyceride accumulation in relation to steatosis	Interaction with key pathways/ receptors	Conclusion and summary of reviewed studies
Perfluorooctanoic acid (PFOA)*	3825-26-1	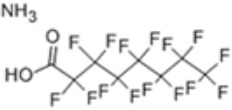	Industrial chemical, non-stick coating	Negative/weak inducer (in human) More potent in rodents	PPARα, ERα	Conflicting evidence from (human) *in vitro* and *in vivo* (human epidemiology and rodent) studies. Mechanistically, strong evidence supports PFOA acting as a (weak-moderate) PPARα agonist, therefore supporting a plausible mechanism for interfering with lipid metabolism, including in the liver. However, differences in the affinity of PFOA to murine and human PPARα have been described (weaker affinity in humans). Therefore, rodent studies might overestimate the steatosis hazard potential *via* the MIE of PPARα Data from human epidemiological studies indicate changes in blood/serum (Liver enzyme activity, cholesterol or triglyceride levels), but these are not consistent across studies. No studies reported on hepatic lipid accumulation (either not reported or not observed), but this is difficult to extrapolate from epidemiological studies/blood samples. Nevertheless, liver weight gain is identified as the occupational human health hazard with the smallest margin of exposure (29). PFOA is listed under the Stockholm Convention on Persistent Organic Pollutants, Annex A: Elimination (30). However, generation of data on well-characterised perfluoroalkyl substances, such as PFOA, will provide an evidence base for the development of suitable alternatives.
Perfluorooctanesulfonic acid (PFOS)	1763-23-1	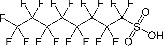	Fluorosurfactant		PPARα (agonism)	PFOS is listed under the Stockholm Convention on Persistent Organic Pollutants, Annex B: Restriction of use (30). (Due to time constraints the literature search was not pursued further, as PFOA is a priority within the GOLIATH project and restrictions to marketing and use under the Stockholm Convention apply.)
Tributyltin chloride (TBT)*	1461-22-9	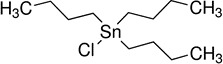	Biocide (fungicide, molluscicide)	Tentative positive Induction (potential)	RXR	Moderate-weak weight of evidence supporting TBT to induce hepatic steatosis in rodent *in vivo* and in human cell lines *in vitro*. Increased lipid accumulation is observed in human *in vitro* models at nanomolar levels, but potentially close to the cytotoxicity threshold. TBT is a model obesogenic chemical, inducing (transgenerational) increase in body weight/visceral lipid accumulation. This provides mechanistic support for a role in inducing and/or promoting hepatic lipid accumulation and steatosis.
Bisphenol A*	80-05-7	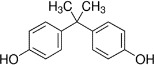	Corrosion inhibitor in fast-drying epoxy resins, thermal paper (receipts)	Tentative Positive (*in vivo*), uncertain *in vitro*		In the wider literature, epidemiological associations are reported but not causative biomarker-related evidence for BPA inducing steatosis/metabolic disruption. Mouse *in vivo* data support steatogenic effect of BPA on a weight of evidence basis. BPA (as a well-known oestrogen) is likely to also have obesity-related effect *via* ERα, as other oestrogens do. Effects on lipid accumulation reported in rodent studies and associations in human epidemiological studies might be secondary, due to dysregulated glucose homeostasis and/or insulin signalling and sensitivity in different tissues, and nuclear receptor signalling. Such secondary hepatic steatosis could be challenging to capture in single organ/tissue *in vitro* systems, such as differentiated HepaRG cells.
Triphenyl phosphate (TPP)*	115-86-6	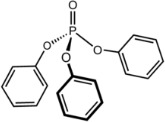	Plasticizer, organophosphate flame retardant	Positive	PXR (agonism), GR (antagonism), PPARγ (agonism), PPARα	*In vivo* data indicate a potential for changes in liver weight (increase), lipid metabolism, and altered blood lipid profile in human, rodent, and (zebra)fish, but the weight of evidence for conclusion on TPP being causative of primary hepatic steatosis is moderate. Key references to conclude on TPP inducing steatosis are human *in vivo* increase of liver weight (31), mouse *in vivo* steatosis (triglyceride accumulation in hepatocytes) (32), and zebrafish *in vivo* lipid accumulation in liver (33); details are listed below. Mechanistic support for TPP contributing to lipid accumulation can be provided by an *in vitro* study reporting increased lipid accumulation in primary human subcutaneous preadipocytes.
Triclosan	3380-34-5	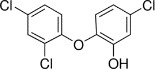	antibacterial and antifungal agent	Uncertain/ negative	Oxidative stress	Moderate weight of evidence does not indicate triclosan to induce primary hepatic steatosis. Though changes to hepatic lipid metabolism might occur due to some evidence associating triclosan with increased body weight and an obesogenic potential. Conflicting evidence is reported from an amphibian study, where early life stage exposure to triclosan induced metabolic syndrome in F0 adults (including hepatic steatosis) (34), but molecular mechanisms in *Xenopus* might be of limited relevance towards human health predictivity due to, e.g. substantial structural differences in the ligand binding pocket of relevant nuclear receptors (PPARγ). However, the reviewed literature indicates sex differences, with males being more susceptible to triclosan effects.
p,p’- Dichlorodiphenyldichloroethylene (DDE)*	72-55-9	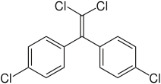	Metabolite of organochlorine insecticide	Positive		Moderate weight of evidence to support DDE causing primary hepatic steatosis. Epidemiological evidence reports an association of DDT, the parent chemical of DDE, with increased incidence of fatty liver (35), and hepatotoxicity and fatty changes in the liver observed in non-human primates exposed to DDT (36). This is supported by a systematic review and meta-analysis associating DDE exposure with increased BMI/adiposity (mechanistic support for lipid accumulation in different tissue) (37). DDT and its metabolite DDE are listed under the Stockholm Convention on Persistent Organic Pollutants, Annex B: Restriction of use (30). DDT and its metabolites are restricted for use for regulatory purposes in some OECD member countries, e.g. Japan.
Cyproconazole	94361-06-5	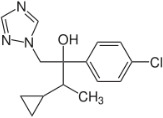	azole fungicide, wood preservative	Positive (tentative)	RARα, PXR, CYP (gene expression and protein abundance) CYP51 (target of triazole fungicides; mode of fungicidal action)	Moderate-strong mechanistic weight of evidence to support cyproconazole causing primary hepatic steatosis *in vitro*. Levels of lipid accumulation observed upon cyproconazole exposure correspond those observed with the assay positive controls (oleate, or oleate-palmitate mixture), or cyproconazole is used as a (positive) reference chemical (38). However, GLP rodent *in vivo* studies (strong weight of evidence) identify the liver as a target organ for (tri)azole toxic effects, including increased relative liver weight, but no histopathological evidence for steatosis or lipid accumulation is reported, including in 2-year chronic exposure rodent studies (39). Reasonable level of *in vivo* literature available, but high level of uncertainty to conclude on steatosis induction *in vivo*.
Tebuconazole	107534-96-3	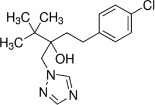	triazole fungicide (plant pathogenic fungi)	Positive (tentative)	CYP51 (target of triazole fungicides; mode of fungicidal action)	Very strong weight of evidence for tebuconazole causing (general) hepatotoxicity, but moderate weight of evidence to support tebuconazole causing primary hepatic steatosis *in vitro*. Rodent *in vivo* studies included for weight of evidence assessment followed (principally) OECD Test Guidelines and GLP standards, but offer some conflicting data: Tebuconazole is reported to induce fatty changes in the liver (steatosis not specified) *in vivo* (38), based on OECD TG 417 (toxicokinetics) studies submitted to EFSA for pesticide active substance risk assessment. Conversely, a study with Wistar rats principally following OECD TG 407 (repeated dose 28-day oral toxicity study) reported hepatocellular hypertrophy, but no steatosis/hepatic lipid accumulation (40), which is also in line with the conclusion on liver effects of the triazole fungicide class (39). The reviewed (rodent) *in vivo* data does not support a role for tebuconazole in causing primary hepatic steatosis. Tebuconazole is prioritised for inclusion in the preliminary proficiency chemical list due to the availability of human *in vivo* data (experimental exposure) for toxicokinetics and its extensive use in farming. (Tri)azole fungicides are also used as pharmaceuticals, however, due to the limited number of industrial and agrochemicals with human *in vivo* data, tebuconazole is being proposed as a chemical group representative chemical; evidence from pharmaceutical chemicals (ketoconazole, see below) was used as mechanistic supporting information. Reasonable level of *in vivo* literature available, but high level of uncertainty to conclude on steatosis induction *in vivo*.
Ketoconazole	65277-42-1	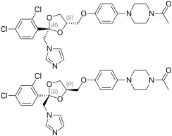	imidazole fungicide, antiandrogen and antifungal pharmaceutical	Uncertain	GR antagonist, CYP enzymes. CYP51 (target of triazole fungicides; mode of fungicidal action)	Strong weight of evidence for ketoconazole adverse effects on the liver, however the weight of evidence for causing primary hepatic steatosis/lipid accumulation is weak: adverse human health effects included hepatitis, cirrhosis, and liver failure, but not steatosis (41, 42). However, human *in vitro* mechanistic data (mitochondrial dysfunction) are supportive of a potential to induce or contribute to the development of steatosis. Stereoisomers may have different toxicological effects. (2R,4S)-(+)-ketoconazole (image top)/ (2S,4R)-(−)-ketoconazole (image bottom). Other pharmaceutical (tri-)azoles were screened (voriconazole, miconazole fluconazole), but due to time constraints, only three (tri-)azole class representative chemicals with substantial literature information available were included in the more detailed literature review.
Benzo[a]pyrene	50-32-8	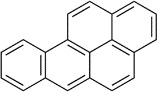	polycyclic aromatic hydrocarbon	Yes, tentative positive	CYP1A1	Moderate weight of evidence supporting induction of primary hepatic steatosis *in vitro* (including mechanistic support and transcriptomic/metabolomic data). Moderate-weak, but not contradicting weight of evidence *in vivo* (rodent and human). Strong weight of evidence supporting induction of steatosis in Amphibians *in vivo*, although human relevance and predictivity is uncertain (e.g., due to substantial species-specific structural differences in PPARγ ligand binding pocket).
Pemafibrate	848259-27-8	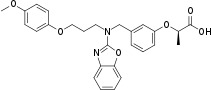	Pharmaceutical	Negative Inactive/ Decrease	PPARα (agonism)	Strong weight of evidence supporting no induction of steatosis in human *in vivo*. Mechanistic data, and pharmaceutical mode of action to lower abnormal blood lipid levels provide mechanistic support for pemafibrate not inducing primary hepatic steatosis. While pemafibrate seems to be a more potent (~100x) pharmaceutical to treat hyperlipidaemia, fenofibrate was prioritised due to longer market authorisation.
Fenofibrate	49562-28-9	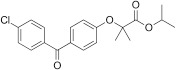	Pharmaceutical (abnormal blood lipid levels)	Negative Inactive/ Decrease	PPARα (agonism)	Moderate weight of evidence supporting no induction of steatosis in human *in vivo*. Mechanistic data, and pharmaceutical mode of action to lower abnormal blood lipid levels provide mechanistic support for fenofibrate not inducing primary hepatic steatosis. However, one study investigating liver changes by magnetic resonance indicates increased liver volume and some indication of potential increased liver lipid content (43). One human *in vitro* study reports partially conflicting data (with increased lipid accumulation at nM concentrations), but this was considered of lower relevance due to a lack of concentration-response kinetics, and lower confidence in high-throughput data, especially as human *in vivo* data are available. While pemafibrate seems to be a more potent (~100x) pharmaceutical to treat hyperlipidaemia, fenofibrate was prioritised due to longer market authorisation.
Pioglitazone	111025-46-8	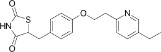	Pharmaceutical, anti-diabetic drug	Negative Decrease/ inactivity in healthy population can be inferred from decreased NASH and steatosis in diabetic patients in clinical trials		Meta-analyses of (human *in vivo*) clinical studies indicate pioglitazone improves the hepatic score in (diabetic) patients with NASH; in particular improvement was seen in steatosis and lobular inflammation. These two studies are of high confidence and were therefore utilised as a primary source of information (full-text evaluation of references was restricted where pharmaceutical mechanisms are well understood. Rosiglitazone (see below) is the thiazolidinedione with least contraindications that proceeded to market and is therefore considered a better suited candidate to be included for preliminary proficiency testing of metabolism disrupting chemicals. Therefore, the weight of evidence is indicated as moderate.
Rosiglitazone	122320-73-4	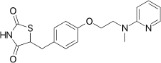	Pharmaceutical, anti-diabetic drug	Negative Decrease/ uncertain	PPAR agonist (esp. PPARγ)	Strong weight of evidence from human *in vivo* studies (clinical trials, meta-analyses) supports decreased lipid accumulation in the liver, improvement of NASH, or at least no induction/increase in lipid accumulation in the liver. The conclusion for the activity of rosiglitazone towards primary hepatic steatosis is based on these studies. Human *in vitro* studies (primarily conducted on HepaRG cells) have not necessarily looked for steatosis endpoints are conflicting, and partially contradicting the human *in vivo* observations (i.e. rosiglitazone is reported as a strong inducer of steatosis (44); this needs to be independently reproduced); the *in vitro* evidence is considered weak due to uncertainties and contradictions. However, extensive mechanistic data presented in *in vitro* studies supports an altered lipid metabolism. Moderate weight of evidence for rosiglitazone inducing steatosis is available in rodents (mice). Predisposition to steatosis was stronger in obese mice/mice on a high-fat diet, and possibly stronger in females. Furthermore, lipid accumulation was dependent on the expression level of PPARγ receptors in the liver: low expression levels were protective of rosiglitazone-induced hepatic steatosis (45). Furthermore, there are interspecies differences in activation profiles of the PPARγ receptor, which could contribute to the interspecies differences indicated here (46). It should be noted that outcomes in clinical trials focused on efficiency of rosiglitazone in (type 2) diabetic patients, as the primary pharmaceutical action is insulin sensitisation. Type 2 diabetes is already a strong indication for, and hallmark of, metabolic disruption, and therefore caution should be taken in the extrapolation of these results to the general population. Rosiglitazone is an established model PPARγ agonist and highly potent obesogen, well documented in the broader literature. Therefore, inclusion of rosiglitazone in the chemical selection is proposed, even though supporting data from human *in vivo* epidemiological and *in vitro* studies is (partially) conflicting. From the group of antidiabetic thiazolidinediones pharmaceuticals, rosiglitazone is prioritised due to having the most substantial literature body, and toxicological characterisation, and to convey mechanistic overlap between other key metabolic disruption processes/test methods under development, such as the hPPARα/γ reporter gene assays, and human *in vitro* adipogenesis assays.
Bis(2-ethylhexyl) phthalate (DEHP)	117-81-7	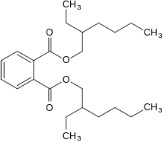	Plasticizer, metabolite	Negative Inactive/ potential increase in hepatic (neutral) lipid accumulation and steatosis; mainly mediated *via* metabolite MEHP not parent chemical		Moderate weight of evidence for no effect of DEHP on steatosis in human *in vivo*. Though several *in vivo* epidemiological studies were retrieved and analysed, steatosis/ lipid accumulation in the liver was not assessed or reported, and the primary focus was on body weight/ BMI, abdominal fat, or analytical chemistry monitoring exposure levels. For DEHP and its main metabolite MEHP studies either reported no association, or a positive association with (increased) body weight/ BMI, or abdominal fat. In addition to human *in vivo* epidemiological data, a non-human primate study (47) was retrieved and included, where 28-day oral exposure to 1 g DEHP/kg bw/d did not induce liver steatosis. Strong weight of evidence for steatosis induction is assigned to human *in vitro*, and rodent and zebrafish *in vivo* studies, also supported by mechanistic information on changes in biomarkers (e.g. PPARγ signalling pathway activation) in the liver. Interspecies differences in PPAR expression in the liver and affinity need to be considered. Steatogenic activity through PPARγ is mediated through the DEHP metabolite, MEHP. Therefore, and to include active metabolites of environmental chemicals in the chemical selection, the evidence for steatogenicity of DEHP is supportive to include its primary metabolite, MEHP in the chemical selection (see below). Additional relevant literature that was abstract-screened, but did not undergo full-text screening due to time constraints and prioritisation of the metabolite MEHP over DEHP was concluded.
Mono-ethylhexyl phthalate (MEHP)	4376-20-9	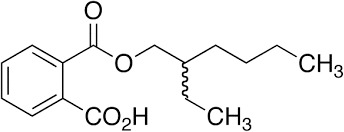	Plasticizer metabolite	Strong positive	PPAR signalling	Strong weight of evidence from human *in vivo* epidemiological studies did not find (or report) an association of MEHP exposure with hepatic steatosis. However, some studies report a positive association of MEHP exposure and body weight/BMI, or abdominal circumference, suggesting changes to the lipid homeostasis in tissues other than the liver, and thus inferring possible changes to lipid homeostasis in the liver as well. Strong weight of evidence from human *in vitro* and animal *in vivo* studies suggesting MEHP induces lipid accumulation both in hepatocytes and other cell lines. The apical endpoint of lipid accumulation is also supported by sound mechanistic data on several levels, including gene expression data and lipidomic analyses. The conclusion regarding MEHP as a steatosis-positive chemical gives higher weight to *in vitro* studies, supported by inferred disrupted lipid homeostasis in other tissues from human *in vivo* studies. MEHP is the bioactive metabolite of DEHP, and unlike DEHP it is a PPARγ agonist (in humans), providing strong mechanistic support for effects of MEHP exposure to (liver) lipid metabolism in general, and increased lipid accumulation more specifically. Therefore, we propose the inclusion of MEHP over DEHP in the provisional chemical selection list. However, if additional testing capacity is available, inclusion of both, DEHP as the parent chemical, and its metabolite MEHP might be informative to indicate and evaluate the metabolic capacity of a test system.
Amiodarone	1951-25-3	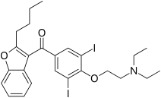	Pharmaceutical; antiarrythmic drug	Positive Strong induction of steatosis	Metabolism predominantly *via* CYP3A4 and CYP2C8 (US FDA)	Very strong weight of evidence supporting induction of hepatic steatosis in human *in vitro*, including substantial complementary supportive mechanistic information. Rodent *ex vivo* data do not conclude on steatosis induction by amiodarone, but mechanistic evidence reported is in line with molecular mechanisms observed in human cell lines *in vitro*. No human *in vivo* data were retrieved, but side effects to the liver (steatosis/lipid accumulation not specified) are included in the label of marketed amiodarone tablets (48). Amiodarone was selected as a reference chemical for hepatotoxicity as a disruptor of mitochondrial function and fatty acid metabolism, inducing steatosis in other EU funded projects: Seurat-1 and LIINTOP. Molecular-level modes of action are (mitochondrial) membrane disruption, proton uncoupling and phospholipid binding, resulting in steatosis, phospholipidosis (by accumulation of phospholipids in lysosomes), and/or cytotoxicity. Intracellular triglyceride accumulation occurs through increased *de novo* lipogenesis and decreased β-oxidation/ mitochondrial respiration.
Docosahexaenoic acid (DHA)	6217-54-5	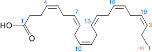	omega-3 fatty acid	Negative/ reduction	PPARα	Strong weight of evidence supports that DHA does not induce hepatic steatosis; evidence (including human *in vivo* dietary supplementation studies) suggests that DHA may contribute to decreased hepatic lipid content. A limitation in reviewing the literature was found to be that most studies (especially dietary supplementation studies) investigated effects of several poly-unsaturated fatty acids (PUFAs) and lacked a DHA-only treatment group, but composition of the PUFA dietary supplement used in clinical trials was often well-characterised, particularly for the content of DHA and eicosapentaenoic acid (EPA). Unsaturated fatty acids are well established as having nutritional modulatory function in lipid metabolism.
Resveratrol	501-36-0	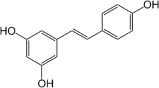	Natural phenol, stilbenoid, phytoalexin; dietary supplement	Negative/ decrease	SIRT1, LXRα	Strong weight of evidence supporting resveratrol not inducing hepatic steatosis. This is consistent *in vitro* (human and murine cell lines), and *in vivo* (human clinical trials, meta-analyses of clinical trials, and in rodents). In most experimental model studies in cells and rodents, resveratrol had no effect on hepatic lipids or decreased hepatic lipid content. However, the decrease in hepatic lipid content could not be confidently replicated in human double-blind placebo controlled *in vivo* clinical studies (in patients with NAFLD), where resveratrol had no effect on hepatic lipid levels and did not lead to a significant decrease. Mechanistically, resveratrol is a SIRT1 agonist, and its activity in lowering intracellular accumulation (especially *in vitro*) is largely attributed to this activity.
Rotenone	83-79-4	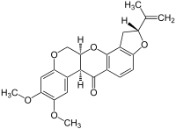	Isoflavone; insecticide, piscicide, pesticide; naturally occurring (in *Fabaceae* plants)	Negative for steatosis/lipid accumulation, but hepatotoxic	Inhibition of mitochondrial complex I, apoptosis, oxidative stress.	Strong weight of evidence supporting rotenone does not induce primary hepatic steatosis at non-cytotoxic concentrations. However, rotenone is classified as hepatotoxic; *in vitro* LOEC for cell viability is e.g., 5 µM (49). *In vivo* literature was not retrieved, probably due to the rapid onset of (cyto)toxicity: In rat *in vitro* (50), the onset of apoptosis was within 20 minutes of exposure, providing mechanistic support for the absence of steatosis. Rotenone is reported as a model chemical to impair mitochondrial respiration (by blocking mitochondrial complex I), resulting in mitochondrial membrane potential alterations, oxidative stress, and apoptosis. Rotenone was included as a reference or proficiency chemical in at least two European projects aimed to address hepatotoxicity or steatosis in *in vitro* methods: the LIINTOP project and SEURAT-1, indicating stakeholder support for inclusion of this chemical in the steatosis chemical selection.
Metformin	657-24-9	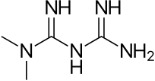	Pharmaceutical; anti-diabetic	Negative No effect/ reduction		Very strong weight of evidence supporting that metformin does not induce primary hepatic steatosis; this observation is consistent *in vitro* and *in vivo*, and metformin is being trialled for treatment of NAFLD and NASH. Data on human *in vivo* hepatic lipid lowering efficiency is inconsistent (no effect or decreased lipid accumulation/improved NASH activity; never increased lipid accumulation); it is largely attributed to the insulin sensitizing (primary therapeutic) properties of metformin, improving glucose homeostasis in the liver. Metformin reduces steatosis in experimental models. In clinical studies in patients with type 2 Diabetes Mellitus or hepatic steatosis, metformin reduced steatosis. In most studies in NASH patients, however, metformin had no effect on steatosis or other histological markers of liver function.
2-propylvaleric acid (valproic acid)	99-66-1	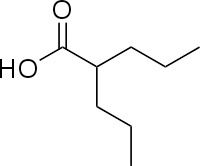	Pharmaceutical; treatment of epilepsy, seizures, bipolar disorder, migraine prevention. Branched short-chain fatty acid	Positive Induction		Strong evidence from human *in vitro* and *in vivo* studies for valproic acid (usually administered as sodium valproate) inducing hepatic steatosis. In rodent, evidence supporting induction of primary hepatic steatosis is moderate. A possible mode of action for increased hepatic lipid accumulation mediated by the valproate metabolite valproyl-CoA (valproic acid Cofactor A ester) inhibiting mitochondrial fatty acid β-oxidation; bioactivation of valproate to the CoA ester seems essential for the induction of lipid accumulation. Inhibited/decreased mitochondrial fatty acid β-oxidation was sometimes accompanied by an increase in gene or protein expression of relevant components of the mitochondrial fatty acid β-oxidation pathway, probably as a physiological reaction to counteract the decreased fatty acid oxidation. Induction of steatosis is usually observed at higher concentrations in the millimolar range, but therapeutic levels observed in human serum overlap with such high experimental concentrations. Prototypical chemical inducing steatosis, this chemical has also been previously selected as a steatosis-positive reference chemical in other EU-funded projects, namely SEURAT-1 and LIINTOP. In most experimental model studies in cells and rodents, valproic acid induced lipid accumulation. In many of the cell models, high mM concentrations (0.5-15 mM) are used to induce lipid accumulation. In some patients, valproic acid produces hepatotoxicity, including steatosis.
Caffeine	58-08-2	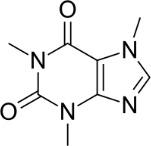	Pharmaceutical/ natural compound; stimulant	Negative/ reduction		Moderate-strong evidence, primarily from rodent and human *in vivo* that caffeine does not induce primary hepatic steatosis. Several studies report and confirm a protective effect of caffeine to prevent or ameliorate lipid accumulation in conditions leading to increased hepatic lipid accumulation (e.g., high-fat diet, or steatosis animal or *in vitro* models). Caffeine reduced lipid accumulation in experimental models. In humans, caffeine intake has been associated with a lower risk of NAFLD. However, there is insufficient clinical data available to conclusively determine the effect of caffeine on NAFLD in humans. A challenge for finding suitable caffeine references for inclusion was that many studies report effects of coffee rather than caffeine. It is likely that this influences the outcome, as multiple other constituents (e.g., other polyphenols and metabolic breakdown products) may skew the results.
Ascorbic acid (vitamin C)	50-81-7 (as salt: 134-03-2)	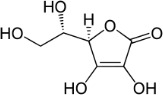	Vitamin/essential nutrient; dietary supplement	Negative/ reduction	PPARα	Strong weight of evidence supporting ascorbic acid/vitamin C not inducing primary hepatic steatosis. Most evidence evaluated reports on rodent *in vivo* studies (mouse, rat, guinea pig). Human *in vitro* and *in vivo* studies were fewer, but the results are concordant with rodent *in vivo* studies, despite ascorbic acid not being essential for rat and mice. Most studies reviewed investigated the hepatoprotective effect of ascorbic acid in models of controlled induced hepatic steatosis (chemically or dietary induced). Protection was assessed either by pre-treatment or co-administration of a defined dose of vitamin C (true protection), or reversion of previous steatosis (therapy). In both scenarios, ascorbic acid had no effect, or improved (decreased) lipid accumulation in hepatocytes, and steatosis-associated blood markers (especially serum AST and ALT). Evidence from human *in vivo* (epidemiological) data is weaker, but in line with observation from (human) *in vitro* and rodent studies. A limitation in the assessment of low ascorbic acid intake in humans is, that ascorbic acid is an essential micronutrient in humans, that needs to be supplied through the diet to prevent other deficit symptoms, also known as scurvy, and it is not ethical to control for very low/no ascorbic acid intake. One study in zebrafish identified a potentially higher baseline susceptibility of male over female fish for developing (microvesicular) hepatic steatosis in the absence of dietary ascorbic acid. However, steatosis was accompanied with general symptoms of scurvy. It must be noted, that unlike for humans, vitamin C is not an essential micronutrient for most rodents, including mice and rats (excluding guinea pigs).
Niacin (nicotinic acid; vitamin B_3_)	59-67-6	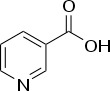	Essential human nutrient; pharmaceutical (dyslipidaemia)	Negative/ reduction		Moderate-strong evidence from *in vitro* (human and rodent), and rodent *in vivo* studies supporting no induction of primary hepatic steatosis. Few studies investigated niacin effects on lipid accumulation in “healthy lean” models, i.e., in animals not fed a high-fat diet or cell-culture models without additional supply of fatty acids, in some form. In models favoring lipid accumulation and/or obesity, niacin reduced body weight gain, improved serum lipid markers, and prevented hepatic lipid accumulation. Human *in vivo* evidence is less conclusive (moderate-weak weight of evidence). On one hand, niacin is being prescribed for the treatment of some types of hyperlipidemia, on the other hand, adverse side effects of hepatotoxicity, including elevated blood liver enzyme markers, and (usually local) fatty infiltrations of the liver are described. Adverse hepatic side effects are more likely with prolonged intake of higher doses of niacin (≥3 g/d); a higher incidence of niacin-induced hepatotoxicity was reported with the no longer authorized form of “sustained release” niacin (currently, niacin is marketed as “extended release” formulation, which is between the “immediate release”/crystalline form and the “sustained exposure” formulation). In some patients, nicotinic acid causes hepatotoxicity, including local fatty infiltration of the liver, (microvesicular) steatosis, increased serum levels of AST, ALT, and ALP, fibrosis, inflammation, and necrosis. The detailed literature review of human *in vivo* studies summarized below might give the false impression of niacin acting as an inducer of hepatic steatosis *in vivo*, but the continued marketing authorization for the treatment of dyslipidemia since the 1950s, and the rather small number of adverse effect drug reports indicates that hepatotoxicity remains a side effect, and is not a major mechanism of disease. However, the potential of niacin inducing or contributing to the development of steatosis *in vivo*, which was not observed in rodents or in *in vitro* models, makes this chemical a less suitable candidate for proficiency testing of steatosis test methods. It has also been noted that reports of induction of microvesicular steatosis in humans may be due to secondary effect and not by primary action on the liver.
Acetaminophen (Paracetamol)	103-90-2	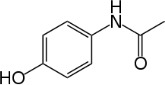	Pharmaceutical (non-steroidal anti-inflammatory drug)	Negative	CYP2E1, CYP3A4	Strong weight of evidence supporting acetaminophen not inducing primary hepatic steatosis, at non-cytotoxic levels. This is supported by data from rodent *in vivo*, rodent and human *in vitro*, as well as human *in vivo* epidemiological studies. In most studies in experimental models and humans, acetaminophen is not associated with steatosis. However, at high doses/concentrations acetaminophen does induce hepatotoxicity (drug-induced liver injury, DILI), including hepatic apoptosis, necrosis, neutrophil infiltration, and increased serum ALT and AST. In contrast to inducing steatosis, underlying steatosis/ fatty liver is a risk factor for developing DILI following acetaminophen treatment. A possible explanation for increased risk of DILI in individuals with steatosis could be a higher activity of CYP2E1, and consequently an imbalance in acetaminophen metabolism forming non-toxic metabolites more towards the reactive toxic metabolite NAPQI (via CYP2E1 and/or CYP3A4).
GW3965	405911-17-3 (hydrochloride)	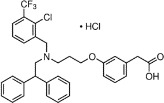	Candidate pharmaceutical	Uncertain	LXR	LXR agonist (EC50 = 190 nM hLXRα and 30 nM hLXRβ)Extensively reviewed and discussed in (16). Other LXR agonists of interest are oxysterols, which are the endogenous ligands for LXR.
Chlorpyrifos	2921-88-2	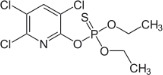	Organophosphate pesticide	Negative/ inactive (tentative)		The weight of evidence for concluding on the activity of chlorpyrifos towards primary hepatic steatosis is weak/insufficient; the retrieved literature does not allow confident conclusion on its activity. While the mechanisms observed upon exposure to chlorpyrifos are possibly facilitating/contributing to the development of steatosis, it is not likely causative of (primary) steatosis at non-toxic levels. Retrieved toxicological evaluations of chlorpyrifos by intergovernmental organizations (high confidence) do not support or indicate hepatotoxicity in general, including steatosis/lipid accumulation. Toxicological effects of concern and leading to withdrawal of marketing authorization in the EU from 2020, are indications of (non-genotoxic) carcinogenicity and neurodevelopmental effects, including in children (51). Sex-specific effects (predominantly male) observed in rodents, predisposing animals for type 2 Diabetes and atherosclerosis in adulthood by early postnatal exposure; this could also be a contributory route to metabolic syndrome/ metabolic disruption in the wider sense, but the studies did not report adverse effects in the liver. EU market approval for plant protection products containing chlorpyrifos (and chlorpyrifos-methyl) as an active ingredient was withdrawn on 6^th^ December 2019, and formally adopted by the European Commission on 10^th^ January 2020. (https://food.ec.europa.eu/plants/pesticides/approval-active-substances/renewal-approval/chlorpyrifos-chlorpyrifos-methyl_en#modal)
Thiacloprid	111988-49-9	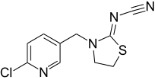	Neonicotinoid insecticide	Uncertain: Negative/ weak induction	Hepatic aromatase, thyroid hormone signalling	The weight of evidence for thiacloprid effects to the liver is moderate-weak in general, and weak/insufficient to conclude on induction of (primary) hepatic steatosis specifically. Whilst there is no strong indication for thiacloprid causing substantial lipid accumulation, some potential for lipid accumulation cannot be ruled out. In rodents, increased liver weight was reported in one study (52), but not in GLP studies reviewed for the identification of Cumulative Assessment Groups by EFSA (53). No accumulation of lipids in hepatocytes or 3T3-L1 pre-adipocytes was detected. However, in HepaRG cells *in vitro*, moderate lipid accumulation was observed at high concentrations (≥ 100 µM (38)). It is unclear if this is due to interspecies differences, or limited toxicokinetics *in vitro*. Human *in vivo* data were not retrieved. Market authorisation in the EU was withdrawn on 3 Feb 2020 (54) based on an unacceptable level of risk for honeybees (A potential structurally similar chemical to pursue could be acetamiprid, CASRN: 135410-20-7, with market approval in EU granted until 2033 (low risk for honeybees)). (55)
Acetamiprid	135410-20-7	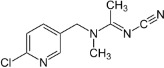	Neonicotinoid insecticide	Tentative negative		Market approval in EU granted until 2033 (low risk for honeybees) (55). From JMPR (2011): no indication for steatosis induction, including evaluation of occupational exposure and human poisoning events. Critical effects in rodents were: Decreased body weight gain and decreased food consumption; hepatocellular hypertrophy. (No new data were available for an update request to the JMPR in 2017).
Thiamethoxam	153719-23-4	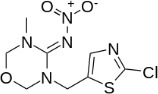	Neonicotinoid insecticide	Uncertain possibly inactive in human *in vitro* (HepaRG), but (weak) positive in rodent *in vivo*		The weight of evidence for thiamethoxam effects to the liver is moderate-weak in general, but weak/insufficient to conclude on induction of (primary) hepatic steatosis specifically. In rodents, GLP studies reviewed for the identification of Cumulative Assessment Groups by EFSA (53) indicate fatty changes in the liver, *in vivo*, however, other hepatic changes observed are more related to cytotoxicity and/or carcinogenicity than to steatosis. However, a recent study reported induction of dyslipidaemia and NAFLD, including histopathological evidence of steatosis in rat liver (56). In HepaRG cells *in vitro*, no lipid accumulation was observed up to 1 mM (38). It is unclear if this is due to interspecies differences, or limited toxicokinetics *in vitro*. Human *in vivo* data were not retrieved. Based on the larger discrepancy between (rodent) *in vivo* and (human) *in vitro* effects for thiamethoxam, inclusion of other neonicotinoid pesticides such as thiacloprid is preferred, despite the very limited amount of retrieved literature. Market approval for EU expired on 30 April 2019 based on an unacceptable level of risk for honeybees. (57)
Fructose	57-48-7	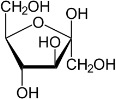	Dietary monosaccharide; ketonic simple sugar	Highly likely positive but not pursued for test method development reasons		Based on the interconnectedness of hepatic carbohydrate and lipid metabolism, responses to energy state, and with respect to the bigger picture of metabolic disruption, inclusion of a dietary sugar (glucose or fructose) was considered. However, with many human *in vitro* steatosis assays under development, glucose is already a culture medium constituent at a substantial concentration (millimolar range), and a glucose-free media formulation is not available/compatible with the assay. This would make assessment of effects of dietary sugars like glucose or fructose difficult. Furthermore, it might result in a change of cell physiology that cannot be maintained for the longer duration of the assay necessary to detect changes in lipid accumulation. A detailed literature review was not pursued further, despite indications of substantial available literature for the role of glucose homeostasis in NAFLD/metabolic disruption and syndrome.
Glucose	50-99-7	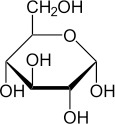	Dietary monosaccharide; aldehyde simple sugar	As above for fructose		(See comment on fructose; literature review was not pursued due to technical limitations of testing sugars in *in vitro* assays).
Rifampicin	13292-46-1	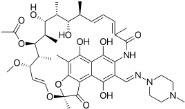	Antibiotic	Uncertain/ tentative positive	PXR	The weight of reviewed evidence is conflicting (limited full-text review conducted). Whilst a relevant study, (58) concludes that rifampicin as a model steatogenic chemical, but it was not listed or recognised as such in an earlier literature review for proposing reference chemicals for (*in vitro*) steatosis test methods (16). Rifampicin is a positive control chemical for PXR activation, especially in humans.
Tetracycline	60-54-8 (64-75-5 hydrochloride)	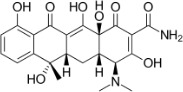	Antibiotic	Positive Induction		Strong weight of evidence supporting tetracycline inducing steatosis *in vitro* and *in vivo*; tetracycline is a model steatosis-inducing chemical. The literature summarised below is partial; due to the strong weight of evidence, including historic data more than 50 years old, not all retrieved studies were subjected to full-text scrutiny. Examples with a focus on *in vitro* studies are listed below. Tetracycline was selected as a reference chemical for hepatotoxicity as a disruptor of mitochondrial function and fatty acid metabolism, inducing steatosis in the EU funded project LIINTOP.
Oleic acid	112-80-1	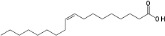	Dietary constituent; (monounsaturated omega-9) fatty acid	Positive Test method positive control		Very strong weight of evidence to support oleic acid inducing hepatic steatosis. Oleic acid is used as a positive control/reference chemical both, *in vitro* and *in vivo*. The literature reviewed is relatively substantive; oleic acid was included as a/the positive control in most reviewed *in vitro* studies (quantitatively) evaluating intracellular lipid accumulation (listed above). As a positive control chemical, it was often applied in equimolar mixture (1:1) with palmitic acid. Unsaturated fatty acids are well established as having nutritional modulatory function in lipid metabolism.
Palmitic acid	57-10-3	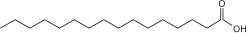	Saturated fatty acid	Positive Induction of steatosis might be subject to serum composition/ batch effects, particularly *in vitro*)		As opposed to oleic acid, palmitic acid alone does not always reliably induce steatosis, particularly under (chemically) defined culture conditions and especially with different sera/serum batches. Therefore, palmitic acid is currently not recommended as a reference or proficiency chemical for a human *in vitro* steatosis assay. The references listed below are selected examples; a complete literature review was not pursued due to time constraints and technical limitations for the use of palmitic acid in the steatosis assay. Exposure of hepatocytes to high levels of exogenous fatty acids is expected to activate PPARα and therefore modulate the baseline gene expression profile (16).

p≤0.05 as indicated in source literature, is considered statistically significant unless stated otherwise.

The development of *in vitro* models representative of a healthy lean individual (i.e., without additional fatty acid stimulation or fatty acid pre-loading) could be considered to be more technically challenging when attempting to capture therapeutic mechanisms leading to a decrease of lipid accumulation, such as very low basal lipid accumulation.

### 2.2 Chemical potency ranges

Whilst *in vitro* test methods or ‘NAMs’ were initially developed within the OECD Test Guideline Programme more for hazard classification and as prioritisation tools for subsequent *in vivo* testing, such that it was sufficient to only have negative or positive classifications, this is not the situation any longer. As we seek to be able to (gradually) replace *in vivo* testing with *in vitro* testing, the need for potency data is growing. The regulatory testing paradigm is now shifting towards IATAs and defined testing approaches, such that relevant *in vitro* test methods can ultimately be combined together in an appropriate integrated testing fashion.

Chemical potency can be used in the context of an AOP to inform as to whether an adverse effect at the molecular level leads to higher cellular and tissue effects, i.e., if it surpasses the “tipping point” leading from one key event to the next, or if physiological adaptation and compensatory mechanisms can prevent adversity at higher levels (62–66). In the context of an IATA, such potency information could be used to inform if or which higher-tier tests are needed to confidently conclude on chemical hazard characterization. For regulatory purposes, chemical potency information can be a first indicator to inform upon the derivation of a toxicological reference dose (RfD), and regulatory exposure limits such as the acceptable daily intake (ADI) (66).

While ideally chemicals of different potency (i.e., negative, weak/moderate/strong inducers) should be included in proficiency chemical lists, this was not robustly feasible for most chemicals that were evaluated. A critical limitation for such classification is the lack of a gold standard (*in vitro*) test method to conclude on such activity. For example, a chemical could be considered a weak/moderate/strong inducer both, based on the absolute magnitude of lipid accumulation achieved, in the tested concentration range, or based on the lowest observed effect concentration (LOEC). Probably, both criteria should inform upon the classification of a chemical, once a sufficiently robust test method is available. Further, the LOEC of a chemical can depend on the endpoint assessed (e.g., lipid accumulation vs. alteration of early molecular-level biomarkers), and therefore was variable across the studies included in the weight-of-evidence evaluation. Whilst not formally concluded upon in the summary tables, information on “active” concentration ranges is given in the relevant columns in **Supplementary Material 2**, where available in the respective study.

## 3 Results

A detailed table for all chemicals considered in full-text assessment, including the extracted evidence basis for the tentative assignment of steatosis activity profiles is listed in **Supplementary Material 2**, a summary of the weight-of-evidence assessment is provided in **Table 1**. **Table 2** summarises the recommendations and prioritisation for *in vitro* human steatosis test method optimisation, proficiency, and (pre-)validation testing, based on the weight-of-evidence assessment, and inclusion/ prioritisation criteria outlined in the Methods section above, in line with OECD practice and guidance for *in vitro* test method development (67).

**Table 2:** Prioritisation summary of potentially suitable proficiency chemicals for *in vitro* steatosis test method optimisation, proficiency, and (pre-)validation testing.

Chemical	Class	Activity towards steatosis/ triglyceride accumulation	Recommendation for steatosis test method proficiency testing?	Comment on suitability	Some mechanisms impacted (in relation to steatosis)
High-priority candidates					
2-propylvaleric acid (valproic acid)	Pharmaceutical; treatment of epilepsy, seizures, bipolar disorder, migraine prevention. Branched short-chain fatty acid	Induction	Yes	Model chemical for steatosis, and also reference and/or proficiency chemical for other test methods, including (neuro)developmental toxicity.	Oxidative stress, increased fatty acid β-oxidation, mitochondrial stress, phospholipidosis, PPAR, retinol metabolism
Oleic acid	Dietary constituent; (monounsaturated omega-9) fatty acid	Induction Test method positive control	Yes	Positive control and model chemical Unsaturated fatty acids are well established as having nutritional modulatory function in lipid metabolism.	
Tetracycline	Antibiotic	Induction	Yes	Model chemical	Inhibition of fatty acid catabolism, triglyceride export Oxidative stress
Triphenyl phosphate (TPP)	Plasticizer, organophosphate flame retardant	Induction	Yes		PXR (agonism), GR (antagonism), PPARγ (agonism), PPARα, Leptin resistance
Tributyltin chloride (TBT)	Metal complex, biocide (fungicide, molluscicide)	Induction (potential)	Yes		RXR, STAT5
Mono-ethylhexyl phthalate (MEHP)	Plasticizer metabolite	Induction (strong)	Yes	MEHP is the active metabolite of DEHP	PPAR
Amiodarone	Pharmaceutical; antiarrythmic drug	Induction (strong) of steatosis	Yes	Model chemical	Metabolism predominantly *via* CYP3A4 and CYP2C8 Mitochondrial function Phospholipidosis
Benzo[a]pyrene	polycyclic aromatic hydrocarbon	Induction (tentative)	Yes		AhR and CYP 1A1, 1B1, 2E1 oxidative stress
Tebuconazole	Triazole fungicide (plant pathogenic fungi)	Induction (tentative)	Yes	Class-representative chemical with human toxicokinetic data available from experimental exposure of volunteers (human *in vivo* data relevant towards hepatic steatosis were not retrieved) Reasonable level of *in vivo* literature available, but high level of uncertainty to conclude on steatosis induction *in vivo*.	CYP51 (target of triazole fungicides; mode of fungicidal action) PPARα, PXR, CAR, LXRα, AhR and related P450 enzymes
Acetaminophen (Paracetamol)	Pharmaceutical (non-steroidal anti-inflammatory drug)	Negative	Yes	Model chemical	CYP2E1, CYP3A4, mitochondrial and oxidative stress
Rotenone	Isoflavone; insecticide, piscicide, pesticide; naturally occurring (in *Fabaceae* plants)	Negative for steatosis/ lipid accumulation, but hepatotoxic	Yes		Inhibition of mitochondrial complex I, apoptosis, oxidative stress
Ascorbic acid (vitamin C)	Vitamin/essential nutrient; dietary supplement	Negative/ decrease	Yes		Antioxidant
Caffeine	Pharmaceutical/ natural compound; stimulant	Negative/ decrease	Yes		Suppression of markers for fatty acid β-oxidation
Docosahexaenoic acid (DHA)	omega-3 fatty acid	Negative/ decrease	Yes		PPARα and γ, Wnt/β-catenin signalling, COX-2
Fenofibrate	Pharmaceutical (abnormal blood lipid levels)	Negative/ decrease	Yes	While pemafibrate seems to be a more potent (~100x) pharmaceutical to treat hyperlipidaemia, fenofibrate was prioritised due to longer market authorisation.	PPARα agonism
Resveratrol	Natural phenol, stilbenoid, phytoalexin; dietary supplement	Negative/ decrease	Yes		SIRT1, LXRα
Rosiglitazone	Pharmaceutical, anti-diabetic drug	Negative/ decrease; uncertain	Yes	Despite some uncertainties, rosiglitazone is given higher priority over pioglitazone (and other thiazolidinedione considered in the early review phase). Rosiglitazone is the thiazolidinedione with least contraindications that proceeded to market and is therefore considered a better suited candidate to be included for preliminary proficiency testing of metabolism disrupting chemicals. An additional asset is the overlap with test chemicals for other metabolic disruption test methods, including rosiglitazone being a model inducer and positive control for white adipose tissue differentiation and lipid accumulation and strong PPARγ agonist.	PPARγ agonist, PXR
Metformin	Pharmaceutical; anti-diabetic	No effect/ decrease of steatosis	Yes		Decreased adiponectin
Lower-priority candidates					
p,p’- Dichlorodiphenyl-dichloroethylene (DDE)	Metabolite of organochlorine insecticide	Induction	Yes (potentially)	Use by certain OECD member states may be limited due to chemical being listed under the Stockholm Convention	Increased lipid accumulation, oxidative stress
Perfluorooctanoic acid (PFOA)	Industrial chemical, non-stick coating	Negative/weak inducer (in human) More potent in rodents	Yes (potentially)	Inclusion will provide evidence base for development of suitable alternatives Support from ongoing work at OECD and UK Environment Agency. Use by certain OECD member states may be limited due to chemical being listed under the POPs Stockholm Convention	PPARα agonist, ERα
Chlorpyrifos	Organophosphate pesticide	Negative (tentative)	Yes (potential)	Uncertain, but no indication for steatosis induction in reviews for regulatory hazard/risk assessment	CYP activity, Oxidative stress
Thiacloprid	Neonicotinoid insecticide	Uncertain: negative/weak induction	Yes (potential)	Uncertainty Inclusion of a neonicotinoid insecticide class chemical would be of interest to support the development of substitution chemicals. Evidence to conclude on activity towards steatosis is weak, but inclusion could strengthen the confidence in the classification.	Hepatic aromatase, thyroid hormone signalling, PXR, PPARγ
Back-up candidates					
Perfluorooctanesulfonic acid (PFOS)	Industrial chemical, non-stick coating, fluorosurfactant	(not concluded)	No (back-up)	(See PFOA above) Use by certain OECD member states may be limited due to chemical being listed under the Stockholm Convention Only consider inclusion if PFOA is not tested	PPARα agonism
Acetamiprid	Neonicotinoid insecticide	(not concluded)	No (back-up)		
GW3965	Candidate pharmaceutical	(not concluded)	No (back-up)	While inclusion of an LXR ligand would be mechanistically valuable, inclusion of experimental pharmaceuticals was not pursued in this study	LXR agonist
Palmitic acid	Saturated fatty acid	Induction	No (back-up)	Positive control Induction of steatosis might be subject to serum composition/batch effects, particularly *in vitro*)	Triglyceride accumulation
Cyproconazole	Azole fungicide, wood preservative	Induction (tentative)	No (back-up)	Reasonable level of *in vivo* literature available, but high level of uncertainty to conclude on steatosis induction *in vivo*.	RARα, PXR, CYP (gene expression and protein abundance) CYP51 (target of triazole fungicides; mode of fungicidal action)
Pioglitazone	Pharmaceutical, anti-diabetic drug	Negative/decrease in healthy population can be inferred from decreased NASH and steatosis in diabetic patients in clinical trials	No (back-up)	(See comment with rosiglitazone above) Rosiglitazone is the thiazolidinedione with least contraindications that proceeded to market and is therefore considered a better suited candidate than pioglitazone to be included for preliminary proficiency testing of metabolism disrupting chemicals.	PPAR agonist (esp. PPARγ)
Pemafibrate	Pharmaceutical	Negative/decrease.	No (back-up)	While pemafibrate seems to be a more potent (~100x) pharmaceutical to treat hyperlipidaemia, fenofibrate was prioritised due to longer market authorisation.	PPARα agonism
Ketoconazole	Imidazole fungicide, antiandrogen and antifungal pharmaceutical	Uncertain	No (back-up)	Provides valuable mechanistic information and class-support for the inclusion of an azole fungicide, including from clinical trials and epidemiological studies	GR antagonist, CYP enzymes. CYP51 (target of triazole fungicides; mode of fungicidal action)
Thiamethoxam	Neonicotinoid insecticide	Uncertain possibly inactive in human *in vitro* (HepaRG), but (weak) positive in rodent *in vivo*	No (back-up)		
Triclosan	antibacterial and antifungal agent	Uncertain/negative	No (back-up)	Uncertainty	Oxidative stress
Rifampicin	Antibiotic	Uncertain/tentative positive	No (back-up)	Model chemical. PXR model agonist	PXR
Currently not recommended for proficiency testing due to identified limitations					
Fructose	Dietary monosaccharide; ketonic simple sugar		No	Technical limitations may apply to the testing of sugars in *in vitro* steatosis assays. Mechanistically, dietary sugars could contribute to hepatic lipid accumulation *via* pancreatic effects (insulin/glucose homeostasis). In this case, steatosis would be a secondary effect.	
Glucose	Dietary monosaccharide; aldehyde simple sugar		No	Technical limitations may apply to the testing of sugars in *in vitro* steatosis assays. Mechanistically, dietary sugars could contribute to hepatic lipid accumulation *via* pancreatic effects (insulin/glucose homeostasis). In this case, steatosis would be a secondary effect.	
Bis(2-ethylhexyl) phthalate (DEHP)	Plasticizer, metabolite	Inactive/potential increase in hepatic (neutral) lipid accumulation and steatosis; mainly mediated *via* metabolite MEHP	No	MEHP is the active metabolite of DEHP. In studies where steatosis/lipid accumulation with DEHP was observed, this could be attributed to the MEHP metabolite.	PPARα, SREBP-1c, oxidative stress
Bisphenol A	Corrosion inhibitor in fast-drying epoxy resins, thermal paper (receipts)	Induction (*in vivo*), uncertain *in vitro*	No	Potential discrepancy in outcome *in vivo* versus *in vitro*. Indications that lipid accumulation is mediated *via* (extrahepatic) endocrine mechanisms *via* ERα.	PPARα, ERα.
Niacin (nicotinic acid; vitamin B_3_)	Essential human nutrient; pharmaceutical (dyslipidaemia)	Negative/decrease	No	In human *in vivo* studies (clinical trials and epidemiology), different adverse effects were associated with the formulation of niacin (i.e., immediate, extended, or sustained release), therefore raising some uncertainty about the predictivity *in vitro*.	Essential vitamin. Redox functions Substrate for ADP-ribose transfer reactions

Recommendations are based on the Weight of Evidence analysis (**Table 1**, **Supplementary Table S2**), and criteria for the selection of chemicals.

Overall, 18 chemicals (9 steatosis inducing chemicals: amiodarone, benzo[a]pyrene, MEHP, oleic acid, TBT chloride, tebuconazole, tetracycline, TPP, valproic acid; 9 negative chemicals: acetaminophen, ascorbic acid, caffeine, DHA, fenofibrate, metformin, resveratrol, rosiglitazone, rotenone) are identified as high-priority tentative proficiency chemicals for *in vitro* human steatosis test method optimisation, proficiency, and (pre-)validation testing. Evidence supporting these chemicals was at least “moderate”, and the reviewed literature was consistent between *in vivo* and *in vitro* findings.

Four chemicals are listed as lower priority candidate chemicals, as they are subject to international restrictions for use and/or transport (DDE, PFOA) or the weight of evidence supporting the conclusion on their activity was weaker (chlorpyrifos, thiacloprid).

While per-/polyfluoroalkyl substances (PFAS), including PFOA and PFOS, have lower priority as proficiency chemicals, the very high persistence of these chemicals in the environment will result in continued exposure over generations. Despite the international efforts to restrict manufacture and release of PFAS, e.g., through inclusion in the Stockholm Convention on Persistent Organic Pollutants (30), sadly, it has recently been demonstrated that the planetary boundary for PFAS toxicity has already been exceeded and these hazardous chemicals are detected above tolerable limits in many environmental matrices, including surface water, rainwater, and ubiquitously in soils (68).

Nine chemicals that underwent detailed chemical-specific database searches were not prioritised for proficiency testing. This includes chemical classes where another chemical with similar structure and/or activity is included in the high(er) priority chemicals (cyproconazole, ketoconazole, pemafibrate, pioglitazone, rifampicin, thiamethoxam), that are proposed as potential replacement chemicals (acetamiprid, PFOS), or that activate pathways of scientific interest to strengthen mechanistic understanding of chemically-induced steatosis, but were excluded per the search criteria (e.g., Liver X Receptor activation by GW3965, an experimental pharmaceutical chemical). It should be noted that pioglitazone had particularly strong literature support and would have been a very good high-priority candidate chemical. However, the structurally similar thiazolidinedione pharmaceutical, rosiglitazone, is the most broadly studied representative of the pharmaceutical group. It also serves as a positive control in parallel test methods addressing key processes of metabolic disruption, such as PPARγ agonism and commitment of differentiating cells to the white adipose tissue fate and lipid accumulation in (pre-)adipocytes.

On the basis of the weight-of-evidence evaluation (**Table 1** and **Supplementary Material 2**, and references therein, chemicals not currently recommended for proficiency testing (5 candidates) include dietary sugars (fructose, glucose). These are technically difficult to test during longer-term cell culture, as glucose levels are maintained in cell culture medium to mimic blood glucose levels in healthy individuals and are critical for cell health and survival. Furthermore, alteration of (dietary) sugars in the cell culture medium might be more reflective of dietary-induced, rather than chemically-induced effects. DEHP is not prioritised due to its bioactive metabolite, MEHP, being selected as a high-priority chemical. As a dietary constituent and essential vitamin niacin was reviewed as a potentially negative candidate chemical. However, despite its use for medicinal purposes and as a dietary supplement since the 1950s, adverse effects including on the liver were reported depending upon on the formulation of the vitamin (i.e., immediate release (crystalline form), sustained, or extended release) and/or idiosyncratic reactions. Also, another vitamin (ascorbic acid) is already included as a high-priority chemical. For BPA the reviewed literature suggests mechanisms leading to steatosis that might be mediated by other organs/tissues (e.g., oestrogen receptor-dependent effects on the pancreas and subsequent alterations to glucose/insulin homeostasis), and a discrepancy between activity observed *in vivo* versus *in vitro* was noted. Whilst BPA led to adverse effects to the liver, including indications of increased lipid accumulation/ steatosis, the literature data from *in vitro* studies is variable.I It is notable that the *in vitro* models are not sufficient to reliably model the interactions between the endocrine pancreas, liver, and/or other organs or tissues. This remains an important limitation to explore further, but on the basis of the reviewed literature herein, BPA is not considered to be a suitable chemical for steatosis proficiency testing, as yet.

However, testing of lower priority and back-up candidate chemicals would be of high regulatory and scientific interest, and can contribute to increasing confidence in the results generated with a test method, once it has been sufficiently demonstrated that the respective method is capable of correctly identifying and distinguishing the predicted activity for the higher priority chemicals.

## 4 Discussion

NAFLD encompasses a spectrum of progressive (reversible) disease states, from benign steatosis to irreversible pathological cirrhosis, with an estimated worldwide prevalence of approx. 25% (69). Strikingly, this was recently also confirmed in a young British cohort (4021 participants, mean age 24 years), with suspected steatosis detected in 20.7 %, and 10.0 % showing findings of severe steatosis (70). While steatosis characterised by accumulation of lipids in hepatocytes, is benign and reversible, if unrecognised and/or untreated it can progress to steatohepatitis with inflammation, fibrosis, and possibly even to irreversible stages of hepatic cirrhosis and hepatocellular carcinoma. The high prevalence of NAFLD, including in the younger population, could result in a substantial public health burden in the next decades (9). Particularly chemical/drug-induced liver disease is a subject of substantial research with regard to its implications in cardiovascular disease and metabolic disruption.

Due to the clinical importance of NAFLD, including steatosis, previous efforts to provide chemicals with high confidence and scientifical consensus on their mechanism and involvement in NAFLD are supported and expanded upon in this study (15, 16). The chemical applicability domain was expanded to account for inclusion of negative/ non-inducing chemicals and address the specific regulatory needs of the OECD Test Guidelines Programme. Further, the chemicals prioritised and tentatively proposed in this study also address mechanisms relevant to chemical-induced metabolic disruption beyond or in addition to hepatotoxicity.

We acknowledge the extensive nutrition research on fatty acid metabolism (as summarised in e.g., 71), however this is outside the scope of this chemical selection evaluation for steatosis/ hepatic lipid accumulation. Independent of the nutritional implications of diet fatty acid composition and balance, the weight of evidence for oleic acid as a positive control chemical for hepatic lipid accumulation/ liver steatosis both, *in vitro* and *in vivo* is very high (see **Supplementary Table 2**).

The work described herein provides a basis upon which suitable test methods can be developed and optimised, with the intention of ultimately adopting appropriate test methods as TGs to be used for the hazard assessment of metabolic disruption, and in this case steatosis.

Within the GOLIATH project, *in vitro* test methods to determine the potential chemical hazard of chemicals towards different key events and endpoints of metabolism disruption, including hepatic steatosis, are being developed and optimised for (pre-)validation, with the aim to inform and develop integrated testing strategies for MDCs.

As such, while steatosis is an important key event in the manifestation of metabolism disruption, it is important to integrate results from other models representative of different tissues/organs in order to achieve the most accurate representation of the (human) *in vivo* situation by *in vitro* methods. E.g., based on the retrieved and reviewed literature, BPA seems to alter hepatic metabolism, and could be inferred to contribute to hepatic steatosis (72–74). However, this was not reliably reproduced in the *in vitro* models reviewed here. A possible explanation is, that *in vivo* a parallel metabolism disrupting action of BPA is on the endocrine pancreas, altering survival of, and subsequently insulin secretion by pancreatic β cells in an oestrogen signalling-related fashion (75, 76). The resulting changes in circulating blood insulin/glucose levels can affect (hepatic) lipid metabolism and lead to lipid accumulation in hepatocytes (steatosis). Indeed, diabetes is a risk factor for the development of steatosis (77, 78). However, such indirect steatogenic effects of BPA *via* modulation of pancreatic insulin secretion might be missed in a single-tissue hepatic model. It is therefore paramount to integrate information from different relevant *in vitro* test methods, for more accurate human health hazard and risk assessment. This can be achieved, for example by delineating the use and combination of test methods in the form of an IATA.

Indeed, it has been recently demonstrated how mechanistic events leading to steatosis can be included in a read-across IATA (13, 14). Using valproic acid as a prototypical steatosis-inducing chemical, the authors comprehensively map how molecular-level events such as nuclear receptor activation can be translated across levels of biological organisation and contribute or lead to microvesicular liver steatosis (Figure 1 in 14). Based upon such mechanistic understanding of the cellular events, a tiered testing strategy for hepatic steatosis is proposed, encompassing amongst others non-target transcriptomics, targeted molecular-level screening *via* reporter gene assays (PPARγ, PXR, AhR, GR, LXR, Nrf2, ESRE, SRXN1, BIP), mitochondrial stress, cellular-level triglyceride accumulation in human hepatocyte cell lines (HepG2, HepaRG, and primary human hepatocytes), and supporting lines of evidence arising from relevant non-human models (e.g., histopathological alterations of liver in zebrafish embryos). Finally, a probabilistic approach towards weighting the evidence and uncertainties (Dempster-Shafer Theory) is successfully employed in order to inform on the hazard potential of specific branched-chain carboxylic acids (14). Despite being driven by the key event “hepatic steatosis”, this IATA case study demonstrates how read-across approaches can be supplemented with *in vitro* NAM data in order to increase confidence in *in silico* findings, and finally contribute to reducing the use of animals for research purposes, replacing animal testing with human-relevant data thus increasing human relevance. Whilst a successful demonstration of applying a read-across IATA to determining the steatosis hazard of a branched chain carboxylic acid based on NAM data, this approach was not applicable to the chemical selection reported in this study. Read-across *in silico* methods depend on a data-rich training set of structurally similar chemicals, whilst to the contrary, the proficiency chemical set for test method development needs to demonstrate a broad structural diversity to cover different chemical applicability domains (see also methods section above). However, once sufficient reliable high-quality data have been generated on the proficiency chemical set and other chemicals, e.g., with a validated test method, it would be beneficial to supplement the proficiency chemicals with *in silico* information, and potentially to employ *in silico* methods to subsequently expand and/or refine the proficiency chemical set.

The availability of an independently selected, reviewed, and expert-endorsed set of reference and proficiency chemicals is a first critical step in the development and validation of regulatory accepted test methods including NAMs. This will be utilised in the development of appropriate test systems and models.

Until relatively recently, regulatory testing relied predominantly upon rodent (*in vivo*) models, but now with the paradigm shift to *in vitro* testing, the use of human cell-based models that are more reflective of human biology are being developed and adopted as regulatory decision-making tools. With a vast diversity of human cell lines available, two models are of particular interest and relevance for modelling human hepatic processes: primary human hepatocytes (PHH) and (differentiated) human HepaRG cells. While primary human hepatocytes can reflect the interindividual diversity of a population, including sex and ethnic backgrounds, such variability reduces the reproducibility needed to ensure regulatory confidence in the results. It is therefore differentiated HepaRG cells that have been prioritised for successful validation of a human hepatic metabolism *in vitro* test method at the level of the OECD (79, 80). Another frequently used human liver model are HepG2 cells. However, HepG2 cells have been reported to be misidentified: originally thought to be a hepatocellular carcinoma cell line, it was shown to be from an hepatoblastoma, which can have implications in the interpretation of biological (and therapeutic) processes (81).

Additionally, it is being recognised that concentration-response data, that are often already recorded in non-animal alternative methods, but not yet utilised for regulatory purposes are key to leverage *in vitro* test methods beyond prioritisation and regulatory hazard identification (66). It was intended that the tentative proposed chemicals for human *in vitro* steatosis test method optimisation, proficiency, and (pre-)validation testing provided in this study cover chemicals with a range of activity (negative, low, moderate, high induction potential) towards steatosis. While good coverage and satisfactory weight-of-evidence of negative and generally positive chemicals was identified (**Table 1**), the intended potency range could not be covered in full with sufficient supporting evidence. This especially applies to chemicals that have low steatosis induction potential, or where steatosis was induced at concentrations close to (cyto-)toxicity. We recommend that initial method optimisation and proficiency testing should be based on the qualitative criterion distinguishing between steatosis (non-)inducing chemicals but testing and reporting on a range of concentrations. Once the above proposed chemicals (**Table 1**) are tested in a sufficiently developed and robust human *in vitro* steatosis test method, a (semi)quantitative secondary criterion for distinguishing between week/ moderate/ strong steatosis inducers could be added, ideally supported by indicative benchmark activity bands obtained with the respective test method.

It is acknowledged that the development of steatosis is a complex outcome, and that secondary effects e.g., due to signalling from other tissues such as the endocrine pancreas pose a challenge to *in vitro* hepatic models. This is true for most *in vitro* models, particularly those addressing more apical endpoints/ higher key events. However, the chemical selection proposed herein does not exclude other relevant endpoint-specific test methods.

In the future, developments in the field of multi-tissue *in vitro* methods, such as organ or human on a chip, may potentially help narrow the whole organism extrapolation gap between *in vitro* and *in vivo* test systems.

## 5 Conclusion

Here we have proposed a minimum set of 18 tentative preliminary proficiency chemicals for human *in vitro* steatosis test method optimisation, proficiency, and (pre-)validation testing. These chemicals have good and unequivocal support from publicly available literature which, together with a weight-of-evidence assessment with respect to their identified activity towards steatosis, showed a reasonable weight of evidence.

The provision of this set of tentative proficiency chemicals will aid the development, refinement, (pre-)validation, and acceptance of (human *in vitro*) models for steatosis, also in the context of the human health hazard and risk assessment of metabolism disrupting chemicals. Finally, this study will contribute to the forthcoming OECD detailed review paper on metabolism disrupting chemicals (OECD Test Guideline Programme workplan project 4.147).

## Author contributions

Conceptualization: MJ, BK. Methodology: BK, MJ. Investigation and review: BK, MJ. Data Curation: BK. Writing – Original Draft: BK, MJ. Writing – Review & Editing: BK, MJ. Supervision: MJ. Project Administration: MJ. Funding Acquisition: MJ. All authors contributed to the article and approved the submitted version.

## Footnotes

1 Impact Assessment available here: https://circabc.europa.eu/ui/group/a0b483a2-4c05-4058-addf-2a4de71b9a98/library/9d5b03e5-6a0b-4214-8ef4-622bd7f50ad6/details.

## References

[1] ECHA EFSA JRC Andersson N Arena M Auteri D. Guidance for the identification of endocrine disruptors in the context of regulations (EU) No 528/2012 and (EC) No 1107/2009. EFSA J (2018) 16:E05311. 32625944 10.2903/j.efsa.2018.5311 PMC7009395

[2] OECD. Detailed Review Paper on the state of science on novel in vitro and in vivo screening and testing methods and endpoints for evaluating endocrine disruptors. ENV/JM/MONO(2012)23. JT03325419, Paris: Organisation For Economic Co-Operation And Development, Environment Directorate (2012).

[3] Bopp S Nepelska M Halder M Munn S. Expert survey on identification of gaps in available test methods for evaluation of endocrine disruptors, EUR 28592 en. Luxembourg: Publications Office Of The European Union (2017).

[4] EC DG ENV. European Commission, Directorate-General for Environment, Setting priorities for further development and validation of test methods and testing approaches for evaluating endocrine disruptors: Final report Vol. 2018. Publications Office (2018). doi: 10.2779/21828

[5] EC DG ENV Joas A Bohn P Geoffroy L. Temporal aspects in the testing of chemicals for endocrine disrupting effects (In relation to human health and the environment) : Final report Vol. 2018. Publications Office (2018). doi: 10.2779/789059

[6] Heindel JJ Blumberg B Cave M Machtinger R Mantovani A Mendez MA. Metabolism disrupting chemicals and metabolic disorders. Reprod Toxicol (2017) 68:3–33. doi: 10.1016/j.reprotox.2016.10.001 PMC5365353 27760374

[7] Legler J Zalko D Jourdan F Jacobs M Fromenty B Balaguer P. The GOLIATH project: Towards an internationally harmonised approach for testing metabolism disrupting compounds. Int J Of Mol Sci (2020) 21:3480. doi: 10.3390/ijms21103480 32423144 PMC7279023

[8] EASL EASD EASO. Clinical practice guidelines for the management of non-alcoholic fatty liver disease. J Hepatol (2016) 64:1388–402. doi: 10.1016/j.jhep.2015.11.004 27062661

[9] Younossi Z Anstee QM Marietti M Hardy T Henry L Eslam M. Global burden of NAFLD and NASH: Trends, predictions, risk factors and prevention. Nat Rev Gastroenterol Hepatol (2018) 15:11–20. doi: 10.1038/nrgastro.2017.109 28930295

[10] EC. Regulation (EC) No 1907/2006 of the European parlament and of the council of 18 December 2006 concenrning the registration, evaluation, authorisation and restriction of chemicals (REACH), establishing a European chemicals agency, amending directive 1999/45/EC and repealing council regulation (EEC) No 793/93 and commission regulation (EC) No 1488/94 as well as Council Directive 76/769/EEC and Commission Directives 91/155/EEC, 93/67/EEC, 93/105/EC and 2000/21/EC. Off J Of Eur Union (2006).

[11] EC. Regulation (EC) No. 1107/2009 of the European parliament and of the council of 21 October 2009 concerning the placing of plant protection products on the market and repealing council directives 79/117/EEC and 91/414/EEC. Off J Of Eur Union (2021).

[12] Willebrords J Pereira IVA Maes M Crespo Yanguas S Colle I Van Den Bossche B. Strategies, models and biomarkers in experimental non-alcoholic fatty liver disease research. Prog In Lipid Res (2015) 59:106–25. doi: 10.1016/j.plipres.2015.05.002 PMC4596006 26073454

[13] OECD. Case study on the use of integrated approaches to testing and assessment for predictivity of a 90 day repeated dose toxicity study (OECD 408) for 2-ethylbutyric acid using a read-across approach from other branched carboxylic acids. In: OECD Series on Testing and Assessment. Paris: OECD Publishing (2018).

[14] Escher SE Aguayo-Orozco A Benfenati E Bitsch A Braunbeck T Brotzmann K. Integrate mechanistic evidence from new approach methodologies (NAMs) into a read-across assessment to characterise trends in shared mode of action. Toxicol In Vitro (2022) 79:105269. doi: 10.1016/j.tiv.2021.105269 34757180

[15] Gómez-Lechón MJ Tolosa L Castell JV Donato MT. Mechanism-based selection of compounds for the development of innovative *In vitro* approaches to hepatotoxicity studies in the LIINTOP project. Toxicol In Vitro (2010) 24:1879–89. doi: 10.1016/j.tiv.2010.07.018 20656008

[16] Jennings P Schwarz M Landesmann B Maggioni S Goumenou M Bower D. Seurat-1 liver gold reference compounds: A mechanism-based review. Arch Of Toxicol (2014) 88:2099–133. doi: 10.1007/s00204-014-1410-8 25395007

[17] Jacobs MN Kubickova B Boshoff E. Candidate proficiency test chemicals to address industrial chemical applicability domains for *In vitro* human cytochrome P450 enzyme induction. Front Toxicol (2022) 4:880818. doi: 10.3389/ftox.2022.880818 35795225 PMC9252529

[18] Knight DJ Deluyker H Chaudhry Q Vidal J-M De Boer A. A call for action on the development and implementation of new methodologies for safety assessment of chemical-based products in the EU – a short communication. Regul Toxicol And Pharmacol (2021) 119:104837. doi: 10.1016/j.yrtph.2020.104837 33249099

[19] Zuang V Dura A Ahs Lopez E Barroso J Batista Leite S Berggren E. Non-animal methods in science and regulation. Luxembourg: Publications Office Of The European Union (2022).

[20] Fentem J Malcomber I Maxwell G Westmoreland C. Upholding the EU's commitment to 'Animal testing as a last resort' under reach requires a paradigm shift in how we assess chemical safety to close the gap between regulatory testing and modern safety science. Altern Lab Anim (2021) 49:122–32. doi: 10.1177/02611929211040824 34461762

[21] Ball N Bars R Botham PA Cuciureanu A Cronin MTD Doe JE. A framework for chemical safety assessment incorporating new approach methodologies within reach. Arch Of Toxicol (2022) 96:743–66. doi: 10.1007/s00204-021-03215-9 PMC8850243 35103819

[22] Carmichael PL Baltazar MT Cable S Cochrane S Dent M Li H. Ready for regulatory use: NAMs and NGRA for chemical safety assurance. ALTEX (2022) 39:359–66. doi: 10.14573/altex.2204281 35796331

[23] Westmoreland C Bender HJ Doe JE Jacobs MN Kass GEN Madia F. Use of new approach methodologies (NAMs) in regulatory decisions for chemical safety: Report from an EPAA deep dive workshop. Regul Toxicol And Pharmacol (2022) 135:105261. doi: 10.1016/j.yrtph.2022.105261 36103951

[24] Audouze K Sarigiannis D Alonso-Magdalena P Brochot C Casas M Vrijheid M. Integrative strategy of testing systems for identification of endocrine disruptors inducing metabolic disorders-an introduction to the OBERON project. Int J Mol Sci (2020) 21(8):2988. doi: 10.3390/Ijms21082988 32340264 PMC7216143

[25] Küblbeck J Vuorio T Niskanen J Fortino V Braeuning A Abass K. The edcmet project: Metabolic effects of endocrine disruptors. Int J Mol Sci (2020) 21:3021. doi: 10.3390/Ijms21083021 32344727 PMC7215524

[26] Janesick AS Dimastrogiovanni G Vanek L Boulos C Chamorro-García R Tang W. On the utility of ToxCast™ and ToxPi as methods for identifying new obesogens. Environ Health Perspect (2016) 124:1214–26. doi: 10.1289/ehp.1510352 PMC4977052 26757984

[27] Foley B Doheny DL Black MB Pendse SN Wetmore BA Clewell RA. Editor's highlight: Screening ToxCast prioritized chemicals for PPARG function in a human adipose-derived stem cell model of adipogenesis. Toxicol Sci (2017) 155:85–100. doi: 10.1093/toxsci/kfw186 27664422 PMC5216650

[28] Filer DL Hoffman K Sargis RM Trasande L Kassotis CD. On the utility of ToxCast-based predictive models to evaluate potential metabolic disruption by environmental chemicals. Environ Health Perspect (2022) 130:57005. doi: 10.1289/EHP6779 35533074 PMC9084331

[29] Butenhoff JL Gaylor DW Moore JA Olsen GW Rodricks J Mandel JH. Characterization of risk for general population exposure to perfluorooctanoate. Regul Toxicol Pharmacol (2004) 39:363–80. doi: 10.1016/j.yrtph.2004.03.003 15135214

[30] UNEP. Stockholm Convention on persistent organic pollutants (POPs) (2019 revised version in press). In: Texts and annexes (2019). Available at: http://chm.pops.int/theconvention/overview/textoftheconvention/tabid/2232/default.aspx.

[31] ECHA ANSES. Analysis of the most appropriate risk management option (RMOA). In: Justification for the selection of a candidate CoRAP substance. ANSES On Behalf Of FR-MSCA (2019).

[32] Wang D Yan S Yan J Teng M Meng Z Li R. Effects of triphenyl phosphate exposure during fetal development on obesity and metabolic dysfunctions in adult mice: Impaired lipid metabolism and intestinal dysbiosis. Environ pollut (2019) 246:630–8. doi: 10.1016/j.envpol.2018.12.053 30605818

[33] Du Z Zhang Y Wang G Peng J Wang Z Gao S. Tphp exposure disturbs carbohydrate metabolism, lipid metabolism, and the DNA damage repair system in zebrafish liver. Sci Rep (2016) 6. doi: 10.1038/srep21827 PMC4761896 26898711

[34] Regnault C Usal M Veyrenc S Couturier K Batandier C Bulteau AL. Unexpected metabolic disorders induced by endocrine disruptors in xenopus tropicalis provide new lead for understanding amphibian decline. Proc Natl Acad Sci U.S.A. (2018) 115:E4416–25. doi: 10.1073/pnas.1721267115 PMC5948982 29686083

[35] La Merrill MA Johnson CL Smith MT Kandula NR Macherone A Pennell KD. Exposure to persistent organic pollutants (POPs) and their relationship to hepatic fat and insulin insensitivity among Asian Indian immigrants in the united states. Environ Sci And Technol (2019) 53:13906–18. doi: 10.1021/acs.est.9b03373 PMC6996970 31746186

[36] Takayama S Sieber SM Dalgard DW Thorgeirsson UP Adamson RH. Effects of long-term oral administration of DDT on nonhuman primates. J Cancer Res Clin Oncol (1999) 125:219–25. doi: 10.1007/s004320050266 PMC12199859 10235477

[37] Cano-Sancho G Salmon AG La Merrill MA. Association between exposure to p,p'-DDT and its metabolite p,p'-DDE with obesity: Integrated systematic review and meta-analysis. Environ Health Perspect (2017) 125:096002. doi: 10.1289/EHP527 28934091 PMC5915185

[38] Lichtenstein D Mentz A Schmidt FF Luckert C Buhrke T Marx-Stoelting P. Transcript and protein marker patterns for the identification of steatotic compounds in human HepaRG cells. Food And Chem Toxicol (2020) 145:111690. doi: 10.1016/j.fct.2020.111690 32810590

[39] EFSA. Scientific opinion on risk assessment for a selected group of pesticides from the triazole group to test possible methodologies to assess cumulative effects from exposure through food from these pesticides on human health. EFSA panel on plant protection products and their residues. EFSA J (2009) 7:1167.

[40] Schmidt F Marx-Stoelting P Haider W Heise T Kneuer C Ladwig M. Combination effects of azole fungicides in Male rats in a broad dose range. Toxicology (2016) 355-356:54–63. doi: 10.1016/j.tox.2016.05.018 27234313

[41] US FDA. FDA drug safety communication: FDA limits usage of nizoral (Ketoconazole) oral tablets due to potentially fatal liver injury and risk of drug interactions and adrenal gland problems. Available at: https://www.Fda.Gov/Drugs/Drug-Safety-And-Availability/Fda-Drug-Safety-Communication-Fda-Warns-Prescribing-Nizoral-Ketoconazole-Oral-Tablets-Unapproved (Accessed 05/01/2023). 24195113

[42] EMA. European Medicines Agency recommends suspension of marketing authorisations for oral ketoconazole, in: Benefit of oral ketoconazole does not outweigh risk of liver injury in fungal infections (2013). Available at: https://Www.Ema.Europa.Eu/En/Documents/Press-Release/European-Medicines-Agency-Recommends-Suspension-Marketing-Authorisations-Oral-Ketoconazole_En.Pdf (Accessed 5/01/2023).

[43] Oscarsson J Önnerhag K Risérus U Sundén M Johansson L Jansson PA. Effects of free omega-3 carboxylic acids and fenofibrate on liver fat content in patients with hypertriglyceridemia and non-alcoholic fatty liver disease: A double-blind, randomized, placebo-controlled study. J Of Clin Lipidol (2018) 12:1390–1403.E4. doi: 10.1016/j.jacl.2018.08.003 30197273

[44] Franco ME Fernandez-Luna MT Ramirez AJ Lavado R. Metabolomic-based assessment reveals dysregulation of lipid profiles in human liver cells exposed to environmental obesogens. Toxicol And Appl Pharmacol (2020) 398. doi: 10.1016/j.taap.2020.115009 32353385

[45] Gao M Ma Y Alsaggar M Liu D. Dual outcomes of rosiglitazone treatment on fatty liver. AAPS J (2016) 18:1023–31. doi: 10.1208/s12248-016-9919-9 27125895

[46] Garoche C Boulahtouf A Grimaldi M Chiavarina B Toporova L Den Broeder MJ. Interspecies differences in activation of peroxisome proliferator-activated receptor γ by pharmaceutical and environmental chemicals. Environ Sci Technol (2021) 55:16489–501. doi: 10.1021/acs.est.1c04318 34843233

[47] Satake S Nakamura C Minamide Y Kudo S Maeda H Chihaya Y. Effect of a Large dose of di (2-ethylhexyl) phthalate (DEHP) on hepatic peroxisome in cynomolgus monkeys (Macaca fascicularis). J Of Toxicol Pathol (2010) 23:75–83. doi: 10.1293/tox.23.75 22272015 PMC3234641

[48] US FDA. Cordarone (Amiodarone hcl) tablets, medicinal product print label. Available at: https://www.Accessdata.Fda.Gov/Drugsatfda_Docs/Label/2010/018972s042lbl.Pdf (Accessed 27/04 2022).

[49] Tolosa L Gómez-Lechón MJ Jiménez N Hervás D Jover R Donato MT. Advantageous use of heparg cells for the screening and mechanistic study of drug-induced steatosis. Toxicol And Appl Pharmacol (2016) 302:1–9. doi: 10.1016/j.taap.2016.04.007 27089845

[50] Isenberg JS Klaunig JE. Role of the mitochondrial membrane permeability transition (MPT) in rotenone-induced apoptosis in liver cells. Toxicol Sci (2000) 53:340–51. doi: 10.1093/toxsci/53.2.340 10696782

[51] EFSA. Statement on the available outcomes of the human health assessment in the context of the pesticides peer review of the active substance chlorpyrifos. EFSA J (2019) 17:23. 10.2903/j.efsa.2019.5809 PMC7009199 32626415

[52] Alarcan J Waizenegger J Solano MLM Lichtenstein D Luckert C Peijnenburg A. Hepatotoxicity of the pesticides imazalil, thiacloprid and clothianidin - individual and mixture effects in a 28-day study in female wistar rats. Food Chem Toxicol (2020) 140:111306. doi: 10.1016/j.fct.2020.111306 32229153

[53] Nielsen E Nørhede P Boberg J Krag Isling L Kroghsbo S Hadrup N. Identification of cumulative assessment groups of pesticides. EFSA Supporting Publications (2012) 9:269e. doi: 10.2903/sp.efsa.2012.EN-269

[54] EFSA. Peer review of the pesticide risk assessment of the active substance thiacloprid. EFSA J (2019) 17. 10.2903/j.efsa.2019.5595 PMC7009292 32626237

[55] EFSA. Peer review of the pesticide risk assessment of the active substance acetamiprid. EFSA J (2016) 14. 10.2903/j.efsa.2016.4406 PMC13093179 42016874

[56] Yang D Zhang X Yue L Hu H Wei X Guo Q. Thiamethoxam induces nonalcoholic fatty liver disease in mice via methionine metabolism disturb via nicotinamide n-methyltransferase overexpression. Chemosphere (2021) 273:129727. doi: 10.1016/j.chemosphere.2021.129727 33524747

[57] EC. Commission implementing regulation (EU) 2018/785 of 29 May 2018 amending implementing regulation (EU) No 540/2011 as regards the conditions of approval of the active substance thiamethoxam (Text with EEA relevance.). Off J Of Eur Union (2018).

[58] Allard J Bucher S Massart J Ferron P-J Le Guillou D Loyant R. Drug-induced hepatic steatosis in absence of severe mitochondrial dysfunction in heparg cells: Proof of multiple mechanism-based toxicity. Cell Biol Toxicol (2021) 37(2):151–75. doi: 10.1007/S10565-020-09537-1 PMC8012331 32535746

[59] Vinken M. Adverse outcome pathways and drug-induced liver injury testing. Chem Res In Toxicol (2015) 28:1391–7. doi: 10.1021/acs.chemrestox.5b00208 PMC4596002 26119269

[60] Abe N Kato S Tsuchida T Sugimoto K Saito R Verschuren L. Longitudinal characterization of diet-induced genetic murine models of non-alcoholic steatohepatitis with metabolic, histological, and transcriptomic hallmarks of human patients. Biol Open (2019) 8. doi: 10.1242/bio.041251 PMC6550083 31023717

[61] Fernandez-Checa JC Bagnaninchi P Ye H Sancho-Bru P Falcon-Perez JM Royo F. Advanced preclinical models for evaluation of drug-induced liver injury – consensus statement by the European drug-induced liver injury network [Pro-Euro-Dili-Net]. J Of Hepatol (2021) 75:935–59. doi: 10.1016/j.jhep.2021.06.021 34171436

[62] Shah I Setzer RW Jack J Houck KA Judson RS Knudsen TB. Using ToxCast data to reconstruct dynamic cell state trajectories and estimate toxicological points of departure. Environ Health Perspect (2016) 124:910–9. doi: 10.1289/ehp.1409029 PMC4937847 26473631

[63] Middleton A Cooper S Cull T Stark R Adeleye Y Boekelheide K. Case studies in cellular stress: Defining Adversity/Adaptation tipping points. Appl In Vitro Toxicol (2017) 3:199–210. doi: 10.1089/aivt.2017.0003

[64] Frank CL Brown JP Wallace K Wambaugh JF Shah I Shafer TJ. Defining toxicological tipping points in neuronal network development. Toxicol And Appl Pharmacol (2018) 354:81–93. doi: 10.1016/j.taap.2018.01.017 29397954

[65] Jacobs MN Colacci A Corvi R Vaccari M Aguila MC Corvaro M. Chemical carcinogen safety testing: OECD expert group international consensus on the development of an integrated approach for the testing and assessment of chemical non-genotoxic carcinogens. Arch Of Toxicol (2020) 94:2899–923. doi: 10.1007/s00204-020-02784-5 PMC7395040 32594184

[66] Jacobs MN Ezendam J Hakkert B Oelgeschlaeger M. Potential of concentration-response data to broaden regulatory application of *In vitro* test guidelines. ALTEX (2022) 39:315–21. 10.14573/altex.2107091 34882776

[67] OECD. Guidance document on good *in vitro* method practices (GIVIMP). In: OECD Series on testing and assessment. Paris: OECD Publishing (2018). doi: 10.1787/9789264304796-En

[68] Cousins IT Johansson JH Salter ME Sha B Scheringer M. Outside the safe operating space of a new planetary boundary for per- and polyfluoroalkyl substances (Pfas). Environ Sci Technol (2022) 56:11172–9. doi: 10.1021/acs.est.2c02765 PMC9387091 35916421

[69] Vernon G Baranova A Younossi ZM. Systematic review: The epidemiology and natural history of non-alcoholic fatty liver disease and non-alcoholic steatohepatitis in adults. Aliment Pharmacol Ther (2011) 34:274–85. doi: 10.1111/j.1365-2036.2011.04724.x 21623852

[70] Abeysekera KWM Fernandes GS Hammerton G Portal AJ Gordon FH Heron J. Prevalence of steatosis and fibrosis in young adults in the UK: A population-based study. Lancet Gastroenterol Hepatol (2020) 5:295–305. doi: 10.1016/S2468-1253(19)30419-4 31954687 PMC7026693

[71] Vannice G Rasmussen H. Position of the academy of nutrition and dietetics: Dietary fatty acids for healthy adults. J Of Acad Of Nutr And Dietetics (2014) 114:136–53. doi: 10.1016/j.jand.2013.11.001 24342605

[72] ANSES. Opinion of the French agency for food, environmental and occupational health & safety (Anses) in response to the consultation of the European food safety authority on its draft opinion regarding the assessment of risks to human health related to dietary exposure to Bisphenol A. (2014). Available at: https://www.anses.fr/en/system/files/SUBSTANCES2014sa0033EN.pdf.

[73] EFSA. Scientific opinion on the risks to public health related to the presence of bisphenol A (BPA) in foodstuffs. EFSA panel on food contact materials, enzymes, flavourings, processing aids and panel on dietetic products, nutrition. EFSA J (2015) 13:3978. doi: 10.2903/j.efsa.2015.3978

[74] Le Magueresse-Battistoni B Multigner L Beausoleil C Rousselle C. Effects of bisphenol a on metabolism and evidences of a mode of action mediated through endocrine disruption. Mol And Cell Endocrinol (2018) 475:74–91. doi: 10.1016/j.mce.2018.02.009 29481862

[75] Babiloni-Chust I Dos Santos RS Medina-Gali RM Perez-Serna AA Encinar J-A Martinez-Pinna J. G Protein-coupled estrogen receptor activation by bisphenol-a disrupts the protection from apoptosis conferred by the estrogen receptors ERα and ERβ in pancreatic beta cells. Environ Int (2022) 164:107250. doi: 10.1016/j.envint.2022.107250 35461094

[76] Dos Santos RS Medina-Gali RM Babiloni-Chust I Marroqui L Nadal A. *In vitro* assays to identify metabolism-disrupting chemicals with diabetogenic activity in a human pancreatic beta-cell model. Int J Of Mol Sci (2022) 23:5040. doi: 10.3390/ijms23095040 35563431 PMC9102687

[77] El-Serag HB Tran T Everhart JE. Diabetes increases the risk of chronic liver disease and hepatocellular carcinoma. Gastroenterology (2004) 126:460–8. doi: 10.1053/j.gastro.2003.10.065 14762783

[78] Williamson RM Price JF Glancy S Perry E Nee LD Hayes PC. Prevalence of and risk factors for hepatic steatosis and nonalcoholic fatty liver disease in people with type 2 diabetes: The Edinburgh type 2 diabetes study. Diabetes Care (2011) 34:1139–44. doi: 10.2337/dc10-2229 PMC3114489 21478462

[79] JRC. Multi-study validation trial for cytochrome P450 induction providing a reliable human metabolically competent standard model or method using the human cryopreserved primary hepatocytes and the human cryopreserved heparg® cell line. In: Validation project report. European Comission Joint Research Centre, Institute For Health And Consumer Protection, European Union Reference Laboratory For Alternatives To Animal Testing (EURL ECVAM) (2014). Available at: https://Tsar.Jrc.Ec.Europa.Eu/System/Files/Published/Cyp_Validation%20project%20report_Final%2020140314_0.Pdf.

[80] Bernasconi C Pelkonen O Andersson TB Strickland J Wilk-Zasadna I Asturiol D. Validation of *In vitro* methods for human cytochrome P450 enzyme induction: Outcome of a multi-laboratory study. Toxicol In Vitro (2019) 60:212–28. doi: 10.1016/j.tiv.2019.05.019 PMC6718736 31158489

[81] López-Terrada D Cheung SW Finegold MJ Knowles BB. Hep G2 is a hepatoblastoma-derived cell line. Hum Pathol (2009) 40:1512–5. doi: 10.1016/j.humpath.2009.07.003 19751877

