# Supplementary material for: Development of a reference and proficiency chemical list for human steatosis endpoints in vitro
Source: Front Endocrinol (Lausanne). 2023 Apr 24;14:1126880. doi: 10.3389/fendo.2023.1126880 (PMC10166001; doi:10.3389/fendo.2023.1126880)
Supplement: Supplementary file 1 [file DataSheet_1.docx]

Supplementary Material 1

# Search terms for Scopus database queries

Search term strings that were used to query the Scopus database to retrieve publications for review for the steatosis chemical selection (dates are denoted in the format YYYMMDD, time indications refer to GMT).

“Broad Search”, Searched 2020-12-09, 09:26 GMT: (6436 entries):

( TITLE-ABS-KEY ( ( liver OR hepat* ) W/3 fat* ) OR TITLE-ABS-KEY ( ( liver OR hepat* ) PRE/3 fat* ) OR TITLE-ABS-KEY ( steato* ) AND TITLE-ABS-KEY ( ( ( lipid OR triglyceride OR fat OR cholesterol ) W/3 ( accumulat* OR homeosta* OR metaboli* ) ) OR ( ( lipid OR triglyceride OR fat OR cholesterol ) PRE/3 ( accumulat* OR homeosta* OR metaboli* ) ) ) AND TITLE-ABS-KEY ( human ) AND NOT TITLE-ABS-KEY ( nanopart* OR infect* OR mixture OR covid ) ) AND ( LIMIT-TO ( DOCTYPE , "ar" ) OR LIMIT-TO ( DOCTYPE , "ed" ) OR LIMIT-TO ( DOCTYPE , "cp" ) OR LIMIT-TO ( DOCTYPE , "le" ) OR LIMIT-TO ( DOCTYPE , "no" ) OR LIMIT-TO ( DOCTYPE , "sh" ) OR LIMIT-TO ( DOCTYPE , "ch" ) OR LIMIT-TO ( DOCTYPE , "bk" ) OR LIMIT-TO ( DOCTYPE , "er" ) ) AND ( LIMIT-TO ( LANGUAGE , "English" ) )

Chemical-specific sub-searches in the Scopus database:

**TBT (20210106 17:06)**

( TITLE-ABS-KEY ( ( ( liver OR hepat* ) W/3 fat* ) ) OR TITLE-ABS-KEY ( ( ( liver OR hepat* ) PRE/3 fat* ) ) OR TITLE-ABS-KEY ( ( ( ( lipid OR triglyceride OR fat OR cholesterol ) W/3 ( accumulat* OR homeosta* OR metaboli* ) ) OR ( ( lipid OR triglyceride OR fat OR cholesterol ) PRE/3 ( accumulat* OR homeosta* OR metaboli* ) ) ) ) AND TITLE-ABS-KEY ( human ) AND TITLE-ABS-KEY ( ( tributyltin OR organotin OR tbt OR 56-35-9 ) ) AND NOT TITLE-ABS-KEY ( ( nanopart* OR infect* OR mixture OR covid ) ) )

**PFOA (20210106 17:08)**

( TITLE-ABS-KEY ( ( ( liver OR hepat* ) W/3 fat* ) ) OR TITLE-ABS-KEY ( ( ( liver OR hepat* ) PRE/3 fat* ) ) OR TITLE-ABS-KEY ( ( ( ( lipid OR triglyceride OR fat OR cholesterol ) W/3 ( accumulat* OR homeosta* OR metaboli* ) ) OR ( ( lipid OR triglyceride OR fat OR cholesterol ) PRE/3 ( accumulat* OR homeosta* OR metaboli* ) ) ) ) AND TITLE-ABS-KEY ( human ) AND TITLE-ABS-KEY ( ( ( perfluorooctanoic AND acid ) OR ( perfluorooctanoate ) OR fc-143 OR ( pentadecafluorooctanoic AND acid ) OR pfoa OR 3825-26-1 ) ) AND NOT TITLE-ABS-KEY ( ( nanopart* OR infect* OR mixture OR covid ) ) )

BPA **(20210106 17:10)**

( TITLE-ABS-KEY ( ( ( liver OR hepat* ) W/3 fat* ) ) OR TITLE-ABS-KEY ( ( ( liver OR hepat* ) PRE/3 fat* ) ) OR TITLE-ABS-KEY ( ( ( ( lipid OR triglyceride OR fat OR cholesterol ) W/3 ( accumulat* OR homeosta* OR metaboli* ) ) OR ( ( lipid OR triglyceride OR fat OR cholesterol ) PRE/3 ( accumulat* OR homeosta* OR metaboli* ) ) ) ) AND TITLE-ABS-KEY ( human ) AND TITLE-ABS-KEY ( ( ( bisphenol AND a ) OR p,p-isopropylidenebisphenol OR bpa OR 80-05-7 ) ) AND NOT TITLE-ABS-KEY ( ( nanopart* OR infect* OR mixture OR covid ) ) )

**TPP (20210106 17:15)**

( TITLE-ABS-KEY ( ( ( liver OR hepat* ) W/3 fat* ) ) OR TITLE-ABS-KEY ( ( ( liver OR hepat* ) PRE/3 fat* ) ) OR TITLE-ABS-KEY ( ( ( ( lipid OR triglyceride OR fat OR cholesterol ) W/3 ( accumulat* OR homeosta* OR metaboli* ) ) OR ( ( lipid OR triglyceride OR fat OR cholesterol ) PRE/3 ( accumulat* OR homeosta* OR metaboli* ) ) ) ) AND TITLE-ABS-KEY ( human ) AND TITLE-ABS-KEY ( ( ( triphenyl AND phosphate ) OR tpp OR 115-86-6 ) ) AND NOT TITLE-ABS-KEY ( ( nanopart* OR infect* OR mixture OR covid ) ) )

**Triclosan (20210106 17:18)**

( TITLE-ABS-KEY ( ( ( liver OR hepat* ) W/3 fat* ) ) OR TITLE-ABS-KEY ( ( ( liver OR hepat* ) PRE/3 fat* ) ) OR TITLE-ABS-KEY ( ( ( ( lipid OR triglyceride OR fat OR cholesterol ) W/3 ( accumulat* OR homeosta* OR metaboli* ) ) OR ( ( lipid OR triglyceride OR fat OR cholesterol ) PRE/3 ( accumulat* OR homeosta* OR metaboli* ) ) ) ) AND TITLE-ABS-KEY ( human ) AND TITLE-ABS-KEY ( ( ( triclosan ) OR ( lexol 300 ) OR ch-3565 OR ( trichloro-2'-hydroxydiphenyl AND ether ) OR 3380-34-5 ) ) AND NOT TITLE-ABS-KEY ( ( nanopart* OR infect* OR mixture OR cvid ) ) )

**DDE (20210106 17:21)**

( TITLE-ABS-KEY ( ( ( liver OR hepat* ) W/3 fat* ) ) OR TITLE-ABS-KEY ( ( ( liver OR hepat* ) PRE/3 fat* ) ) OR TITLE-ABS-KEY ( ( ( ( lipid OR triglyceride OR fat OR cholesterol ) W/3 ( accumulat* OR homeosta* OR metaboli* ) ) OR ( ( lipid OR triglyceride OR fat OR cholesterol ) PRE/3 ( accumulat* OR homeosta* OR metaboli* ) ) ) ) AND TITLE-ABS-KEY ( human ) AND TITLE-ABS-KEY ( ( dichlorodiphenyldichloroethylene OR dde OR 72-55-9 ) ) AND NOT TITLE-ABS-KEY ( ( nanopart* OR infect* OR mixture OR cvid ) ) )

**Conazoles 20210107 17:40**

( TITLE-ABS-KEY ( ( ( liver OR hepat* ) W/3 fat* ) ) OR TITLE-ABS-KEY ( ( ( liver OR hepat* ) PRE/3 fat* ) ) OR TITLE-ABS-KEY ( ( ( ( lipid OR triglyceride OR fat OR cholesterol ) W/3 ( accumulat* OR homeosta* OR metaboli* ) ) OR ( ( lipid OR triglyceride OR fat OR cholesterol ) PRE/3 ( accumulat* OR homeosta* OR metaboli* ) ) ) ) AND TITLE-ABS-KEY ( human ) AND TITLE-ABS-KEY ( ( *conazole OR cyproconazole OR 94361-06-5 OR ( cipac AND 600 ) OR tebuconazole OR 107534-96-3 OR ( triazole AND fungicide ) ) ) AND NOT TITLE-ABS-KEY ( ( nanopart* OR infect* OR mixture OR cvid ) ) )

**BaP (20210107 17:46)**

( TITLE-ABS-KEY ( ( ( liver OR hepat* ) W/3 fat* ) ) OR TITLE-ABS-KEY ( ( ( liver OR hepat* ) PRE/3 fat* ) ) OR TITLE-ABS-KEY ( ( ( ( lipid OR triglyceride OR fat OR cholesterol ) W/3 ( accumulat* OR homeosta* OR metaboli* ) ) OR ( ( lipid OR triglyceride OR fat OR cholesterol ) PRE/3 ( accumulat* OR homeosta* OR metaboli* ) ) ) ) AND TITLE-ABS-KEY ( human ) AND TITLE-ABS-KEY ( ( "Benzo[a]pyrene" OR 50-32-8 OR benzo?pyrene OR "3,4-Benzopyrene" ) ) AND NOT TITLE-ABS-KEY ( ( nanopart* OR infect* OR mixture OR cvid ) ) )

**Pemafibrate (20210107 17:54)**

( TITLE-ABS-KEY ( ( ( liver OR hepat* ) W/3 fat* ) ) OR TITLE-ABS-KEY ( ( ( liver OR hepat* ) PRE/3 fat* ) ) OR TITLE-ABS-KEY ( ( ( ( lipid OR triglyceride OR fat OR cholesterol ) W/3 ( accumulat* OR homeosta* OR metaboli* ) ) OR ( ( lipid OR triglyceride OR fat OR cholesterol ) PRE/3 ( accumulat* OR homeosta* OR metaboli* ) ) ) ) AND TITLE-ABS-KEY ( human ) AND TITLE-ABS-KEY ( ( pemafibrate OR 848259-27-8 OR "UNII-17VGG92R23" ) ) AND NOT TITLE-ABS-KEY ( ( nanopart* OR infect* OR mixture OR cvid ) ) )

**Lanifibranor (20210107 18:00)**

( TITLE-ABS-KEY ( ( ( liver OR hepat* ) W/3 fat* ) ) OR TITLE-ABS-KEY ( ( ( liver OR hepat* ) PRE/3 fat* ) ) OR TITLE-ABS-KEY ( ( ( ( lipid OR triglyceride OR fat OR cholesterol ) W/3 ( accumulat* OR homeosta* OR metaboli* ) ) OR ( ( lipid OR triglyceride OR fat OR cholesterol ) PRE/3 ( accumulat* OR homeosta* OR metaboli* ) ) ) ) AND TITLE-ABS-KEY ( human ) AND TITLE-ABS-KEY ( ( lanifibranor OR 927961-18-0 OR "IVA-337" OR iva337 OR "UNII-28Q8AG0PYL" ) ) AND NOT TITLE-ABS-KEY ( ( nanopart* OR infect* OR mixture OR cvid ) ) )

**Rosiglitazone 20210107 18:09**

( TITLE-ABS-KEY ( ( ( liver OR hepat* ) W/3 fat* ) ) OR TITLE-ABS-KEY ( ( ( liver OR hepat* ) PRE/3 fat* ) ) OR TITLE-ABS-KEY ( ( ( ( lipid OR triglyceride OR fat OR cholesterol ) W/3 ( accumulat* OR homeosta* OR metaboli* ) ) OR ( ( lipid OR triglyceride OR fat OR cholesterol ) PRE/3 ( accumulat* OR homeosta* OR metaboli* ) ) ) ) AND TITLE-ABS-KEY ( human ) AND TITLE-ABS-KEY ( ( rosiglitazone OR 122320-73-4 OR "Brl-49653" OR avandia ) ) AND NOT TITLE-ABS-KEY ( ( nanopart* OR infect* OR mixture OR covid ) ) ) AND ( LIMIT-TO ( DOCTYPE , "ar" ) OR LIMIT-TO ( DOCTYPE , "sh" ) ) AND ( LIMIT-TO ( LANGUAGE , "English" ) ) AND ( LIMIT-TO ( FREETOREAD , "all" ) )

**Saroglitazar 20210107 18:12**

( TITLE-ABS-KEY ( ( ( liver OR hepat* ) W/3 fat* ) ) OR TITLE-ABS-KEY ( ( ( liver OR hepat* ) PRE/3 fat* ) ) OR TITLE-ABS-KEY ( ( ( ( lipid OR triglyceride OR fat OR cholesterol ) W/3 ( accumulat* OR homeosta* OR metaboli* ) ) OR ( ( lipid OR triglyceride OR fat OR cholesterol ) PRE/3 ( accumulat* OR homeosta* OR metaboli* ) ) ) ) AND TITLE-ABS-KEY ( human ) AND TITLE-ABS-KEY ( ( saroglitazar OR 495399-09-2 OR "UNII-E0YMX3S4JD" OR lipaglyn ) ) AND NOT TITLE-ABS-KEY ( ( nanopart* OR infect* OR mixture OR covid ) ) )

**PFOS (20210107 18:17)**

( TITLE-ABS-KEY ( ( ( liver OR hepat* ) W/3 fat* ) ) OR TITLE-ABS-KEY ( ( ( liver OR hepat* ) PRE/3 fat* ) ) OR TITLE-ABS-KEY ( ( ( ( lipid OR triglyceride OR fat OR cholesterol ) W/3 ( accumulat* OR homeosta* OR metaboli* ) ) OR ( ( lipid OR triglyceride OR fat OR cholesterol ) PRE/3 ( accumulat* OR homeosta* OR metaboli* ) ) ) ) AND TITLE-ABS-KEY ( human ) AND TITLE-ABS-KEY ( ( pfos OR 1763-23-1 OR "Perfluorooctanesulfonic acid" OR "Perfluorooctane sulfonate" OR "Perfluorooctane sulfonic acid" ) ) AND NOT TITLE-ABS-KEY ( ( nanopart* OR infect* OR mixture OR covid ) ) )

**DEHP 20210107 18:20**

( TITLE-ABS-KEY ( ( ( liver OR hepat* ) W/3 fat* ) ) OR TITLE-ABS-KEY ( ( ( liver OR hepat* ) PRE/3 fat* ) ) OR TITLE-ABS-KEY ( ( ( ( lipid OR triglyceride OR fat OR cholesterol ) W/3 ( accumulat* OR homeosta* OR metaboli* ) ) OR ( ( lipid OR triglyceride OR fat OR cholesterol ) PRE/3 ( accumulat* OR homeosta* OR metaboli* ) ) ) ) AND TITLE-ABS-KEY ( human ) AND TITLE-ABS-KEY ( ( "Bis(2-ethylhexyl) phthalate" OR 117-81-7 OR dehp OR "Di(2-ethylhexyl) phthalate" OR ( diethylhexyl AND phthalate ) ) ) AND NOT TITLE-ABS-KEY ( ( nanopart* OR infect* OR mixture OR covid ) ) )

**DHA 20210107 18:28**

( TITLE-ABS-KEY ( ( ( liver OR hepat* ) W/3 fat* ) ) OR TITLE-ABS-KEY ( ( ( liver OR hepat* ) PRE/3 fat* ) ) OR TITLE-ABS-KEY ( ( ( ( lipid OR triglyceride OR fat OR cholesterol ) W/3 ( accumulat* OR homeosta* OR metaboli* ) ) OR ( ( lipid OR triglyceride OR fat OR cholesterol ) PRE/3 ( accumulat* OR homeosta* OR metaboli* ) ) ) ) AND TITLE-ABS-KEY ( human ) AND TITLE-ABS-KEY ( ( "Docosahexaenoic acid" OR 6217-54-5 OR doconexent OR "cervonic acid" ) ) AND NOT TITLE-ABS-KEY ( ( nanopart* OR infect* OR mixture OR covid ) ) ) AND ( LIMIT-TO ( DOCTYPE , "ar" ) ) AND ( LIMIT-TO ( SUBJAREA , "MEDI" ) OR LIMIT-TO ( SUBJAREA , "BIOC" ) OR LIMIT-TO ( SUBJAREA , "PHAR" ) OR LIMIT-TO ( SUBJAREA , "IMMU" ) ) AND ( LIMIT-TO ( FREETOREAD , "all" ) ) AND ( LIMIT-TO ( LANGUAGE , "English" ) )

**Choline – limited 20210107 18:33**

( TITLE-ABS-KEY ( ( ( liver OR hepat* ) W/3 fat* ) ) OR TITLE-ABS-KEY ( ( ( liver OR hepat* ) PRE/3 fat* ) ) OR TITLE-ABS-KEY ( ( ( ( lipid OR triglyceride OR fat OR cholesterol ) W/3 ( accumulat* OR homeosta* OR metaboli* ) ) OR ( ( lipid OR triglyceride OR fat OR cholesterol ) PRE/3 ( accumulat* OR homeosta* OR metaboli* ) ) ) ) AND TITLE-ABS-KEY ( human ) AND TITLE-ABS-KEY ( ( choline OR 62-49-7 OR bilineurine ) ) AND NOT TITLE-ABS-KEY ( ( nanopart* OR infect* OR mixture OR covid ) ) ) AND ( LIMIT-TO ( FREETOREAD , "all" ) ) AND ( LIMIT-TO ( DOCTYPE , "ar" ) OR LIMIT-TO ( DOCTYPE , "sh" ) ) AND ( LIMIT-TO ( LANGUAGE , "English" ) )

**Metformin – limited 20210107 18:37**

( TITLE-ABS-KEY ( ( ( liver OR hepat* ) W/3 fat* ) ) OR TITLE-ABS-KEY ( ( ( liver OR hepat* ) PRE/3 fat* ) ) OR TITLE-ABS-KEY ( ( ( ( lipid OR triglyceride OR fat OR cholesterol ) W/3 ( accumulat* OR homeosta* OR metaboli* ) ) OR ( ( lipid OR triglyceride OR fat OR cholesterol ) PRE/3 ( accumulat* OR homeosta* OR metaboli* ) ) ) ) AND TITLE-ABS-KEY ( human ) AND TITLE-ABS-KEY ( ( metformin* OR 657-24-9 OR "1,1-Dimethylbiguanide" OR metiguanide ) ) AND NOT TITLE-ABS-KEY ( ( nanopart* OR infect* OR mixture OR covid ) ) ) AND ( LIMIT-TO ( FREETOREAD , "all" ) ) AND ( LIMIT-TO ( DOCTYPE , "ar" ) ) AND ( LIMIT-TO ( LANGUAGE , "English" ) )

**Valproic acid – limited 20210107 18:47**

( TITLE-ABS-KEY ( ( ( liver OR hepat* ) W/3 fat* ) ) OR TITLE-ABS-KEY ( ( ( liver OR hepat* ) PRE/3 fat* ) ) OR TITLE-ABS-KEY ( ( ( ( lipid OR triglyceride OR fat OR cholesterol ) W/3 ( accumulat* OR homeosta* OR metaboli* ) ) OR ( ( lipid OR triglyceride OR fat OR cholesterol ) PRE/3 ( accumulat* OR homeosta* OR metaboli* ) ) ) ) AND TITLE-ABS-KEY ( human ) AND TITLE-ABS-KEY ( ( "valproic acid" OR valproate OR 99-66-1 OR "2-propylvaleric acid" OR dipropylacet* ) ) AND NOT TITLE-ABS-KEY ( ( nanopart* OR infect* OR mixture OR covid ) ) ) AND ( LIMIT-TO ( DOCTYPE , "ar" ) OR LIMIT-TO ( DOCTYPE , "sh" ) OR LIMIT-TO ( DOCTYPE , "no" ) ) AND ( LIMIT-TO ( FREETOREAD , "all" ) ) AND ( LIMIT-TO ( LANGUAGE , "English" ) )

**Resveratrol – limited 20210107 18:52**

( TITLE-ABS-KEY ( ( ( liver OR hepat* ) W/3 fat* ) ) OR TITLE-ABS-KEY ( ( ( liver OR hepat* ) PRE/3 fat* ) ) OR TITLE-ABS-KEY ( ( ( ( lipid OR triglyceride OR fat OR cholesterol ) W/3 ( accumulat* OR homeosta* OR metaboli* ) ) OR ( ( lipid OR triglyceride OR fat OR cholesterol ) PRE/3 ( accumulat* OR homeosta* OR metaboli* ) ) ) ) AND TITLE-ABS-KEY ( human ) AND TITLE-ABS-KEY ( ( resveratrol OR 501-36-0 OR "3,4',5-Trihydroxystilbene" ) ) AND NOT TITLE-ABS-KEY ( ( nanopart* OR infect* OR mixture OR covid ) ) ) AND ( LIMIT-TO ( FREETOREAD , "all" ) ) AND ( LIMIT-TO ( DOCTYPE , "ar" ) OR LIMIT-TO ( DOCTYPE , "sh" ) ) AND ( LIMIT-TO ( LANGUAGE , "English" ) )

**2-oxoglutaric acid 20210108 10:44**

( TITLE-ABS-KEY ( ( ( liver OR hepat* ) W/3 fat* ) ) OR TITLE-ABS-KEY ( ( ( liver OR hepat* ) PRE/3 fat* ) ) OR TITLE-ABS-KEY ( ( ( ( lipid OR triglyceride OR fat OR cholesterol ) W/3 ( accumulat* OR homeosta* OR metaboli* ) ) OR ( ( lipid OR triglyceride OR fat OR cholesterol ) PRE/3 ( accumulat* OR homeosta* OR metaboli* ) ) ) ) AND TITLE-ABS-KEY ( human ) AND TITLE-ABS-KEY ( ( 2-oxoglutar* OR 2-ketoglutar* OR a-ketoglutar* OR 328-50-7 ) ) AND NOT TITLE-ABS-KEY ( ( nanopart* OR infect* OR mixture OR covid ) ) )

**Doxepin 20210108 10:50**

( TITLE-ABS-KEY ( ( ( liver OR hepat* ) W/3 fat* ) ) OR TITLE-ABS-KEY ( ( ( liver OR hepat* ) PRE/3 fat* ) ) OR TITLE-ABS-KEY ( ( ( ( lipid OR triglyceride OR fat OR cholesterol ) W/3 ( accumulat* OR homeosta* OR metaboli* ) ) OR ( ( lipid OR triglyceride OR fat OR cholesterol ) PRE/3 ( accumulat* OR homeosta* OR metaboli* ) ) ) ) AND TITLE-ABS-KEY ( human ) AND TITLE-ABS-KEY ( ( doxepin* OR sinequan OR 1668-19-5 ) ) AND NOT TITLE-ABS-KEY ( ( nanopart* OR infect* OR mixture OR covid ) ) )

**Caffeine 20210108 10:53**

( TITLE-ABS-KEY ( ( ( liver OR hepat* ) W/3 fat* ) ) OR TITLE-ABS-KEY ( ( ( liver OR hepat* ) PRE/3 fat* ) ) OR TITLE-ABS-KEY ( ( ( ( lipid OR triglyceride OR fat OR cholesterol ) W/3 ( accumulat* OR homeosta* OR metaboli* ) ) OR ( ( lipid OR triglyceride OR fat OR cholesterol ) PRE/3 ( accumulat* OR homeosta* OR metaboli* ) ) ) ) AND TITLE-ABS-KEY ( human ) AND TITLE-ABS-KEY ( ( caffeine OR 58-08-2 OR thein OR guaranine OR methyltheobromine OR trimethylxanthine ) ) AND NOT TITLE-ABS-KEY ( ( nanopart* OR infect* OR mixture OR covid ) ) )

**GW3965 20210108 10:57**

( TITLE-ABS-KEY ( ( ( liver OR hepat* ) W/3 fat* ) ) OR TITLE-ABS-KEY ( ( ( liver OR hepat* ) PRE/3 fat* ) ) OR TITLE-ABS-KEY ( ( ( ( lipid OR triglyceride OR fat OR cholesterol ) W/3 ( accumulat* OR homeosta* OR metaboli* ) ) OR ( ( lipid OR triglyceride OR fat OR cholesterol ) PRE/3 ( accumulat* OR homeosta* OR metaboli* ) ) ) ) AND TITLE-ABS-KEY ( human ) AND TITLE-ABS-KEY ( ( gw3965 OR 405911-09-3 OR gw?3965 OR "3-(3-(N-(2-Chloro-3-trifluoromethylbenzyl)(2,2-diphenylethyl)amino)propoxy)phenylacetic acid" ) ) AND NOT TITLE-ABS-KEY ( ( nanopart* OR infect* OR mixture OR covid ) ) )

**Niacin 20210108 11:02**

( TITLE-ABS-KEY ( ( ( liver OR hepat* ) W/3 fat* ) ) OR TITLE-ABS-KEY ( ( ( liver OR hepat* ) PRE/3 fat* ) ) OR TITLE-ABS-KEY ( ( ( ( lipid OR triglyceride OR fat OR cholesterol ) W/3 ( accumulat* OR homeosta* OR metaboli* ) ) OR ( ( lipid OR triglyceride OR fat OR cholesterol ) PRE/3 ( accumulat* OR homeosta* OR metaboli* ) ) ) ) AND TITLE-ABS-KEY ( human ) AND TITLE-ABS-KEY ( ( niacin OR "nicotininc acid" OR 59-67-6 OR 3-pyridinecarboxylic AND acid OR pyridine-3-carboxylic AND acid ) ) AND NOT TITLE-ABS-KEY ( ( nanopart* OR infect* OR mixture OR covid ) ) )

**Acetaminophen 20210108 11:18**

( TITLE-ABS-KEY ( ( ( liver OR hepat* ) W/3 fat* ) ) OR TITLE-ABS-KEY ( ( ( liver OR hepat* ) PRE/3 fat* ) ) OR TITLE-ABS-KEY ( ( ( ( lipid OR triglyceride OR fat OR cholesterol ) W/3 ( accumulat* OR homeosta* OR metaboli* ) ) OR ( ( lipid OR triglyceride OR fat OR cholesterol ) PRE/3 ( accumulat* OR homeosta* OR metaboli* ) ) ) ) AND TITLE-ABS-KEY ( human ) AND TITLE-ABS-KEY ( ( acetaminophen OR paracetamol OR 103-90-2 OR tylenol OR 4-acetamidophenol ) ) AND NOT TITLE-ABS-KEY ( ( nanopart* OR infect* OR mixture OR covid ) ) )

**6,6'-di-tert-butyl-4,4'-butylidenedi-m-cresol (santowhite) 20210108 11:34 (no documents retrieved)**

( TITLE-ABS-KEY ( ( ( liver OR hepat* ) W/3 fat* ) ) OR TITLE-ABS-KEY ( ( ( liver OR hepat* ) PRE/3 fat* ) ) OR TITLE-ABS-KEY ( ( ( ( lipid OR triglyceride OR fat OR cholesterol ) W/3 ( accumulat* OR homeosta* OR metaboli* ) ) OR ( ( lipid OR triglyceride OR fat OR cholesterol ) PRE/3 ( accumulat* OR homeosta* OR metaboli* ) ) ) ) AND TITLE-ABS-KEY ( human ) AND TITLE-ABS-KEY ( ( 85-60-9 OR santowhite OR sumilit OR "6,6'-di-tert-butyl-4,4'-butylidenedi-m-cresol" OR "Annullex PBA 15" ) ) AND NOT TITLE-ABS-KEY ( ( nanopart* OR infect* OR mixture OR covid ) ) )

**Chlormequat 20210108 11:40 (only 1 hit – Lichtenstein 2020)**

( TITLE-ABS-KEY ( ( ( liver OR hepat* ) W/3 fat* ) ) OR TITLE-ABS-KEY ( ( ( liver OR hepat* ) PRE/3 fat* ) ) OR TITLE-ABS-KEY ( ( ( ( lipid OR triglyceride OR fat OR cholesterol ) W/3 ( accumulat* OR homeosta* OR metaboli* ) ) OR ( ( lipid OR triglyceride OR fat OR cholesterol ) PRE/3 ( accumulat* OR homeosta* OR metaboli* ) ) ) ) AND TITLE-ABS-KEY ( human ) AND TITLE-ABS-KEY ( ( chlormequat OR 7003-89-6 OR 999-81-5 OR "2-Chloro-N,N,N-trimethylethanaminium" OR "chlorocholine chloride" ) ) AND NOT TITLE-ABS-KEY ( ( nanopart* OR infect* OR mixture OR covid ) ) )

**Chlorpyrifos 20210108 11:45**

( TITLE-ABS-KEY ( ( ( liver OR hepat* ) W/3 fat* ) ) OR TITLE-ABS-KEY ( ( ( liver OR hepat* ) PRE/3 fat* ) ) OR TITLE-ABS-KEY ( ( ( ( lipid OR triglyceride OR fat OR cholesterol ) W/3 ( accumulat* OR homeosta* OR metaboli* ) ) OR ( ( lipid OR triglyceride OR fat OR cholesterol ) PRE/3 ( accumulat* OR homeosta* OR metaboli* ) ) ) ) AND TITLE-ABS-KEY ( human ) AND TITLE-ABS-KEY ( ( chlorpyrifos OR 2921-88-2 OR chlorpyriphos ) ) AND NOT TITLE-ABS-KEY ( ( nanopart* OR infect* OR mixture OR covid ) ) )

Thiacloprid 20210108 11:49

( TITLE-ABS-KEY ( ( ( liver OR hepat* ) W/3 fat* ) ) OR TITLE-ABS-KEY ( ( ( liver OR hepat* ) PRE/3 fat* ) ) OR TITLE-ABS-KEY ( ( ( ( lipid OR triglyceride OR fat OR cholesterol ) W/3 ( accumulat* OR homeosta* OR metaboli* ) ) OR ( ( lipid OR triglyceride OR fat OR cholesterol ) PRE/3 ( accumulat* OR homeosta* OR metaboli* ) ) ) ) AND TITLE-ABS-KEY ( human ) AND TITLE-ABS-KEY ( ( thiacloprid OR 111988-49-9 OR calypso OR "UNII-DSV3A944A4" ) ) AND NOT TITLE-ABS-KEY ( ( nanopart* OR infect* OR mixture OR covid ) ) )

Thiamethoxam 20210108 11:51 (only 1hit – Mesnage 2018 neonicotinoid insecticides)

( TITLE-ABS-KEY ( ( ( liver OR hepat* ) W/3 fat* ) ) OR TITLE-ABS-KEY ( ( ( liver OR hepat* ) PRE/3 fat* ) ) OR TITLE-ABS-KEY ( ( ( ( lipid OR triglyceride OR fat OR cholesterol ) W/3 ( accumulat* OR homeosta* OR metaboli* ) ) OR ( ( lipid OR triglyceride OR fat OR cholesterol ) PRE/3 ( accumulat* OR homeosta* OR metaboli* ) ) ) ) AND TITLE-ABS-KEY ( human ) AND TITLE-ABS-KEY ( ( thiamethoxam OR actara OR 153719-23-4 OR diacloden ) ) AND NOT TITLE-ABS-KEY ( ( nanopart* OR infect* OR mixture OR covid ) ) )

Acetamiprid 20210108 11:54 (no documents found)

( TITLE-ABS-KEY ( ( ( liver OR hepat* ) W/3 fat* ) ) OR TITLE-ABS-KEY ( ( ( liver OR hepat* ) PRE/3 fat* ) ) OR TITLE-ABS-KEY ( ( ( ( lipid OR triglyceride OR fat OR cholesterol ) W/3 ( accumulat* OR homeosta* OR metaboli* ) ) OR ( ( lipid OR triglyceride OR fat OR cholesterol ) PRE/3 ( accumulat* OR homeosta* OR metaboli* ) ) ) ) AND TITLE-ABS-KEY ( human ) AND TITLE-ABS-KEY ( ( acetamiprid OR 135410-20-7 OR 160430-64-8 OR mospilan ) ) AND NOT TITLE-ABS-KEY ( ( nanopart* OR infect* OR mixture OR covid ) ) )

Thiram 20210108 11:56

( TITLE-ABS-KEY ( ( ( liver OR hepat* ) W/3 fat* ) ) OR TITLE-ABS-KEY ( ( ( liver OR hepat* ) PRE/3 fat* ) ) OR TITLE-ABS-KEY ( ( ( ( lipid OR triglyceride OR fat OR cholesterol ) W/3 ( accumulat* OR homeosta* OR metaboli* ) ) OR ( ( lipid OR triglyceride OR fat OR cholesterol ) PRE/3 ( accumulat* OR homeosta* OR metaboli* ) ) ) ) AND TITLE-ABS-KEY ( human ) AND TITLE-ABS-KEY ( ( thiram OR 137-26-8 OR thiuram OR tmtd OR "Tetramethylthiuram disulfide" ) ) AND NOT TITLE-ABS-KEY ( ( nanopart* OR infect* OR mixture OR covid ) ) )

Monoethyl phthalate 20220215 16:15

( TITLE-ABS-KEY ( ( ( liver OR hepat* ) W/3 fat* ) ) OR TITLE-ABS-KEY ( ( ( liver OR hepat* ) PRE/3 fat* ) ) OR TITLE-ABS-KEY ( ( ( ( lipid OR triglyceride OR fat OR cholesterol ) W/3 ( accumulat* OR homeosta* OR metaboli* ) ) OR ( ( lipid OR triglyceride OR fat OR cholesterol ) PRE/3 ( accumulat* OR homeosta* OR metaboli* ) ) ) ) AND TITLE-ABS-KEY ( human ) AND TITLE-ABS-KEY ( ( "monoethyl phthalate" OR 2306-33-4 OR "benzenedicarboxylic acid" OR mehp ) ) AND NOT TITLE-ABS-KEY ( ( nanopart* OR infect* OR mixture OR covid ) ) )

Rotenone 20220215 16:16

( TITLE-ABS-KEY ( ( ( liver OR hepat* ) W/3 fat* ) ) OR TITLE-ABS-KEY ( ( ( liver OR hepat* ) PRE/3 fat* ) ) OR TITLE-ABS-KEY ( ( ( ( lipid OR triglyceride OR fat OR cholesterol ) W/3 ( accumulat* OR homeosta* OR metaboli* ) ) OR ( ( lipid OR triglyceride OR fat OR cholesterol ) PRE/3 ( accumulat* OR homeosta* OR metaboli* ) ) ) ) AND TITLE-ABS-KEY ( human ) AND TITLE-ABS-KEY ( ( rotenone OR 83-79-4 ) ) AND NOT TITLE-ABS-KEY ( ( nanopart* OR infect* OR mixture OR covid ) ) )

GW3965 20220215 16:22

( TITLE-ABS-KEY ( ( ( liver OR hepat* ) W/3 fat* ) ) OR TITLE-ABS-KEY ( ( ( liver OR hepat* ) PRE/3 fat* ) ) OR TITLE-ABS-KEY ( ( ( ( lipid OR triglyceride OR fat OR cholesterol ) W/3 ( accumulat* OR homeosta* OR metaboli* ) ) OR ( ( lipid OR triglyceride OR fat OR cholesterol ) PRE/3 ( accumulat* OR homeosta* OR metaboli* ) ) ) ) AND TITLE-ABS-KEY ( human ) AND TITLE-ABS-KEY ( ( gw3965 OR 405911-17-3 OR "3-[3-[N-(2-Chloro-3-trifluoromethylbenzyl)-(2,2-diphenylethyl)amino]propyloxy]phenylacetic acid hydrochloride" ) ) AND NOT TITLE-ABS-KEY ( ( nanopart* OR infect* OR mixture OR covid ) ) )

Acetamiprid 20220215 16:25

( TITLE-ABS-KEY ( ( ( liver OR hepat* ) W/3 fat* ) ) OR TITLE-ABS-KEY ( ( ( liver OR hepat* ) PRE/3 fat* ) ) OR TITLE-ABS-KEY ( ( ( ( lipid OR triglyceride OR fat OR cholesterol ) W/3 ( accumulat* OR homeosta* OR metaboli* ) ) OR ( ( lipid OR triglyceride OR fat OR cholesterol ) PRE/3 ( accumulat* OR homeosta* OR metaboli* ) ) ) ) AND TITLE-ABS-KEY ( human ) AND TITLE-ABS-KEY ( ( acetamiprid OR "N-(6-Chloro-3-pyridylmethyl)-N′-cyano-acetamidine" OR 190604-92-3 ) ) AND NOT TITLE-ABS-KEY ( ( nanopart* OR infect* OR mixture OR cvid ) ) )

Fructose 20220215 16:30

( TITLE-ABS-KEY ( ( ( liver OR hepat* ) W/3 fat* ) ) OR TITLE-ABS-KEY ( ( ( liver OR hepat* ) PRE/3 fat* ) ) OR TITLE-ABS-KEY ( ( ( ( lipid OR triglyceride OR fat OR cholesterol ) W/3 ( accumulat* OR homeosta* OR metaboli* ) ) OR ( ( lipid OR triglyceride OR fat OR cholesterol ) PRE/3 ( accumulat* OR homeosta* OR metaboli* ) ) ) ) AND TITLE-ABS-KEY ( human ) AND TITLE-ABS-KEY ( ( fructose OR 57-48-7 OR "(3S,4R,5R)-1,3,4,5,6-Pentahydroxyhexan-2-one" OR levulose OR fructofuranose OR "arabino-hexulose" ) ) AND NOT TITLE-ABS-KEY ( ( nanopart* OR infect* OR mixture OR cvid ) ) ) AND ( LIMIT-TO ( OA , "publisherfullgold" ) OR LIMIT-TO ( OA , "publisherhybridgold" ) ) AND ( LIMIT-TO ( DOCTYPE , "ar" ) ) AND ( LIMIT-TO ( LANGUAGE , "English" ) ) AND ( LIMIT-TO ( SRCTYPE , "j" ) )

Rifampicin 20220215 16:33

( TITLE-ABS-KEY ( ( ( liver OR hepat* ) W/3 fat* ) ) OR TITLE-ABS-KEY ( ( ( liver OR hepat* ) PRE/3 fat* ) ) OR TITLE-ABS-KEY ( ( ( ( lipid OR triglyceride OR fat OR cholesterol ) W/3 ( accumulat* OR homeosta* OR metaboli* ) ) OR ( ( lipid OR triglyceride OR fat OR cholesterol ) PRE/3 ( accumulat* OR homeosta* OR metaboli* ) ) ) ) AND TITLE-ABS-KEY ( human ) AND TITLE-ABS-KEY ( ( rifampicin OR 13292-46-1 OR rifadin ) ) AND NOT TITLE-ABS-KEY ( ( nanopart* OR infect* OR mixture OR cvid ) ) )

Oxysterols 20220215 16:42

( ( TITLE-ABS-KEY ( ( ( liver OR hepat* ) W/3 fat* ) ) OR TITLE-ABS-KEY ( ( ( liver OR hepat* ) PRE/3 fat* ) ) OR TITLE-ABS-KEY ( ( ( ( lipid OR triglyceride OR fat OR cholesterol ) W/3 ( accumulat* OR homeosta* OR metaboli* ) ) OR ( ( lipid OR triglyceride OR fat OR cholesterol ) PRE/3 ( accumulat* OR homeosta* OR metaboli* ) ) ) ) AND TITLE-ABS-KEY ( human ) AND TITLE-ABS-KEY ( ( oxysterol OR hydroxysterol OR hydrocholesterol ) ) AND NOT TITLE-ABS-KEY ( ( nanopart* OR infect* OR mixture OR cvid ) ) ) ) AND ( steatosis OR "lipid accumulation" OR triglyceride )

Tetracycline 20220215 16:51

( TITLE-ABS-KEY ( ( ( liver OR hepat* ) W/3 fat* ) ) OR TITLE-ABS-KEY ( ( ( liver OR hepat* ) PRE/3 fat* ) ) OR TITLE-ABS-KEY ( ( ( ( lipid OR triglyceride OR fat OR cholesterol ) W/3 ( accumulat* OR homeosta* OR metaboli* ) ) OR ( ( lipid OR triglyceride OR fat OR cholesterol ) PRE/3 ( accumulat* OR homeosta* OR metaboli* ) ) ) ) AND TITLE-ABS-KEY ( human ) AND TITLE-ABS-KEY ( ( tetracycline OR sumycin OR 60-54-8 OR 64-75-5 ) ) AND NOT TITLE-ABS-KEY ( ( nanopart* OR infect* OR mixture OR cvid ) ) )
