# Supplementary material for: Development of a reference and proficiency chemical list for human steatosis endpoints in vitro
Source: Front Endocrinol (Lausanne). 2023 Apr 24;14:1126880. doi: 10.3389/fendo.2023.1126880 (PMC10166001; doi:10.3389/fendo.2023.1126880)
Supplement: Supplementary file 2 [file Table_1.docx]

Supplementary Material 2

Supplementary Table : Evaluation of chemicals to be proposed as preliminary reference and proficiency chemicals for (pre-)validation of an *in vitro* human steatosis test method. p≤0.05 is considered statistically significant unless stated otherwise.

| **Chemical** | **CAS No.** | **Structure** | **Use** | **Model** | **Triglyceride accumulation in relation to steatosis** | **Interaction with key pathways/ receptors/ mechanisms** | **ref** | **Comments/ inclusion in test method preliminary proficiency chemical set ?** | **Quality of the study/relevance** (and comments on potential biases or limitations) |
| --- | --- | --- | --- | --- | --- | --- | --- | --- | --- |
| **Perfluorooctanoic acid (PFOA)** | 3825-26-1 | 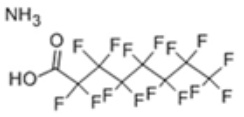 | Industrial chemical, non-stick coating | **SUMMARY** | **Negative/weak inducer (in human)**  **More potent in rodents** | PPARα, **ERα** |  | **Conflicting evidence from (human) in vitro and in vivo (human epidemiology and rodent) studies.** Mechanistically, strong evidence supports PFOA acting as a (weak-moderate) PPARα agonist, therefore supporting a plausible mechanism for interfering with lipid metabolism, including in the liver.  However, differences in the affinity of PFOA to murine and human PPARα have been described (weaker affinity in humans). Therefore, rodent studies might overestimate the steatosis hazard potential through PPARα.  Data from human epidemiological studies indicate changes in blood/serum (Liver enzyme activity, cholesterol or triglyceride levels), but these are not consistent across studies. No studies reported on hepatic lipid accumulation (either not reported or not observed), but this is difficult to extrapolate from epidemiological studies/blood samples. Nevertheless, liver weight gain is identified as the occupational human health hazard with the smallest margin of exposure (Butenhoff et al., 2004).  PFOA is listed under the Stockholm Convention on Persistent Organic Pollutants, Annex A: Elimination (UNEP, 2019). | Will contribute to evidence base for the development of suitable alternatives  Support from ongoing work at OECD and UK Environment Agency |
|  |  |  |  | Human, in vitro (differentiated HepaRG cells, 24-48 h, 100-750 µM) | Not concluded | Strong decrease of CYP7A1 expression (protein & genes assessed mostly related to bile acid/cholesterol synthesis, transport, detoxification) | (Behr et al., 2020) | PFOA and PFOS did not affect cholesterol levels but altered bile acid synthesis (strong effects at concentrations >10 µM). Suggested cholestatic effects. (Further: no 48 h-cytotoxicity in HepaRG up to 500 µM PFOA; PFOS toxic above 100 µM) |  |
|  |  |  |  | Human, in vitro (differentiated HepaRG cells, 72 h, 1.5×10^-8^ - 1×10^-3^ M) | Not assessed |  | (Landesmann et al., 2012) | PFOA (high concentrations, approx. 200 µM) induces ROS in differentiated HepaRG after 72 h exposure |  |
|  |  |  |  | Human, in vitro (differentiated HepaRG cells, 24 h, 3 d, 7d; 100 pM - 100 µM (cytotoxicity)/ 100 pM – 1 µM (metabolomics)) | Induction in differentiated HepaRG (human in vitro; above 1 nM) |  | (Franco et al., 2020) | PFOA induces steatosis in differentiated HepaRG cells (increased total lipids from 1 nM PFOA). PFOA was cytotoxic (24 h: <80 % viability at 100 µM ; 24 h LC50 = 145.30 +-10.70 µM, 7 d LC50 = 17.03 +-1.35 µM). Lipid homeostasis was shifted upon exposure in favour of obesity-related lipid species: di- and triglycerides. |  |
|  |  |  |  | Human, in vitro (human primary hepatocytes and HepG2, 1-100 µM, 5 min – 16 h)) | Not assessed (inferred: no induction) | PPARα, **ERα**, PPARγ, HNF4α | (Buhrke et al., 2015) | Microarray transcriptomic analysis.  In HepG2 cells, cell viability was significantly (p<0.001) decreased at ≥50 µM. Metabolic activity and cell proliferation were increased at ≥10 µM.  c-Jun, c-Fos, AP-1: altered gene expression, but this did not translate into functional tests/proteomics). PFOA stimulated cellular proliferation and metabolic activity of cells. PFOA-induced proliferation occurs largely independent of c-Jun and c-Fos.  PPARα activation (and downstream regulation of lipid metabolism in human hepatocytes) is only induced at high concentrations of PFOA, described as “non-physiological”. | High confidence, moderate relevance |
|  |  |  |  | Human, epidemiology (n=200) |  | HNF4α, PXR, CAR, LXR, **PPARα**/γ, FXR, (**ERα**) | (Bassler et al., 2019)) | Epidemiology (n=200) (Bassler et al., 2019): serum PFOA was associated with hepatocyte apoptosis (CK18 M30 increased), and inflammation (IFNγ increased, but TNFα decreased). Potential for sex differences in adipocytokine and complement (C3a) responses (possibly via ERα) |  |
|  |  |  |  | Human, epidemiology (n=222) | Potentially induction; elevated blood lipids and triglycerides |  | (Spratlen et al., 2020): | Prenatal PFOA exposure was associated with higher cord blood lipids and triglycerides (but not cholesterol) |  |
|  |  |  |  | Human, epidemiology (nested cohort; n=753; 663 male, 90 female) | Not concluded |  | (Eriksen et al., 2013) | positive association between plasma PFOA/PFOS and cholesterol (stronger for PFOS than for PFOA). Low-level exposed population. |  |
|  |  |  |  | Human, epidemiology (n=32,254) | No |  | (Darrow et al., 2016) | Modelled cumulative serum PFOA was associated with increased ALT levels, indicative of hepatotoxicity, but not with liver disease (including and specifically: no enlarged liver, fatty liver, cirrhosis) |  |
|  |  |  |  | Human, in vivo (longitudinal occupational assessment. n=179) | No assessment of hepatic lipids (but inferred no induction of steatosis, from blood biochemistry/ liver enzymes) |  | (Olsen et al., 2012) | Of initially subscribed 204 participants, 179 workers who did not take lipid-lowering medication were included in the study.  Adverse associations were not observed between changes in PFOA/ PFOS and non-HDL cholesterol, HDL, or hepatic clinical chemistries | Authors with affiliation in chemical industry |
|  |  |  |  | Review (Risk characterization for general population exposure) | Not assessed/concluded (but hazard potential for liver weight increase) |  | (Butenhoff et al., 2004) | PFOA serum level of children (n=645), adults (n=598), and the elderly (n=238) from the United States: upper bound of the 95^th^ percentile estimate in the range of 0.011–0.014 µg/mL (ppm). General population-serum concentrations associated with the lower 95% confidence limit of a modelled 10 percent response or incidence level (LBMIC10) using USEPA BMDS software were used for calculation of margin of exposure.  Liver weight increase LBMIC10: 23 µg/mL (based on monkey data), margin of exposure: 1600 body weight increase LBMIC10: 60 µg/mL (based on monkey data), margin of exposure: 4300  “Liver weight increase” is the endpoint with the lowest margin of exposure (of the investigated endpoints).  The authors conclude that “These MOE values represent substantial protection of children, adults, and the elderly.” | Authors with affiliation in chemical industry |
|  |  |  |  | Review | Induction (but not resulting in manifestation of adverse effects in humans, in vivo) | PPARα | (Kennedy Jr et al., 2004) | Increased β-oxidation of fatty acids increases in several CYP-mediated reactions, and inhibition of the secretion of very low-density lipoproteins and cholesterol from the liver.  Effects on lipid metabolism and transport result in decreased serum cholesterol and triglyceride levels, while simultaneously leading to accumulation of lipids in the liver.  PPARα activation inducing hepatocellular carcinoma in rats is likely not relevant in humans, due to differences in PPARα sensitivity between rats and humans.  Mention of sex differences in clearance of PFOA in rats (single dose of 25 mg/kg bw via gavage): while >95% clearance was observed in femae after 24 h, only 50% were cleared in males in the same time (77% clearance in males after 168 h). | High confidence and relevance |
|  |  |  |  | Mouse, in vivo (male C57BL/6 (H-2b) mice, 5-6 weeks old at beginning of experiment. 10 d exposure: 0.002-0.02% (w/w) PFOA in feed) | Not concluded/ assessed | PPARα, C3a (only in vivo, not in vitro) | (Botelho et al., 2015) | Complement activation is involved in PFOA-induced hepatic injury in mice  Mice were exposed in groups of 4 mice each, exposure groups: no PFOA added (control diet), or PFOA (w/w) content of: 0.002%, 0.005%, 0.01%, 0.02%. | High |
|  |  |  |  | Mouse, in vivo (adult male SV129 WT and PPARα-null mice. Oral gavage, 7 days 10 mg/kg/day) | yes |  | (Das et al., 2017) | FOA induced steatosis in WT mice, but did not significantly increase steatosis in PPARα-null mutants; β-oxidation of isolated liver mitochondria was not inhibited  Animals were exposed in groups of four. | High dose |
| **Perfluorooctanesulfonic acid (PFOS)** | 1763-23-1 | 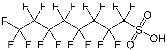 | Fluorosurfactant |  |  | PPARα (agonism) |  | PFOS is listed under the Stockholm Convention on Persistent Organic Pollutants, Annex B: Restriction of use (UNEP, 2019).  (Due to time constraints the literature search was not pursued further, as PFOA is a priority within the GOLIATH project and restrictions to marketing and use under the Stockholm POPs Convention apply.) |  |
| **Trib****utyltin chloride (TBT)** | 1461-22-9 | 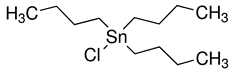 | Biocide (fungicide, molluscicide) | **SUMMARY** | **Tentative positive**  **Induction (potential)** | RXR |  | **Moderate-weak weight of evidence** **supporting TBT to** **induce hepatic steatosis** in rodent in vivo and in human cell lines in vitro.  Increased lipid accumulation is observed in human in vitro models at nanomolar levels, but potentially close to the cytotoxicity threshold.  TBT is a model obesogenic chemical, inducing (transgenerational) increase in body weight/visceral lipid accumulation. This provides mechanistic support for it inducing and/or promoting hepatic lipid accumulation and steatosis. |  |
|  |  |  |  | Human, in vitro (differentiated HepaRG cells, 24 h, 3 d, 7d; 100 pM - 100 µM (cytotoxicity/ 100 pM – 1 µM (metabolomics)) | Induction (10-100 nM, NOT at 1000 nM); questionable neutral lipid accumulation at 100 nM |  | (Franco et al., 2020) | TBT induces steatosis in differentiated HepaRG cells (increased total lipids at 10 and 100 nM TBT; decreased at 1000 nM TBT). TBT was cytotoxic (24 h LC50 = 3.30 +- 0.1 µM; 7 d LC50 = 0.24 +- 0.13 µM). Increased accumulation of neutral lipids at 100 nM, but dose-response kinetic missing.  Decreased dosing range for metabolomics due to cytotoxicity at higher concentrations. | Weak/moderate candidate; maybe difficult due to cytotoxicity – but also GOLIATH chemical |
|  |  |  |  | Human, in vitro (differentiated HepaRG, 3 h – 14 d; HepG2 cells, 72 h) | Lipid accumulation in HepaRG (50 nM) and HepG2 (10 nM) cells | PPARγ, (RXRα – not involved in lipid accumulation)  Increased expression of SREBF1 and FASN genes (involved in de novo lipogenesis) | (Stossi et al., 2019) | TBT promotes RXRα protein turnover (via proteasome) and lipid accumulation, but these events are not correlated. Glycolysis rather than RXRα-mediated processes could possibly play a role in lipid accumulation.  Treatment with 5-50 nM TBT for 14 d (HCA), or 5 pM – 50 nM for 3 days (four-point concentration response) |  |
|  |  |  |  | Mouse, in vivo (n=10 female, 10 male, pre-/perinatal exposure, 0.5 mg/kg bw/d to dams/mice; <developmental NOAEL) | Increased adiposity and steatosis at 14 and 20 weeks of age in offspring | Down-regulation of growth hormone receptor (GHR) and STAT5 signalling | (Katz et al., 2020) | Developmental exposure to TBT increased adiposity and steatosis at 14 and 20 weeks of age in offspring. In males, liver adenocarcinomas were increased at 45 weeks, in females, adiposity was more severely increased than in males, but not fatty liver or tumor development, indicating **sex differences**.  Exposure of 5 week old females to TBT chloride via drinking water throughout breeding (at 7 weeks old/after 2 weeks exposure), gestation, and lactation. Males were introduced/exposed to TBT at 7 weeks old. After weaning, pups were given non-treated water. Litter size: 12 (vehicle), 15 (TBT); average: 2 pups/dam. 5 pups from each litter were analyzed for histology at 14, 20 or 45 weeks. The experiment was repeated with the same mating/exposure scenario with litter from the vehicle-control group. | Small sample size, but very thorough study |
|  |  |  |  | Mouse, in vivo (pregnant C57BL/6J mice, n=8-12; 0.05 or 0.5 mg TBT/kg bw; daily injection of TBT on embryonic day 12-20) | yes |  | (Grün et al., 2006, Chamorro-García and Blumberg, 2014) | Alterations in **adipose depots** (in males persistent until 10 weeks of age) accompanied by **hepatic steatosis**, but no increase in general bodyweight. | High confidence |
|  |  |  |  | Mouse, in vivo (female C57BL/6J mice (6 per treatment group); exposure via drinking water: 5.42 - 542 nM TBT from 7 d before mating throughout pregnancy) | Yes, lipid accumulation in livers in generations F1, F2, and, to a minor extent, in F3. |  | (Chamorro-García et al., 2013) | F0 (dams) were exposed via drinking water, F1 animals were exposed in utero, F2 animals were exposed as germ cells in F1 mice (in utero), F3 animals were unexposed. Sibling inbreeding was avoided; 6 couples per exposure group were propagated to the next generation; 4-5 litters per exposure group were included in the analysis.  Lipid accumulation in liver observed at all TBT doses in F1, at medium/high dose in F2 and, to a minor extent, in F3. Females seem more prone to lipid accumulation in the liver, especially in F2. | High confidence |
|  |  |  |  | Mouse, in vivo (pregnant C57BL/6J mice, n=8-12; 5-500 nM dibutyltin or 50 nM TBT) | Not concluded |  | (Chamorro-García et al., 2018) | Follow-up study of (Chamorro-García and Blumberg, 2014) extending exposure from 7 d pre-mating until delivery: dibutyltin led to **increased fat storage, decreased glucose tolerance, and increased circulating leptin levels in male, but not female, mice**.  To avoid confounding litter effect, litter sizes for inclusion were strictly controlled: litters with <6 or >8 litters were rejected, as were litters of <2 animals of the same sex. |  |
|  |  |  | Organotin catalyst (dibutyltin laurate) | Human, in vitro (L02 cells, 24 h, 0.05-100 µM dibutyltin laurate.) | (not concluded) | Exposure to dibutyltin dilaurate: SREBP1C (decreased), mTOR pathway (diminished), PPARα (gene expression increased) | (Qiao et al., 2018) | Dibutyltin dilaurate causes increased biomarkers of lipolysis and decreased lipogenesis in human liver L02 cells, possibly via suppressed mTOR pathway. | Problematic cell line: Contaminated. Shown to be a HeLa derivative (Ye et al., 2015). Originally thought to originate from a normal fetal liver. Mechanistic study with non-TBT organotin chemical. Moderate confidence in this study. |
| **Bisphenol A** | 80-05-7 | 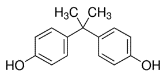 | Corrosion inhibitor in fast-drying epoxy resins, thermal paper (receipts) | **SUMMARY** | **Tentative positive (in vivo)**, uncertain in vitro |  |  | **In the wider literature, epidemiological associations but not causative biomarker-related evidence for BPA inducing steatosis/metabolic disruption. Mouse in vivo data support steatogenic effect of BPA on a weight of evidence basis.**  BPA (as a well-known oestrogen) is likely to also have obesity-related effect via ERα, as other oestrogens do.  Effects on lipid accumulation reported in rodent studies and associations in human epidemiological studies might be secondary, due to dysregulated glucose homeostasis and/or insulin signalling and sensitivity in different tissues, and nuclear receptor signalling. Such secondary hepatic steatosis could be challenging to capture in single organ/tissue in vitro systems, such as differentiated HepaRG cells. |  |
|  |  |  |  | Human, in vitro (differentiated HepaRG cells, 24 h, 3 d, 7d; 100 pM - 100 µM (cytotoxicity/ 100 pM – 1 µM (metabolomics)) | Induction (0.1-10 nM, NOT at 100-1000 nM) |  | (Franco et al., 2020) | BPA induces steatosis in differentiated HepaRG cells (increased total lipids between 0.1-10 nM; no difference to control at 100-1000 nM BPA). Strong accumulation of neutral lipids (LOEC = 10 nM)  LC50 24 h: 3.30 ± 0.10 µM, LC50 7d: 0.27 ± 0.13 µM | Moderate-low confidence |
|  |  |  |  | Human, in vitro (HepG2 cells, 24-72 h, 10^-4^-10^-12^ M) |  | Oxidative stress, inflammatory cytokine release, mitochondrial dysfunction | (Huc et al., 2012) | Low levels of BPA (10^-6^-10^-12^ M) induce mitochondrial dysfunction (ROS production, membrane hyperpolarization, lipid accumulation, lipid peroxidation, pro-inflammatory cytokine release); effects on lipid metabolism in vitro were not studied. |  |
|  |  |  |  | Human, in vitro (differentiated HepaRG cells, 72 h, 1.5×10^-9^ - 1×10^-4^ M) | Not concluded | Oxidative stress, chromatin condensation | (Landesmann et al., 2012) | at approx. 10 µM |  |
|  |  |  |  | Review for regulatory purposes | uncertain, but rather no steatosis. |  | (EFSA, 2015) | Effects of BPA on (increased) **hepatic lipid accumulation and/or lipogenesis are rather linked to low doses**; a non-monotonic dose-response was proposed/suspected but is currently not supported by EFSA.  A key study reporting low-dose (5-500 µg/kg bw/d; 28-day oral treatment of mice) effects reports accumulation of cholesterol esters and triglycerides in the liver, alongside induction of liver enzymes and increased plasma levels of glucose or cholesterol (Marmugi et al., 2012). However, four other studies cited did not observe increased insulin levels, nor triglyceride accumulation or adverse effects on the in the corresponding dose range.  In summary, in vivo studies reporting metabolic effects mostly focused on glucose homeostasis/diabetes, or adiposity endpoints, not on lipid accumulation in the liver. Nevertheless, data and endpoints are (partially) conflicting, and derivation of a clear conclusion, particularly on the induction of primary hepatic steatosis by BPA is not supported by the literature presented. | Very high confidence and relevance |
|  |  |  |  | Review for regulatory purposes; response to draft (EFSA, 2015) | BPA can have effects on metabolism, but steatosis or effects on the liver not specified |  | (ANSES, 2014) | The report highlights controversies in the conclusions made by EFSA. In contrast to EFSA, who focused on exposure of the general population/via food, ANSES extends the considered population to cover also occupational exposure, e.g. of cashiers handling thermal paper receipts.  Specifically, ANSES considers the available experimental evidence “sufficient to consider that BPA can have effects on metabolism”, and the most reasonable metabolism endpoint/process affected is glucose and insulin regulation and/or pancreatic morphology and function. | Very high confidence and relevance |
|  |  |  |  | Review | Unlikely to induce primary steatosis (but not specifically concluded upon) |  | (Le Magueresse-Battistoni et al., 2018) | Effects on metabolism (in humans) are most likely to affect insulin secretion, effects on pancreas function/type II diabetes, and glucose homeostasis. Effects towards obesity linked to the well-documented estrogenicity of BPA are noted. | Very high confidence, high relevance |
|  |  |  |  | Human, epidemiology/ environmental exposure (adult males; n=76) | Not concluded/assessed; study used blood plasma | IL-6 | (Savastano et al., 2015) | Visceral adiposity (24 out of 76 subjects) correlated with higher plasma levels of BPA, triglycerides (elevated), and pro-inflammatory cytokines (esp. IL-6). |  |
|  |  |  |  | Human, epidemiology (US, NHANES; n=11,236) |  |  | (Kim et al., 2019) | High levels of BPA were associated with NAFLD using both, the hepatic steatosis index (HIS) and the US fatty liver index (USFLI).  NHANES (National Health and Nutrition Examination Survey) cohort is representative of US population | High confidence and relevance  Very large sample size |
|  |  |  |  | Human, epidemiology (representative Canadian household population; n=4733) | Not concluded |  | (Do et al., 2017). | Urinary bisphenols are associated with BMI-defined obesity. |  |
|  |  |  |  | Mouse, in vivo (male 6-week-old CD1 mice & C57BL/6J mice, n=6/group; 5 groups, 28 d exposure via diet to 0.05-50 ppm BPA) | Altered lipogenesis and triglyceride accumulation in the liver | Lipogenesis (ACLY, ACACA, ACACB, FASN, ELOVL5, SCD1, GPAT, LPIN1, THRSP-SPOT14, PNPLA3, PNPLA5), fatty acid oxidation (to a lesser extent; PECI, CYP4A14), cholesterol biosynthesis (to a lesser extent; MVD, LSS, HMGCR, SQLE) | (Marmugi et al., 2012) | Focus on effects in CD1 mice; confirmation study in C57BL/6J mice confirmed trend in effects/observations, but with smaller magnitude.  No effect on body weight gain or liver weight, plasma glucose, od cholesterol (LDL-C, HDL-C); plasma insulin was significantly increased at 5-500 µg/kg bw/d with reverse dose-response pattern (highest increase at lowest dose), significant increase in plasma triglycerides upon exposure to 500 µg/kg bw/d.  **Low doses** influence de novo lipogenesis in male CD1 mice, thereby contributing to hepatic steatosis.  Oral exposure corresponds to 0 (controls), 5, 50 (TDI), 500, or 5,000 µg BPA/kg bw/d (NOAEL), assuming diet consumption of 10% bw/d. | High confidence |
|  |  |  |  | Mouse, in vivo (pregnant CD1 female mice, exposed daily orally via micro-pipettor from gestational day 9-18 (during period of differentiation of preadipocytes) to 5-50,000 µg/kg bw/d BPA; effects observed in male offspring only) | Low dose (50 µg/kg bw/day), but not higher doses (≥500 µg/kg bw/day) increased liver weight |  | (Angle et al., 2013) | Litter size per BPA treatment group [µg/kg bw/d BPA]: 14 (negative control), 9 (BPA-5), 12 (BPA-50), 12 (BPA-500), 11 (BPA-5,000), 14 (BPA-50,000), 9 (DES-0.1/positive control). Study was concluded when males were 5 months old.  **Prenatal exposure to BPA levels at or below the current [2013] predicted NOAEL of 5000 µg/kg bw/d** resulted in significant increase in postnatal body weight gain, adipocyte number and volume and the overall amount of abdominal fat, altered food intake, serum insulin, adiponectin and leptin levels, and impaired glucose tolerance and insulin sensitivity in **male offspring**. These effects were not statistically significant at the highest dose, supporting **non-monotonic dose-response** for BPA effects on traits of metabolic syndrome. | High confidence |
| **Triphenyl phosphate (TPP)** | 115-86-6 | 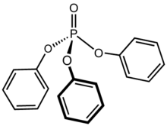 | Plasticizer, organophosphate flame retardant | **SUMMARY** | **Positive** | PXR (agonism), GR (antagonism), PPARγ (agonism), PPARα |  | In vivo data indicate a potential for changes in liver weight (increase), lipid metabolism, and altered blood lipid profile in human, rodent, and (zebra)fish, but the **weight of evidence for conclusion on TPP being causative of primary hepatic steatosis is moderate**.  Key references to conclude on TPP inducing steatosis are human in vivo increase of liver weight (ECHA and ANSES, 2019), mouse in vivo steatosis (triglyceride accumulation in hepatocytes) (Wang et al., 2019), and zebrafish in vivo lipid accumulation in liver (Du et al., 2016); details are listed below.  Mechanistic support for TPP contributing to lipid accumulation can be provided by an in vitro study reporting increased lipid accumulation in primary human subcutaneous preadipocytes. |  |
|  |  |  |  | Human, in vitro (primary subcutaneous preadipocytes from female donors, 0-20 µM TPP; exposure from day 0 or 2 until day 14) | Not concluded/ not applicable (but lipid accumulation observed in **preadipocytes**) | Gene expression increased: **PPARG**, FABP4 (also protein, significant at 20 µM), LPL, PLIN, CEBPα, **SREBF1**  Decreased: INSIG1, INSIG2  Enriched pathways: adipogenesis, LXR/RXR activation, cholesterol biosynthesis I, LPS/IL-1-mediated inhibition of RXR function, PKA signalling, AMPK signalling, Type II diabetes mellitus signalling | (Tung et al., 2017) | Increased adipogenesis. Significant lipid accumulation at 20 µM.  For gene expression, interestingly, increased markers were observed especially at days 6 and 9 of treatment, and changes in gene expression were relatively reduced/less pronounced at day 12/end of differentiation.  Potential modes of action: via LXR, TR, PKA, and/or nuclear receptor subfamily 1 group H members. |  |
|  |  |  |  | Human, in vivo (epidemiology; n=259 (118 male, 141 female); median age 29 years) | Not assessed, but increase in plasma TG and TC associated with TPP metabolite 4-OH-TPP |  | (Zhao et al., 2019) | Association of urinary metabolites of three aryl organophosphate flame retardant (TPP, tricresyl phosphate, 2-ethylhexyl diphenyl phosphate) metabolites and plasma levels of triglycerides and total cholesterol.  Log-transformed triglyceride and cholesterol levels showed a linear interquartile increase with measured 4-OH-TPP and 5-OH-EHDPP (p trend<0.001 in creatinine-corrected concentrations; for TPP and triglycerides: 28.4% (95% CI: 26.5-30.0%) increase between lowest and highest quartile) | No exclusion (but moderate incidence: 29/259 moderate or heavy drinker) based on alcohol consumption, smoking, or BMI (median: 21.2, range: 15.7-36.9); no information on volunteer health status |
|  |  |  |  | Review (human health risk assessment) for regulatory purposes | Increased liver weight (steatosis not concluded)  TPP-treatment related obesity/increase in body weight was correlated with leptin-levels | PXR agonist, GR antagonist  No TRβ binding in reporter cell transactivation assay, no binding to transthyretin, but potentially leading to increased Thyroxine (T4) levels in human (especially in women) | (ECHA and ANSES, 2019) | Summary of relevant human data:  Human data indicate possible effects on thyroid hormone signalling.  Studies in rodents and rabbits indicate no acute toxicity (oral LD50 > 5 g/kg bw), no genotoxicity, no immunotoxicity, no acute neurotoxicity in hens, except for decreased plasma acetylcholine esterase activity.  In rats, liver weight was increased after repeated exposure (35 d via diet) to ~ 350 mg TPP/kg bw/d or (90 d via diet) 7500 ppm (30% in males, 21% in females). Additionally, 90 d exposure induced centrilobular hepatocellular hypertrophy at ≥1500 ppm TPP.  An (unpublished) GLP study (OECD TG414) concludes on a NOAEL for maternal and developmental toxicity at 80 mg TPP/kg bw/d, based on treatment-related loss of one litter in the high dose (200 mg/kg bw/d), and aberrant development of the lungs (litter incidence rate 1.6-2.4%).  A study into TPP-altered metabolism revealed, that perinatal exposure (GD 8.5 – PND 21) induced metabolic disturbances leading to increased weight gain and enhanced adiposity, correlating with serum leptin levels, thus probably attributable to increased appetite and obesity-associated leptin-resistance. This was more pronounced in females than in males.  Androgenicity: no specific androgenic potential Estrogenicity: some estrogenicity in the micromolar range (but close to cytotoxicity limit at 3.45 µM)  In fish, there is substantial evidence for endocrine disruption, especially due to estrogenicity and potentially thyroid disruption/ disruption of the hypothalamic-pituitary(-thyroid) axes. | Very high confidence, high relevance |
|  |  |  |  | Mouse, in vivo (primigravida pregnant ICR mice, n=6 per group. Exposure via oral gavage on GD 6-PND 21; 10-1000 µg/kg bw. Offspring of high-dose were divided into low- vs. high-fat diet groups) | Yes. Triglyceride accumulation in liver upon perinatal exposure to 1000 µg/kg bw | Gene expression: **PPARG**↑, MOGAT1↑, FASN↑ (upregulated on low-fat diet, downregulated on high-fat diet), no induction of PPARA | (Wang et al., 2019) | All doses, but especially 100-1000 µg/kg bw increased total body weight.  Body weight, liver weight, and liver triglyceride content were significantly increased upon perinatal exposure to 1000 µg TPP/kg bw compared to vehicle control; low-fat diet upon perinatal TPP exposure resulted in a comparable increase as high-fat diet after vehicle treatment; perinatal TPP exposure followed by high-fat diet exacerbated the increase.  NAFLD score was significantly increased in all animals on a high-fat diet; TPP-exposed animals on a low fat-diet showed increased, but not statistically significant NAFLD scores. In Oil Red-stained liver histology specimen, some increase in Oil Red staining is apparent with TPP-exposed low-fat diet samples but is most prominent in TPP-exposed high-fat diet specimen. | High confidence and relevance  Steatosis was only observed at high dose (1000 µg/kg bw) |
|  |  |  |  | Mouse, in vivo (female FVB mice, 5-week-old. Acute exposure (n≥4 per treatment group) via vein-tail injection (1 h; TPP: 0.1-100 µg; DPP: 0.1-1 µg), oral gavage (1 h; TPP: 0.1-100 µg; DPP: 0.1-1 µg), or drinking water (overnight; TPP or DPP: 0.1-10 mg/L). Chronic exposure via drinking water (n= 10 per group; 12 weeks; DPP: 0.1-10 mg/)) | Dose-dependent **decreased** lipid content in liver, and decreased bodyweight | Gene expression: **PPARα**-regulated: **HMGCS2↓**, CPT1A↓, CD36, SCD1, FADS2 | (Selmi-Ruby et al., 2020) | Diphenyl phosphate (DPP) was assessed as a biomarker for aryl phosphate (including TPP) exposure. It was concluded to not be appropriate, at least in biological fluids.  While TPP could only be detected (LOD=0.7 ng/mL) in blood of 2/20 animals upon exposure to ≥10 µg (tail vein or gavage). DPP was readily quantifiable in all DPP-exposure scenarios; upon TPP exposure DPP levels in blood were 100x lower than after DPP treatment, and <LOQ (LOQ: 0.5 ng/mL) at the two low dose levels.  Chronic exposure for 12 weeks to DPP led to significantly increased levels in blood (≥1 mg/L) and measurable levels in the liver, mammary glands, and visceral fat at all tested concentrations.  Even at low-dose exposure to 100 µg DPP/ml drinking water, reduction of the fatty acid catabolic processes, centred on acylcarnitine and mitochondrial β-oxidation, was observed. (but no significant effect on endogenous fatty acid synthesis)  DPP decreased expression of genes involved in lipid catabolic processes and regulated by PPARα. Immunohistochemistry analysis showed a specific down-regulation of HMGCS2, a kernel target gene of PPARα. Overall, DPP absorption disrupted body weight-gain processes. | Not all biomarkers related to hepatic steatosis were addressed, as the focus of the study was analytical chemistry. |
|  |  |  |  | Rodent, in vivo (primigravida pregnant ICR mice, s.c. neonatal exposure PND 1-10, 2 and 200 mg TPP or DPP per day). | Not concluded. (but increased serum lipids and lipid-sub-species, especially in male mice) |  | (Wang et al., 2018) | sex-specific metabolic disturbance of TPP. Specifically, low dose of TPP altered the metabolic profiles of male mice (and increased body weight gain between 6-12 weeks postnatal, but no changes in abdominal adipose weight (12 weeks) or glucose tolerance (10 weeks). Low dose up-regulated lipid-related metabolites, while a high dose down-regulated the pyruvate metabolism and TCA cycles. (in adult males: 2 mg TPP upregulation: lipid, taurine, PC, UFA, and PUFA. 200 mg TPP upregulation: lipid, taurine, PUFA, and UFA levels; downregulated: choline, succinate, lactate, and pyruvate.) (In adult females: no statistically significant difference in 2 mg TPP group. 200 mg TPP: downregulation: lipid, UFA, PUFA, choline and acetate.)  At 12 weeks, no difference in estradiol levels. No increase in uterine weight in uterotrophic bioassay (1-600 mg/kg bw/day, 3 days in 13-d-old female ICR mice or SD rats) |  |
|  |  |  |  | Rodent, in vivo (male ICR mice 4 week old, 0, 100 and 300 mg/kg bw/d, oral via diet for 5 weeks. N=7 per dose group) | No steatosis relevant parameters assessed, although disruption of cholesterol synthesis pathways may be (indirectly) relevant mechanisms.  Not relevant for adipogenesis steatosis relevant endpoints, none measured or reported on. Decrease of liver weight noted at 300mg/kg. But no analysis of lipid content/adipocytes in the liver. | Hepatic malondialdehyde (MDA) contents increased significantly in both TPP treated groups, while the contents of glutathione (GSH) decreased significantly in 300 mg/kg TPP | (Chen et al., 2015) | Effects of TPP and tris(2-chloroethyl) phosphate (TCEP) on the induction of oxidative stress and ‘endocrine disruption were evaluated in five week old male mice. After receiving 100, 300 mg/kg/ body weight oral exposure to TPP and TCEP for 35 day (short term chronic exposure) , the body and testis weights decreased in the 300 mg/kg TPP and TCEP treated groups. Hepatic malondialdehyde (MDA) contents increased significantly in both TPP treated groups, while the contents of glutathione (GSH) decreased significantly in 300 mg/kg TPP (and both TCEP treated groups).  Not surprisingly, the hepatic activities of antioxidant enzymes including glutathione peroxidase (GPX), catalase (CAT) and glutathione S-transferase (GST) as well as their related gene expression were affected by TPP (or TECP).  300 mg/kg of TPP (or TECP) treatment resulted in histopathological damage and the decrease of testicular testosterone levels. The expression of main genes related to testosterone synthesis including steroidogenic acute regulatory protein (**StAR**), low-density lipoprotein receptor (LDL-R), cytochrome P450 cholesterol side-chain cleavage enzyme (P450scc) and cytochrome P450 17-hydroxysteroid dehydrogenase (P450-17) in the testes also decreased after the exposure to 300 mg/kg TPP (or TCEP) for 35 days.  **Authors Conclusion**: Combined interpretation of the physiology, histopathology and gene expression results: 4 week chronic exposure at 300 mg/kg of TPP and TCEP can induce oxidative stress and disrupt the steroidogenic pathway in mice, but potential confounding by the chemical delivery not addressed adequately, nor detail on bedding and phytoestrogen free diet.  **Strong support** for adverse impact upon StAR and steroidogenic pathway.  **Weaknesses: potential confounder**: anhydrous ethanol stated to have volatilized entirely, but how this was assessed is not given. There was no vehicle control group to check for this potential confounding. It would have been useful to look at sperm morphology (particularly easy in mouse) and conduct microRNA analyses. | Useful paper for NOAEL of 100 mg/kg and LOAEL of 300 mg/kg based on hepatic GSH depletion, but potential confounding by the chemical delivery not addressed adequately. |
|  |  |  |  | Zebrafish, in vivo (AB-strain, 5-month-old adults; exposure via water, n=30 fish per tank (15 male/15 female) 3 parallel tanks per treatments group, 50 and 300 µg/L for 7 d) | Yes, most likely. Changes indicative of lipid accumulation observed in liver histopathology at both concentrations (50 and 300 µg/L). |  | (Du et al., 2016) | 96h-LC_50_: 1.026 mg/L (following OECD TG203)  Metabolomics: significant changes in Carbohydrate metabolism (glucose, UDP-glucose, glycolate, lactate, succinate, fumarate), lipid metabolism (choline, acetylcarnitine, esterified cholesterol, arachidonic acid, timnodonic acid, linoleic acid, and αH_2_ fatty acids), amino acid metabolism (glutamate, glutamine, leucine), and osmolyte metabolism (TMAO, dimethylamine).  Transcriptomics, affected pathways: primarily carbohydrate and lipid metabolism, and DNA repair. Further, glycosphingolipid biosynthesis, PPAR signalling pathway, and fatty acid elongation  Blood markers elevated at both concentrations tested: glucose, pyruvate (less pronounced at high-dose), triacylglycerides, triacylcarnitines. HDL-C only elevated at high-dose, LDL-C not altered.  Liver histology: both doses induced obvious vacuolization and enlarged sinusoidal vessels, indicating lipid accumulation. Additionally, pyknotic nuclei indicate liver apoptosis – this was more pronounced in the high-dose group. |  |
| **Triclosan** | 3380-34-5 | 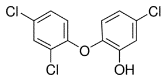 | antibacterial and antifungal agent | **SUMMARY** | **Uncertain/ negative** | Oxidative stress |  | **Moderate weight of evidence does not indicate triclosan to induce primary hepatic steatosis**. Though changes to hepatic lipid metabolism might occur due to some evidence associating triclosan with increased body weight and an obesogenic potential. Conflicting evidence is reported from an amphibian study, where early life stage exposure to triclosan induced metabolic syndrome in F0 adults (including hepatic steatosis) (Regnault et al., 2018), but molecular mechanisms in *Xenopus* might be of limited relevance towards human health predictivity due to, e.g. substantial structural differences in the ligand binding pocket of relevant nuclear receptors (PPARγ).  However, the reviewed literature indicates **sex differences, with males being more susceptible** to triclosan effects. |  |
|  |  |  |  | Human, in vitro (primary human hepatocyte spheroids, 100 µM, 28 d exposure) | No induction. Steatosis (and fibrosis) genes were downregulated |  | (Vilas-Boas et al., 2021) | Concentration-dependent decrease in ATP content (substantial at ≥100 µM) in primary human hepatocyte spheroids from day 7 (repeated exposure, up to 28 d) |  |
|  |  |  |  | Human, in vitro (differentiated HepaRG cells, 72 h, 7.6×10^-9^ - 5×10^-4^ M) | Not concluded |  | (Landesmann et al., 2012) | Chromatin condensation, decreased cell count, **and ROS production** in differentiated HepaRG (72 h exposure; at approx. 60-100 µM) |  |
|  |  |  |  | Human, in vitro (L02 and HepG2 cells, 48 h, 0.01-20 µM.) | **Decreased** triglycerides in HepG2 cells; (**elevated** triglycerides and lipid accumulation in L02 cells) | Oxidative stress | (Zhang et al., 2019a) | **Lipid accumulation and ROS production in L02** (non-cancerous) **hepatocytes**, but fast detoxification via phase II metabolism and **elevated antioxidant defense in hepatoblastoma HepG2 cells**. Intracellular lipid dysregulation was observed in both cell lines (e.g. triglycerides were up regulated in L02 cells, but decreased in HepG2). | Problematic cell line L02: Contaminated. Shown to be a HeLa derivative (Ye et al., 2015). Originally thought to originate from a normal fetal liver. |
|  |  |  |  | Human, in vivo. Epidemiology (mother-child pairs, n=850) |  |  | (Wu et al., 2018) | Prenatal exposure (measured by urinary triclosan) had no effect on birth weight, but especially early exposure (1^st^ and 2^nd^ trimester) was associated with increased weight in 2-year old infants. The effect was more prominent in females and suggests **potential sex-differences**. |  |
|  |  |  |  | Human, in vivo. Randomized controlled trial (n=383; annual assessment for 5 years) | no |  | (Cullinan et al., 2015) | Triclosan toothpaste decreased cholesterol (total cholesterol, high and low density lipoprotein). Some inflammatory biomarkers were altered, but clinical significance of this finding remains unclear. **No influence of triclosan toothpaste use on liver or kidney function** was concluded. |  |
|  |  |  |  | Human, in vivo. Experimental exposure (0.3% triclosan in toothpaste; 2-cm strip of toothpaste for 3 min, 2x/d for 14 days; n=12) | Not concluded |  | (Allmyr et al., 2009) | Blood plasma triclosan concentrations increased from 0.009–0.81 ng/g to 26–296 ng/g (ranges) upon exposure; no changes in 4β-hydroxycholesterol (indicative of CYP3A4 activation via PXR) or thyroid hormones were detected. Liver parameters were not monitored. |  |
|  |  |  |  | Review | No/mild steatogenic potential. |  | **EFSA opinion** (SCCP, 2009) | **Decreased** **triglycerides** in high dose (600 mg/kg/d) 90-day subchronic oral toxicity study in rats. Further: increased liver weight (more pronounced in males); mild hepatic centrilobular cytomegaly and **fatty metamorphosis** in mid-and high-dose **males**.  104-week long-term toxicity/carcinogenicity study in rats: transient changes in liver enzymes and triglycerides; slight relative liver weight decrease at high dose; hepatocyte hypertrophy and hepatocytic inclusions. (similar findings in 95-week hamster study; also more pronounced in **males**). Hepatic effects in mice were more extensive, including **increased cholesterol** levels (both sexes) and hypertrophy of the liver (**males** only) and occurring at low doses (10 mg/kg bw/d). | Strong confidence: opinion and review for regulatory purposes |
|  |  |  |  | Mouse, in vivo (male C57BL/6 mice; exposed from 3 weeks old for 8 months through chow diet; 0.08% triclosan) | Not concluded | Oxidative stress (ROS production)  CAR activation, no significant effect on PPARα activation  Inflammatory cytokines TNFα, IL-6, and fibrogenic TGFβ | Yueh et al. 2014) | Triclosan acts as a liver tumor promotor (upon tumorigenesis initiation by diethylnitrosamine) and enhances fibrogenesis. Increased cytokines (including inflammatory TNFα, IL-6, and fibrogenic TGFβ) in the liver. |  |
|  |  |  |  | Amphibian, in vivo (*Xenopus tropicalis*, transgenerational study. F0 female tadpoles (n=150) exposed to 50 ng/L TCS (7 d old to mature adult stage/12 months)) | Liver steatosis and hypertriglyceridaemia induced | Oxidative stress, ER stress  enriched KEGG pathways (transcriptomics): “FoxO signaling pathway” (3.7-fold); “Protein processing in endoplasmic reticulum” (3.4-fold) “Metabolism of xenobiotics by cytochrome P450” (7.4-fold) and “Chemical carcinogenesis” (7.1-fold) | (Regnault et al., 2018) | Exposure during early life stages resulted in metabolic syndrome (decreased glucose tolerance, liver steatosis, liver mitochondrial dysfunction, pre-diabetes (liver transcriptomic signature and pancreatic insulin hypersecretion)) and delayed metamorphosis in adults (F0). F1 progeny displayed decreased size and weight at metamorphosis. (Also mating of exposed animals was less efficient: 3 out of 5 females rejected the male despite sexual maturity, indicating TCS-related changes to sexual behaviour in females))  Steatosis assessed histopathologically; modifications observed upon TCS exposure correspond to NAFLD, but not (yet) NASH. |  |
|  |  |  | [no triclosan intervention] | Human, in vitro (liver tissue from 27 NAFLD patients and 20 control individuals);  mouse in vivo (different genotypes; male; C57BL/6 background. n=4-8 per treatment group; treatment by dietary intervention for 4-16 weeks) |  | Macrophage M1 p38α (mediated via: CXCL2, IL-1β, CXCL10, IL-6, TNF-α) | (Zhang et al., 2019b) | hepatocyte-specific p38a knockout (p38α^ΔHep^), macrophage-specific p38α knockout (p38α^ΔMΦ^) and wild-type (p38α^fl/fl^) mice fed with high-fat diet (HFD), high-fat/high-cholesterol diet (HFHC), or methionine-and choline-deficient diet (MCD). p38 inhibitors were administered to HFHC-fed wild-type mice for disease treatment.  Compared to p38α^fl/fl^ littermates, p38α^ΔHep^ mice developed significant nutritional steatohepatitis induced by HFD, HFHC or MCD. Meanwhile, p38α^ΔMΦ^ mice exhibited less severe steatohepatitis and insulin resistance than p38α^fl/fl^ mice in response to a HFHC or MCD.  Macrophage M1 deficient p38α^ΔMΦ^ mice partly compensated the pro-inflammatory mediation of steatohepatitis through polarization of M2 macrophages (CD45^+^F4/80^+^CD11b^+^CD206^+^) and enhanced liver arginase activity.  Restoration of TNF-α, CXCL10, or IL-6 induced lipid accumulation and inflammatory responses in p38α^fl/fl^ hepatocytes co-cultured with p38α^ΔMΦ^ macrophages. | Strong study; interspecies (mouse/ human) differences addressed.  Not relevant for triclosan-induced steatosis, but generally relevant for mechanism of steatosis |
| **p,p’-** **Dichlorodiphenyldichloroethylene (DDE)** | 72-55-9 | 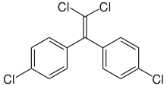 | Metabolite of organochlorine insecticide | **SUMMARY** | **Positive** |  |  | **Moderate weight of evidence** to support DDE causing primary hepatic steatosis.  Epidemiological evidence reports an association of DDT, the parent chemical of DDE, with increased incidence of fatty liver (La Merrill et al., 2019), and hepatotoxicity and fatty changes in the liver observed in non-human primates exposed to DDT (Takayama et al., 1999). This is supported by a systematic review and meta-analysis associating DDE exposure with increased BMI/adiposity (mechanistic support for lipid accumulation in different tissue) (Cano-Sancho et al., 2017).  DDT and its metabolite DDE are listed under the Stockholm Convention on Persistent Organic Pollutants, Annex B: Restriction of use (UNEP, 2019). DDT and its metabolites are restricted for use for regulatory purposes in some OECD member countries, e.g. Japan. |  |
|  |  |  |  | Human, in vivo (epidemiology/ biomonitoring; NHANES cohort. Random subset of approx. 2500 participants included in analysis in each survey period) | Not concluded |  | (CDC Department of Health and Human Services, 2009) | Average US household representative survey.  Geometric mean (and 95% confidence interval) of *p,p’*-DDE in the respective survey years was: 260 (226-298) ng/g lipid (1999-2000), 295 (267-327) ng/g lipid (2001-2002), and 238 (195-292) ng/g lipid (2003-2004). (whole-weight, instead of lipid-adjusted, serum concentrations, as well as information on other isoforms of DDE or the parent DDT, or 50^th^, 75^th^, 90^th^, and 95^th^ percentiles are listed in the original publication) | Very high confidence.  Newer versions of the report/ measurements of POPs do not discriminate the different POPs due to low levels measured in the population |
|  |  |  |  | Human, in vivo (epidemiology, CHAMACOS cohort, n=468 women) | Not concluded (focus on adiposity measures; no association of serum DDE with adiposity measures) |  | (Warner et al., 2018) | Longitudinal birth cohort of low-income Mexican American mother-child dyads living in an agricultural community in California. Study was initiated in 1999 (prenatal); anthropometric visits included in this evaluation coincide with the child’s age of 9-12 years (three time points).  Positive associations were observed between serum DDT and BMI, waist circumference, and body fat percent. (no consistent statistically association for DDE)  Geometric mean concentration (if detected): *p,p’-*DDT: 4.7 ng/g lipid (detected in 57.5% of samples); *p,p’-*DDE: 291.2 ng/g lipid (detected in 100% of samples)  Plots of univariate exposure-response relationships for chemical exposure and change in BMI suggest inverse associations for DDT and DDE: while **positive association was statistically significant for DDT, DDE shows a clear negative trend** (though not statistically significant). | Very high confidence |
|  |  |  |  | Systematic review/ meta analysis (Articles included in data-extraction: n=39) | Not concluded (focus on adiposity; DDE and DDT were classified as presumed obesogens for humans) |  | (Cano-Sancho et al., 2017) | **Positive associations between exposure to *p,p*’-DDE and BMI** z-score (β=0.13 BMI z-score (95% CI: 0.01, 0.25) per log increase of *p,p*’-DDE)  Systematic review that applied the National Toxicology Program framework based on the Grading of Recommendations Assessment, Development and Evaluation “GRADE” approach, including a risk of bias assessment, identified 19 in vivo studies and 7 in vitro studies that supported the biological plausibility of the obesogenic effects of *p,p*’-DDE and *p,p*’-DDT. | Very high confidence |
|  |  |  |  | Rat, in vivo (male Wistar rats, 4-6 weeks old, n=8 per group; 4 week exposure, 10 mg/kg bw via diet) | Steatosis induced | Oxidative stress, namely via **UCP2** and **SOD2** (high fat diet induced rather GPx1, malonaldehyde was elevated by both, DDE and diet)  CYP2B induction | (Migliaccio et al., 2019) | No change in liver weight was observed, but diet and DDE exposure increased the hepatic lipid content.  Carnitin-palmitoyl transferase (CPT) system, as a measure of mitochondrial fatty acid oxidation, and β-oxidation were significantly increased upon DDE exposure, notably above levels of diet-induced levels. Histological staining of liver slices revealed no significant increase in lipid droplets upon exposure to DDE alone, however a vacuolated appearance around blood vessels, and an increase in eosinophilic cells was noted. Macrophage infiltration into liver tissue was confirmed by immunohistological staining (CD68^+^). |  |
|  |  |  |  | Rat, in vivo (male Wistar rats, 8 weeks old, n=6/group; 12 week exposure, 100 µg/kg bw/d via drinking water) | Hepatic fat accumulation | Synthesis of polyunsaturated fatty acids (i.e. arachidonic acid, docosahexaenoic acid) | (Rodríguez-Alcalá et al., 2015) | The applied daily dose (100 µg/kg bw/d) is 2.5 times lower than the LOAEL at the time of the study. *p,p’*-DDE was administered to mice fed a standard or high-fat diet.  DDE exposure increased liver fat content; adipose tissue fatty acid composition was mostly unaffected. High-fat diet exacerbated hepatic lipid accumulation.  Fatty acid changes in the liver were mainly in palmitic, stearic, oleic, trans fatty acids, and linoleic acids (all increased). Arachidonic acid and docosahexaenoic acid, which were not supplied in the diet were altered as well. Therefore, changes in the n6 and/or n3 metabolic pathways, which are related to the synthesis of anti-inflammatory mediators, are hypothesised. | Moderate confidence |
|  |  |  | DDT | Human, in vivo (epidemiology pilot MASALA; n=147) | Increased incidence of fatty liver with increasing **DDT** plasma levels |  | (La Merrill et al., 2019) | Plasma samples were analysed for 30 (persistent) environmental pollutants by GC-MS/MS; study focused on South Asians (aged 45-84 years) living in America.  DDT plasma levels were associated with hallmarks of metabolic disease, including increased obesity (body mass index and/or waist circumference), adiposity, insulin insensitivity, **fatty liver**, and (pre)diabetes. Particularly *p,p’-*DDT was associated with **increased hepatic fat,** circulating insulin levels and decreased insulin sensitivity **independent of obesity**.  **DDT was associated with a 2.4 increased odds of fatty liver** in a subset of 134 study participants, who were examined for fatty liver (liver-to-spleen ration by computed tomography). It is concluded that **DDT possibly primes the liver to be susceptible to fat accumulation** through effects both dependent and independent of the positive association of DDT on adiposity. | High confidence.  Most significant findings are for DDT, the parent compound of DDE |
|  |  |  |  | Non-human primate, in vivo (cynomolgus (n=13) and rhesus monkeys (n=11); 130 months exposure to 20 mg/kg **DDT** in the diet, followed by ≈180 months observation period) | Not concluded specifically, but hepatotoxicity was reported. |  | (Takayama et al., 1999) | **Fatty changes in the liver** were observed in 52.9% of the DDT group and 29.4% of the control group; **histologic evidence of hepatotoxicity** was microscopically documented in **7 DDT monkeys** (liver cell necrosis, oval-cell proliferation, clear hepatocyte foci). Further, CNS and spinal cord abnormalities were documented in 6 DDT monkeys. Further, in DDT monkeys 2 malignant tumours (metastatic hepatocellular carcinoma, adenocarcinoma of the prostate) and 6 benign tumours were recorded (no tumours in control group; n=17).  Conclusion: evidence for CNS and hepatotoxicity, but not carcinogenesis of DDT in non-human primates. | Very high confidence and moderate relevance |
| **Cyproconazole** | 94361-06-5 | 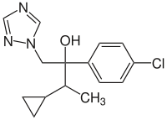 | azole fungicide, wood preservative | **SUMMARY** | **Positive (tentative)** | RARα, PXR, CYP (gene expression and protein abundance)  CYP51 (target of triazole fungicides; mode of fungicidal action) |  | **Moderate-strong mechanistic weight of evidence** to support cyproconazole causing primary hepatic steatosis **in vitro**. Levels of lipid accumulation observed upon cyproconazole exposure correspond those observed with the assay positive controls (oleate, or oleate-palmitate mixture), or cyproconazole is used as a (positive) reference chemical (Lichtenstein et al., 2020).  However, GLP rodent in vivo studies (**strong weight of evidence**) identify the liver as a target organ for (tri)azole toxic effects, including **increased relative liver weight, but no histopathological evidence for steatosis** or lipid accumulation is reported, including in 2-year chronic exposure rodent studies (EFSA, 2009). | Reasonable level of in vivo literature available, but high level of uncertainty to conclude on steatosis induction in vivo. |
|  |  |  |  | Human, in vitro (differentiated HepaRG cells, 24-72 h, 25-200 µM) | Steatosis in HepaRG cells in vitro | RARα, PXR  (further gene expression changes: CYP1A2, **CYP2B6**, **CYP2E1 (↓)**, **CYP3A4 (↑)**, CYP3A7, **CYP7A1 (↓)**, G6PC, INSIG1, **RGCC (↓)**, SULT1B1, SULT1C2, TFF3, UGT2B7  Changes in protein abundance: **ACOX1 (↓)**, ALDH1A1, ALDH2, **CES2, CYP2A6, CYP3A4 (↑)**, **FASN (↓)**, GPD1, HAAO, KHK, MAPK8(↓), MTTP, UGT2B7) | (Luckert et al., 2018) | Cyproconazole dose-dependently activated RARα and PXR (other NRs screened AhR, CAR, FXR, GR, LXRα, PPARα, PPARγ, PPARδ, RXRα, VDR), disrupted mitochondrial functions, induced triglyceride accumulation, and the formation of fatty liver cells.  Activation of nuclear receptors by cyproconazole was studied in transiently transfected HEK-293 (CAR, FXR, LXRα, PPARα, PPARγ, PPARδ, PXR, RARα, RXRα) and HepG2 cell (AHR, GRE, CAR-CYP2B6, PXR-CYP2B6, VDR-CYP2B6) reporter gene systems. Exposure to cyproconazole: 24 h, 15-60 µM. | Strong confidence |
|  |  |  |  | Human, in vitro (differentiated HepaRG cells, 72 h, 7.6×10^-9^ - 5×10^-4^ M) | Not concluded/ assessed | Oxidative stress (0.01 µM) | (Landesmann et al., 2012) | ROS induction (0.01 µM) and decreased cell count, nuclear staining intensity and nuclear are reduction (approx. 60-100 µM) in differentiated HepaRG (72 h exposure) |  |
|  |  |  |  | Human, in vitro (differentiated HepaRG cells, 24-72 h, 25-250 µM) | Strong induction *in vitro* (HepaRG; EC50=92.4 µM) |  | (Lichtenstein et al., 2020) | 200 µM cyproconazole as positive control; steatosis induction of approx. 2.5-fold (AdipoRed assay; similar to oleate-palmitate 250 µM positive control) | Very high confidence |
|  |  |  |  | Human, in vitro (HepG2 (24 h exposure) and differentiated HepaRG (72 h exposure), 200 µM) | Strong induction |  | (Lasch et al., 2020) | Cyproconazole was used as a positive control chemical for an in vitro lipid accumulation study in hepatocytes. 200 µM cyproconazole induced ~1.4-2.5-fold increase in intracellular triglyceride content in HepaRG cells (data not shown for HepG2). | High confidence and relevance |
|  |  |  |  | Review | Fatty changes *in vivo* (rodent) |  | (Lichtenstein et al., 2020) | Data reviewed based on test guideline studies according to OECD TG 417. Data source: list of endpoints on residues, active substance.  Fatty changes classified based on “Cumulative Assessment Group 2b: fatty changes” (Nielsen et al., 2012). Also, high oral absorption and high liver residue in vivo. | Very high confidence; literature data based on guideline studies |
|  |  |  |  | Review for regulatory purposes | **No steatosis**. Liver as the primary target organ for toxicity, but no lipid accumulation | CYP51 (target of triazole fungicides; mode of fungicidal action) | (EFSA, 2009) | Risk assessment for a selected group of pesticides from the triazole group (including cyproconazole and tebuconazole) to test possible methodologies to assess cumulative effects from exposure through food from these pesticides on human health; majority of studies included are rodent in vivo studies.  Studies, **including 2-year chronic exposure GLP studies,** **do not indicate lipid accumulation in the liver**. Increased relative liver weight is reported frequently, and one of the most sensitive toxicological endpoints. However, this could be explained by observed hepatocyte hypertrophy and swelling; no lipid accumulation or steatosis was reported. (Tri)azol fungicides act by inhibiting CYP51, which in mammals is involved in cholesterol biosynthesis. Other CYP enzymes are affected as well (including CYP2A and CYP3A family), and this can affect steroid hormone synthesis and impact upon the (hepatic) metabolic balance, particularly metabolite flows between oxidases, including mixed-function oxidases. | Very high confidence and relevance  Also for conclusion on triazole fungicide chemical group |
|  |  |  |  | Rat, in vivo (28-day oral exposure study (via feed) in 9-week old male Wistar rats, 1-1000 ppm. OECD TG 407) | **No steatosis** in Wistar male **rats** |  | (Schmidt et al., 2016) | Initial reduction of food consumption, reduced bodyweight in first week of exposure at 1000 ppm (=high dose). Haematological parameters were unaltered. No treatment-related gross pathological findings were noted, but absolute and relative liver weight was increased (30% absolute, 40-45% relative) in highest dose group. In the liver, mild centrilobular hypertrophy of hepatocytes was reported in most animals at the highest dose. Anisonucleosis was observed in all rats at the two highest dose levels, but not at intermediate or lower levels. | Very high quality study (GLP, principally following OECD TG 407) |
| **Tebuconazole** | 107534-96-3 | 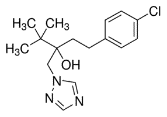 | triazole fungicide (plant pathogenic fungi) | **SUMMARY** | **Positive (tentative)** | CYP51 (target of triazole fungicides; mode of fungicidal action) |  | **Very Strong weight of evidence** for tebuconazole causing (general) hepatotoxicity, but  **Moderate weight of evidence** to support tebuconazole causing primary hepatic steatosis in vitro.  Rodent in vivo studies included for weight of evidence assessment followed (principally) OECD Test Guidelines and GLP standards, but offer some conflicting data:  Tebuconazole is reported to induce fatty changes in the liver (steatosis not specified) in vivo (Lichtenstein et al., 2020), based on OECD TG 417 (toxicokinetics) studies submitted to EFSA for pesticide active substance risk assessment. Conversely, a study with Wistar rats principally following OECD TG 407 (repeated dose 28-day oral toxicity study) reported hepatocellular hypertrophy, but no steatosis/hepatic lipid accumulation (Schmidt et al., 2016), which is also in line with the conclusion on liver effects of the triazole fungicide class (EFSA, 2009).  The reviewed (rodent) **in vivo data does not support tebuconazole causing primary hepatic steatosis**.  Tebuconazole is prioritised for inclusion in the preliminary proficiency chemical list due to the availability of human in vivo data (experimental exposure) for toxicokinetics and its extensive use in farming. (Tri)azole fungicides are also used as pharmaceuticals, however, due to the limited number of industrial and agrochemicals with human in vivo data, tebuconazole is being proposed as a chemical group representative chemical; evidence from pharmaceutical chemicals (ketoconazole, see below) was used as mechanistic supporting information. | Reasonable level of in vivo literature available, but high level of uncertainty to conclude on steatosis induction in vivo. |
|  |  |  |  | Human, in vitro (differentiated HepaRG cells, 24-72 h, 1-200 µM) | Strong induction *in vitro* (HepaRG) |  | (Lichtenstein et al., 2020) | Peak (≈3-fold induction) at 60-100 µM, then decrease at higher concentrations. LOEC estimated at ≈20-30 µM | Very high confidence |
|  |  |  |  | Human, in vitro (HepG2 and HepaRG cells, 24 h – 6 d, 1.25-20 µM) | Triglyceride accumulation in HepaRG (LOEC 5 µM) | In HepaRG: CAR, PPARα, **PXR**  in HepG2: CAR, LXRα, **PPARα (antagonism),** **PXR**  10 µM tebuconazole, HepaRG microarray: SLC27A5, GPD1, INSIG1, TXNIP, CYP19A1, CYP2E1, PLIN2, RGCC, IL1B, G6PC, HMGCR, CYP4A11.  1.25-20 µM tebuconazole, HepaRG qPCR (significant, with concentration-response): AKR1B10, **ChREBP**, **FASN**, GPD1, INSIG1, **SREBF1** | (Knebel et al., 2019a) | Induction of steatosis-related genes and triglyceride accumulation in HepG2 and HepaRG cells upon exposure to tebuconazole and propiconazole; propiconazole seems to be slightly more potent in steatosis induction (despite no difference in LOEC), resulting in higher fold-induction at 20 µM (≈2.8-fold vs. 2.3-fold; tested by Adipored staining, confirmed by GC-FID. Especially accumulation of longer-chain fatty acids: C52 and C54). **Knock-out of PXR, but not CAR, in HepaRG abolished triazole-induced lipid accumulation.**  Positive/sensitivity control: mixture of oleic acid and palmitic acid (1:1; 225 µM) induces approx. 2.8-fold induction. | Strong confidence |
|  |  |  |  | Human, in vitro (HepG2 and differentiated HepaRG, 1.25-40 µM, 24 h) | Not assessed | AhR agonism  CYP1A2 (enzyme activity induction in human only, not in rat), CYP1A1 | (Knebel et al., 2019b) | To identify influence of different xenobiotic-sensing nuclear receptors, several HepaRG knockout sub-cell lines were used in addition to the WT.  Liver samples from a previous study in Wistar rats (Schmidt et al., 2016) were used as well.  For some experiments, test chemicals were pre-incubated with rat S9 mix to mimic hepatic metabolism for 30 min, followed by 4 h exposure of HepG2 cells, then medium was exchanged for chemical-free medium and cells were cultivated for 20 more hours.  In rat liver*, Cyp1a1* and Cyp*1a2* gene expression was significantly increase at 1000 ppm; enzyme activity (by EROD assay) was only increased for Cyp1a1, not for Cyp1a2.  In human cells, CYP1A1 gene expression was significantly increased (LOEC HepG2: 2.5 µM; HepaRG: 1.25 µM); CYP1A2 gene expression was significantly increased (LOEC HepaRG: 1.25 µM, HepG2 not reported) and all tested concentrations (5-40 µM) significantly induced CYP1A2 enzyme activity. |  |
|  |  |  |  | Human, in vivo. Epidemiology (n=7) | (not assessed) |  | (Fustinoni et al., 2014) | Analysis of urinary excretion, not of liver pathology.  Median dermal exposure levels of 6180 and 1020 µg calculated from tebuconazole contamination of coveralls. |  |
|  |  |  |  | Human, in vivo. Experimental exposure (n= 3 male/ 3 female, single oral dose: 1.5 mg, single dermal dose: 2.5 mg during 1 h) | (not assessed) |  | (Oerlemans et al., 2019) | Analysis of urinary excretion, but no information on liver biochemistry, or pathology. |  |
|  |  |  |  | Review for regulatory purposes | **No steatosis**. Liver as the primary target organ for toxicity, but no lipid accumulation | CYP51 (target of triazole fungicides; mode of fungicidal action) | (EFSA, 2009) | Risk assessment for a selected group of pesticides from the triazole group (including cyproconazole and tebuconazole) to test possible methodologies to assess cumulative effects from exposure through food from these pesticides on human health; majority of studies included are rodent in vivo studies.  Studies, including **2-year chronic exposure GLP studies,** **do not indicate lipid accumulation in the liver**. Increased relative liver weight is reported frequently, and one of the most sensitive toxicological endpoints. However, this could be explained by observed hepatocyte hypertrophy and swelling; no lipid accumulation or steatosis was reported. (Tri)azol fungicides act by inhibiting CYP51, which in mammals is involved in cholesterol biosynthesis. Other CYP enzymes are affected as well (including CYP2A and CYP3A family), and this can affect steroid hormone synthesis and impact upon the (hepatic) metabolic balance, particularly metabolite flows between oxidases, including mixed-function oxidases. | Very high confidence and relevance  Also for conclusion on triazole fungicide chemical group |
|  |  |  |  | Review | Fatty changes *in vivo* (rodent) |  | (Lichtenstein et al., 2020) | Data reviewed based on test guideline studies according to OECD TG 417. Data source: list of endpoints on residues, active substance.  Fatty changes classified based on “Cumulative Assessment Group 2b: fatty changes” (Nielsen et al., 2012). Also, high oral absorption and high liver residue in vivo. | Very high confidence; literature data based on guideline studies |
|  |  |  |  | Rat, in vivo (28-day oral exposure study (via feed) in 9-week old male Wistar rats, 1-1000 ppm. OECD TG 407) | **No steatosis** in Wistar male **rats** |  | (Schmidt et al., 2016) | 28-day oral exposure study (via feed) in 9-week old male Wistar rats (principally following **OECD TG 407**): Haematological parameters were unaltered. No treatment-related gross pathological findings were noted, but a significant increase in relative liver weight (7%) at the highest dose was reported. In the liver, mild centrolobular hypertrophy of hepatocytes and vacuolisation was reported in all animals at the highest dose. Anisonucleosis was observed in all animals at the highest dose and 2/5 animals at 10 ppm, but not at intermediate or lower doses. | Very high quality study (GLP, principally following OECD TG 407) |
| **Ketoconazole** | 65277-42-1 | 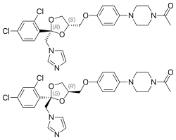 | imidazole fungicide, antiandrogen and antifungal pharmaceutical | **SUMMARY** | Uncertain | GR antagonist, CYP enzymes.  CYP51 (target of triazole fungicides; mode of fungicidal action) |  | **Strong weight of evidence for ketoconazole adverse effects on the liver, however the weight of evidence for causing primary hepatic steatosis/lipid accumulation is weak:** adverse human health effects included hepatitis, cirrhosis, and liver failure, but not steatosis (US FDA, EMA, 2013). However, human in vitro mechanistic data (mitochondrial dysfunction) are supportive of a potential to induce or contribute to the development of steatosis.  Stereoisomers may have different toxicological effects. (2R,4S)-(+)-ketoconazole (image top)/ (2S,4R)-(−)-ketoconazole (image bottom).  Other pharmaceutical (tri-)azoles were screened (voriconazole, miconazole fluconazole), but due to time constraints, only three (tri-)azole class representative chemicals with substantial literature information available were included in the more detailed literature review. |  |
|  |  |  |  | Human, in vitro (HepG2 and differentiated HepaRG; 1-100 µM; 24-48 h)  Rodent, in vitro (isolated mouse liver mitochondria from male C57BL/6 mice; 1-100 µM; 24-48 h) | Not concluded | Mitochondrial dysfunction (impaired membrane potential, disruption of electron transport chain enzyme complexes, decreased mtDNA, apoptosis) | (Haegler et al., 2017) | Cytotoxicity in HepG2: ≥50 µM; decreased intracellular ATP at ≥10 µM. after 24 h exposure, mitochondrial membrane potential was decreased and electron transport chain enzyme complexes impaired, accumulation of superoxide anion, decreased mtDNA, induction of apoptosis. This was exacerbated by vitamin B12 antagonist pre-treatment or ATP depletion.  Cytotoxicity in HepaRG: ≥100 µM, slightly accentuated by CYP3A4 and CYP1A2 induction  In isolated mouse liver mitochondria: impaired membrane potential and complex I activity | High relevance |
|  |  |  |  | Human, in vitro (undifferentiated HepaRG in 2D or 3D spheroid culture; exposure after 7 d pre-growth for 1-7 d; 9-step two-fold dilution from 640 µM) | Not assessed |  | (Ott et al., 2017) | High-thoughput HepaRG CYP induction assay. CYP3A4 activity was assessed by P450-Glo Luciferin-IPA assay (Promega), CYP1A activity by EROD assay, cytotoxicity as ATP content by CTG assay (Promega).  Therapeutic maximum concentration in vivo: 11.3 µM.  Cytotoxicity: IC50 in 2D: 403.6 µM (24 h), 81.0 µM (7 d) IC50 in 3D: 169.4 µM (24 h), 47.9 µM (7 d) | High confidence, moderate relevance (high for cytotoxicity)  The authors declared a conflict of interest for the micromolds used to produce the HepaRG 3D spheroids |
|  |  |  |  | Regulatory advice | Liver injury/ hepatotoxicity (but not specifically steatosis) | CYP3A4 inhibition (very potent)  Antiandrogen effects through interference with steroidogenesis | (US FDA, EMA, 2013) | Serious hepatic injury was identified as a major risk with **oral** ketoconazole medication in human, especially with high doses administered over a short time, or low doses over longer time.  Adverse outcomes leading to the suspension of marketing authorisation for oral ketoconazole in the EU (by EMA), and for revised recommendations for oral ketoconazole treatment by the US FDA were reported cases of hepatitis, cirrhosis, and liver failure with fatal outcomes or requiring liver transplantation. Hepatotoxicity occurred between 1-6 months after onset of treatment with the recommended dose of 200 mg/d, but has also been reported earlier than 1 month after commencing of treatment | Very high confidence and relevance. |
|  |  |  |  | Human, in vivo (epidemiology; retrospective cohort study to identify liver injury following oral antifungal medication, n=69,830, 20-79 years old free of liver and systemic diseases) | **Markedly increased risk** of acute hepatic injury. Steatosis not assessed (due to lack of clinical code for inclusion, at that time). |  | (García Rodríguez et al., 1999) | Incidence of acute liver injury with ketoconazole: 134.1 per 100,000 person-months.  Of the investigated antifungals, ketoconazole was associated with the highest relative risk of acute liver injury (228.0, 95% CI: 33.9-933.0).  Ketoconazole treatment included in cohort: n=632 women, n=429 men.  Two cases of hepatic injury were recorded following ketoconazole treatment (one male, one female); the clinical pattern of liver injury was hepatocellular and cholestasis.  Despite the large size of the recruited cohort, the number of detected cases associated with antifungal treatment (n=6) is low, therefore effect modification by gender, age or ethnicity could not be concluded. | Very high confidence and relevance; basis for change in recommendations for oral ketoconazole use by US FDA and EMA |
|  |  |  |  | Human, in vivo (clinicopathological study; n=55: 46 female, 9 male)) | **No steatosis.** Hepatic injury likely (91%) caused by ketoconazole treatment, but hepatocellular or cholestatic pattern; not steatosis. |  | (Stricker et al., 1986) | Causal relationship of non-fatal hepatic injury being linked to ketoconazole treatment was “probable” in 27 cases, “possible” in 23 (i.e., “likely” in 50 cases).  Hepatic injury was frequently accompanied by jaundice (44%); eosinophilia, fever, and rash were uncommon (<10%).  Biochemical characterisation of hepatic injury: hepatocellular (54%), cholestatic (16%), mixed cholestatic-hepatocellular (30%). Histological examination of 14 specimen showed a hepatocellular pattern (57%) with extensive centrilobular necrosis and mild-moderate bridging; in 43% of histological specimen cholestasis predominated.  A metabolic idiosyncratic mechanism of ketoconazole-induced hepatic injury is hypothesised. |  |
| **Benzo[a]pyrene** | 50-32-8 | 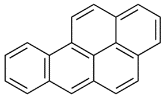 | polycyclic aromatic hydrocarbon | **SUMMARY** | Yes, tentative positive | CYP1A1 |  | **Moderate weight of evidence** supporting induction of primary hepatic steatosis in vitro (including mechanistic support and transcriptomic/metabolomic data). **Moderate-weak**, but not contradicting weight of evidence in vivo (rodent and human). **Strong weight of evidence** supporting induction of steatosis in **Amphibians in vivo**, though human relevance and predictivity is uncertain (e.g., due to substantial species-specific structural differences in PPARγ ligand binding pocket). |  |
|  |  |  |  | Human, in vitro (differentiated HepaRG cells, 72 h, 1.5×10^-10^ - 1×10^-5^ M) | No steatosis at non-cytotoxic levels |  | (Landesmann et al., 2012) | Decreased cell count, nuclear staining intensity, and nuclear area, but no ROS induction (at approx. 0.01 µM; no ROS production up to 200 µM) |  |
|  |  |  |  | Human, in vitro (HaCaT cells (wild-type and AhR knock-down), 24-48 h, 0.1-3.5 µM) | Not concluded, but B[a]P induced changes in lipid metabolism in an AhR-dependent fashion | CYP1A1 (and, to a minor extent, CYP1B1)  Strong induction of CYP1A1 and CYP1B1 protein expression at 1 and 3.5 µM (weak induction at 0.1 µM) | (Potratz et al., 2016) | In HaCaT wild-type cells, cell viability was reduced to ≈80% at 20 µM.  Substantial B[a]P-dependent CYP induction observed at 1 and 3.5 µM (weak induction at 0.1 µM) on both, protein and gene expression level.  PCaa_C36:2 (phosphatidylcholine, diacyl, 36 C atoms, 2 double bonds) levels of cells were increased after B[a]P exposure and identified as a potential biomarker for canonical AhR activation after PAH exposure. | High confidence, but lower relevance towards steatosis |
|  |  |  |  | Human, in vitro (differentiated nonsteatotic and steatotic (150 µM stearate/oleate-induced) HepaRG cells; 2 weeks, 2.5-5 µM) | yes | Mitochondrial dysfunction (mitochondrial respiration ↓), oxidative stress (ROS)  AhR activation  **APOA4, PLIN1**, CYP2E1 (↑), CYP3A4 (**↓**) | (Bucher et al., 2018a) | Deleterious effects of B[a]P/ethanol co-exposure were at least partly dependent on B[a]P-mediated activation of AhR, particularly with respect to impairment of mitochondrial respiration.  Steatohepatitis-like appearance was more pronounced after B[a]P/ethanol co-exposure (as opposed to each chemical alone), and steatotic HepaRG cells were more susceptible than the non-steatotic model.  **B[a]P induced triglyceride accumulation in both, steatotic and non-steatotic cells was lower than ethanol-induced TG accumulation.**  APOA4 and PLIN1, biomarkers of NAFLD, paralleled changes in triglyceride accumulation. | High confidence |
|  |  |  |  | Human, in vitro (differentiated nonsteatotic and steatotic (150 µM stearate/oleate-induced) HepaRG **cells**; 2 weeks, 1-2.5 µM) | yes | Inflammation increased in steatotic HepaRG (2.5 µM B[a]P): **IL6, IL1β**.  Metabolism: **CYP1A1**, CYP1A2 (reduced expression in steatotic cells), CYP1B1, **CYP2E1**  AhR signalling, inflammation, oxidative stress (ROS) | (Bucher et al., 2018b) | Compared to non-steatotic models, steatotic models exhibited higher cytotoxicity and stronger inflammatory response upon exposure.  In steatotic HepaRG (i.e., stearate/oleate fatty acid overload prior and during exposure) B[a]P detoxification via CYP enzymes was decreased, leading to higher levels/longer residence time of toxic B[a]P metabolites.  Cytotoxicity EC_10_ in HepaRG: 4.68±0.95 µM in steatotic cells, 9.12±2.24 µM in non-steatotic cells; cytotoxicity was exacerbated by co-exposure to ethanol.  Results were confirmed/complemented in vitro (hybrid human/rat WIF-B9 cells; 5 d, 10 nM) and in vivo (obese zebrafish larval test, 4-12 dpf. Exposure 5-12 dpf, 25 nM). | High confidence; complementary results observed in another mammalian in vitro cell model and a relevant zebrafish model. |
|  |  |  |  | Human, in vitro (oleic acid-stimulated HepG2 cells; 24 h, 5 µM) | Not concluded for B[a]P | CYP1A1 | (Huang et al., 2018) | CYP1A1, which expression is increased in oleic acid-stimulated HepG2 cells, catalyses (or is involved in the catalysis of) lipid peroxidation of lipids; B[a]P exposure exacerbated CYP1A1-mediated lipid peroxidation.  Cells were pre-treated with oleic acid (0.2 mM, 24 h) | Moderate confidence |
|  |  |  |  | Human, in vitro (SMMC-7721 cells; 1 month, 0.01-100 nM B[a]P) | Not concluded | Amino acid, carbohydrate, lipid, and nucleotide metabolism | (Ba et al., 2015) | Metabolomics study and network analysis revealed that amino acid, carbohydrate, and lipid metabolism pathways and the nucleotide metabolism pathway were influenced by prolonged B[a]P exposure.  B[a]P exposure caused a decline in the glycolysis process but enhanced the glycolytic capability of SMMC-7721 cells in vitro. | Problematic cell line: Contaminated. Shown to be a HeLa derivative (Rebouissou et al., 2017, Ye et al., 2015). Originally thought to originate from a hepatocellular carcinoma.  Lower confidence due concerns over cell line identity. |
|  |  |  |  | Human, in vitro (HepG2 cells, 3-60 h, 3 µM B[a]P) | Not concluded | RNA microarray: **TP53** (↑), **NRF2** (↑), apoptosis (↑), genotoxic stressor-response (↑); amine, sterol**, lipid & carbohydrate metabolism** (↓), transcription regulation (↓), nucleosome assembly (↓).  **Critical transcription factors for B[a]P effects: NF-κB, c-MYC, SRF, AP1, E2F1** | (van Delft et al., 2010) | Only one concentration was investigated, since previous studies showed that duration of exposure has a higher effect on gene expression profiles than dose; 3 µM were chosen as the lowest dose of B[a]P with a maximum accumulation of cells in S phase (higher concentration only extend the period of the S phase).  HepG2 efficiently cleared B[a]P from medium (1% remaining after 30-36 h, < LoD after 48 h).  No increased apoptosis was observed upon B[a]P exposure, but changes in cell-cycle timing. B[a]P induces DNA double strand breaks (as analysed by γH2AX foci) via DNA adducts.  B[a]P concentration in culture medium was verified after exposure by LC-MS; RNA integrity was analysed and used as a quality criterion for gene expression analyses  Steatosis/ lipid-accumulation-relevant mechanisms may have been missed, since the study focuses on carcinogenesis | High confidence |
|  |  |  |  | Hybrid human/rat, in vitro. (steatotic WIF-B9 hepatocytes, 3 h – 5 d, 10 nM B[a]P with/without 5 mM ethanol) | no conclusion on induction of steatosis, but B[a]P promotes apoptosis/ cell death in steatotic cells | **p53**  **AhR, ADH**, oxidative stress (ROS and **nitric oxide**)  CYP1 enzyme | (Tête et al., 2018) | Steatosis deceased CYP1 (EROD assay) enzyme activity upon B[a]P exposure and co-exposure.  B[a]P (co-)exposure exacerbated cell death in the steatotic in vitro cell model, suggesting in vivo aggravation of steatosis and progress to more severe stages, such as steatohepatitis. |  |
|  |  |  |  | Human, in vivo (epidemiology/ pilot metabolome-wide association study. N=30 serum samples) | Not concluded | Enriched pathways: linoleate metabolism, carnitine shuttle, (xenobiotic) drug metabolism, butanoate metabolism, glycerphospholipis metabolism, prostaglandin biosynthesis, methionine/ cysteine metabolism | (Walker et al., 2016) | 30 unidentified serum samples (out of approximately 60 million banked samples) were obtained from the US Department of Defense Serum Repository.  Serum samples were analysed for metabolites by LC-HRMS; free B[a]P in serum was determined by GC-MS.  Minimum, 25th percentile, median, 75th percentile, and maximum concentration (ng/mL) of 0.13, 1.45, 2.09, 3.18, and 37.2; corresponding to an average serum B[a]P concentration of 3.39 ng/mL±1.19 SE. Enriched pathways included linoleate metabolism (P=0.0017), carnitine shuttle (P=0.0035), drug metabolism (P=0.005), butanoate metabolism (P=0.009), glycerphospholipid metabolism (P=0.029), prostaglandin formation (P=0.029), and methionine/cysteine metabolism (P=0.043). | Small sample size (pilot study) |
|  |  |  |  | Human, in vivo (epidemiology pilot; n=147) | Not concluded (analytical chemistry focus) |  | (La Merrill et al., 2019) | Plasma samples were analysed for 30 (persistent) environmental pollutants by GC-MS/MS; study focused on South Asians living in America. | High confidence |
|  |  |  |  | Rodent, in vivo (adult C57BL/6 mice (n=24 exposed; sex not specified), single dose of B[a]P (5.0 µL, 5 mg/mL; ≈1.0 mg/kg bw) administered into the bronchial bifurcation by tracheal cannula. Observations up to 21 d post exposure) | Potentially positive | Lipid profile altered (no protein/transcriptomic biomarkers assessed) | (Li et al., 2020) | B[a]P exposure induced alteration of hepatic lipids by promoting the uptake from blood or the biosynthesis and transformation in the liver.  B[a]P exposure markedly altered glycerophospholipids, glycerolipids, and fatty acid metabolism in the mouse liver, with increasing of triacylglycerol (TG), phosphatidylinositol (PI) and PC, and decreasing of LysoPCs phosphatidylethanolamines (PEs), lysophosphatidylethanolamine (LysoPEs), free fatty acids (FFAs) and eicosanoids. (lipidomics study)  Excessive TGs detected in liver samples were mainly from the transportation of blood at the early period of B[a]P exposure; continuous accumulation of TGs was attributed to biosynthesis in middle/late exposure period. TG hydrolysis was enhanced in later exposure stages. | Moderate-high confidence, moderate relevance |
|  |  |  |  | Rodent, in vitro (primary rat hepatocytes from male Wistar rats or C57BI/6 mice; 48 h, 20 µM B[a]P) | Not concluded, but likely increase in steatosis under steatogenic diet-like conditions | **Mammalian *INDY* gene** (**SLC13AB**, encoding plasma membrane sodium citrate transporter, **NaCT**)  **AhR** (↑); **FAS** (↑)**, SREBP-1c** (↑), ACC (↑, minor induction) | (Neuschäfer-Rube et al., 2015) | m*INDY* ablation protects against diet-induced steatosis; m*INDY* is induced in patients with hepatic steatosis. Diet-induced hepatic steatosis is enhanced by AhR activation.  B[a]P exposure induced m*INDY* expression, supporting the hypothesis of m*INDY* being a downstream target of AhR signalling. m*INDY* induction increased citrate uptake and incorporation into lipids (sterols and fatty acids); probably exacerbated by B[a]P-mediated increased lipogenesis.  Additionally, B[a]P is alo a potential PXR ligand (and CYP3A4 inducer), though this is unlikely to be the primary cause of m*INDY* induction (also CAR activation is unlikely to have influenced the B[a]P-mediated m*INDY* expression). | High confidence |
|  |  |  |  | Amphibian, in vivo (*Xenopus tropicalis*, transgenerational study. F0 tadpoles exposed to 50 ng/L BaP (7 d old to mature adult stage/12 months)) | Yes, **steatosis sustained throughout F0, F1, and F2** | Enriched KEGG pathways in F1 (underlined: changes sustained until F2): “Steroid biosynthesis” (F1: 40.4-fold, p=1.12 E11; F2: 29.9, p=1.58 E-5), “Biosynthesis of antibiotics” (F1: 6.6-fold, p=1.36 E-8; F2: 5.3-fold, p=1.58 E-4), “Terpenoid backbone biosynthesis” (22.5-fold, p=5.22 E-5), “Metabolic pathways” (2-fold, p=1.09 E-4), “Pyruvate metabolism” (9.5-fold, p=0.007), “PPAR signalling pathway” (6.5-fold, p=0.02). | (Usal et al., 2021) | 5 F0 females and 10 F0 males were selected for mating for F1.  Hepatic steatosis was detected in F0, F1, and F2 adult females (males not examined); liver histopathology indicated progression of NAFLD to NASH.  In F1 and F2: down-regulation of lipid metabolism (especially cholesterol metabolism, fatty acid metabolism, and fatty acid transport), except for upregulated *slc27a6* (long-chain fatty acid transport across membranes).  F2 progeny from B[a]P exposed F0 frogs showed delayed metamorphosis and sexual maturity; females showed increased hepatosomatic (1.2-fold, p=0.043) and adiposomatic (1.8-fold, p=0.026) indexes; histopathology resembled NASH.  While F2 females were not insulin resistant, insulin secreting capacity was markedly reduced. Also, glucose tolerance and muscle glycogen stores were decreased. |  |
|  |  |  |  | Amphibian, in vivo (*Xenopus tropicalis*, transgenerational study. F0 female tadpoles (n=150) exposed to 50 ng/L BaP (7 d old to mature adult stage/12 months)) | Liver steatosis and hypertriglyceridaemia induced | Oxidative stress, ER stress  Enriched KEGG pathways (transcriptomics): “Metabolic pathways” (2.9-fold), “Biosynthesis of amino acids” (3.5-fold), “Steroid hormone biosynthesis” (3.7-fold); “Peroxisome” (3.7-fold), “Chemical carcinogenesis” (3.8-fold); “Carbon metabolism” (3.8-fold); “Metabolism of xenobiotics by cytochrome P450” (4.1-fold); “Glycine, serine and threonine metabolism” (7.8-fold); “Glyoxylate and dicarboxylate metabolism” (8-fold); “Protein export” (13.2-fold) and “Protein processing in endoplasmic reticulum” (5.4-fold) | (Regnault et al., 2018) | Exposure during early life stages resulted in metabolic syndrome (decreased glucose tolerance, liver steatosis, liver mitochondrial dysfunction, pre-diabetes (liver transcriptomic signature and pancreatic insulin hypersecretion)) and delayed metamorphosis in adults (F0). F1 progeny displayed decreased size and weight at metamorphosis.  F0 animals still displayed hepatic disorders and insulin secretory defect after 1 year depuration period.  Steatosis was assessed histopathologically; modifications observed upon B[a]P exposure correspond to NASH; despite extensive necrotic lesions no fibrosis was observed. |  |
| **Pemafibrate** | 848259-27-8 | 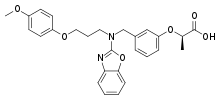 | Pharmaceutical | **SUMMARY** | **Negative**  **Inactive/ decrease** | **PPARα** (agonism) |  | **Strong weight of evidence** supporting no induction of steatosis in human in vivo. Mechanistic data, and pharmaceutical mode of action to lower abnormal blood lipid levels provide mechanistic support for pemafibrate not inducing primary hepatic steatosis.  While pemafibrate seems to be a more potent (~100x) pharmaceutical to treat hyperlipidaemia, fenofibrate was prioritised due to longer market authorisation. |  |
|  |  |  |  | Human, in vivo. (Randomized, double-blind, placebo-controlled clinical phase II trial; n=118. Clinical trial NCT03350165. 0.2 mg pemafibrate twice daily orally for 72 weeks) | no |  | (Nakajima et al., 2021) | **Liver fat content was not decreased by the intervention**, but liver stiffness was reduced at 48 and 72 weeks, with significant reduction in serum alanine aminotransferase (ALT) and low-density lipoprotein cholesterol (LDL-C). |  |
|  |  |  |  | Human, in vivo. (Randomized, double blind, active-controlled clinical phase III trial; n=223, 24-week study) |  |  | (Ishibashi et al., 2018) | Serum triglyceride levels decreased significantly. 0.2 mg/d and 0.4 mg/d pemafibrate were superior to fenofibrate (106.6 mg/d) in reducing serum TG. (liver outcome not assessed directly) |  |
|  |  |  |  | Human, in vivo (randomized, double-blind placebo-controlled multicentre clinical phase III trial; n=489) | Inactivity can be inferred due to a decrease of serum triglycerides and reduction in adverse liver/kidney effects |  | (Arai et al., 2018) | Study to examine equivalent efficacy of pemafibrate compared to maximum therapeutic dose of fenofibrate.  Pemafibrate efficacy was at least equivalent (or better) than fenofibrate, particularly with respect to reducing serum triglyceride levels. Pemafibrate intervention resulted in fewer adverse effects on kidney/liver-related laboratory results. |  |
|  |  |  |  | Meta-analysis of randomized clinical trials (2 clinical trials, total of 1623 patients) |  |  | (Ida et al., 2019) | Serum triglyceride concentration was decreased by pemafibrate similar to fenofibrate. Pemafibrate improved HDL and non-HDL (LDL) cholesterol levels and, by homeostasis model assessment, insulin resistance. Compared to placebo and fenofibrate treatment, pemafibrate significantly decreased hepatobiliary enzyme activity and total adverse events, but LDL cholesterol was higher. |  |
|  |  |  |  | In silico, molecular docking |  | **PGC-1α** (PPARα co-activator) | (Yamamoto et al., 2018) | In silico molecular docking studies (confirmed by in vitro luciferase assay) of pemafibrate and fenofibrate to hPPARα explain the high affinity and efficacy of pemafibrate (over fenofibrate) |  |
|  |  |  |  | Mouse, in vivo (STAM mouse model: induced NASH in male C57BL/6J mice; 6-week old; 21-day treatment: 0.1 mg/kg bw with high-fat diet. n=6 per group) | Reduction of NAFLD/NASH in mice without affecting triglyceride content | **VCAM-1** (↓), myeloid cell markers, inflammation- and fibrosis-related genes. PDK4 (↑; glucose vs fatty acid oxidation), LIPE, MGLL, **MOGAT1** (TG re-esterification), VLDLR, AQP9, GYK, **PNPLA2**, ABHD5 | (Sasaki et al., 2020) | Improved macrovesicular steatosis (but not biochemical steatosis score) and F4/80 positive macrophage accumulation. Enhanced TG hydrolysis and fatty acid β-oxidation (and re-esterification), increased number and reduced size of lipid droplets. RNA-seq analysis revealed enriched genes/pathways related to lipid metabolic processes were upregulated, while immune system processes were downregulated. |  |
| **Fenofibrate** | 49562-28-9 | 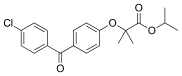 | Pharmaceutical (abnormal blood lipid levels) | **SUMMARY** | **Negative**  **Inactive/ decrease** | PPARα (agonism) |  | **Moderate weight of evidence** supporting no induction of steatosis in human in vivo. Mechanistic data, and pharmaceutical mode of action to lower abnormal blood lipid levels provide mechanistic support for fenofibrate not inducing primary hepatic steatosis. However, one study investigating liver changes by magnetic resonance indicates increased liver volume and some indication of potential increased liver lipid content (Oscarsson et al., 2018).  One human in vitro study reports partially conflicting data (with increased lipid accumulation at nM concentrations), but this was considered of lower relevance due to a lack of concentration-response kinetics, and lower confidence in high-throughput data, especially as human in vivo data are available.  While pemafibrate seems to be a more potent (~100x) pharmaceutical to treat hyperlipidaemia, fenofibrate was prioritised due to longer market authorisation. |  |
|  |  |  |  | Human, in vitro (differentiated HepaRG cells, 24 h, 3 d, 7d; 100 pM - 100 µM (cytotoxicity/ 100 pM – 1 µM (metabolomics)) | Equivocal.  Steatosis induced at 0.1-1 nM (increased total lipids), but no induction at ≥10 nM |  | (Franco et al., 2020) | Fenofibrate induces steatosis (increased total lipids between 0.1-1 nM; no difference to control at 10-1000 nM). **No accumulation of neutral lipids**.  LC50 24 h: 1915.00 ± 37.08 µM; LC50 7 d: 303.60 ± 13.40 µM | Moderate confidence |
|  |  |  |  | Human, in vitro (oleic acid-induced steatosis model of differentiated HepaRG cells. 14 d; 25 µM) | Reduced steatosis | Decreased expression: LXRα (no change in LXRβ), ALB, CYP2B6 | (Rogue et al., 2014) | Test concentrations correspond to IC10 values for cell viability by MTT test.  Reduction of triglyceride accumulation in oleic acid overload-induced steatosis in HepaRG cells in vitro. (Fenofibrate was less potent than rosiglitazone and glitazars in reducing steatosis.) |  |
|  |  |  |  | Human, in vitro (differentiated HepaRG cells, 14 d, 100 µM)) | (not concluded) |  | (Savary et al., 2015) | 100 µM fenofibrate (= IC10 in MTT cell viability assay; approx. 4-fold therapeutic plasma concentration) altered genes involved in lipid metabolism, inflammation/oxidative stress, cholesterol/bile transport and metabolism, amino acid metabolism, carbohydrate metabolism, and xenobiotic metabolism (based on Ingenuity Pathway Analysis; details from gene array in publication). No conclusion on steatosis is given in this publication. |  |
|  |  |  |  | Human, in vitro (HepG2 and differentiated HepaRG, pre-loaded for 14 h with 62 µM oleate palmitate (2:1); 62.5-500 µM, 24 h) | Induction, very strong (up to >500% in HepaRG) | Oxidative stress (ROS), decreased mitochondrial membrane potential | (Tolosa et al., 2016) | Exposure of cells to test chemicals was conducted in fatty-acid-free medium.  Fenofibrate is included as a steatogenic chemical (Drug-induced liver Injury category: not assigned), also causing mitochondrial impairment and apoptosis  In HepaRG: Significant lipid accumulation at ≥125 µM accompanied by increased oxidative stress/ROS production and decreased membrane potential. Cell viability was impaired at 500 µM. | Very high confidence and relevance |
|  |  |  |  | Human, in vivo. (Randomized, double blind, active-controlled clinical phase III trial; n=223, 24-week study) | No assessment of liver outcome, but decreased serum triglyceride levels |  | (Ishibashi et al., 2018) | Serum triglyceride levels decreased significantly. 0.2 mg/d and 0.4 mg/d pemafibrate were superior to fenofibrate (106.6 mg/d) in reducing serum TG. (liver outcome not assessed directly) |  |
|  |  |  |  | Human, in vivo (double-blind, randomised, placebo-controlled clinical study, n=78 overweight or obese individuals with NAFLD and hypertriglyceridemia. 4 g PUFA for 12 weeks) | Not concluded on steatosis specifically, but **increased liver and liver lipid volume** |  | (Oscarsson et al., 2018) | Clinical study on the effects of an omega-3 carboxylic acid supplement (4 g/day; containing DHA, EPA, and other fatty acids; n=25), 200 mg/day fenofibrate (n=27), or placebo (n=26) treatment in NAFLD. Double-blind parallel-group study.  Upon conclusion of the study, liver proton density fat fraction was not significantly different between treatment groups. **Liver and pancreas volume** were significantly increased compared to placebo, and were positively correlated (rho 0.45, p=0.02). Compared to PUFA treatment, fenofibrate **increased total liver fat and liver volume**.  Serum triglycerides were decreased; as well as plasma DHA and increased plasma acylcarnitine and butyrycarnitine, estimated delta-9 desaturase activity and urine F2-isoprostane concentration. | High relevance, high confidence (smaller sample size, but thorough assessment; magnetic resonance imaging) |
|  |  |  |  | Human, in vivo. (Randomized, double-blind placebo-controlled multicentre clinical phase III trial; n=489) |  |  | (Arai et al., 2018) | Doses administered were: pemafibrate (administration twice daily): 0.1 mg/d (n=40), 0.2 mg/d (n=120), 0.4 mg/d (n=80); fenofibrate (administration once daily): 100 mg/d (n=80), 200 mg/d (n=120); or placebo (n=40). Administration period was 12 weeks with 4-week follow-up period.  Non-inferiority and/or superiority of pemafibrate over fenofibrate was shown, particularly with respect to reducing serum triglyceride levels. Pemafibrate intervention resulted in fewer adverse effects on kidney/liver-related laboratory results. | High confidence, moderate-high relevance |
|  |  |  |  | Meta-analysis of randomized clinical trials (2 clinical trials, total of 1623 patients) | Not concluded on liver, only blood/plasma measured |  | (Ida et al., 2019) | Serum triglyceride concentration was decreased by pemafibrate similar to fenofibrate. Compared to placebo and fenofibrate treatment, pemafibrate significantly decreased hepatobiliary enzyme activity and total adverse events, but LDL cholesterol was higher. |  |
| **Pioglitazone** | 111025-46-8 | 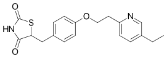 | Pharmaceutical, anti-diabetic drug | **SUMMARY** | Negative  Decrease/ inactivity in healthy population can be inferred from decreased NASH and steatosis in diabetic patients in clinical trials |  |  | Meta-analyses of (human in vivo) clinical studies indicate pioglitazone **improves the hepatic score** in (diabetic) patients with NASH; in particular improvement was seen in steatosis and lobular inflammation.  These two studies are of high confidence and were therefore utilised as a primary source of information (full-text evaluation of references was restricted where pharmaceutical mechanisms are well understood. Rosiglitazone (see below) is the thiazolidinedione with least contraindications that proceeded to market and is therefore considered a better suited candidate to be included for preliminary proficiency testing of metabolism disrupting chemicals. Therefore, the **weight of evidence is indicated as moderate**. |  |
|  |  |  |  | Meta-analysis of randomized clinical trials (10 studies, n=964) |  |  | (Singh et al., 2015) | Pentoxifylline and obeticholic acid improve fibrosis, and vitamin E, thiazolidinediones (TZDs; rosiglitazone or pioglitazone), and obeticholic acid improve ballooning degeneration in patients with NASH. TZDs are superior to vitamin E on improving steatosis and lobular inflammation. |  |
|  |  |  |  | Meta-analysis of randomized clinical trials (4 studies, n=334) |  |  | (Boettcher et al., 2012) | TZDs (rosiglitazone and pioglitazone) improved ballooning degeneration, lobular inflammation, and necroinflammation. Improvement in fibrosis was only marginally significant for pioglitazone only (not for rosiglitazone). TZD treatment increased total body fat. |  |
| **Rosiglitazone** | 122320-73-4 | 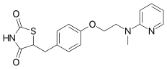 | Pharmaceutical, anti-diabetic drug | **SUMMARY** | **Negative**  **Decrease/ uncertain** | PPAR agonist (esp. **PPARγ**) |  | **Strong weight of evidence** from **human in vivo studies** (clinical trials, meta-analyses) supports **decreased lipid accumulation in the liver**, improvement of NASH, or at least no induction/increase in lipid accumulation in the liver. The conclusion for the activity of rosiglitazone towards primary hepatic steatosis is based on these studies.  Human in vitro studies (primarily conducted on HepaRG cells) have not necessarily looked for steatosis endpoints are conflicting, and partially contradicting the human in vivo observations (i.e. rosiglitazone is reported as a strong inducer of steatosis (Franco et al., 2020); this needs to be independently reproduced); the **in vitro evidence is considered weak due to uncertainties** and contradictions. However, extensive mechanistic data presented in in vitro studies supports an altered lipid metabolism.  **Moderate weight of evidence** for rosiglitazone **inducing steatosis** is available in **rodents (mice).** Predisposition to steatosis was stronger in obese mice/mice on a high-fat diet, and possibly stronger in females. Furthermore, lipid accumulation was dependent on the expression level of PPARγ receptors in the liver: low expression levels were protective of rosiglitazone-induced hepatic steatosis (Gao et al., 2016). Furthermore, there are interspecies differences in activation profiles of the PPARγ receptor, which could contribute to the interspecies differences indicated here (Garoche et al., 2021).  It should be noted, that outcomes in clinical trials focused on efficiency of rosiglitazone in (type 2) diabetic patients, as the primary pharmaceutical action is insulin sensitisation. Type 2 diabetes is already a strong indication for, and hallmark of, metabolic disruption, and therefore caution should be taken in the extrapolation of these results to the general, assumedly healthy, population.  Rosiglitazone is an established model PPARγ agonist and highly potent obesogen, well documented in the broader literature. Therefore, inclusion of rosiglitazone in the chemical selection is proposed, even though supporting data from human in vivo epidemiological and in vitro studies is (partially) conflicting. From the group of antidiabetic thiazolidinediones pharmaceuticals, rosiglitazone is prioritised due to having the most substantial literature body, and toxicological characterisation, and to convey mechanistic overlap between other key metabolic disruption processes/test methods under development, such as the hPPARα/γ reporter gene assays, or human in vitro adipogenesis assays. |  |
|  |  |  |  | Human, in vitro (differentiated HepaRG cells, 24 h, 3 d, 7d; 100 pM - 100 µM (cytotoxicity/ 100 pM – 1 µM (metabolomics)) | Strong induction (≥10 nM) |  | (Franco et al., 2020) | Rosiglitazone induces steatosis in differentiated HepaRG cells (increased total lipids above 10 nM). Moderate accumulation of neutral lipids (LOEC = 100 nM)  LC50 24 h: 116.20 ± 4.23 µM, LC50 7 d: 23.25 ± 1.51 µM |  |
|  |  |  |  | Human, in vitro (oleic acid-induced steatosis model of differentiated HepaRG cells. 14 d; 50 µM) | Reduction of oleic acid overload-induced steatosis (50 µM) | De novo lipogenesis (ELOVL6, FASN, PPARG, SCD1, **SREBP1** and **THRSP**), FAO and mitochondrial biogenesis (ACADL, **ACOX1**, **CPT1A**, **CPT2**, **ECH1**, CYP4A11, HADHA and PPARA), lipid hydrolysis and formation of lipid droplets (**ADFP**, **PLIN4** and LPL), lipoprotein synthesis (**APOC3**), nuclear receptors (**LXRα**/β, FXR, PXR and CAR) as well as other liver functions (ALB and **CYP2B6**) | (Rogue et al., 2014) | Test concentrations correspond to IC10 values for cell viability by MTT test.  PPAR agonists decreased triglyceride accumulation caused by oleic acid overload by up to 50%. Fatty acid oxidation genes were induced, while many genes involved in de novo lipogenesis were downregulated. Also, FXR, LXR and CAR nuclear receptor expression was modulated upon 2-week exposure. (In contrast, rosiglitazone was found to induce accumulation of neutral lipids in murine hepatocytes in vivo) |  |
|  |  |  |  | Human, in vitro (differentiated HepaRG cells, 70 µM, 14 d exposure) | Not concluded/evaluated (focus on gene expression analysis) |  | (Savary et al., 2015) | Exposure of differentiated HepaRG cells for 14 d to 70 µM rosiglitazone (= IC10 in MTT cell viability assay; approx. 70-fold therapeutic plasma concentration) altered genes involved in lipid metabolism, inflammation/oxidative stress, cholesterol/bile transport and metabolism, amino acid metabolism, carbohydrate metabolism, and xenobiotic metabolism (based on Ingenuity Pathway Analysis; details from gene array in publication). No conclusion on steatosis is given in this publication. |  |
|  |  |  |  | Human, in vitro (differentiated HepaRG cells and primary human hepatocytes, 50 - 150 µM, 24 h exposure) | Not concluded | Upregulated in PHH & HepaRG: CD36, SLC27A4, SLC27A2, ACSL1, ACSL5, FABP3, **FABP4**, CPT1A, HADHA, FGF21, PEX11A, **ADFP**, CIDEC, PLIN1, PLIN4, ANGPTL4, VLDLR, AKR1B1, AKR1B10, CYP1A1, **CYP2B6**, **CYP3A4**, CYP3A7, PLA1A, IL1B, ABCB4, ABCC2, **HMOX1**, POR, OASL, MBL2, CD14, IRF7, SGK2  Upregulated in PHH & HepaRG: BDH1, ADH1B, EPHX2, ABAT, AGXT2, CTH, GLS2, HAL, HPD, OTC, TAT, CXCL10, SAA4, CYP7A1, CAV1, SLC10A1  (in bold: microarray results validated by qPCR. Further: PDK4, FABP1, ALB, ALD-B, PPARA, PPARG1) | (Rogue et al., 2011) | 150 µM rosiglitazone reduced the intracellular ATP content significantly (to ≈80%).  Comparative gene expression profile of the response of primary human hepatocytes and/or HepaRG cells to rosiglitazone (50-150 µM) treatment. |  |
|  |  |  |  | Human, in vitro (undifferentiated HepaRG in 2D or 3D spheroid culture; exposure after 7 d pre-growth for 1-7 d; 9-step two-fold dilution from 208 µM) | Not assessed |  | (Ott et al., 2017) | High-throughput HepaRG CYP induction assay. CYP3A4 activity was assessed by P450-Glo Luciferin-IPA assay (Promega), CYP1A activity by EROD assay, cytotoxicity as ATP content by CTG assay (Promega).  Therapeutic maximum concentration in vivo: 1.0 µM.  Cytotoxicity: IC50 in 2D: 4.4 µM (24 h), 5.0 µM (7 d) IC50 in 3D: 1.6 µM (24 h), 19.0 µM (7 d)  Interlaboratory IC50 comparison: 121.8-144.5 µM (14 h, 3D HepaRG), 1.6 µM (24 h, 3D HepaRG), 352 µM (24 h, 3D rat hepatocytes), 326 µM (5 d, 3D rat hepatocytes) | High confidence, moderate relevance (high for cytotoxicity)  The authors declared a conflict of interest for the micromolds used to produce the HepaRG 3D spheroids |
|  |  |  |  | Human, in vivo (Therapeutic intervention, n=25, biopsy-proven NASH; 4 mg rosiglitazone twice daily for 48 weeks) | Decreased steatosis (improved NASH/ necroinflammatory score) |  | (Neuschwander-Tetri et al., 2003) | Mean global necroinflammatory score significantly improved with treatment and biopsies of 10 patients (45%) no longer met published criteria for NASH after treatment. Treatment also improved serum ALT and insulin sensitivity. Serum ALT rebounded to nearly pre-treatment levels within 6 months of completing treatment, but improvement of insulin sensitivity was more sustained. |  |
|  |  |  |  | Human, in vivo (Therapeutic intervention, n=20, biopsy-proven NASH, 4 mg rosiglitazone twice daily for 48 weeks) | Decreased macrovesicular steatosis, but not microvesicular steatosis |  | (Caldwell et al., 2007) | Rosiglitazone therapy did not affect the number of mitochondria, but increased occurrence of crystals in mitochondria. Macrovesicular steatosis, but not microvesicular steatosis, and Mallory bodies were significantly decreased (improved) by treatment. |  |
|  |  |  |  | Meta-analysis of randomized clinical trials (10 studies, n=964) | Decrease of steatosis |  | (Singh et al., 2015) | Pentoxifylline and obeticholic acid improve fibrosis, and vitamin E, thiazolidinediones (TZDs; rosiglitazone or pioglitazone), and obeticholic acid improve ballooning degeneration in patients with NASH. TZDs are superior to vitamin E on improving steatosis and lobular inflammation. |  |
|  |  |  |  | Rodent, in vivo (female C57BL/6J mice (6 per treatment group); exposure via drinking water: 500 nM ROSI from 7 days before mating throughout pregnancy) | Yes, more pronounced in females |  | (Chamorro-García et al., 2013) | F0 (dams) were exposed via drinking water, F1 animals were exposed in utero, F2 animals were exposed as germ cells in F1 mice (in utero), F3 animals were unexposed. Sibling inbreeding was avoided; 6 couples per exposure group were propagated to the next generation; 4-5 litters per exposure group were included in the analysis.  Lipid accumulation in liver observed **only in female liver in F1**, not in males or in F2/F3. | High confidence |
|  |  |  |  | Rodent , in vivo. (male C57BL/6 mice, 20 mg/kg bw (non-obese mice)/ 5 mg/kg bw (obese mice) twice weekly for 9-15 weeks. Preventive study: 5 mg/kg bw daily for 3 weeks) | Potentially yes. Steatosis was induced by rosiglitazone in mice on a high-fat diet/in obese mice, and was dependent on PPARγ expression levels in the liver. | **PPARγ**, **FAS**, **SCD1**, UCP2, HSL, ATGL, **CD36**, **FABP4**, **MGAT1**, DGAT1 | (Gao et al., 2016) | In mice on a high-fat diet, susceptibility to rosiglitazone-induced steatosis was dependent on PPARγ expression levels: low levels of PPARγ expression (associated also with higher levels of CD36, FABP4, and MGAT1 expression) in the liver were protective against, but higher levels of PPARγ expression in the liver exacerbated liver steatosis in obese mice.  In obese mice, 20 mg rosiglitazone/kg bw showed marked toxicity, resulting in weight loss. |  |
| **Bis(2-ethylhexyl) phthalate (DEHP)** | 117-81-7 | 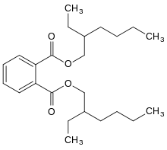 | Plasticizer, metabolite | **SUMMARY** | **Negative**  **Inactive/ potential increase** in hepatic (neutral) lipid accumulation and steatosis; mainly mediated via metabolite MEHP |  |  | **Moderate weight of evidence** for no effect of DEHP on steatosis in human in vivo. Though several in vivo epidemiological studies were retrieved and analysed, steatosis/ lipid accumulation in the liver was not assessed or reported, and the primary focus was on body weight/ BMI, abdominal fat, or analytical chemistry monitoring exposure levels. For DEHP and its main metabolite MEHP studies either reported no association, or a positive association with (increased) body weight/ BMI, or abdominal fat. In addition to human in vivo epidemiological data, a non-human primate study (Satake et al., 2010) was retrieved and included, where 28-day oral exposure to 1 g DEHP/kg bw/d did not induce liver steatosis.  **Strong weight of evidence** for steatosis induction is assigned to human in vitro, and rodent and zebrafish in vivo studies, also supported by mechanistic information on changes in biomarkers (e.g. PPARγ signalling pathway activation) in the liver. Interspecies differences in PPAR expression in the liver and affinity need to be considered.  Steatogenic activity through PPARγ is mediated through the DEHP metabolite, MEHP. Therefore, and to include active metabolites of environmental chemicals in the chemical selection, the evidence for steatogenicity of DEHP is supportive to include its primary metabolite, MEHP in the chemical selection (see below). Additional relevant literature that was abstract-screened, but did not undergo full-text screening due to time constraints and prioritisation of the metabolite MEHP over DEHP is listed at the end of this chemical. |  |
|  |  |  |  | Human, in vitro (differentiated HepaRG cells, 72 h, 1.5×10^-8^ - 1×10^-3^ M) | (not concluded) |  | (Landesmann et al., 2012) | No change in nuclear staining and **no ROS production** (72 h exposure; up to 200 µM).  No indication of hepatotoxicity. |  |
|  |  |  |  | Human, in vitro (differentiated HepaRG cells, 24 h, 3 d, 7d; 100 pM - 100 µM (cytotoxicity/ 100 pM – 1 µM (metabolomics)) | Increase (moderate)  Slight increase in total lipids (LOEC = 1000 nM), but very strong accumulation of neutral lipids |  | (Franco et al., 2020) | LC50 24 h: 163.70 ± 9.97 µM, LC50 7 d: 137.20 ± 11.41 µM  Slight concentration-dependent increase in total lipids (to max. 444.8 ± 32.3 µM); significant increase only at 1 µM.  Very **strong accumulation of neutral lipids** (LOEC=10 nM; >2-fold accumulation). DEHP was the strongest lipid homeostasis disruptor in the study of environmental obesogens, modifying lipid profiles in strong favour of obesity-related lipid species, such as triglycerides. | High relevance, but moderate confidence (high-throughput screening data) |
|  |  |  |  | Human, in vitro (HepG2 lipid overload model (500 µM oleic acid + 1% BSA), 48 h exposure, 2.5-200 µM DEHP) | Aggravation of steatosis. (DEHP promotes lipid accumulation in oleic acid-induced HepG2 cells) | **PPARα**, **SREBP-1c** (proteins; increased)  Redox homeostasis disrupted | (Zhang et al., 2017) | Cell proliferation increased in lower concentration conditions (≤50 µM), but viability impaired at higher concentrations (viability < 70% upon exposure to 200 µM DEHP); as a result: highest concentration excluded from further experimental steps. | Moderate confidence.  While the experimental side seems acceptable, the confidence in the results is decreased by low quality of English language in the publication. |
|  |  |  |  | Human, in vivo (epidemiology; subpopulation of NHANES study, n=6,005 women)) | (not concluded) But related positive associations of DEHP metabolites MBP and MEHP with BMI and waist circumference. |  | (Yaghjyan et al., 2015) | Women aged ≥18 years who were not pregnant and had no history of diabetes were included in this study; seven urinary phthalate metabolites were monitored and correlated with BMI, waist circumference, total cholesterol, triglycerides, HDL-C and LDL-C. All examined metabolites are metabolites of DEHP.  **BMI** was positively associated with monobutyl phthalate (MBP) and mono-2-ethylhexyl phthalate (MEHP) (odds ratio (OR) = 1.13; 95% confidence interval (CI), 1.03–1.23 and OR= 1.12; 95% CI, 1.03–1.23, respectively). **Waist circumference** was positively associated with MBP (OR = 1.13; 95% CI, 1.03–1.24). A higher ratio of MEHP to mono-(2-ethyl-5-hydroxyhexyl) phthalate (MEHP) was positively associated with both BMI (OR= 1.21; 95% CI, 1.09–1.34) and waist circumference (OR = 1.20; 95% CI, 1.10– 1.31). There were no other significant associations. | Very high confidence, moderate relevance |
|  |  |  |  | Human, in vivo (epidemiology. N=97 pregnant women) | (not concluded) | Oxidative stress | (Waits et al., 2020) | Urinary phthalate metabolites in the late third trimester were associated with oxidative/nitrosactive stress biomarkers | High confidence, but less relevant |
|  |  |  |  | Human, in vivo (epidemiology, n=2,884 non-pregnant children aged 6-19 years; subpopulation of 2003-2008 NHANES) | not concluded; no significant association of high-molecular weight phthalate and DEHP metabolites with BMI/obesity |  | (Trasande et al., 2013) | Urinary low molecular weight phthalate metabolites were associated with increased odds of overweight, obesity and increased BMI z-score among non-Hispanic black children. High molecular weight phthalates and DEHP metabolites did not show significant associations, and no significant associations were detected among other ethnic groups. | Very high confidence |
|  |  |  |  | Human, in vivo (epidemiology; Australian Barwon infant study, n=841 pregnant women, urine spot samples at 36 weeks pregnancy) | Not concluded/assessed |  | (Sugeng et al., 2020) | Study on phthalate exposure from the diet, use of (volatile) household products, household characteristics, and personal care products.  DEHP was measured by MEHP metabolite and detected in 33% of samples (LOD: 4.1 µg/L). geometric mean concentration: 4.0 µg/L (95% CI: 3.8-4.1 µg/L). | Very high confidence, low relevance |
|  |  |  |  | Human, in vivo (epidemiology; population-based prospective cohort. n=757-1128 mother-child pairs) | Perinatal DEHP exposure was not associated with childhood obesity or organ fat accumulation |  | (Sol et al., 2020a) (Sol et al., 2020b) | 2^nd^ trimester (but not 1^st^ or 3^rd^) maternal higher DEHP in urine was associated with a 0.18 (95% CI: 0.31-0.06) standard deviation score lower blood glucose concentration among boys aged 9.7 (±0.2) years.  Median maternal urinary phthalate concentrations in nM: 1^st^ trimester: 171.9 in boys, 174.2 in girls 2^nd^ trimester: 96.8 in boys, 89.4 in girls 3^rd^ trimester: 132.3 in boys, 146.8 in girls  Maternal urinary DEHP was not associated with childhood (measured at ~10 years) general or organ fat outcomes. |  |
|  |  |  |  | Systematic review and meta-analysis (**rodent**; n=31 included studies) | Not concluded, but positive association of early life exposure with increased fat pad weight, but not body weight |  | (Wassenaar and Legler, 2017) | Early life exposure to DEHP or MEHP was significantly associated with increased fat (pad) weight (mean difference 0.02, 95% CI: 0.00-0.03). The association with body weight was non-significant (mean difference: -0.14, 95% CI: -0.32-0.04). No meta-analysis could be conducted for other outcomes due to data scarcity (i.e. < 5 studies per outcome). | Very high confidence, moderate relevance |
|  |  |  |  | Review | Not concluded | PPARα | (Desvergne et al., 2009)  (Feige et al., 2007, Zoete et al., 2007) | Co-crystallisation of PPARγ with rosiglitazone or MEHP revealed similar binding patterns and interaction points with the PPARγ ligand binding domain. In contrast, the parent compound DEHP was neither able to enter the ligand binding pocket of PPARγ, nor to maintain stable contacts with the required protein moieties.  The DEHP metabolite MEHP, but not the parent compound, can activate PPARα as well as PPARγ. | High confidence and moderate relevance (structural biochemistry discussion, no information on tissue biology/toxicology)  Pivotal study for mechanistic understanding |
|  |  |  |  | Review/ commentary/ opinion | (not concluded) | **PPARα**, acyl CoA oxidase | (Willhite, 2001) | Discussion on interspecies differences in carcinogenicity risk assessment of DEHP and the (un-)suitability of rodent models for human health hazard identification/characterisation and subsequent risk assessment.  PPARα is identified as a critical regulator for peroxisome proliferation and subsequent carcinogenesis in rodents. However, rodents have more responsive peroxisome proliferator responsive elements (PPRE; regulating CYP4A1 and acyl-CoA oxidase expression) compared to human PPRE; the “human [acyl CoA oxidase] gene promotor is incapable of eliciting peroxisome proliferator-mediator gene expression as occurs in rats and mice”.  The author does not mention PPARγ and focuses solely on PPARα as an initiator of peroxisome proliferation.  Developmental toxicity observed in male offspring of prenatally exposed Sprague-Dawley rats (malformations of androgen-dependent organs: hypospadias, cryptorchidism, testicular agenesis) is not related to PPARα; characteristic testicular lesions were observed also in PPARα-KO mice. | High confidence in accuracy of the data presented, but reference only marginally relevant towards steatosis.  Reference considers mainly information from pre-2000, thus doesn’t take more recent advances in risk assessment into account. |
|  |  |  |  | Non-human primate, in vivo (Cynomolgus monkey, n=3 male, 4 female; 28 day oral exposure: 1 g/kg bw/d) | No steatosis, but slightly enlarged mitochondria in the liver. | PPARα agonism | (Satake et al., 2010) | Electron microscopical examination of liver histological specimen revealed enlarged mitochondria. No abnormality in peroxisome appearance was observed, but their number was significantly increased in females, both in the centrilobular and periportal area.  At termination, no changes were recorded for the hepatic fatty acid oxidation system or carnitine acyltransferase activity; carnitine palmitoyl transferase activity was slightly increased.  No hepatomegaly or hepatic proliferation were observed at the administered, very high, dose. | High confidence and relevance |
|  |  |  |  | Mouse, in vivo (SPF ICR male mice, 3 weeks old, exposure by gavage for 28 d. N=20 mice/group; 500 mg/kg bw/d, 1000 mg/kg bw/d) | Increased steatosis | **HIF-1α** induction (downstream targets: EPO, ET1, VEGF, Flk1, bFGF, Glut1, pdk1, I1cam, Flt1), PPARα, **PPARγ**, **FXR** (↓), **LXR** (↓), **SREBP1**, **RXR**, | (Zhao et al., 2020) | Other treatment groups were untreated (water), vehicle (corn oil), and DEHP + lycopene (5 mg/kg bw/d).  Only DEHP treatment alone induced significant histopathological changes in the liver, at both doses, but especially in the high-dose group. Oedema, structural abnormality, disordered hepatic plate, and steatosis were found in animals exposed to DEHP.  Biochemical alterations of DEHP exposure in serum were elevated AST/ALT, total protein, total and direct bilirubin, as well as total cholesterol, triglycerides, non-esterified fatty acids, and lipase. | Moderate confidence, but good size of exposure groups |
|  |  |  |  | Zebrafish, in vivo (HFD steatosis zebrafish larvae 4-9 dpf, exposure from 5 dpf to 2.56 nM DEHP and/or 43 mM ethanol) | Increased steatosis | Gene expression increased: *Cyp4t8,* ceruloplasmin, NF-κB, *AhR* | (Tête et al., 2020) | Pathological progression of liver steatosis (to a NASH-like state) alongside increased *Cyp4t8* (human CYP4A homolog).  Clear lipid accumulation was detected from 1 d of HFD feeding. 43 mM ethanol in medium result in 10 mM internal dose (previous data, not shown), 2.54 – 2.85 nM DEHP correspond to human exposure data.  HFD markedly increased DEHP/ethanol co-exposure toxicity, with ballooning, vacuolised hepatocytes, and cellular dropouts in liver tissue samples. |  |
|  |  |  |  |  |  |  |  | Additional relevant literature that was abstract-screened, but did not undergo full-text screening due to time constraints and prioritisation of the metabolite MEHP over DEHP:  (Zhang et al., 2012, Schaedlich et al., 2018, Melnick, 2001, Manteiga and Lee, 2017, Lv et al., 2016, Lapinskas et al., 2005, Kratochvil et al., 2019, Dong et al., 2017, Svensson et al., 1998, Buckley et al., 2016, Shoaito et al., 2019, Roberts, 1999, Faouzi et al., 1999, Zhao et al., 2016, Bai et al., 2019, Feige et al., 2010) |  |
| **Mono-ethylhexyl phthalate (MEHP)** | 4376-20-9 | 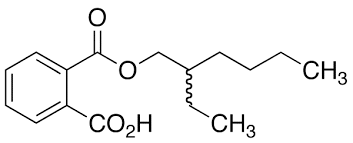 | Plasticizer metabolite | **SUMMARY** | **Strong positive** | PPAR signalling |  | **Strong weight of evidence** from human **in vivo epidemiological studies did not find (or report) an association of MEHP exposure with hepatic steatosis.** However, some studies report a positive association of MEHP exposure and body weight/BMI, or abdominal circumference, suggesting changes to the lipid homeostasis in tissues other than the liver, and thus inferring possible changes to lipid homeostasis in the liver as well.  **Strong weight of evidence** from human in vitro and animal in vivo studies suggesting **MEHP induces lipid accumulation** both, in hepatocytes and other cell lines. The apical endpoint of lipid accumulation is also supported by sound mechanistic data on several levels, including gene expression data and lipidomic analyses.  The conclusion on MEHP as a steatosis-positive chemical gives higher weight to in vitro studies, supported by inferred disrupted lipid homeostasis in other tissues from human in vivo studies.  MEHP is the bioactive metabolite of DEHP, and unlike DEHP it is a PPARγ agonist (in humans), providing strong mechanistic support for effects of MEHP exposure to (liver) lipid metabolism in general, and increased lipid accumulation more specifically. Therefore, we propose the inclusion of MEHP over DEHP in the provisional chemical selection list. However, if additional testing capacity is available, inclusion of both, DEHP as the parent chemical, and its metabolite MEHP might be informative to indicate and evaluate the metabolic capacity of a test system. |  |
|  |  |  |  | Human/rat, in vitro (human/rat hybrid pre-steatotic hepatocyte cell line WIF-B9, 5 d, 500 nM MEHP + 5 mM ethanol) | Increased steatosis | **AhR**-dependent decrease in alcohol dehydrogenase activity, **CYP4A1-3 (↑)**, CYP2E1 (reduced in steatotic cells), caspase/p53 apoptosis, oxidative stress | (Tête et al., 2020) | Cells were exposed to mono(2-ethylhexyl) phthalate (MEHP), the main metabolite of DEHP, in combination with ethanol in order to study if co-exposure exacerbates cell death and lipid accumulation.  2 d prior to exposure, cells were treated with fatty acids (450 µM oleic acid, 100 µM palmitic acid) complexed with albumin.  Cells showed a higher apoptotic and necrotic index in the steatotic state, but this was exacerbated by co-treatment of MEHP and ethanol (approx. 2-fold increase). |  |
|  |  |  |  | Human, in vitro (JEG-3 human trophoblast cell line. 24-72 h exposure to DEHP (up to 50 µM) or MEHP (up to 25 µM; 10 µM for lipidomics)) | Not concluded, but in trophoblast cells changes in lipidome were observed, including triacylglycerol accumulation | **PPARγ** (Kd=12 µM) | (Petit et al., 2018) | Lipidomic changes were observed in glycerolipids, glycerophospholipids and markedly elevated triacylglycerols.  Cell viability was impaired (<80%; NOEC) at <25 µM DEHP or <10 µM MEHP. For lipidomics, cells were exposed to 10 µM MEHP; the absolute concentration of MEHP remained ±constant during 72 h exposure (reduction from 11.6±2.1 µM at exposure to 9.9±1.8 µM after 72 h); loss of MEHP by sorption to the cell culture plastics cannot be excluded, but negligible leaching (0.375±0.04 µM) from plastics into DMSO-only medium was noted.  PPARγ was induced (measure in ELISA) after 24 h exposure: 1.40x at 1 µM, 1.35x at 5 µM, and 1.50x at 10 µM MEHP.  132 lipids were significantly different between control and MEHP treated JEG-3 cells, of these, 73 lipid species were triacylglycerols (increase of ~80% if MEHP-exposed cells). diacyl- and monoacylglycerols are downregulated.  Lipidomic changes observed with MEHP resemble those observed upon treatment of JEG-3 cells with rosiglitazone, a model PPARγ agonist. | Very high confidence and relevance |
|  |  |  |  | Human, in vitro (HepG2, 24-48 h; 0.8-100 µM) | Yes. Intracellular triglyceride accumulation (LOEC: 20 µM MEHP for both, 24 and 48 h exposure) | Gene expression after 24 h exposure:  SREBP-1c ↑, ChREBP ↑, FASN ↓, and SCD ~↓  Proteins expression: ACC1 (↑ 24 h; ↓ 48 h), FASN (↑ 24 h; ↓ 48 h), SCD (↑ 24 h; ↓ 48 h). SREBP-1c remained unchanged. | (Bai et al., 2019) | Cell viability <90% at ≥50 µM MEHP (24 h, 48 h); ~90% at ≥50 µM MEHP (72 h)  Notably, some biomarker genes showed significantly altered expression at (6 and) 24 h, but this change was diminished at 48 h (e.g. for SREBP 1c, FASN, and ChREBP). | High confidence and relevance |
|  |  |  |  | Human, in vitro (HepG2; 24 h, 4-100 µM) | Yes, intracellular fatty acid accumulation | Inhibition of fatty acid β-oxidation enzymes  Proteomics: acyl-CoA dehydrogenase family member 9 (ACAD-9), enoyl-CoA hydratase 1 (ECHS1), **HADH**, and **HSD17B10**. Further: ACOT9, TRAP1 | (Xu et al., 2020a) | Liver enzyme activity assay was performed with fresh liver preparation from 2-month-old C57BL/6J mice; enzyme reactions were conducted for 30 min at 37 °C.  Cell viability <90% at ≥100 µM. At 50 or 100 µM MEHP, there was no indication of apoptosis (Caspase-3). Therefore, 50 and 100 µM were used for lipidomics; 4 and 100 µM were used for chemical proteomics.  Lipidomics identified 140 dysregulated lipids of the subclasses fatty lipids (FA), glycerophosphocholines (PC), glycerophosphoethanolamines (PE), glycerophosphoserines (PS), glycerophosphoglycerols (PG), glycerophosphates (PA), glycerophosphoinositols (PI), monoradylglycerolipids (MG), diradylglycerolipids (DG), triradylglycerolipids (TAG), ceramides (Cer), sphingomyelins (SM), carnitines (Car), acylcarnitines (ACar), hexosyl ceramides (HexCer), and cardiolipins (CL); the majority (132 out of 140) were more abundant upon MEHP exposure. Lipid species with a concentration-response change were ACar (22:3), linoleic acid, DG (36:2), PC (34:2), TAG (48:2), SM (d32:1). All eight free fatty acids were significantly accumulated  18 proteins were identified as potential targets for MEHP, of these acyl-CoA dehydrogenase family member 9 (ACAD-9), enoyl-CoA hydratase 1 (ECHS1), HADH, and HSD17B10 were identified with high confidence. Six proteins were related to the fatty acid β-oxidation pathway, indicating that fatty acid degradation may play an important role in MEHP-induced lipid accumulation. | High confidence, very high relevance |
|  |  |  |  | Human, in vivo (epidemiology; subpopulation of NHANES study, n=6,005 women)) | Not concluded, but related positive associations of DEHP metabolites MBP and MEHP with BMI and waist circumference. |  | (Yaghjyan et al., 2015) | Women aged ≥18 years who were not pregnant and had no history of diabetes were included in this study; seven urinary phthalate metabolites were monitored and correlated with BMI, waist circumference, total cholesterol, triglycerides, HDL-C and LDL-C. All examined metabolites are metabolites of DEHP.  **BMI** was positively associated with monobutyl phthalate (MBP) and mono-2-ethylhexyl phthalate (MEHP) (odds ratio (OR) = 1.13; 95% confidence interval (CI), 1.03–1.23 and OR= 1.12; 95% CI, 1.03–1.23, respectively). **Waist circumference** was positively associated with MBP (OR = 1.13; 95% CI, 1.03–1.24). A higher ratio of MEHP to mono-(2-ethyl-5-hydroxyhexyl) phthalate (MEHP) was positively associated with both BMI (OR= 1.21; 95% CI, 1.09–1.34) and waist circumference (OR = 1.20; 95% CI, 1.10– 1.31). There were no other significant associations. |  |
|  |  |  |  | Human, in vivo (epidemiology. n=97 pregnant women) | (not concluded) | Oxidative stress | (Waits et al., 2020) | Urinary phthalate metabolites in the late third trimester were associated with oxidative/nitrosactive stress biomarkers | High confidence, but less relevant |
|  |  |  |  | Human, in vivo (epidemiology, cross-sectional study for occupational exposure in hairdressers, n=66 female, n=2 male. Control: n=32, 65.6% female/34.4% male) | (not concluded) |  | (Kolena et al., 2017) | Age range in apprentice hairdressers was 16-20 years (median: 17.7); the average age in the control group was 23.8 years.  Anthropometric measures collected from each study participant were body height and weight, waist and hip girth, BMI, waist-to-hip ratio, waist-to-height ratio, fat mass index and fat-free mass index; body weight, body fat percentage, muscle mass percentage and visceral fat level were estimated by bioelectrical impedance, a pulmonary function test was performed.  In first-morning-void urine samples detected DEHP metabolites of MEHHP, MEP, MnBP, and MiBP in all samples; MEOHP (97.06%) and MEHP (86.76%) were detected. MEHP had a level ratio of 12.37%; urinary concentrations were 10.23 (±29.37) ng/mL in the hairdresser and 5.63 (±5.56) ng/mL in the control group.  The study concluded on decreased pulmonary function scores correlated to body composition (especially waist-to-height/hip ratio and BMI); this was not necessarily chemical-related. | Moderate/low confidence and relevance.  Urine concentrations were not adjusted for dilution, e.g. by normalisation to creatinine. |
|  |  |  |  | Human, in vivo (epidemiology. n=102 male (51 normal weight, 51 overweight/obese; age 18-55 years); urinalysis and blood sample after 12 h fasting) | (not concluded) | Serum ALT and AST  Serum TG and HDL-c  Cross-references: thyroid hormone signalling and antiandrogen effect | (Milošević et al., 2018) | Only healthy adults were included in the study; participants diagnosed with liver disease, T2D, infection autoimmune/endocrine diseases, alcohol/drug use, or diagnosed malignancy were excluded.  DEHP metabolite monoethyl phthalate (MEP) was detected in 28.43% of volunteers (29/102; 10 normal weight 72.97±52.53 µg/g creatinine)/19 obese (146.43±214.05 µg/g creatinine)), MEHP in 20.59% of volunteers (21/102; 11 normal weight (140.05±92.24 µg/g creatinine)/10 obese (118.05±117.68 µg/g creatinine)). No statistically significant pattern was observed.  In normal-weight MEHP-positive subgroup: significant increment in BM. Urine MEHP was negatively correlated with serum HDL in normal weight cohort.  In obese group: linear correlation between MEP concentration in urine and TG serum levels, visceral adiposity index, lipid accumulation product, TG to HDL-ratio. In normal weight MEP-positive subgroup: significant increment in serum transaminase levels.  Phthalate levels in urine may be rather correlated with increase TG and decreased HDL cholesterol, indicative of cardiometabolic risk and insulin resistance, while phthalate exposure (MEP) may be related to increased serum ALT and AST. | High confidence, moderate relevance  Rather small cohort, but strict and targeted exclusion criteria  Noted limitations: no other environmental pollutants (e.g. BPA, PFHCs, PCBs) were analysed and may skew the results. |
|  |  |  |  | Systematic review and meta-analysis (**rodent**; n=31 included studies) | Not concluded, but positive association of early life exposure with increased fat pad weight, but not body weight |  | (Wassenaar and Legler, 2017) | Early life exposure to DEHP or MEHP was significantly associated with increased fat (pad) weight (mean difference 0.02, 95% CI: 0.00-0.03). The association with body weight was non-significant (mean difference: -0.14, 95% CI: -0.32-0.04). No meta-analysis could be conducted for other outcomes due to data scarcity (i.e. < 5 studies per outcome). | Very high confidence, moderate relevance |
|  |  |  |  | Review | Not concluded | PPARα | (Desvergne et al., 2009)  (Feige et al., 2007, Zoete et al., 2007) | Co-crystallisation of PPARγ with rosiglitazone or MEHP revealed similar binding patterns and interaction points with the PPARγ ligand binding domain. In contrast, the parent compound DEHP was neither able to enter the ligand binding pocket of PPARγ, nor to maintain stable contacts with the required protein moieties.  The DEHP metabolite MEHP, but not the parent compound, can activate PPARα as well as PPARγ. | High confidence and moderate relevance  (structural biochemistry discussion, no information on tissue biology/toxicology)  Pivotal study for mechanistic understanding |
|  |  |  |  | Human, in chemico/ in silico | Not concluded/assessed | **PPARγ agonism** | (Kratochvil et al., 2019) | Hydrogen/deuterium exchange mass spectrometry and docking, and surface plasmon resonance analysis of molecular binding to PPARγ were analysed for DEHP and its metabolites MEHP and MEOHP.  Binding to the ligand binding pocket of PPARγ was confirmed for the two metabolites MEHP and MEOHP, but not for the parent chemical DEHP. Upon binding of MEHP or MEOHP, conformational changes of the receptor resemble those observed with activation by the endogenous ligand 15-deoxy-delta-12,14-prostaglandin J2. This productive agonism was also confirmed by GeneBLAzer® PPARγ transactivation assay. For MEHP cytotoxicity IC_10_ of 203 µM and PPARγ activation EC_10_ of 1.2 µM were derived. | Very high confidence, moderate relevance |
|  |  |  |  | Fish, in vivo (AB strain zebrafish, WT and nrf2a*^fh318-/-^* mutant; embryolarval test, 200 µg/L MEHP, 6-96 hpf (histology, n=30 per group) or 6-120 hpf + 10 d depuration) | Yes. | Nrf2a (oxidative stress response)  *Fabp1a1* (via **PPARα**)  Gene expression unchanged on dfp 15 for: *pparaa, pparg, apoa1a, fabp1b1* (PPARγ responsive) | (Sant et al., 2021) | At 96 hpf, some animals showed vacuolisation in the liver; this was more pronounced upon exposure to MEHP.  At 15 dpf, Nrf2a-deficiency resulted in 7.7% growth (body length) reduction; MEHP exposure did not significantly affect growth. Further, MEHP exposure resulted in hepatic steatosis and increased expression of the PPARα target *fabp1a1*. Lipid accumulation in the liver was higher in mutant larvae, but this was not statistically different to WT. Additionally, lipid accumulation in the brain was increased upon MEHP exposure in WT larvae. |  |
| **Amiodarone** | 1951-25-3 | 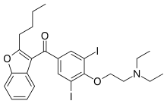 | Pharmaceutical; antiarrythmic drug | **SUMMARY** | **Positive**  **Strong induction of steatosis** | Metabolism predominantly via CYP3A4 and CYP2C8 (US FDA) |  | **Very strong weight of evidence** supporting induction of hepatic steatosis in human in vitro, including substantial complementary supportive mechanistic information.  Rodent ex vivo data do not conclude on steatosis induction by amiodarone, but mechanistic evidence reported is in line with molecular mechanisms observed in human cell lines in vitro. No human in vivo data were retrieved, but effects on side effect to the liver (steatosis/lipid accumulation not specified) are included in the label of marketed amiodarone tablets (US FDA).  **Amiodarone was selected as a reference chemical for hepatotoxicity as a disruptor of mitochondrial function and fatty acid metabolism, inducing steatosis in other EU funded projects: Seurat-1 and LIINTOP.** Molecular-level modes of action are (mitochondrial) membrane disruption, proton uncoupling and phospholipid binding, resulting in steatosis, phospholipidosis (by accumulation of phospholipids in lysosomes), and/or cytotoxicity. Intracellular triglyceride accumulation occurs through increased de novo lipogenesis and decreased β-oxidation/ mitochondrial respiration. |  |
|  |  |  |  | Human, in vitro (differentiated HepaRG, 96 h exposure, 5-25 µM) | Increase | DNL enzyme gene expression increased at 20 µM: ACLY (unaltered: ACACA, FASN, hSCD1) | (Allard et al., 2020) | Positive control chemical for steatosis  **LOEC** in differentiated HepaRG cells: Mitochondrial fatty acid oxidation decreased: 25 µM; de novo lipogenesis increased: 15 µM; apoB secretion into medium: not altered; neutral lipid accumulation increased: 15 µM. (steatosis induced also in primary human hepatocytes in vitro) | Very high confidence and relevance |
|  |  |  |  | Human, in vitro (differentiated HepaRG, 1 -14 d exposure, 5-20 µM) | Induction of steatosis after repeated dose exposure (phospholipidosis after single dose) | Gene expression significantly changed (in **bold**: at more than one concentration) after 24 h exposure: SLC27A4 (↑), PPARGC1A (↑), ACLY (↑), SCD1 (↑), SREBP1 (↑), **THRSP (↑),** LPIN1 (↑), LSS (↑), ADFP (↑), PLIN4 (↑), **CYP2E1 (↓),** CYP3A4 (↑) after 14 d exposure: SLC27A4 (↑), **PPARGC1A (↑),** FASN (↑),SREBP1 (↑), THRSP (↑), LPIN1 (↑), SOAT1 (↑), PLIN4 (↑), ASML3A (↑), **GDPD3 (↑),** **CYP2E1 (↓),** **CYP3A4 (↑**) | (Anthérieu et al., 2011) | Dose-dependent induction of triglycerides (TG) was observed after repeat exposure to amiodarone (LOEC = 10 µM); TG accumulation was proportional to the increased expression of lipogenic genes. Cholesterol esters were increased (LOEC = 5 µM; lowest concentration tested), but abundance of cholesterol esters was very low in control and remained very low in all treatment groups.  Fatty acid oxidation impaired both, after acute (24 h) and chronic (14 d) exposure.  Induction of phospholipidosis (especially of phosphatidyl ethanolamine and phosphatidylcholine) after short-term exposure (24 h) and vesicular steatosis after repeated exposure over 14 d.  Gene expression studies: acute 24-h exposure: 10 µM, 20 µM; chronic 14-d exposure: 5 µM, 10 µM, 20 µM | Very high confidence and relevance |
|  |  |  |  | Human, in vitro (HepG2 and differentiated HepaRG, pre-loaded for 14 h with 62 µM oleate palmitate (2:1); 6.25-50 µM, 24 h) | Induction, (up to ~150% in HepaRG at non-toxic concentrations) | Oxidative stress (ROS; significant at all concentrations (p<0.01)), decreased mitochondrial membrane potential (significant at all concentrations)  Gene expression changed: CROT, ANGPTL3, FOXA1, EHHADH, MTTP, IGFBP1, PGC1A | (Tolosa et al., 2016) | Exposure of cells to test chemicals was conducted in fatty-acid-free medium.  Amiodarone is included as a model steatogenic chemical (Drug-induced liver Injury category: “severe”), also causing mitochondrial impairment – which is possibly also a contributory mechanism to intracellular lipid accumulation.  In HepaRG: Significant lipid accumulation at all tested concentrations; cell viability was decreased at 50 µM.  Gene expression (25 µM) analysed, but unchanged: PPARA, SREBP1C | Very high confidence and relevance |
|  |  |  |  | Human, in vitro (HepG2, 72 h exposure, 0-100 µM) | Not concluded/ assessed | Significantly different differentially expressed proteins: heterogenous nuclear ribonucleoprotein A1 (HNRNPA1) ↑ (mRNA processing; stabilisation of CYP2A5 mRNA), AKR1C1 ↑ (xenobiotic metabolism)  In western blot: liver carboxyl esterase significantly increased, heterogenous nuclear ribonucleoprotein A1 increased (not significantly) | (van Summeren et al., 2011) | Cytotoxicity range finding derived the exposure concentration for proteomics: 7.5 µM (=IC_20_)  Aldo-keto reductase family 1 C1 (AKR1C1), a phase I metabolising enzyme, can indicate an excess amount of lipid peroxides. | High confidence and relevance.  HepG2 cells have a low expression of CYP enzymes; amiodarone is extensively metabolised through CYP3A4 into its toxic metabolite. This may lead to higher toxicity in metabolically more competent cell lines, or in vivo. |
|  |  |  |  | Human, in vitro (undifferentiated HepaRG, passage 22-25. 1-14 d exposure, 1-125 µM) | (not assessed) |  | (Limonciel et al., 2011) | Cytotoxicity assessed by intracellular ATP content (increased at 1 µM, diminished at ≥75 µM) and resazurin reduction (LOEC 10 µM).  Peak lactate concentrations in medium were reached on day 3 after exposure to ≥75 µM | Lower relevance towards steatosis, deviating culture medium used for HepaRG cultivation, HepaRG cells were not differentiated |
|  |  |  |  | Human, in vitro (differentiated HepaRG cells, 72 h, 1.5×10^-9^ - 1×10^-4^ M) | (not concluded) |  | (Landesmann et al., 2012) | ROS induction and decrease in cell count and nuclear staining intensity in differentiated HepaRG (72 h exposure; at approx. 0.1 µM)  ROS induction is used as an integrative proxy for known mechanisms of hepatotoxicity, including mitochondrial damage, inflammation/ immune reactions, and metabolism. |  |
|  |  |  |  | Human, in vitro (undifferentiated HepaRG in 2D or 3D spheroid culture; exposure after 7 d pre-growth for 1-7 d; 9-step two-fold dilution from 162 µM) | Not assessed |  | (Ott et al., 2017) | High-throughput HepaRG CYP induction assay. CYP3A4 activity was assessed by P450-Glo Luciferin-IPA assay (Promega), CYP1A activity by EROD assay, cytotoxicity as ATP content by CTG assay (Promega).  Therapeutic maximum concentration in vivo: 5.3 µM.  Cytotoxicity: IC50 in 2D: >162 µM (24 h), >162 µM (7 d) IC50 in 3D: 83.3 µM (24 h), 48.6 µM (7 d)  Interlaboratory IC50 comparison: 178 µM (24 h, 3D HepaRG), 83.3 µM (24 h, 3D HepaRG), 48.6 µM (7 d, 3D HepaRG), 6.5 µM (7 d, primary human hepatocytes) | High confidence, moderate relevance (high for cytotoxicity)  The authors declare a conflict of interest for the micromolds used to produce the HepaRG 3D spheroids |
|  |  |  |  | Review | Induction of steatosis | HIF-1α, PPAR α  Phospholipase inhibitor. Carnitine palmitoyltransferase I inhibition, inhibition of fatty acid secretion via microsomal triglyceride transfer protein | (Jennings et al., 2014) | **Amiodarone was selected as a reference chemical for hepatotoxicity as a disruptor of mitochondrial function and fatty acid metabolism.** Molecular-level modes of action are (mitochondrial) membrane disruption, proton uncoupling and phospholipid binding, resulting in steatosis, **phospholipidosis**, and/or cytotoxicity.  Disruption of mitochondrial fatty acid oxidation diverts energy metabolism towards glycolysis with subsequent lactate production (potentially linked to HIF-1α pathway activation)  Amiodarone is initially drawn into mitochondria by the proton gradient, and later the accumulated drug inhibits complexes I, II, and V of the electron transport chain.  **Induction of PPARα-related genes may be a result of steatotic processes and a compensatory rather than a causative effect.**  Further data for this reference chemical are available online: <https://wiki.toxbank.net/wiki/Amiodarone> (incl. curated information on toxicokinetics, genomics, proteomics, physico-chemical properties) | High confidence, chemical selection report for SEURAT-1 project, accepted by relevant stakeholders, including co-authors from EFSA, EURL ECVAM/JRC, academia, industry and national public health authorities |
|  |  |  |  | Rodent, ex vivo (isolated mouse liver mitochondria; 200 µM) | Not concluded |  | (Fromenty et al., 1990) | Title: “Dual effect of amiodarone on mitochondrial respiration. Initial protonophoric uncoupling effect followed by inhibition of the respiratory chain at the levels of complex I and complex II”  Amiodarone accumulated in liver mitochondria, leading to collapse of the membrane potential.  Pre-incubation of mitochondria with 150-200 µM amiodarone decreased mitochondrial respiration mediated by fatty acids (β-oxidation) and citrate.  Concluding, amiodarone has a dual effect: initially uncoupling, followed by inhibition of respiratory chain complexes I and II by accumulated amiodarone, resulting in decreased respiration/ATP production. |  |
|  |  |  |  | Rodent, ex vivo (24-week-old C57BL/5 mice, n = ≥4/group, precision-cut liver slices, 24 h incubation, 0-100 µM (50 µM for gene expression profiling)) | Not concluded, but indication of steatogenic potential (lipid metabolism upregulated) | PPARγ agonism  Gene expression altered: Abcd3, Acat1, Ehhadh, Ly6d, Lpcat3, Pex1, Pck2, Fgf21 | (Szalowska et al., 2014) | 5 mm-diameter cylindrical liver cores were obtained by biopsy; after processing, slices were 0.2 mm thick and weighted ~6 mg per slice.  Genes significantly upregulated (fold-change ≥1.5) in GSEA and proposed as biomarkers for amiodarone/valproate-like acting chemicals: Abcd3, Acat1, Ehhadh, Ly6d, Lpcat3, Pex1, Pck2, Fgf21  While amiodarone and valproate induced gene expression changes clustered together, tetracycline induced a distinctly different pattern. Main differences were found in gene sets related to lipid metabolism, fatty liver, and peroxisomes, which were upregulated by both AMI and VA, and downregulated by TET.  AMI and VA downregulated several GO annotation clusters affiliated to immune functions, extracellular matrix, and development  In contrast to AMI and VA, TET downregulated functional clusters related to lipid synthesis, β-oxidation, *Pparα* signalling, inflammation, apoptosis, and other clusters related to energy and bile acid homeostasis. Functional clustering identified clusters such as lipid synthesis, β-oxidation, mitochondria, peroxisomes, and PPARα -dependent lipid metabolism. | High confidence, moderate relevance |
| **Docosahexaenoic acid (DHA)** | 6217-54-5 | 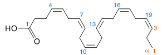 | omega-3 fatty acid | **SUMMARY** | **Negative/ reduction** | PPARα |  | **Strong weight of evidence** supports DHA to **not induce hepatic steatosis**; some evidence (including human in vivo dietary supplementation studies) suggests that DHA may contribute to decreased hepatic lipid content.  A limitation in reviewing the literature was found to be that most studies (especially dietary supplementation studies) investigated effects of several poly-unsaturated fatty acids (PUFAs) and lacked a DHA-only treatment group, but composition of the PUFA dietary supplement used in clinical trials was often well-characterised, particularly for the content of DHA and eicosapentaenoic acid (EPA). |  |
|  |  |  |  | Human, in vitro (HepG2 cells pre-loaded with palmitic acid and treated with DHA 50 µM, 24 h)  Rodent, in vivo (C57BL/6, male mice, n = 10/group on HFD; administered with DHA (100 mg/kg) by gavage twice a week for 8 weeks) | Reduced lipid accumulation.  DHA counteracted lipid accumulation in mice and cells. | Sirt1 | (Luo et al., 2020) | In the mice, DHA counteracted the following hepatic changes induced by HFD: ↑ lipid accumulation, ↓ mRNA for Pgc1α, IL10, AdipoQ, ↑ mRNA for Acadm, IL6, Tnfα, Mcp1; DHA also increased mRNA levels of Mttp and Apob100. In the cells, DHA counteracted the following palmitic acid induced changes: ↑ lipid accumulation, ↓ Sirt1 protein, ↑ acetylated-p65 protein, ↓ mRNA of Pgc1α and Mttp; DHA also ↓ Acc mRNA and ↑ Apob100 mRNA. Sirt1 knockdown counteracted most of DHA’s effects in the cells and mice. |  |
|  |  |  |  | Human, in vitro (Hep3B, Huh-7, and HepG2 cells. 12-72 h, 5-60 µM) | Not concluded | Wnt/β-catenin signalling, COX-2 | (Lim et al., 2009) | Study of the effects of DHA, EPA, or arachidonic acid (AA) on the growth and viability of human hepatocellular carcinoma cell lines, including Hep3B, Huh-7, and HepG2 cells. Contains no results on lipid accumulation.  For 24 h DHA exposure, cell viability was decreased (<~80%) at ≥10 µM (Hep3B), ≥10 µM (Huh7), and ≥60 µM (HepG2). |  |
|  |  |  |  | Rodent, in vivo (C57BL/6J, male Ldlr^-/-^ mice (“western diet NASH model), n = 8/group; administered a DHA product in the diet for 16 weeks at a 2% total energy level.) | Not induced; uncertain due to impurities in DHA product |  | (Depner et al., 2013a) | The DHA product used for dosing was impure and contained only 39% DHA acyl chains together with several other fatty acids including, 12:0 (5.7%), 14:0 (15%), 16:0 (8.9%), 16:1 (n-7), (3.5%), 18:1 (n-9) (27%), and 18:2(n-6) (1.2%) | Low confidence due to impurities in DHA used, and uncertainty if findings can be attributed to DHA |
|  |  |  |  | Review on the effects of omega-3 polyunsaturated fatty acid (n-3 PUFAs) on NAFLD. |  |  | (Di Minno et al., 2012) | Most of the information provided is for PUFAs and not specifically for DHA. In one clinical study in which DHA (250 or 500 mg/day) treatment was evaluated in children with NAFLD (n = 20/group) for 6 months, DHA treatment was associated with lower odds of severe steatosis. |  |
|  |  |  |  | Human, in vivo (observational study that evaluated the plasma lipodomic profiles in healthy volunteers (n = 50), and in patients with nonalcoholic fatty liver disease (n = 25) or NASH (n = 50)) | Not concluded/assessed |  | (Puri et al., 2009) | Analysis and comparison of plasma lipids and eicosanoid metabolites; patients presented with non-alcoholic fatty liver (n=25), NASH (n=50), or lean normal control subjects (n=50).  No hepatic lipid measurements were included in the study. Plasma levels of DHA was decreased in NASH but not nonalcoholic fatty liver patients. The DHA to docosapentaenoic acid ratio was decreased within phosphatidylcholine and phosphatidylethanolamine pools in plasma from nonalcoholic fatty liver and NASH patients. | Low relevance. No DHA intervention, but fatty acid ratio in plasma studied |
|  |  |  |  | Human, in vivo (observational study that evaluated the fatty acid frofile in liver tissue samples from control subjects without NAFLD (n = 18) and in patients with simple steatosis (n = 63) and NASH (n = 40)) | Not assessed.  DHA content in liver tissue was increased in steatosis and NASH | mRNA levels of the following genes were increased in NASH compared to simple steatosis tissue: SCD1, ELOVL6, SREBP1, FAS, and PPARγ. Increased steatosis scores were associated with decreased C18:0/C16:0 ratios, and increased C18:1n9/C18:0 ratios. | (Yamada et al., 2015) | Insulin resistance was increased in NASH but not simple steatosis patients versus control. Increased hepatic inflammation scores were associated with increased SCD1 mRNA levels, decreased C18:0/C16:0 fatty acid ratios, and increased C16:1n7/C16:0 ratios. Increased hepatic ballooning scores were associated with increased SCD1, ELOVL6, SREBP-1c, FAS, ACC, and PPARγ mRNA levels, decreased C18:0/C16:0 ratio, and increased C16:1n7/C16:0 ratios. Increased hepatic fibrosis scores were associated with increased SREBP-1c mRNA levels, decreased C18:0/C16:0 ratio and increased C16:1n7/C16:0 ratio.  The levels of several different types of fatty acids, including DHA, were increased in liver tissue from simple steatosis and NASH patients compared to controls. | Low relevance. No DHA intervention |
|  |  |  |  | Human, in vivo (observational cross-sectional study on the associations between omega-3 PUFA levels in erythrocyte membranes and fatty liver index in older people (n = 620)) | Not concluded |  | (Rose et al., 2016) | Fatty liver index was calculated based on body mass index (BMI), waist circumference, and plasma triglyceride (TG) and gamma glutamyl transferase (GGT) levels.  No direct hepatic lipid measurements were included in the study. In females and not in males, erythrocyte membrane DHA levels were slightly lower in individuals with a fatty liver index score indicative of NAFLD. | Low relevance. No DHA intervention |
|  |  |  |  | Human, in vivo (clinical study, n=33, 4 g/d DHA ethyl ester for 4 weeks) | Not concluded |  | (Park and Harris, 2003) | Clinical study on the effects of omega-3 fatty acid supplementation (incl. DHA or EPA) on plasma levels of triglycerides and chylomicrons, in order to clarify if reduced postprandial triglyceride content uponOmega-3 fatty acid supplementation is a result of reduced production or increased clearance of chylomicrons. Results for DHA and EPA were similar and pulled together after concluding the study.  Dosing regime: 4-week olive oil placebo (4 g/d) run-in, 4-week wash-out period, 4 week test-period (4 g/d of DHA, EPA, safflower oil, or placebo).  DHA supplementation reduced postprandial triglyceride (p=0.08, not significant) plasma apolipoprotein B48 (p<0.001) and 100 (p<0.01) levels. In the fed state, DHA reduced chylomicron triglyceride half-lives and chylomicron particle size, and increased pre-heparin lipoprotein lipase. | Did not include any hepatic lipid measurements. |
|  |  |  | Chenodeoxycholic acid (CDCA), DHA and eicosapentanoic acid (EPA) | Human, in vitro (HepG2 cells, CDCA with/without 5 µM DHA + 5 µM DHA, 0.5 h) | Not assessed | Increased PPARα activation, and increased PPARα and LXRα mRNA | (Tillman et al., 2016) | Study evaluated the effects of combination treatment with EPA + DHA on bile acid induced apoptosis in HepG2 cells. Did not evaluate DHA alone and included no steatosis endpoints. DHA + EPA.  (EPA+DHA-induced) PPARα transcriptional activity was decreased by CDCA. |  |
|  |  |  | DHA metabolite, 17-hydroxy-docosahexaenoic acid (17-HDHA | Rodent, in vivo (C57BL/6NHsd, male, n = 6/group; administered intraperitoneally (i.p.) for 14 days (dose not stated)) | No induction on HFD | Protein levels in liver:  ↑ PPARα/γ, ↓ precursor and cleaved SREBP1, ↓ NFκB. | (Rodriguez-Echevarria et al., 2018) | High-fat, fructose-enriched diet mouse model of NAFLD  Histological hepatic steatosis, ballooning, and fibrosis scores were not significantly different between HFD and HFD + 17-HDHA groups. 17-HDHA, however, reduced hepatic inflammation scores. | In many of the figures of the paper, it appears that 17-HDHA had been mislabeled as 17-HEPE. All of the 17-HEPE labels in the figures were taken to be 17-HDHA. |
|  |  |  | EPA+DHA | Rodent, in vivo (mouse) | Not concluded |  | (Wolstenholme et al., 2018) | Mouse study on the effects of diet supplementation with menhaden oil (containing ω-3 fatty acids including EPA and DHA) and/or safflower oil (containing ω-6 fatty acids) on ethanol consumption, ethanol effects, and body fat composition (liver fat results are not presented). Contains no steatosis relevant data. | Low relevance. No exposure to pure DHA |
|  |  |  | EPA + DHA ethyl esters | Human, in vivo (clinical study; n=38 healthy men. 4 g/day omega 3 supplement for 8 weeks) | Decreased intracellular triacyl glyceride content |  | (Green et al., 2020) | Clinical study on the effects of combination treatment with DHA + EPA on intrahepatocellular triglyceride content and hepatic de novo lipogenesis and fatty acid oxidation in healthy men. The effect of DHA + EPA was also evaluated in Huh7 cells.  Decreased fasting plasma triacyl glyceride content, after eight weeks of supplementation; intrahepatic triacyl glycerides, fasting and postprandial hepatic de novo lipogenesis were significantly decreased; dietary fatty acid oxidation, fasting and postprandial plasma glucose concentrations were increased.  In vivo findings were validated in vitro (Huh7 cells), confirming the intracellular decrease in triacylglycerols and proposing this might be due to a metabolic shift from fatty acid esterification to fatty acid oxidation. | High confidence, moderate relevance.  Did not include a DHA alone group. |
|  |  |  | DHA + EPA | Human, in vivo (clinical study; n=37 patients with well-controlled diabetes; PUFA supplement (2160 mg EPA + 1440 mg DHA) daily for 48 weeks | No effect on steatosis |  | (Dasarathy et al., 2015) | Clinical study evaluating the effects of combination treatment with DHA + EPA on NASH.  At the end of treatment, hepatic steatosis and the activity score improved (p<0.05) and lobular inflammation worsened (p<0.001) with placebo but was unchanged with PUFA. At the end of treatment, insulin resistance (serum glucose and HOMA) worsened with PUFA but not placebo. | DHA alone treatment was not evaluated. |
|  |  |  | DHA + EPA | Human, in vivo (clinical study, n=78 NASH patients; 50 mL PUFA (1:1 EPA+DHA) daily for 6 months) | Decreased steatosis in NASH patients |  | (Li et al., 2015b) | Clinical study on the effects of polyunsaturated fatty acid (PUFA) treatment in NASH patients. The study did not include a DHA alone treatment, only a PUFA treatment containing DHA and EPA.  Significantly improved: serum liver enzyme activity (ALT, AST), serum levels of triglycerides, total cholesterol, C-reactive protein, malondialdehyde (as a proxy for fatty acid oxidation), and fibrosis marker (type IV collagen, pro-collagen type III pro-peptide). Histologically, improved grades of steatosis, necroinflammation, fibrosis, and ballooning were all profoundly improved. | Did not include a DHA alone treatment group. |
|  |  |  | DHA + EPA | Human, in vivo (clinical study, n=78 overweight or obese individuals with NAFLD and hypertriglyceridemia. 4 g PUFA for 12 weeks) | No effect on steatosis; decrease of serum triglycerides |  | (Oscarsson et al., 2018) | Clinical study on the effects of an omega-3 carboxylic acid supplement (4 g/day; containing DHA, EPA, and other fatty acids; n=25), 200 mg/day fenofibrate (n=27), or placebo (n=26) treatment in NAFLD.  Upon conclusion of the study, liver proton density fat fraction was not significantly different between treatment groups. Serum triglycerides were decreased with PUFA, other parameters were unchanged compared to placebo (including fat volume of liver, pancreas, and adipose tissue) | Did not include a DHA alone treatment group. |
|  |  |  | DHA + EPA | Human, in vivo (open-label single arm intervention pilot, n=12. 6 g/d fish oil (containing 1.9 g EPA and 1.5 g DHA) for 6 weeks) | Not concluded |  | (Nording et al., 2013) | Clinical study characterising plasma total lipidomic and oxylipin profiles, which were significantly altered by the intervention.  Very high interindividual variability in effects of omega 3 supplementation | Contains no results for DHA alone.  Small sample size |
|  |  |  | DHA + EPA | Human, in vivo (clinical study in NAFLD patients) |  |  | (Manousopoulou et al., 2019) | Clinical study on the plasma proteomic effects | Low relevance. Did not include a DHA alone group, and did not include any steatosis endpoints. |
|  |  |  | DHA + EPA | Human, in vivo (clinical study) |  |  | (Kuttner et al., 2019) | Effects of PUFA on hepatic steatosis in carriers and non-carriers of the PNPLA3 p.148M genetic variant, which is a strong genetic determinant of NAFLD. | Did not include a DHA alone treatment group. |
| **Resveratrol** | 501-36-0 | 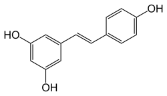 | Natural phenol, stilbenoid, phytoalexin; dietary supplement | **SUMMARY** | **Negative/ decrease** | SIRT1, LXRα |  | **Strong weight of evidence supports that resveratrol does not induce hepatic steatosis.** This is consistent in vitro (human and murine cell lines), and in vivo (human clinical trials, meta-analyses of clinical trials, and in rodents).  In most experimental model studies in cells and rodents, resveratrol had no effect on hepatic lipids or decreased hepatic lipid content. However, the decrease in hepatic lipid content could not be confidently replicated in human in vivo clinical studies (in patients with NAFLD), where resveratrol had no effect on hepatic lipid levels but did not lead to a significant decrease.  Mechanistically, resveratrol is a SIRT1 agonist, and its activity in lowering intracellular accumulation (especially in vitro) is largely accounted to this activity. |  |
|  |  |  |  | Human, in vitro (HepG2 pre-loaded with palmitate; 1-50 µM resveratrol, 20 min – 24 h) | Decreased triglyceride accumulation | ↑ phospho-STAT3, ↓ FAS mRNA, ↓ Atf4 mRNA. | (Ardid-ruiz et al., 2019) |  |  |
|  |  |  |  | Human, in vitro (HepG2; 10 µM resveratrol for 2 h followed by 1.5 mM oleic acid for 24 h) | Decreased lipid and intracellular triglyceride levels | ↑ mitochondrial membrane potential, ↑ **SIRT1** activity, ↑ phospho-AMPKα Thr172, ↓ ATP, mRNA changes (↑ CPT1A1, ↓ FAS, ↑ PPARγ, ↑ SIRT1). | (Rafiei et al., 2019) |  |  |
|  |  |  |  | Human, in vitro (HepG2; 10-30 µM resveratrol, 2-48 h). | Not assessed | Resveratrol mRNA effects: ↑ UGT1A1+2B7, ↑ ST1E1. | (Lançon et al., 2007) | No lipid measurements were included. |  |
|  |  |  |  | Human, in vitro (HepG2, 10-100 µM, 24 h) | Not assessed (reduction/ no effect on steatosis inferred from reduced ER stress) | ↑ SIRT1 leads to ↓ ER stress | (Lee et al., 2019b) | ER stress induced by thapsigargin (0.5-2 µM) and/or tunicamycin (0.5-5 mg/mL); for ER stress induction prior to resveratrol treatment (100 µM, 24 h): 3 µg/mL tunicamycin for 24 h.  Resveratrol as a model SIRT1 agonist; EX-527 (10-30 µM) used as SIRT1 antagonist. | No results on lipid accumulation |
|  |  |  |  | Human, in vitro (HepG2 glucose-induced (25-33 mM) steatosis model, 10-20 µM resveratrol, 24 h) | Reduction/ no induction |  | (Izdebska et al., 2018) | High-glucose induces microvacuolar steatosis, which is prevented by co-exposure to resveratrol; no cytotoxicity was observed with resveratrol.  Lipid and other staining results are presented in the paper are not quantified. | Low confidence (no quantification of fluorescence, poor quality morphology photos presented) |
|  |  |  |  | Rodent, in vitro (RAW264.7 murine macrophage cell line, 65 µg/mL oleate vs. 65 µg/mL oleate + 1.5 µg/mL resveratrol; 24 h) | Not concluded, but prevention of oleate-induced intracellular lipid accumulation in murine macrophages | Gene expression: Oleate-only: Cd36, Fatp1, **Fatp2**, **Dgat1**, **Cpt1a**, and **Pnpla3** were significantly upregulated, while those of **Scara1**, **Fabp4**, **Fabp5**, and **Srebp1c** were significantly downregulated. [in bold: reversed by resveratrol co-exposure)  Resveratrol co-treatment: Cd36, Lipa, and Lpl were significantly increased and those of Fabp1 and Fatp1 were significantly decreased | (Ye et al., 2019) | Publication on the metabolomic effects of oleate treatment with and without resveratrol in macrophages.  Resveratrol co-exposure prevented accumulation of neutral lipids (by Nile Red staining).  “Most FFAs, such as palmitic acid, palmitoleic acid, stearic acid, linoleic acid, and eicosanoic acid, were all significantly decreased in oleate-treated macrophages. In contrast, oleic acid, 11,14-eicosadienoic acid and 5,8,11-eicosatrienoic acid were significantly increased in oleate-treated macrophages, and these effects were attenuated or abolished by RSV. Notably, glycerol, glycerol 3-phosphate, 1-monopalmitin, 2-monopalmitin, 1-monooleoylglycerol, and 2-monooleoylglycerol significantly accumulated in oleate-treated macrophages, but these accumulation events were alleviated by RSV.”  Oleate induces disturbances in glycerolipid metabolism and then leads to triglyceride accumulation in macrophages.  Gene expression data indicate disordered lipid transport, increased FFA import into mitochondria for oxidation, and enhanced TG synthesis and lipolysis during neutral lipid accumulation in oleate-treated macrophages. mRNA expression levels of Cd36, Lipa, and Lpl were significantly increased and those of Fabp1 and Fatp1 were significantly decreased in macrophages cotreated with oleate and RSV | Moderate relevance (no effects in hepatocytes) |
|  |  |  |  | Rodent, in vivo (Wistar, male, n = 10-20/group, on HFD) orally dosed with resveratrol (100 mg/kg/day) for 10 weeks.  Human, in vitro (HepG2 steatosis model (high glucose + insulin induced) 50 µM resveratrol, 24 h) | Decreased hepatic lipid and triglyceride content | Hepatocyte effects of resveratrol: ↑ phospho-AMPK, altered mRNA (↓ SREBP-1c, ↓ FAS). | (Shang et al., 2008) | In the HFD rats, resveratrol decreased hepatic lipid and triglyceride content. This was confirmed in the HepG2 cell steatosis model: resveratrol decreased intracellular triglyceride content. |  |
|  |  |  |  | Rodent, in vivo (Wistar rat NAFLD model (elderly male on HFD), n = 7/group; 25 mg/kg bw/d, i.p. for 8 weeks) | Not assessed/concluded | In liver: ↓ apoptotic cells, gene expression increased: LXR, FXR, SIRT1 | (Hajighasem et al., 2018) | Mean age of rats at beginning of the study: 40-50 weeks. In HFD group, HFD was fed for 6 weeks prior to initiation of experiments;  Included no hepatic lipid measurements. Resveratrol reduced levels of AST, ALT, and ALP in liver tissue.  Resveratrol alone improved biochemical markers and liver function, yet this was exacerbated in combination with physical exercise. |  |
|  |  |  |  | Rodent, in vivo (C57BL/6J mice, male on HFD, n = 3-10/group; 200 mg/kg bw/d resveratrol, 8 weeks, oral via diet)  Human, in vitro (HepG2 cells, 25-100 µM resveratrol, 3-48 h) | Decreased liver weight and cholesterol content (triglycerides/ neutral lipids not assessed) | ↑ CYP7A1 mRNA/protein/activity.  In HepG2 ↑ CYP7A1 mRNA/protein (effect inhibited by the LXRα inhibitor, GGPP), ↑ **LXRα** transcriptional activity | (Chen et al., 2012) | Resveratrol decreased liver weight and cholesterol content. Hepatic neutral lipid and triglyceride content was not measured in the study. |  |
|  |  |  |  | Rodent, in vivo ( C57BL/6J mice, ethanol-induced steatosis model, male, n = 4-8/group; 200-400 mg/kg bw/d resveratrol, 2 weeks, oral via diet) | Decreased liver lipid and triglyceride levels, and plasma ALT levels | altered protein (↓ PGC-1α acetylation, ↑ **SIRT**, ↑ p-AMPKα+β, ↑ AMPKα+β, ↑ p-ACC, ↓ SREBP-1c), altered mRNA (↑ SIRT, ↓ SCD1, ↓ FAS, ↓ GPAT1, ↓ ACCα, ↓ ME, ↑ PGC-1α, ↑ AOX, ↑ MCAD, ↑ CPT1a, ↓ PPARγ, ↑ adipoR1+R2). Also increased plasma adiponectin levels. | (Ajmo et al., 2008) |  |  |
|  |  |  |  | Rodent, in vivo (C57BL/6J mice, male, n = 5/group; 2-20 mg/kg bw/d resveratrol, 2-4 weeks, oral via diet)  Rodent, in vitro (RAW264.7 murine monocyte/ macrophage cell-line, 5-50 µM resveratrol, 6 h) | No effect on steatosis/lipid accummulation | In the HFD model, resveratrol decreased hepatic CD14 mRNA. In the HFD + LPS model, resveratrol hepatic mRNA changes incl.: ↓ TNF-α, ↓ IL-6, ↓ CD14, and other hepatic effects incl: ↓ p-STAT3 protein, ↓ F4/80+ cells (Kupffer cells), ↓ CD14+ cells (activated Kupffer cells), ↓ CD14:F4/80 ratio, ↓ p-STAT3+ cells, ↓ p-STAT3:F4/80 ratio. | (Kessoku et al., 2016) | Experimental mouse model of NASH: mice were fed either a HFD diet (for 12 weeks prior to experiments), or a HFD diet with lipopolysaccharide (0.25 mg/kg bw/d) to induce inflammation (the administered lipopolysaccharide is considered a low dose).  In the HFD NAFL model, resveratrol had no effect on hepatic lipid or triglyceride content. In the HFD + LPS model, resveratrol reduced hepatic scores for fibrosis, lobular inflammation, and hepatocyte ballooning, but had no effect on steatosis. |  |
|  |  |  |  | Rodent , in vivo. (C57BL/6 male mice on high-fat diet intragastrically administered with resveratrol (400 mg/kg bw/d) for 30 days.  Human/rodent, in vitro (HepG2 cells and primary mouse hepatocytes treated with palmitic acid + resveratrol (10-40 µM, 24h)) | Reduction | In murine hepatocytes incl.: mRNA/protein changes (↑ SIRT1, ↓ PLIN, ↓ ATF6, ↓ CREBH, ↓ Fsp27β, ↓ CIDEC), ↑ ATF6 + SIRT1 interaction, ↑ **SIRT1** transcription via ATF6 inhibition, ↑ ATF6 deacetylation via SIRT1 | (Zhou et al., 2018) | In the HFD fed mice, resveratrol reduced hepatic triglyceride, lipids, lipid droplet counts/diameter/volume, total cholesterol, and LDL cholesterol, and increased HDL cholesterol. In palmitic acid treated HepG2 cells and mouse hepatocytes, resveratrol reduced lipids, triglyceride content, lipid droplet counts/diameter/volume.  SIRT1 knockdown/overexpression ↓/↑ resveratrol’s effects, respectively. ATF6 knockdown/overexpression ↑/↓ resveratrol’s effects, respectively. |  |
|  |  |  |  | Rodent, in vivo (obese OLETF rats, n = 6/group; diet supplemented with 0.5% resveratrol for 4 weeks) | No effect on hepatic triglyceride or cholesterol levels |  | (Nagao et al., 2013) |  |  |
|  |  |  |  | Rodent, in vivo (C57BL/6J mice, ethanol-induced steatosis model, male, n=8/group; 100 mg/kg bw/d resveratrol, oral gavage, 28 d) | Resveratrol reduced hepatic lipid content | Decreased ROS levels. | (Chen et al., 2016) | Hepatic lipid content reduction based on only a qualitative evaluation of lipid staining data. |  |
|  |  |  |  | Review on the effects of resveratrol and other polyphenols on hepatic steatosis | Decreased lipid content in vitro and in vivo (rodents and human) | Gene expression changes: ↓ SREBP1c, ↓ FAS, ↑ **SIRT1**, ↓ SCD, ↓ ACC, ↓ ME, ↑ PGC-1α, ↑ ACO, ↑ CPT-1a. Also had the following effects: ↓ ACC activity, ↑ phospho-AMPK, ↑ SIRT1, ↓ PGC-1α acetylation, ↑ mitochondrial number, ↓ oxidative stress, ↓ lipid peroxidation, ↓ lipogenic enzyme expression, ↓ necroinflammation, ↑ fatty acid oxidation enzymes (incl. FAS, G6PDH, ME), ↑ fatty acid oxidation, ↓ PAP, ↑ UCP2. | (Aguirre et al., 2014) | In 4 different publications, resveratrol (10-50 µM, 30 min or 24h) decreased triglyceride or lipids in HepG2 cells or rat hepatocytes. In 12 in vivo studies (0.5-450 mg/kg/day doses, 4-10 week treatment) in rodents, resveratrol decreased hepatic lipid levels. In one clinical trial in healthy obese male volunteers, resveratrol (150 mg/day for 1 month) decreased intrahepatic lipid content | High relevance |
|  |  |  |  | Review of clinical findings on the effects of dietary supplements (incl. resveratrol) on liver fat. | No effect on liver fat content |  | (Kilchoer et al., 2020) | Results of a meta-analysis of findings from 4 clinical trials showed resveratrol to have no effect on liver fat. |  |
|  |  |  |  | Meta-analysis of placebo-controlled clinical trials (n=4) on efficacy of resveratrol supplementation against NAFLD | No effect on liver biochemistry, in NAFLD patients (hepatic lipid content not assessed) |  | (Zhang et al., 2016) | Data evaluated include weight, BMI, blood pressure, plasma biochemistry (inc. liver enzymes, bilirubin, TNF-α, cholesterol, HOMA-IR).  “Although the present study did find some positive effects of resveratrol on metabolic parameters, the improvement in liver function and fatty liver was less apparent than expected.”  Resveratrol supplementation did not alter TNFα or bilirubin levels. Anthropometric and clinical parameters (body-Mass index, systolic/diastolic blood pressure) were unchanged by resveratrol treatment. No effect on liver enzyme activity (AST, ALT, and GGT). However, cholesterol and low-density lipoprotein plasma levels were significantly elevated after resveratrol intervention. | High relevance, moderate confidence (very thorough methodology, but no results on the effects of resveratrol on hepatic fat content is provided/ considered) |
|  |  |  |  | Meta-analysis of findings from randomized clinical intervention studies (n=7) on the effects of resveratrol in NAFLD | Reduction/no effect |  | (Jakubczyk et al., 2020) | In the discussion text it is mentioned that resveratrol improved measures of steatosis in 1 study, but had no effect on steatosis in 3 other cited studies.  Included studies report supplementation with 500-3000 mg/d resveratrol for 56-180 d.  Of the analysed parameters, only serum alanine aminotransferase activity was significantly (p=0.041) improved after supplementation, the rest remained unchanged.  This study includes studies evaluated earlier (Zhang et al., 2016). | High relevance, moderate confidence (very thorough methodology, but no results on the effects of resveratrol on hepatic fat content is provided/ considered) |
|  |  |  |  | Human, in vivo (clinical study in NAFLD patients (n = 10/group); 3000 mg/d resveratrol for 8 weeks) | No effect |  | (Chachay et al., 2014) | Resveratrol had no effect on hepatic triglyceride content, and increased plasma ALT and AST levels. |  |
|  |  |  |  | Human, in vivo (randomized, double-blind, placebo-controlled clinical trial in non-obese women with normal glucose tolerance (n = 15/group); 75 mg/d resveratrol for 12 weeks) | No effect on intrahepatic triglyceride content |  | (Yoshino et al., 2012) | Metabolic parameters incl. intrahepatic triglyceride content were studied; resveratrol had no effect on intrahepatic triglyceride content. |  |
|  |  |  |  | Human, in vivo (randomized, placebo-controlled, double-blind, parallel group clinical trial in middle aged men with metabolic syndrome (n = 21-24/group); 75 or 500 mg resveratrol, twice daily for 16 weeks) | No effect on intrahepatic lipid levels |  | (Kjær et al., 2017) | Various metabolic parameters, including hepatic lipid content were studied. |  |
|  |  |  | No chemical intervention | Rodent, in vivo (SIRT liver-specific KO mice (CD1 background), and WT mice, n=10/group (sex not specified)) | not assessed for resveratrol  SIRT1 liver-specific knock-out increased hepatic lipid accumulation | **SIRT1** and FGF21 signaling | (Li et al., 2014) | Most of the results in the paper describe the effects of liver specific SIRT1 KO in mice on fatty liver; fed vs. 24-h-fasted, and re-fed (24 h fasting, 6-h *ad-libitum* feeding).  SIRT1 and FGF21 both are regulated physiologically by a fasting-feeding-refeeding cycle; SIRT1 is a major regulator of fasting-inducible FGF21. FGF21 was increased 8-fold in fasted WT mouse plasma, being a **potential biomarker**.  It is noted, that SIRT1 is regulated by diet differently in the liver and other tissues, including white adipose tissue and the muscle.  In vitro (HepG2) data are referenced: **resveratrol** (10 µM, 24 h) decreased lipid accumulation, while also being a **model inducer of SIRT1 signaling**. | High confidence, low relevance. No resveratrol intervention  Mechanistic support for SIRT1-signalling in steatosis |
| **Rotenone** | 83-79-4 | 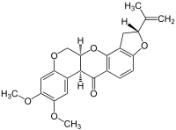 | Isoflavone; insecticide, piscicide, pesticide; naturally occurring (in *Fabaceae* plants) | **SUMMARY** | **Negative** for steatosis/lipid accumulation, but hepatotoxic | Inhibition of mitochondrial complex I, apoptosis, oxidative stress. |  | **Strong weight of evidence supporting rotenone does not induce primary hepatic steatosis at non-cytotoxic concentrations.** However, rotenone is classified as hepatotoxic; in vitro LOEC for cell viability is e.g., 5 µM (Tolosa et al., 2016). In vivo literature was not retrieved, possibly due to the rapid onset of (cyto)toxicity: In rat in vitro (Isenberg and Klaunig, 2000), the onset of apoptosis was within 20 minutes of exposure, providing mechanistic support for the absence of steatosis.  Rotenone is reported as a model chemical to impair mitochondrial respiration (by blocking mitochondrial complex I), resulting in mitochondrial membrane potential alterations, oxidative stress, and apoptosis.  Rotenone was included as a reference or proficiency chemical in at least two European projects aimed to address hepatotoxicity or steatosis in in vitro methods: the LIINTOP project and SEURAT-1, indicating stakeholder support for inclusion of this chemical in the steatosis chemical selection. |  |
|  |  |  |  | Human, in vitro (differentiated HepaRG cells, 72 h, 1.5×10^-9^ - 1×10^-4^ M) | (not concluded) | Oxidative stress (ROS production) | (Landesmann et al., 2012) | Positive control for oxidative stress: 100 µM rotenone. Positive control is accompanied with substantial, concentration-dependent reduction in number (POD <10^-7^ M) and altered size (POD ~10^-6^.5 M) of hepatocytes and shows ROS-indicative nuclear staining (POD <10^-7^ M) compared to DMSO vehicle control. |  |
|  |  |  |  | Human, in vitro (differentiated **HepaRG, and HepG2** pre-loaded with fatty acids (62 µM oleate-palmitate (2:1) mixture for 14 h prior to exposure), 0.5-10 µM, 24 h) | No | Oxidative stress (ROS production), altered mitochondrial membrane potential | (Tolosa et al., 2016) | Evaluating the utility of HepaRG for assessment of steatosis confirmed lipid accumulation as the most sensitive endpoint; increased ROS production/oxidative stress and altered membrane potential were also observed.  **HepaRG exhibited superior lipid overaccumulation and thus sensitivity toward steatogenic chemicals, over HepG2**. Sensitivity of HepaRG was also greater to detect oxidative stress/ROS production; no significant differences were observed between the two cell lines regarding the detection of mitochondrial membrane potential changes. Both cell lines were pre-incubated with a 62 µM oleate-palmitate (2:1) mixture for 14 h prior to exposure, for fatty acid pre-loading; exposure to chemicals was in fatty-acid-free medium.  A review of information indicates toxicity via mitochondrial impairment, and apoptosis; rotenone is not primarily classified as an *a priori* steatogenic chemical at the onset of this study (“non-steatotic, hepatotoxic”), this was confirmed in HepaRG cells.  LOEC cell viability: 5 µM, LOEC lipid accumulation: > 10 µM (maximum concentration tested, and higher than cytotoxicity LOEC).  Gene expression analysis was conducted only for steatogenic chemicals, | High confidence, very high relevance |
|  |  |  |  | Review | No |  | (Gómez-Lechón et al., 2010) | Rotenone was selected for apoptosis and mitochondrial impairment assays, not for the steatosis assay due to no identified steatogenic potential.  Support for inclusion in this chemical selection was based on the RC-ZEBET in vitro/in vivo validation programme (Halle et al., 2003). | High confidence and relevance.  Mechanism-based chemical selection for in vitro hepatotoxicity approaches in the LIINTOP project (“Optimisation of liver and intestine in vitro models for pharmacokinetics and pharmacodynamics studies”) |
|  |  |  |  | Review | No steatosis at concentrations below cytotoxicity | Oxidative stress  Mitochondrial complex I inhibition  HIF-1α inhibition | (Jennings et al., 2014) | Rotenone was selected as a reference chemical for hepatotoxicity as a disruptor of mitochondrial function (inhibition of mitochondrial complex I). Affected biological pathways are related to oxidative phosphorylation, mitochondrial membrane potential and oxidative stress/ROS production, the anticipated primary hepatotoxic outcome is apoptosis, resulting in cytotoxicity.  Inhibition of complex I results in a shift of energy metabolism from oxidative phosphorylation to glycolysis.  Further data for this reference chemical are available online: <https://wiki.toxbank.net/wiki/Rotenone> (incl. curated information on toxicokinetics, genomics, proteomics, physico-chemical properties) | High confidence, chemical selection report for SEURAT-1 project, accepted by relevant stakeholders, including co-authors from EFSA, EURL ECVAM/JRC, academia, industry and national public health authorities |
|  |  |  |  | Human, in vitro (HepG2, 0.5-10 µM, 24 h) | No |  | (Donato et al., 2012) | HepG2 cells were pre-incubated with a 62 µM oleate-palmitate (2:1) mixture for 14 h prior to exposure, for fatty acid pre-loading; exposure to test chemicals was in fatty-acid-free medium. | High confidence and relevance  Study preceding (Tolosa et al., 2016) |
|  |  |  |  | Rodent, in vitro (rat liver cell line WB-F344, 0.5-5 µM for up to 12 h) | No |  | (Isenberg and Klaunig, 2000) | Induction of liver cell apoptosis through inhibition of mitochondrial respiration (complex I inhibition) in a time- and concentration-dependent manner.  Onset of apoptosis upon rotenone treatment was within 20 min, and peaked after 2 h; the morphology of cells then remained unchanged until 12 h post treatment. |  |
| **Metformin** | 657-24-9 | 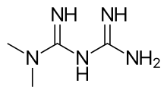 | Pharmaceutical; anti-diabetic | **SUMMARY** | **Negative**  **No effect/ reduction** |  |  | **Very strong weight of evidence supporting metformin not inducing primary hepatic steatosis**; this observation is consistent in vitro and in vivo, and metformin is being trialled for treatment of NAFLD and NASH.  Data on human in vivo hepatic lipid lowering efficiency is inconsistent (no effect or decreased lipid accumulation/improved NASH activity; never increased lipid accumulation); it is largely attributed to the insulin sensitizing (primary therapeutic) properties of metformin, improving glucose homeostasis in the liver.  Metformin reduces steatosis in experimental models. In clinical studies in patients with type 2 Diabetes Mellitus or hepatic steatosis, metformin reduced steatosis. In most studies in NASH patients, however, metformin had no effect on steatosis or other histological markers of liver function. |  |
|  |  |  |  | Human, in vitro (primary human hepatocyte 3D spheroid steatosis model, 10-100 µM, 7 d). | Reduction/ no effect |  | (Kozyra et al., 2018) | Metformin (50 and 100 µM) reduced lipid accumulation when it was applied 1 week after the induction of steatosis, but it was not able to prevent steatosis induction. |  |
|  |  |  |  | Human, in vitro (primary human hepatocytes and HepG2 cells treated with metformin (1 mM, treatment time not indicated) | Reduction | In cells: reduced mRNA levels of: ACSL1, TNF-α, FATP5, CD36, miRNA processing proteins [XPO5, DICER, AGO2], and increased p-AMPK levels, and several miRNAs (incl. miR-30b, 146b, 422a, 16, and 30b) | (Latorre et al., 2020) | In HepG2 cells, the effect of AMPK and endoribonuclease Dicer (DICER) knockdown on metformin’s effects were evaluated. The effect of knocking down AMPK in the liver, in vivo in mice was evaluated. Gene and miRNA expression analysis was also carried out with human liver biopsy samples with varying degrees of steatosis (none, borderline, substantial).  In primary human hepatocytes and HepG2 cells, metformin reduced lipid levels. In HepG2 cells, AMPK and DICER knockdown enhanced lipid accumulation, and counteracted the lipid lowering effects of metformin.  In mice, hepatic AMPK knockdown increased lipid accumulation. mIR-16, 30b, and 30c reduced lipid accumulation in cells, and in human liver samples, steatosis was associated with low levels of the miRNA. |  |
|  |  |  |  | Human and rodent, in vitro (AMPK KO vs. WT primary mouse hepatocytes, or primary human hepatocytes; 0.25-2 mM, 3 h). | Decreased intracellular triglyceride content | Increased p-AMPKα and p-ACC,  reduced mRNA levels: Acly, Acc, Chrebp, Fasn, S14 | (Boudaba et al., 2018) | Metformin (0.25 to 2 mM) decreased triglyceride content and lipogenesis, and increased β fatty acid oxidation, and these effects were completely or greatly reduced in AMPK KO cells. AMPK activators enhanced metformin’s effects.  In mice, liver-specific knockout of AMPK did not predispose to fatty liver. However, re-activation of downregulated AMPK in fatty liver, normalised hepatic lipid content by inhibiting lipid synthesis and stimulating fatty acid oxidation. |  |
|  |  |  |  | Rodent, in vivo (WT and Oct KO ob/ob mice (FVB/N or C57BL/6J background), males, n=3-6) | Not assessed; inferred no effect/reduction (via Oct1 inhibition) | Oct1 KO increased p-ACC, p-AMPK, and reduced Creb1, Crtc2, Pgc1α, Srebp1. | (Chen et al., 2014) | The results in the paper mainly relate to Oct1, and there are no results directly showing effects of metformin on fatty liver endpoints.  Studies were carried out in the mouse lines and in OCT1 expressing Hek293 cells to determine if metformin inhibits OCT1.  Oct1 KO reduced steatosis in ob/ob mice, and metformin was shown to inhibit Oct1 mediated transport of thiamine in vitro (IC_50_ = 1.4 mM) and in vivo (after a 50 mg/kg acute IV dose, or after 7 days chronic daily i.p. dosing with 100 mg/kg). |  |
|  |  |  |  | Rodent, in vivo ( C57BL/6N mice, methionine- and choline-deficient (MCD) diet-induced mouse NASH model; males, n=6-10/group; 2.4 mg/d metformin via drinking water for 15 weeks) | Decrease |  | (Katsura et al., 2015) | Metformin decreased MCD diet-induced hepatic lipid accumulation, fibrosis, and increased serum levels of alanine aminotransferase (ALT), aspartate aminotransferase (AST), and alkaline phosphatase (ALP). |  |
|  |  |  |  | Rodent, in vivo (C57BL/6J Ob/Ob and WT mice (sex not specified), n = 8/group, 300 mg/kg/d metformin, i.p., 4 weeks)  Human and rodent, in vitro (primary mouse hepatocytes and HepG2 cell steatosis models, 0.5 mM metformin, 2 h). | Decreased lipid accumulation | In the mouse and cell models, metformin upregulated **SIRT1** and autophagy. | (Song et al., 2015) | In ob/ob mice, metformin reduced hepatic lipid content, and serum levels of AST and ALT. In the cells, metformin reduced lipid accumulation. | High confidence and relevance |
|  |  |  |  | Rodent, in vivo (C57BL/6 mice on HFD, male, n = 5-7/group; metformin 50-200 mg/kg bw/d, oral by gavage for 15 d) | Decreased hepatic lipid levels | In the liver, metformin increased leptin receptor mRNA, and decreased Srebp-1c, Fasn, and Acc-1 mRNA . | (Tang et al., 2016) |  |  |
|  |  |  |  | Meta-analysis (n=9 randomized clinical trials) evaluating the effects of insulin sensitizers incl. metformin for the treatment of NASH in humans | No effect |  | (Rakoski et al., 2010) | Metformin did not improve hepatic steatosis, inflammation, fibrosis, or hepatocyte ballooning. |  |
|  |  |  |  | Meta-analysis of randomized controlled clinical trials evaluating the effects of metformin on NASH in humans | No effect |  | (Said and Akhter, 2017) | Metformin did not improve hepatic steatosis, ballooning, or fibrosis, and worsened inflammation. |  |
|  |  |  |  | Systematic review (n=91 articles) and meta-analysis evaluating the effectiveness of metformin in treating NASH | No effect |  | (Younossi et al., 2014) | Metformin reduced serum levels of AST and ALT in some trials, but failed to improve histological markers of NASH in most studies. Current guidelines do not recommend metformin for treating NAFLD. |  |
|  |  |  |  | Human, in vivo (prospective randomized clinical trial in obese patients with hepatic steatosis, n=25/group; 1 g/d metformin + dietary intervention, 6 months) | Reduction of steatosis prevalence | Decreased serum adiponectin | (Garinis et al., 2010) | Metformin reduced the proportion of people with hepatic steatosis.  Metformin decreased fasting plasma glucose, insulin resistance, serum adiponectin, weight, BMI, and waist circumference |  |
|  |  |  |  | Human, in vivo (clinical trial in T2DM patients, n = 12; insulin + metformin (dose titrated from 500 mg daily to 1000 mg twice daily), 3 months) | Reduces hepatic triglyceride content |  | (Lingvay et al., 2007) | Treatment with metformin and insulin in combination reduced liver triglyceride content.  The study did not include a metformin alone group, due to necessary management of diabetes. | Small sample size |
|  |  |  |  | Human, in vivo (pilot clinical trial of metformin for the treatment of NASH, n = 26 (13 female); 2000 mg/d, 48 weeks) | No effect on steatosis, but improvement of NASH |  | (Loomba et al., 2009) | Endpoints assessed were repeat metabolic studies, magnetic resonance imaging, and liver biopsy. Histological response to metformin treatment was achieved in 30% of study participants; most patients lost weight. Weight loss was significantly associated with improved NASH activity score and serum ALT levels (p<0.01). Improved insulin sensitivity did not correlate with histological improvement. |  |
|  |  |  |  | Human, in vivo (open-label pilot clinical trial for metformin treatment of NAFLD, n = 15 in total, n = 10 for biopsy samples; 20 mg/kg bw/d for 1 year) | Reduction/no effect |  | (Nair et al., 2004) | Metformin reduced liver steatosis (in 3/10 patients), inflammation (in 2/10) and fibrosis (in 1/10), and transiently reduced serum ALT and AST. |  |
|  |  |  |  | Human, in vivo (randomized clinical trial for NASH treatment, n = 17/group; 850 mg bidaily + dietary intervention for 6 months) | No effect |  | (Uygun et al., 2004) | Metformin reduced serum levels of ALT and AST, but had no effect on hepatic necro-inflammatory activity or fibrosis. Metformin reduced insulin resistance and BMI.  Hepatic steatosis was assessed by ultrasonography. |  |
|  |  |  |  | Human, in vivo (randomized, placebo-controlled pilot clinical trial evaluating metformin treatment (titrated 500-1000 mg/d + lifestyle interventions) in nondiabetic patients with insulin resistance and NASH n=7-9/group for 12 months) | No effect |  | (Shields et al., 2009) | Metformin had no effect on liver steatosis, ballooning, inflammation, fibrosis, or serum ALT and AST levels. |  |
|  |  |  |  | Human, in vivo (phase 2 clinical trial evaluating metformin for treatment of NASH in non-diabetic children, n=10, 500 mg twice daily for 24 weeks) | Reduction of fat content in liver |  | (Schwimmer et al., 2005) | The design was a single-arm open-label study of oral metformin 500 mg twice daily for 24 weeks. In order to minimize gastrointestinal side-effects, subjects were initially started at metformin 500 mg once daily. After subjects demonstrated tolerance of metformin for a minimum of 1 week the dosing was increased to 500 mg twice daily.  Metformin reduced liver fat measurements (p<0.01), and serum ALT and AST levels. Metformin improved insulin sensitivity and overall quality of life. |  |
|  |  |  |  | Human, in vivo (randomized, placebo-controlled clinical study for metformin to treat NASH, n = 19-22/group); 850-1700 mg/d for 12 months) | Not assessed/ concluded |  | (Shargorodsky et al., 2012) | Focus of the study was on cardiovascular parameters, not lipid accumulation in liver.  Metformin transiently reduced serum ALP and ALT, but not AST. The study did not evaluate other markers of liver function. |  |
|  |  |  |  | Human, in vivo (epidemiology; population-based cohort of post-menopausal women, n = 345) | Potentially increased odds of steatosis in non-Hispanic white women |  | (Kim et al., 2013) | Study evaluating the racial/ethnic differences in hepatic steatosis.  Use of metformin and thiazolidinediones (pioglitazone) was associated with an increased odds of hepatic steatosis in non-Hispanic white women, but not in African-Americans; African-American women who used metformin and thiazolidinediones had the lowest prevalence of hepatic steatosis (19%) and non-Hispanic white women who used medications had the highest (71%).  Self-reported use of medication may be a proxy for other risk factors predisposing the individual, or contributing to hepatic steatosis, though this does not explain the difference in odds observed for different ethnicities. |  |
| **2-propylvaleric acid (valproic acid)** | 99-66-1 | 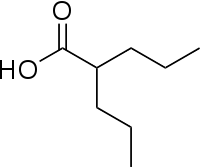 | Pharmaceutical; treatment of epilepsy, seizures, bipolar disorder, migraine prevention. Branched short-chain fatty acid | **SUMMARY** | **Positive**  **Induction** |  |  | **Strong evidence from human in vitro and in vivo studies** for valproic acid (usually administered as sodium valproate) **inducing hepatic steatosis**.  In rodent, evidence supporting induction of primary hepatic steatosis is **moderate**.  A possible mode of action for increased hepatic lipid accumulation is through the valproate metabolite valproyl-CoA (valproic acid Cofactor A ester) inhibiting mitochondrial fatty acid β-oxidation; bioactivation of valproate to the CoA ester seems essential for the induction of lipid accumulation.  Inhibited/decreased mitochondrial fatty acid β-oxidation was sometimes accompanied by an increase in gene or protein expression of relevant components of the mitochondrial fatty acid β-oxidation pathway, probably as a physiological reaction to counteract the decreased fatty acid oxidation.  Induction of steatosis is usually observed at higher concentrations (in the millimolar range), but therapeutic levels observed in human serum overlap with such high experimental concentrations.  **Prototypical chemical inducing steatosis, that has also been previously selected as a steatosis-positive reference chemical in other EU-funded projects, such as the SEURAT-1 and LIINTOP projects.**  In most experimental model studies in cells and rodents, valproic acid induced lipid accumulation. In many of the cell models, high mM concentrations (0.5-15 mM) are used to induce lipid accumulation. In some patients, valproic acid produces hepatotoxicity, including steatosis. |  |
|  |  |  |  | Human, in vitro (HepG2 and differentiated HepaRG, pre-loaded for 14 h with 62 µM oleate palmitate (2:1); 1-8 mM, 24 h) | Induction | Oxidative stress (ROS; significant at all tested concentrations), decreased mitochondrial membrane potential (significant at ≥4 mM)  Gene expression changed: EHHADH, MTTP, IGFBP1, SREBP1C, ANGPTL3, FOXA1, PGC1A | (Tolosa et al., 2016) | Exposure of cells to test chemicals was conducted in fatty-acid-free medium.  Valproate is included as a model steatogenic chemical (Drug-induced liver Injury category: “severe”), also causing mitochondrial impairment and oxidative stress.  In HepaRG: Significant lipid accumulation at all tested concentrations. Cell viability was reduced at 8 mM.  Gene expression analysed (8 mM), but unchanged: CROT, PPARA | Very high confidence and relevance |
|  |  |  |  | Human, in vitro (differentiated HepaRG, pre-loaded for 24 h with 83 µM oleate and 167 µM palmitate (in 0.25% BSA); 0.5-21 mM, 3 or 7 days) | Increased lipid accumulation (≥3 mM) | Gene expression: ↑ ADRP, ↑ CD36, ↑ FATP1+2, ↓ FATP5, ↑↓ ACSL5, ↑ CPT1, ↓↑ SREBP-1c, ↑ FAS, ↑ GPAT1, ↑ DGAT2, ↓ FABP, ↓ MTTP, ↓ ApoB100.  Further: oxidative stress (↑ ROS), ↑ fatty acid β-oxidation | (Grünig et al., 2020) | 1, 3, 6, 9, 12, 21 mM produced lipid accumulation vs control levels of ~1, 1.2, 1.4, 1.7, 1.8, and 1.4x after 3 days; and ~0.9, 1.1, 1.9, 2, 2.3, 1.2x after 7 days.  LOEC lipid accumulation: 3 mM (3 days), 6 mM (7 days).  Intracellular **ATP content was decreased at ≥9 mM** (3 day exposure; membrane toxicity by adenylate cyclase release from 6 mM). Flow cytometry indicates a substantial share (~30 %; ~15% in control) of cells exposed to ≥12 mM valproate for 3 days are at least in early apoptosis (Annexin V stainig) (7 days: ~40 % (~15% in control), at ≥6 mM valproate).  LOEC β-oxidation of ^14^C-palmitate (relative to colvent control): 12 mM (in isolated fresh primary mouse mitochondria; decrease), 12 mM (HepaRG; 15 min acute exposure; decrease), no effect (HepaRG, 3 day exposure), ≤1 mM (HepaRG, 7 day exposure; increase). Increased β-oxidation after 7-day exposure correlates with a significant increase in mitochondrial copy number (significant at ≥3 mM).  Other effects of valproic acid incl.: ↓ membrane intactness, ↑ citrate synthase activity, ↓ β-ketothiolase activity, Δ acylcarnitine patterns, ↑ free fatty acids, ↓ secreted ApoB100, , ↓ activity of mitochondrial complex I/II/III, ↓ cytochrome b protein. Many of the effects were observed at concentrations ≥1 or ≥3 mM. | Very high confidence and relevance |
|  |  |  |  | Human, in vitro (cryopreserved differentiated HepaRG (HPRGC10); 66.5-2300 µg/mL, 24 or 72 h) | Increased diacylglycerol and triglyceride levels | Metabolomic changes induced include: ↓carnitine, Δ spermidines, ↓ creatine, ↓ acetylcholine.  Upregulation of neutral lipids (triglycerides and ceramides) alongside downregulation of carnitine (as a result of inhibited β-oxidation)  S-adenosylmethionine (suggested as possible marker of NAFLD; key metabolite in one-carbon metabolism) | (Cuykx et al., 2018) | This study characterized the metabolic (metabolomic) profile of steatosis in HepaRG cells, with sodium valproate as the positive control and model inducer of steatosis.  Initial range-finding experiments were performed with 250-10,000 µg/mL (24 h) or 47-10,000 µg/mL (72 h); IC_10_ values (Neutral Red Uptake assay) and 1/10^th^ IC_10_ were used for subsequent metabolomics experiments.  IC_10_ values: 2,300 µg/mL (24 h), 665 µg/mL (72 h)  The testing concentrations (IC_10_ and 1/10 IC_10_) induced some morphological changes to the differentiated cultures: cells remained as clearly distinguishable clusters of hepatocytes from biliary cells. The 24 h 1/10 IC_10_ exposed samples showed some faint indication of faded lining between hepatocellular clusters and biliary cells; After 72 h cellular stress was morphologically evident (less organized hepatic clusters and biliary cells), without substantial dead cells. IC_10_ concentration severely distorted cell morphology, and effects were exacerbated by longer incubation (72 h). Cell appeared swollen, with blurred edges. Clusters were disorganized and cellular debris indicates some cell death.  Increases in triglyceride levels were observed at ≥66.5 µg/mL (lowest tested concentration). Statistical analysis was done by principal component analysis (unsupervised multivariate statistics; samples within a 95% confidence interval were accepted for further analysis) and partial least squares-discriminant analysis (supervised multivariate statistics). Molecular features of interest were selected based on the correlation of the signal variance between exposure group and the negative control group, the impact of the feature on the model based on abundance differences and a visual graphic control of the abundance differences in the plot between both groups. Two independent experiments were included in the analysis; only markers reliably identified and concordant in both experiments were included in the comparative analysis (92 metabolites after 24 h exposure; 113 metabolites after 72 h exposure).  Decreased incorporation of saturated fatty acids in favour of incorporation of heavy, unsaturated acyl-chains into triglycerides (possibly via the arachidonic acid metabolism pathway). | Very high confidence and relevance.  A large extent of supplementary data available with the publication. |
|  |  |  |  | Human, in vitro (HepG2 pre-loaded with 62 µM oleate-palmitate (2:1) for 14 h pre-exposure; 0.125-20 mM valproate, 24 h in fatty acid-free medium) | Increased (at ≥0.5 mM) |  | (Donato et al., 2012) | Multiparametric high-content image analysis assay.  Includes lipid accumulation data for the following chemicals: Steatogenic chemicals: acetylsalicylic acid, amiodarone, clofibrate, cyclosporine A, didanosine, doxycycline, DL-methionine, fenofibrate, fialuridine, stavudine, tamoxifen, tetracycline, tianeptine, ticlopidine, and zidovudine Negative controls: amikacin, rotenone, t-butyl hydroperoxide, cumene hydroperoxide, colchicine, citrate.  0.125, 0.25, 0.5, 1, 2, 4, 10, and 20 mM gave rise to % control lipid levels of 115, 120, 125, 140, 150, 170, 190, 220%.  LOEC: lipid accumulation (0.5 mM; increase), cell viability (4 mM; decrease), ROS (10 mM; increase >5-fold), mitochondrial membrane potential (10 mM; decrease) |  |
|  |  |  |  | Human, in vitro (primary human hepatocytes; 15 mM, 5 d) | Induction | Changes in methylation patterns in nuclear DNA regions associated with oxidative stress and steatosis pathways, and hypomethylation in mtDNA regions | (Wolters et al., 2017) | Valproic acid induced observable steatosis (effect size not indicated) after 24h at 15mM. 15 mM did not induce cytotoxicity after 24 or 48h. |  |
|  |  |  |  | Human, in vitro (cryopreserved primary human hepatocytes, 0-30 mM; 1-5 d) | Induction inferred | . Expression changes in steatosis relevant genes: ↑ PPARα+γ, ↓ LXRα, ↓ LXRβ, ↓ RXR, ↑ AhR, ↓ ChREBP, ↓ SREBP-1c, ↓ SCD1, ↓ ABCA1, ↑ ABCG1, ↓ ABCG5+8, ↓ APOE, ↓ CYP7A1, ↓ ACC, ↑ CD36. | (van Breda et al., 2018) | Cryopreserved primary hepatocytes from three donors were obtained (Hu4197, Hu8084, Hu4227) and pooled for further cultivation and analysis; cells were cultured in a 2-layer collagen sandwich according to the cell supplier’s protocol.  An initial screening experiment with 0-30 mM valproate for cytotoxicity (MTT assay) and lipid accumulation (BODIPY staining) was conducted.  Valproic acid is said to have induced fat accumulation in a dose-dependent manner at 24 and 48h, but the data is not shown. No cytotoxicity was observed.  An AOP for chemically induced liver steatosis is presented in the paper.  Valproic acid altered the expression of many genes and miRNAs, and also altered the methylation of many genes. The data indicated that valproic acid inhibited expression of the transcription factors HNF1A and ONECUT1. Pathways modulated included those involved in complement and coagulation cascades, nuclear receptor pathways, apoptosis, drug metabolism, fatty acid metabolism, inflammation, carcinogenesis, and general metabolism and signalling pathways |  |
|  |  |  |  | Human, in vitro (differentiated E6/E7^LOW^ human hepatocytes (co-cultured 1:1 with microvascular cardiac endothelial cells) or HepG2/C3A cells; time-resolved monitoring up to 46 h exposure; 5-30 mM) | Increased accumulation of neutral and phospholipids  TC50 (46 h) = 2.5±0.4 mM in 2D; strong and significant increase in neutral lipids | Increased lipogenesis and mitochondrial stress, and decreased respiration, glycolysis, and ATP production. | (Ehrlich et al., 2018) | Microfluidic sensor-integrated liver on chip array in which oxygen is monitored using two-frequency phase modulation of tissue-embedded microprobes, while glucose, lactate and temperature are measured in real time using microfluidic electrochemical sensors.  Valproic acid increased lipid accumulation (~2x in E6/E7^LOW^ cells, and ~4x in HepG2 organoids) and phospholipid accumulation (~0.7x in E6/E7^LOW^ cells, and ~1.2x in HepG2 organoids) (it is not clear at what concentrations the latter fold changes in lipids were observed)  The cytotoxicity EC_50_ was 2.5 mM in E6/E7^LOW^ cells and 14 mM (42 h exposure) or -27 mM (24 h exposure) in HepG2/C3A organoids; differences in cytotoxicity thresholds in the two cell lines suggests requirement of metabolic activation for toxicity. In HepG2/C3A organoids, time to onset of metabolic response (based on increased oxygen consumption) was 6-36 h (30-5 mM valproate, respectively); the lowest effect level was calculated to be 280±97 µM valproate (at infinite time).  Valproate exposure led to a sustained 15% increase in lipogenesis followed by mitochondrial stress (E6/E7^LOW^ cells: TC_50_ = 2.5±0.4 mM in 2D; HegG2/C3A cells: TC_50_ = 27±2 mM (24 h), TC_50_ = 14±2 mM (42 h)); significant induction of steatosis in 2D and 3D cultures. The accumulation of neutral lipids was strong and significant (p<0.001, n=4), and evidence of phospholipidosis was seen.  Time to onset of steatosis in differentiated HepG2/C3A cells was concentration and time dependent (time to onset: 6-36 h; 6 h in 30 mM; 36 h in 5 mM).  Oxygen consumption is monitored using tissue-embedded microprobes, coupled with real-time measurement of glucose, lactate and temperature. Dynamic changes in metabolic fluxes around central carbon metabolism are calculated, producing a unique fingerprint of the liver’s response to stimuli and allowing detection of effects below the threshold of cellular damage. | High confidence, moderate-high relevance  Time-resolved in vitro metabolic data |
|  |  |  |  | Human, in vitro (HepG2; 25-100 µM, 24 h) | No accumulation of neutral lipids |  | (Park et al., 2012) | Valproic acid increased phospholipidosis at 100 µM but had no effect on steatosis. No cytotoxicity was observed.  Drug-induced phospholipidosis and/or steatosis was assessed based on LipidTox staining, NBD-PE assay, and, in case of disagreement of results, confirmed by transmission electron microscopy.  Contains steatosis results for several other chemicals, including amiodarone and acetaminophen. |  |
|  |  |  |  | Human, in vitro (HepG2, 0.5-16 mM, 0-120 h) | not assessedin healthy culture | Gene expression: ↑ GRP78/BiP, ↑ calreticulin, ↓ GADD153  Enzyme activity: **↓ GSK3β activity**, SREBP-2 | (Kim et al., 2005) | ER stress was induced by tunicamycin, A23187, or glucosamine. Effects of valproate to revert or exacerbate it were observed.  Valproate (pre-treatment with 0.5 mM, 18 h) prevented ER-stress-mediated apoptosis and intracellular lipid accumulation.  Protection from ER stress is mediated via the (overexpressed) ER-resident chaperones GRP78/BiP and calreticulin (i.e., independent of PERK pathway). Pre-treatment also prevents expression/maturation of (mature) SREBP-2 protein – a regulator of lipid uptake/metabolism, that is activated by ER stress.  Cytotoxicity (LDH release assay) >10% at ≥8 mM. |  |
|  |  |  |  | Human, in vitro (Huh7, 0.5 mM, 24 h) |  |  | (Elphick et al., 2012) | In addition to human Huh7 cells, *Dictyostelium discoideum*, was used as a model organism to study lipid droplet formation and accumulation in a shorter timeframe (30 min).  Valproic acid increased lipid accumulation in *Dictyostelium*, 0.5 mM valproate increased lipid droplet intensity by ~150% of control, in Huh7 cells lipid droplets were increased as well.  In *Dictyostelium*, valproic acid increased fatty acid uptake, and decreased fatty acid secretion.  Structure-activity relationship *in silico* analysis reveals that **lipid accumulation occurs independently on valproate-mediated teratogenicity** and inositol depletion.  A large array of other, structurally related, chemicals was studied for lipid accumulation in *Dyctiostelium* in parallel. |  |
|  |  |  |  | Human, in vitro (HepG2, 5-10 mM, 24 h).  Human, in vivo (biopsy-proven NAFLD patients; n=32; non-NAFLD subjects who underwent surgery for cholelithiasis, n=10)) | Not assessed (study focused on changes in miRNA) | In cells, valproate produced the following miRNA subtype changes: ↑ 3929, ↑ 663a, ↑ 1260a, ↑ 21-5p, ↑ 202-3p, ↑ 22-5p, ↑ 29a-3p.  miR-29a-3p and 22-5p were increased in serum from NAFLD patients. | (López-Riera et al., 2017) | Evaluated changes in miRNA profiles induced by steatogenic drugs including valproate (5 and 10 mM, 24h) in HepG2 cells. Also measured miRNA profiles in serum from NAFLD patients.  Contains miRNA expression profile data for several other steatogenic and non-steatogenic drugs.  NAFLD patients receiving fibrates as medication showed increased miRNA markers and showed a more severe steatosis grade.  Intracellular lipid accumulation (neutral lipids, triglycerides was assessed for a subset of chemicals used in this study, and were not available for valproate. |  |
|  |  |  |  | Human, in vitro (primary human hepatocytes, 0.2-5 mM, 2-24 h) | Not assessed | Increased NEURL1B expression, KEGG pathways modulated by valproic acid included: ↑ fatty acid degradation, ↑ PPAR signalling, ↑ retinol metabolism. | (Aguayo-Orozco et al., 2018) | The “*Toxicogenomics Project-Genomics-assisted Toxicity Evaluation System*” (TG-GATE) database was used as a resource of gene expression data (recorded on an Affymetrix HG U133 Plus 2.0 microarray chip).  Some of the gene expression data presented is for the overall cumulative sum of expression changes induced by multiple steatogenic chemicals. Valproic acid increased neutralized E3 ubiquitin protein ligase 1B (NEURL1B) expression, and sequesters coenzyme A by reacting with it to form VPA-CoA  Contains toxicogenomic data for 7 negative control chemicals (carbamazepine, diclofenac, indomethacin, naproxen, nifedipine, nimesulide, sulindac) and the following 28 steatogenic chemicals: allyl alcohol, amiodarone, acetaminophen, acetamide, amitriptyline, aspirin, coumarin, colchicine, clomipramine, cyclosporin A, clozapine, diltiazem, disulfiram, ethanol, ethinylestradiol, ethionamide, hydroxyzine, imipramine, lomustine, methapyrilene, methyltestosterone, phenylbutazone, rifampicin, terbinafine, tetracycline, vitamin A, valproic acid, pirinixic acid. |  |
|  |  |  |  | Human, in vitro (iPSCfrom healthy subjects and ALper’s sundrome patients, 20 mM, 12-24 h). | Not assessed |  | (Li et al., 2015a) | Alpers syndrome is a neurometabolic disorder caused by mutations in mitochondrial DNA polymerase (POLG) that is associated with an increased risk of fatal valproic acid hepatotoxicity.  The following effects produced by valproic acid were greater in the Alpers hepatocytes: ↑ apoptosis, ↑ cleaved caspase 9, ↑ cytosolic cytochrome C; and the augmented apoptosis observed in the Alpers hepatocytes were counteracted by carnitine, N-acetylcysteine, or cyclosporine A. |  |
|  |  |  |  | Human, in vitro (primary human hepatocytes, 0.2-5 mM, 2-24 h) | Not assessed | Gene expression altered: AHR, ESR1, NR1H4, GR (NR3C1), PPARG. | (AbdulHameed et al., 2019) | The “*Toxicogenomics Project-Genomics-assisted Toxicity Evaluation System*” (TG-GATE) database was used as a resource of gene expression data (recorded on an Affymetrix HG U133 Plus 2.0 microarray chip).  Contains toxicogenomic data for the following 18 model steatogenic chemicals: amiodarone, amitriptyline, bromobenzene, carbon tetrachloride, colchicine, coumarin, diltiazem, disulfiram, ethanol, ethionamide, ethinyl estradiol, hydroxyzine, imipramine, lomustine, puromycin aminonucleoside, tetracycline, vitamin A, and valproic acid.  Valproic acid did not modulate any Srebf1 target genes. It is mentioned that valproic acid causes mitochondrial toxicity, which leads to oxidative stress. |  |
|  |  |  |  | Human, in vitro (primary human hepatocytes or liver tissue samples, 0.2-5 mM, 2-24 h) | Not assessed |  | (Grinberg et al., 2014) | Analysed data in publicly available gene expression databases, including the Open TG-GATES database and genome-wide data sets from human liver tissue samples, as well as generating further data with primary human hepatocytes n=5 donors for 5 mM valproate 24 h exposure; n=8 donors to determine “unstable baseline genes”) (control; cultured in collagen sandwich up to 14 d). The aim was to identify changes in gene expression induced in hepatocytes by 143 chemicals (incl. valproic acid). Also carried out experiments to evaluate the gene expression changes induced by valproic acid and a few other chemicals in primary human hepatocytes and compared the results to that from the databases.  Up- and down-regulation were defined as at least threefold difference from freshly isolated hepatocytes/baseline.  Genome-wide liver disease datasets used from public repositories: ArrayExpress (E-MEXP-3291) and Gene Expression Omnibus (GSE25097). E-MEXP-3291. (NASH: n=16 vs. health liver: n=19; cirrhosis: n=40 vs. non-tumour liver tissue: n=243). RNA seq data: hepatocellular carcinoma (n=163) vs. matched non-tumor tissue (n=49) (Genes with a fold change of at least 1.3 and a false discovery rate (FDR) adjusted p value ≤0.05 in the pairwise comparison of healthy/non-tumor tissue to diseased tissue were considered differentially expressed).  Data from the databases showed valproic acid to modulate the expression level of many genes. Gene expression data generated in primary hepatocytes corresponded to the database results for only some cases. E.g., **TG-GATE data suggested that the gene THRSP, which is implicated in the pathogenesis of liver steatosis, is upregulated, however, this could not be confirmed in primary hepatocytes in vitro in this study.** | Very high confidence, moderate relevance |
|  |  |  | Valproyl-CoA ester | Human, in chemico (fibroblast preparations; 10-100 µM)  Rodent, in chemico (*S.cerevisiae*-expressed recombinant CPT1A, ) | Not assessed, but induction can be inferred from mechanistic data. | inhibition: CPT I activity. | (Aires et al., 2010) | CPT1 is the rate-limiting enzyme in mitochondrial fatty acid β-oxidation pathway.  The anti-epileptic drug and branched-chain fatty acid valproate is bioactivated to the CoA ester, and has previously been shown to inhibit mitochondrial fatty acid β-oxidation.  In this study, valproyl-CoA inhibited human CPT1 and rat CPT1A. this inhibition was competitive with palmitoyl-CoA and non-competitively to carnitine. |  |
|  |  |  |  | Rodent, in vivo (B6.V-*Lep^ob^*/J leptin obese mutation mice (n=4/ group, sex not stated), 0.5% w/v valproate, 14 d via drinking water)  Mouse, in vitro (primary hepatocytes from male C57BL/6 mice, 800 µM, 1-24 h)  Human, in vitro (primary hepatocytes (four preparations; details see on right), (0.2-2 mM) 800 µM, 1-24 h) | Decreased lipid accumulation and liver weight in obese mice in vivo. (Serum triglycerides decreased, but not significantly. | In vitro: increased **AMPK**, ACC (via metabolite)  HDAC(1) inhibition | (Avery and Bumpus, 2014) | Exposure to: 800 µM VPA, or 20 µM 2-ene-VPA, 4-ene-VPA, 3-OH-VPA, 3-ketoVPA, or vehicle solvent (DMSO or water).  Human primary hepatocytes were isolated from four individuals: male (55 years old), female (43 years old), female (59 years old), male (36 years old); with viabilities of >75 %.  In the mice, valproic acid decreased hepatic fat accumulation (based on a qualitative evaluation of histology images only), and liver mass.  In the cells, valproic acid or its metabolites increased. AMPK and acetyl CoA carboxylase (ACC), and these effects were counteracted by an AMPK inhibitor and a CYP inhibitor. Valproic acid inhibited human recombinant HDAC1 and HDAC activity in mouse hepatocyte nuclear extracts. | Moderate-low relevance (due to the use of an obesity rodent model) |
|  |  |  |  | Rodent, in vivo (SD rats, male, n = 8/group, 250 and 500 mg/kg bw/d i.p, 4-15 d). | Induction; significant at 500 mg/kg bw/dfor 4 days, 250 mg/kg bw/d for 15 d) | Valproic acid altered the levels of 24 hepatic lipids and 36 plasma lipids. | (Goda et al., 2018) | Lipidomics were performed on the livers and plasma of rats.  At 250 and 500 mg/kg/day doses steatosis was induced (effect size not quantified), and hepatic triglyceride levels were increased at 4 day (mg/g levels for the control, 250 and 500 mg/kg groups = 5.55, 6.7, and 10.85^*^) and 15 day (mg/g levels for the control, 250 and 500 mg/kg groups = 5.58, **10.78^*^,** and 4.63) timepoints, * = statistically significant. Lack of significant increase after 15 d exposure potentially due to toxicity: this dose resulted in decreased locomotor activity and ataxic gait immediately after dosing in all animals throughout dosing with this dose (500 mg/kg bw/d); bodyweight was significantly (p<0.01) lower in this group at 15 d treatment, and this was considered greater than the maximum tolerated dose. No treatment-related changes in body weight or food consumption were observed in the 250 mg/kg bw/d group. | High confidence and relevance |
|  |  |  |  | Rodent, in vitro (FL83B murine hepatocytes, 0.02-10 mM, 24 h; and/or co-exposure with oleic acid). | No effect (but valproate exacerbated oleic acid-induced intracellular lipid accumulation) | Gene expression: ↑ Cd36, ↑ Lrp, ↑ Dgat2, ↓ Ldlr. | (Chang et al., 2016) | Oleic acid increased lipid accumulation, and oleic acid induced lipid accumulation was significantly potentiated by valproic acid at ≥0.1 mM, with 0.1, 0.5, 1, 5, and 10 mM increasing lipid accumulation by ~4.2, 4.5, 6.1, 7, and 8 fold compared to a ~2.5x increase induced by oleic acid alone. In combination with oleic acid, 5 and 10 mM valproic acid reduced cell viability by ~30 and 45%, respectively.  Oleic acid induced mRNA changes included: ↑ Acca, ↑ Fasn, ↑ Ldlr, ↑ Plin2 mRNA, and oleic acid’s effects on Acca, Fasn, and Ldlr were counteracted by valproic acid, while the increase in Plin2 induced by oleic acid was potentiated by valproic acid. Valproic acid induced PPARγ nuclear translocation and increased CD36 cell surface levels. |  |
|  |  |  |  | Rodent, ex vivo (liver slices from C57BL/6 male mice (n = ≥4 mice), 24 weeks old. 0-500 µM (200 µM for gene expression profiling), 24 h) | Not assessed. Valproate was studied as a model steatogenic chemical | Gene expression altered: Abcd3, Acat1, Ehhadh, Ly6d, Lpcat3, Pex1, Pck2, Fgf21 | (Szalowska et al., 2014) | 5 mm-diameter cylindrical liver cores were obtained by biopsy; after processing, slices were 0.2 mm thick and weighted ~6 mg per slice.  Also tested valproic acid in PPARα/γ/β/δ CALUX human U2-OS reporter cell line assays.  Genes significantly upregulated (fold-change ≥1.5) in GSEA and proposed as biomarkers for amiodarone/valproate-like acting chemicals: Abcd3, Acat1, Ehhadh, Ly6d, Lpcat3, Pex1, Pck2, Fgf21 Valproic acid modulated genes related to the following processes: ↑ lipid metabolism, ↓ extracellular matrix remodelling, ↓ inflammation, Δ PPAR signalling. In PPAR reporter assays, valproic acid activated PPARα/γ/(β/δ).  While amiodarone and valproate induced gene expression changes clustered together, tetracycline induced a distinctly different pattern. Main differences were found in gene sets related to lipid metabolism, fatty liver, and peroxisomes, which were upregulated by both AMI and VA, and downregulated by TET.  AMI and VA downregulated several GO annotation clusters affiliated to immune functions, extracellular matrix, and development  In contrast to AMI and VA, TET downregulated functional clusters related to lipid synthesis, β-oxidation, *Pparα* signalling, inflammation, apoptosis, and other clusters related to energy and bile acid homeostasis. |  |
|  |  |  |  | Review on ADME screening tools and clinical pharmacology strategies to aid NASH drug discovery (focus on rodent studies). | Not assessed specifically, but mentions modulation of microvesicular steatosis |  | (Dash et al., 2018) | It is mentioned that in rats, valproic acid modulated microvesicular fatty liver and hepatic dysfunction via its effects on fatty acid β-oxidation, in co-administration with high-fat diet. | High confidence, moderate-low relevance |
|  |  |  |  | Human, in vivo (cross-sectional case-control clinical study, n=38 children (14 receiving valproate, 10 healthy control), cumulative valproate dose: 26.93±18.17 g, ≥18 months (42.00±26.73 months)) | Increased liver fat content, and biochemical markers (ALT, AST) |  | (Saleh et al., 2012) | Valproic acid treatment was associated with increased liver fat content.  Valproate treatment lasted for 42.00±26.73 months (mean ± SD), with a cumulative dose of 26.93±18.17 g valproate, resulting in valproate serum levels of 70.59±5.70 µg/mL.  Study participants were epileptic children or adolescents receiving valproate (n=14), carbamazepine (n=14), or both pharmaceuticals (n=10) for treatment (total n=38; 21 male, 17 female, age 2.5-14 years, mean: 8.4±3.42)). Subjects were divided into groups by BMI (even distribution of lean or overweight subjects per group). 10 healthy control subjects (5 male, 5 female; age 3.5-13 years (mean: 7.7±3.19)) were included in this study. Abdominal Computerized Tomography (CT), and biochemical measurements of fasting insulin, glucose, serum lipids, and liver enzymes were recorded throughout the course of this study. Fatty liver was determined by CT and detected in 42.8% of valproate-only treated subjects (21.4% receiving carbamazepine only, 60% when receiving both drugs) and no indication of fatty liver was found in healthy subjects. All fatty-liver-positive subjects were overweight, and 53.3 showed insulin resistance.  No significant changes in valproate-only treatment vs. control were detected for serum lipid profile (total cholesterol, triglycerides, low/high density lipoprotein), atherogenic ratio, or apolipoproteins (ApoA1, ApoB100).  The authors conclude that valproate therapy is associated with increased risk of insulin resistance (HOMA-IR increased, p<0.05; glucose/insulin ratio: decreased, p<0.001) and non-alcoholic fatty liver disease (ALT and AST increased, p<0.05; liver/spleen attenuation decreased, p<0.001), while carbamazepine therapy was associated with dyslipidaemia. | Moderate confidence, high relevance.  Small sample size |
|  |  |  |  | Human, in vivo (case report of hepatotoxic adverse effects in 2 infants treated with valproic acid) | Increase |  | (Appleton et al., 1990) | Valproic acid was associated with hepatotoxicity in 2 infants, including steatosis, fibrosis, necrosis ↑ AST, and liver failure. | Very small sample size, case report |
|  |  |  |  | Human, in vivo (case report of hepatotoxicity in one patient that was associated with sodium valproate) | Not reported |  | (Palm et al., 1984) | There is no mention of steatosis. Valproate treatment was associated with hepatotoxicity, including liver failure, and hepatic necrosis. | Low relevance  Very small sample size, case report |
|  |  |  |  | Human, in vivo (Valproate-associated liver failure pharmacovigilance study, Germany). | Not specified |  | (Schmid et al., 2013) | This study evaluated all reported cases (to the German Federal Institute for Drugs and Mediacal Devices) of adverse hepatic (side) effects in the treatment with valproate between 1993 and 2009.  132 cases of severe valproate-associated liver failure were identified, of which 34.8% were under valproate monotherapy. 34/132 cases (25.8%) had a fatal outcome (of which 32.4% were under valproate monotherapy).  Valproic acid was associated with hepatotoxicity, including liver failure. Contains no information on hepatic steatosis though. | High confidence, moderate-low relevance |
|  |  |  |  | Human, in vivo (pharmacovigilance report for adverse drug reactions in children, United Kingdom) | Not specified |  | (Clarkson et al., 2002) | Sodium valproate was associated with 21 cases of fatal hepatic failure in children. It is not mentioned if valproate was associated with steatosis. | High confidence, moderate-low relevance |
|  |  |  |  | Review on the selection of reference chemicals for liver pathologies, including steatosis | It is mentioned that valproic acid induces microvesicular steatosis, and that it is a reference chemical for steatosis. | ↓ CoA, ↓ pyruvate dehydrogenase, ↓ α-ketoglutarate dehydrogenase, ↓ fatty acyl-CoA dehydrogenases, ↓ carnitine shuttling.  Modulates the following signalling pathways: ERK, PKC, GSK-3, Akt/PBK.  Metabolism: PGH1, CYP3A5, CYP2C9, CYP2A6, CYP2B6, UDP-GST | (Jennings et al., 2014) | Further data for this reference chemical are available online: <https://wiki.toxbank.net/wiki/Valproic_Acid> (incl. curated information on toxicokinetics, genomics, physico-chemical properties)  Steatotic mechanisms: Valproic acid, as a fatty acid, competes with fatty acids in the β-oxidation pathway, which accounts for 40% of its metabolism and presumably underlies its steatogenicity.  Therapeutic activities: **increased GABA activity**, attenuation of the NMDA receptor, inhibition of Na^+^ channels, inhibition of voltage dependent L-type Ca^2+^ channels, inhibition of voltage-gated K^+^ channels | High confidence, chemical selection report for SEURAT-1 project, accepted by relevant stakeholders, including co-authors from EFSA, EURL ECVAM/JRC, academia, industry and national public health authorities |
|  |  |  |  | Review | Induction |  | (Gómez-Lechón et al., 2010) | Mechanism-based chemical selection for in vitro hepatotoxicity approaches in the LIINTOP project (“Optimisation of liver and intestine in vitro models for pharmacokinetics and pharmacodynamics studies”)  Sodium valproate was selected for steatosis and mitochondrial impairment assays, and as a chemical requiring bioactivation.  Support for inclusion in this chemical selection was based, amongst others, on EURL ECVAM/ICCVAM recommendation, and the RC-ZEBET in vitro/in vivo validation programme (Halle et al., 2003). | Very high confidence and relevance. |
|  |  |  |  | Review on adverse outcome pathways and drugiinduced liver injury testing | not assessed |  | (Vinken, 2015) | Valproic acid is presented as a model steatogenic chemical |  |
|  |  |  |  | Literature review on valproic acid metabolism and its effects on mitochondrial fatty acid oxidation. | Induction (no original research) | Mechanisms inducing hepatotoxicity include: ↓ mitochondrial fatty acid oxidation, ↓ coenzyme A, ↓ carnitine, ↑ oxidative stress, ↓ SCAD, ↓ MCAD, ↑ ACD, ↓ CPTI. | (Silva et al., 2008) | Valproate causes hepatotoxicity in some patients treated for epilepsy, including microvesicular steatosis, necrosis, and hepatic failure. | Moderate relevance (literature review) |
|  |  |  |  | Literature review of acute fatty liver and microvesicular fat diseases in (late) pregnancy | Not specified |  | (Sherlock, 1983) | Onset of acute fatty liver of pregnancy is usually between 30^th^ and 38^th^ week of pregnancy, and in some patients accompanying other symptoms suggest pre-eclampsia. This review focused on physiological changes, and not on adverse drug effects.  Mentions that valproate causes hepatotoxicity, including microvacuolar steatosis. | High confidence, low relevance for valproate-induced steatosis. |
|  |  |  |  | Human, in vivo (epidemiology, prospective cohort study of the Drug Induced Liver Injury Network. 2004-2008 | lipid accumulation not quantified | POLG | (Stewart et al., 2010) | Prospective cohort study investigating associations of mitochondrial DNA polymerase γ (POLG) genetic variations in otherwise healthy individuals with valproate liver toxicity.  POLG genetic substitution variations were associated with an increased risk of valproic acid induced hepatotoxicity. | High confidence, low relevance |
|  |  |  |  |  |  |  |  | Other potentially supportive literature from human in vivo studies reviewed, but not satisfying criteria for detailed listing (e.g., no monotherapy group, or not reporting on hepatic lipid levels): (Turnbull et al., 1986, Li et al., 2019, Rauchenzauner et al., 2008, Meral et al., 2011, Faghihi et al., 2012, Bodin et al., 2001, Malinen et al., 2018, Ellingrod et al., 2012) |  |
| **Caffeine** | 58-08-2 | 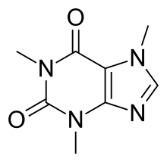 | Pharmaceutical/ natural compound; stimulant | **SUMMARY** | **Negative/ reduction** |  |  | **Moderate-strong evidence, primarily from rodent and human in vivo that caffeine does not induce primary hepatic steatosis**. Several studies report and confirm a protective effect of caffeine to **prevent or ameliorate lipid accumulation** in conditions leading to increased hepatic lipid accumulation (e.g., high-fat diet, or steatosis animal or in vitro models).  Caffeine reduced lipid accumulation in experimental models. In humans, caffeine intake has been associated with a lower risk of NAFLD. Insufficient clinical data is, however, available to conclusively determine the effect of caffeine on NAFLD in humans.  A challenge for finding suitable caffeine references for inclusion was that many studies report effects of coffee rather than caffeine. It is likely that this influences the outcome, as other constituents (e.g., other polyphenols and metabolic breakdown products) may skew the results. |  |
|  |  |  |  | Human, in vitro (HepG2, 0.5-4 mM, 24 h) | Reduction | Gene expression  decreased: SREBP1c/2, FASN, SCD1, HMGR, LDLR,  increased: CD36  Protein levels increased: p-AMPK, p-ACC | (Quan et al., 2013) | Caffeine reduced lipid accumulation including triglycerides.  Changes in gene expression and enzyme phosphorylation were reversible by AMPK inhibition. |  |
|  |  |  |  | Human, in vitro (HepG2, 30-1500 µM, 0.5-24 h).  Rodent, in vitro (3T3-L1 murine preadipocytes, 30-1500 µM, 0.5-24 h (10 d for lipid accumulation)) | Negative |  | (Riedel et al., 2012) | In 3T3-L1 cells, caffeine increased fatty acid uptake, but had no effect on lipid accumulation. Lipid accumulation results are not presented for the HepG2 cells.  In HepG2 cells, caffeine depolarized the inner mitochondrial membrane, increased uncoupling protein 2 (UCP-2) and energy charge potential, and decreased mitochondrial respiration, fatty acid uptake, fatty acid oxidation, and anaerobic lactate production.  Caffeine was cytotoxic to HepG2 cells at 3 mM, therefore this concentration was included for testing of mechanistic endpoints. |  |
|  |  |  |  | Human/rodent, in vitro (*de-facto* co-culture of 3T3-L1 murine adipocytes and human Caco-2 colon epithelial cells, Caco-2: 10 mM, 24 h; 3T3-L1: basolateral conditioned medium, 2 h - 3 d) | Negative/ reduction | In 3T3-L1  Gene expression decreased (3 d exposure): *Pparg, Cebpa, Adipoq*  Protein expression decreased (2 h exposure): C/EBPβ, C/EBPδ (not accompanied by decreased gene expression) | (Mitani et al., 2017) | Incubation of 3T3-L1 cells with medium taken from caffeine-exposed Caco-2 cells reduced lipid accumulation in 3T3-L1 mature adipocytes. Caco-2 cells were differentiated on transwell inserts prior to exposure for 20-22 d, and formation of a tight epithelial barrier was confirmed by transepithelial electric resistance of 1,985±109 Ω*cm^2^. They were then exposed to 10 mM caffeine for 24 h via the apical compartment; caffeine-conditioned medium was then collected from basolateral compartment. Caffeine, but not its metabolites, were detected in basolateral conditioned medium.  While Caco-2-conditioned medium decreased lipid accumulation in 3T3-L1 adipocytes significantly (to ~75%), exposure of 3T3-L1 cells directly to 1-50 µM caffeine did not significantly alter intracellular lipid content. | Moderate confidence and relevance  Interesting relevant exposure scenario |
|  |  |  |  | Rodent, in vivo (C57BL/6 mice, male, n = 5/group, non-HFD diet: 30 mg/kg bw/d, i.p., 24 h or 3 d. HFD diet: 0.05% (w/v) caffeine via drinking water, 4 weeks)  Human, in vitro (ATG5-transfected HepG2, co-exposure to 0.5 mM oleic acid/palmitic acid (2:1) and 1.5 mM caffeine, ~16 h) | Negative/ decrease | Increased autophagy via suppression of mTOR, and markers of fatty acid β-oxidation. | (Sinha et al., 2014) | Animals were fed an HFD or normal (non-HFD) diet.  Caffeine decreased lipid accumulation in the HepG2 cells (at 1.5 mM) and in the HFD mice. | Moderate relevance |
|  |  |  |  | Rodent, in vivo (C57BL/6, 3-week old male mice, n = 8/group; 10-20 mg/kg bw/d, oral gavage, 10 weeks)  Human, in vitro (HepG2 pre-loaded with 1 mM oleate for 2 h; 2 mM caffeine, 24 h) | Negative/ reduction | Activated the cAMP/CREB/SIRT3/AMPK/ACC pathway. | (Zhang et al., 2015) | In mice, caffeine reduced liver fat, lipids, ALT, and AST. In cells, caffeine reduced lipid levels, and this effect was counteracted by inhibiting cAMP, sirtuin 3 (SIRT3), or AMPK activity.  Markers of steatosis (liver triglycerides, liver total cholesterol, plasma ALT, plasma AST) showed similar levels to normal diet for both caffeine doses fed with a high-energy diet. (All were significantly increased on high-energy diet alone) |  |
|  |  |  |  | Rodent, in vivo (CFY, male rats, n = 6-8/group, 2.5 g/kg diet, 7-25 d) | Negative/ decrease |  | (Fears, 1978) | Animals were fed ad lib. Either on a stock pelleted diet, or on a high-cholesterol semi-synthetic diet for 7 days, after which they were allocated to experimental groups.  In rats on a low cholesterol diet, caffeine had no effect on hepatic fatty acid synthesis or triglyceride content. In rats on a high cholesterol + cholic acid diet, caffeine decreased hepatic fatty acid synthesis. In ex vivo liver slices, 2 mM caffeine reduced fatty acid synthesis. |  |
|  |  |  |  | Fish, in vivo (zebrafish larvae (WT) fatty liver model, 1-8% caffeine via diet, duration of caffeine treatment not clear. n= 15 larvae/group) | Negative/ reduction | On gene expression level, caffeine increased lipid β-oxidation (ACO), decreased lipogenesis (SREBP1, ACC1, CD36, UCP2), decreased endoplasmic reticulum stress (PERK, IRE-1, ATF6, BIP), decreased inflammation (IL-1B, TNFa), and autophagy (ATG12, Beclin-1). Protein expression of of CHP, BIP, and IL-1β was confirmed. | (Zheng et al., 2015a) | Zebrafish larvae were fed 20-180 mg/d from 5 dpf; fatty liver incidence increased in the high-feed group significantly (p<0.001) within 20 days.  Initially, 100 larvae/group were raised under different feeding regimen. At 20 dpf, zebrafish larvae were allocated to caffeine dosing or control group à 15 larvae; caffeine exposure group received 1, 2.5, 5, or 8% (w/v) caffeine in 20 mg feed daily.  Caffeine (2.5 to 8%) significantly attenuated hepatic lipid and triglyceride accumulation (decrease to ~70% lipid levels; low-feed control: ~15%), compared to caffeine-free high-feed control. Total cholesterol remained unchanged by feeding regime. | Low confidence, moderate relevance  (use of an overfed/steatosis model, low quality English in publication, and uncertainties about exposure regimen) |
|  |  |  |  | Literature review on the effects of plant derived natural products (including caffeine) on NAFLD. | Negative/ reduction | Increased conversion of ADP to ATP, and activated a cAMP/ CREB/ SIRT3/ AMPK/ ACC pathway. | (Xu et al., 2018) | In high energy diet fed mice, caffeine reduced liver fat, lipids, ALT, and AST. In oleate treated cells, caffeine reduced lipid levels, and this effect was counteracted by inhibiting cAMP, SIRT3, or AMPK activity.  Contains a review of the findings from the Zhang et al. 2015 paper below. |  |
|  |  |  |  | Literature review on the effects of coffee and caffeine in rodent and cell models of liver diseases. | Reduction |  | (Salomone et al., 2017) | Caffeine reduced steatosis in HFD fed mice and in fatty acid treated HepG2 cells. Caffeine increased autophagy. |  |
|  |  |  |  | Human, in vivo (epidemiology, NHANES, n=18,550 included participants) | Negative/ reduction |  | (Birerdinc et al., 2012) | Study analysed data from US National Health and Nutrition Examination Surveys (NHANES 2001-2008) to determine the associations between NAFLD and the intake of different nutrients in humans. Of the initial study population (41,658 participants in NHANES 2001-2008), 18,550 were considered eligible for inclusion in this study. Of those, 1782 (10.41 ± 0.37%) fulfilled the definition of NAFLD, and 16,768 were used as control subjects.  Caffeine intake was found to be associated with a lower risk of NAFLD (p<0.001), but the mechanism by which such potential hepatoprotective effect is established remains to be elucidated. | Very high confidence and relevance  Study representative of US households, cross sectional |
|  |  |  |  | Human, in vivo (pilot randomized placebo-controlled clinical trial, caffeine for the treatment of NAFLD and diabetes mellitus. n=26 patients, 6-7 per intervention group, 200 mg/d, 12 weeks) | Negative |  | (Mansour et al., 2020) | Clinical study evaluating caffeine treatment (200 mg caffeine with or without 200 mg chlorogenic acid) in patients with NAFLD and Diabetes Mellitus (n = 6-7/group) for 12 weeks.  200 mg caffeine were administered either alone, or in combination with chlorogenic acid, placebo (starch).  Caffeine alone had no effect on serum levels of ALT and AST. The study did not include histological or other markers of NAFLD. | Moderate confidence and relevance |
|  |  |  |  |  |  |  |  | Other potentially supportive literature from human in vivo studies reviewed, but not satisfying criteria for detailed listing (e.g., caffeine content not specified, studies with coffee (i.e., a mixture), or not reporting on hepatic lipid levels): (Watanabe et al., 2017, Panchal et al., 2012, Saab et al., 2014, Wijarnpreecha et al., 2017, Shen et al., 2016, Chen et al., 2019, Torres and Harrison, 2013, Marventano et al., 2016, Walton et al., 2013, Veronese et al., 2018, Voerman et al., 2020, Graeter et al., 2015, Hayat et al., 2021, Molloy et al., 2012, Papandreou et al., 2019, Wahrburg et al., 1994, Trovato et al., 2013) |  |
| **Ascorbic acid (vitamin C)** | 50-81-7 (as salt: 134-03-2) | 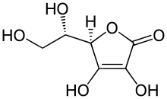 | Vitamin/essential nutrient; dietary supplement | **SUMMARY** | **Negative/ reduction** |  |  | **Strong weight of evidence supporting ascorbic acid/vitamin C not inducing primary hepatic steatosis.** Most evidence evaluated reports on rodent in vivo studies (mouse, rat, guinea pig). Human in vitro and in vivo studies were fewer, but the results are in line with rodent in vivo studies, despite ascorbic acid not being essential for rat and mice.  Most studies reviewed investigated the hepatoprotective effect of ascorbic acid in models of controlled induced hepatic steatosis (chemically or dietary induced). Protection was assessed either by pre-treatment or co-administration of a defined dose of vitamin C (true protection), or reversion of previous steatosis (therapy). In both scenarios, ascorbic acid had no effect, or improved (decreased) lipid accumulation in hepatocytes, and steatosis-associated blood markers (especially serum AST and ALT).  Evidence from human in vivo (epidemiological) data is weaker, but in line with observation from (human) in vitro and rodent studies. A limitation in the assessment of low ascorbic acid intake in humans is that as an essential dietary micronutrient in humans (to prevent e.g. scurvy), it is not ethical to control for very low/no ascorbic acid intake.  One study in zebrafish identified a potentially higher baseline susceptibility of male over female fish for developing (microvesicular) hepatic steatosis in the absence of dietary ascorbic acid. However, steatosis was accompanied with general symptoms of scurvy.  It must be noted, that unlike for humans, vitamin C is not an essential micronutrient for most rodents, including mice and rats (excluding guinea pigs). |  |
|  |  |  |  | Human, in vitro (HepG2, 100-1000 µM + 10 ng/mL TNF-α, 2 4h). | Not assessed |  | (Gu et al., 2019) | Study did not evaluate any steatosis endpoints. Vitamin C (100 µM) reduced TNF-α induced hypoxia, inflammation, and ER stress.  Vitamin C reduced expression of hypoxia-inducible factor 1-alpha (Hif1α), monocyte chemoattractant protein 1 (Mcp1), 78-kDa glucose-regulated protein (Grp78), and increased levels of adiponectin, fibroblast growth factor 21 (Fgf21), and fibroblast growth factor receptor 2 (Fgfr2). Ffgr2 knockdown inhibited some of vitamin C’s effects. |  |
|  |  |  |  | Rodent, in vivo (C57BL/6J mice, male, n = 8/group, 1% (w/w) in high-fat diet, 15 weeks) | Negative/ decreased | Reduced inflammation, fibrosis, apoptosis, serum ALT, serum AST, increased gene expression for fatty acid oxidation | (Lee et al., 2019a) | Vitamin C reduced hepatic steatosis (lipids and triglycerides), compared to control on high-fat diet without vitamin C.  Vitamin C reduced hepatic inflammation (TNFα and MCP-1), fibrosis [transforming growth factor β (TGFβ), alpha smooth muscle actin (α-SMA), Collagen α1], apoptosis [↑ B-cell lymphoma 2 (Bcl2), ↓ Caspase 8], and serum ALT and AST levels, and increased hepatic mRNA for fatty acid β-oxidation proteins [peroxisome proliferator activated receptor alpha (PPARα), acyl-coenzyme A oxidase (Acox), carnitine palmitoyltransferase I (Cpt-1), very long-chain acyl-CoA dehydrogenase (Vlcad)]. |  |
|  |  |  |  | Rodent, in vivo (C57BL/6 male mice, n = 6/group, 1- 90 mg/kg bw/d administered intragastrically. Two exposure scenarios (details on the right)). | Negative/ reduction |  | (Zeng et al., 2020) | Two exposure scenarios were compared: prophylaxis: animals on a normal (i.e., not high-fat) diet were receiving vitamin C 12 weeks therapy: animals on a high-fat diet for 6 weeks were administered vitamin C for twelve weeks (steatosis was confirmed in two sample animals before initiation of vitamin C exposure)  Prophylactic treatment with low and medium but not high vitamin C doses reduced hepatic steatosis. Therapeutic treatment with medium but not high or low doses also reduced steatosis. |  |
|  |  |  |  | Rodent, in vivo (C57BL/6 male mice, n = 20-24/group, 1.5 g/L via drinking water for 11 weeks) | Negative/ decrease | In HFD fed mice, the following hepatic alterations were observed in the KO vs. WT mice: increased Chrebp, Srebp-1c, and decreased Fasn, and these alterations were counteracted by vitamin C supplementation. | (Lee et al., 2021) | Vitamin C deficient senescence marker protein 30 (SMP30) KO and WT mice (male, C57BL/6, n = 20 or 24/group, respectively) fed a HFD and exposed to vitamin C (1.5 g/L) in the drinking water for 11 weeks. Mouse WT and SMP30 KO primary hepatocytes challenged with high sugar and cholesterol and treated with vitamin C (75 µM every 8h for 24h). SMP30 is an enzyme in the vitamin C biosynthetic pathway in mice.  In WT mice, vitamin C had no effect on HFD-induced hepatic steatosis or inflammation, but it reduced ballooning. In the KO mice, HFD induced steatosis, inflammation and ballooning was decreased, and Vitamin C supplementation counteracted these effects. In hepatocytes treated with high sugar and cholesterol, culturing without vitamin C decreased triglyceride levels. | High confidence, low relevance |
|  |  |  |  | Rodent, in vivo ( *Tet1^tm1.1Jae/J^* C57BL/6 transgenic male mice, n = 3 – 9/group. High-fat diet + Vit.C, ~0.36 g/kg bw/d via drinking water, 6-7 weeks)  Rodent, in vitro (primary hepatocytes with/without transfection of m*Tet1*, co-exposure to vehicle, 400 µM palmitate, or 200 µM oleate; 200 µM Vit.C, 24-48 h) | Negative/ decrease | Tet1 | (Yuan et al., 2021) | Transgenic DNA demethylase ten-eleven translocation protein 1 (Tet1) WT, homozygous knockout, and haplo-insufficient mice (C57BL/6, males, n = 3 – 9/group) fed a HFD from 4 weeks-old and treated with vitamin C (~0.36 g/kg/day; starting from age of 9 weeks/5 weeks post commence of HFD) provided in the drinking water for 6-7 weeks.  Vitamin C counteracted the increased hepatic lipid and triglyceride accumulation associated with Tet1 insufficiency in fatty acid treated cells and HFD fed mice.  Vitamin C counteracted the following HFD/fatty acid induced hepatic alterations in the Tet1 insufficient models: increased DNA methylation, decreased mRNA for hormone-sensitive lipase (Hsl), Ppar-α, Cpt1a, Acox1, Pgc1a, and increased mRNA for Srebf1, Fasn, Acly, Acc, Cd36, and solute carrier family 27 member 1 (slc27a1). | Moderate confidence, low relevance |
|  |  |  |  | Rodent, in vivo (Wistar rats, male, n = 6/group, 30 mg/kg bw/d, oral, 4 weeks) | Negative/ reduction |  | (Oliveira et al., 2003) | Rat choline deficient diet model of fatty liver (male Wistar rats, n = 6/group) treated with vitamin C (30 mg/kg/day, orally) for 4 weeks.  Vitamin C is said to have reduced hepatic steatosis, but the data presented is not very convincing. Only an insufficient selection of representative images are provided as histological results. | Low confidence, moderate-ow relevance |
|  |  |  |  | Rodent, in vivo (Wistar rats, male, n = 7/group, co-exposure to emamectin benzoate (hepatotoxicity inducer; 10 mg/kg bw/d) and 200 mg Vit.C/kg,bw/d, intraperitoneally, 28 d) | Negative/ decrease |  | (Khaldoun Oularbi et al., 2017) | Rat emamectin benzoate (EMB) induced liver toxicity model (male Wistar, n = 7/group) treated with vitamin C [200 mg/kg, intraperitoneally (i.p)] for 28 days.  It is said that vitamin C reduced histological markers of liver toxicity, but the presented data is not very convincing. No quantitative histological data is presented, and an insufficient selection of representative images are provided. | High confidence but low relevance.  Test following OECD TG 407 for repeated dose 28-day oral toxicity studies in rodents. |
|  |  |  |  | Rodent, in vivo (Hartley guinea pigs, male, n = 6/group, high-palm oil diet, 200 or 600 mg/kg bw/d via drinking water, 18 weeks)  Human, in vitro (HepG2 steatosis model (0.01-0.7 mM palmitate for 12 h), 0.16-3.1 mM) | Negative/ decrease | SOC3Improved insulin sensitivity. | (Xu et al., 2020b) | Male guinea pigs (Hartley, n = 6/group) fed a high palm oil diet treated with vitamin C (~200 or ~600 mg/kg/day) provided in the drinking water for up to 18 weeks. Palmitic acid challenged HepG2 cells treated with vitamin C (0.16 to 3.1 mM).  The anti-steatotic effects of vitamin C were abolished by SOC3 overexpression. Vitamin C improved insulin sensitivity.  Vitamin C reduced steatosis (triglyceride and lipids) in the guinea pig (at 200 and 600 mg/kg/day) and the cell (at 1.6 and 2.17 mM) models.  Palmitate (co-)exposure or pre-loading not specified for HepG2 experiment or results; palmitate concentration for co-exposure with ascorbic acid not specified. | Moderate-low confidence, moderate relevance |
|  |  |  |  | Rodent, in vivo (guinea pigs, male, n = 6/group, steatohepatitis model, 2.5 g/kg bw/d orally, 3 weeks) | Negative/ reduction |  | (Park et al., 2018) | Steatohepattis was induced by methionine-choline-deficient diet.  Avery high dose of Vitamin C was administered and is said to have reduced steatosis.  Similar to humans, guinea pigs require dietary sources of vitamin C and (unlike other rodent, including mice and rats) cannot synthesize vitamin C in their digestive tract. | Low confidence, low relevance  No quantitative liver histology data is provided, only 4 insufficient blurred representative images |
|  |  |  |  | Rodent, in vivo (guinea pig, male, n = 6/group; 0.25 g/kg bw/d, 30 d) | Negative/ decrease | Increased liver levels of reduced glutathione (GSH), GSH peroxidase (GPX), GSH reductase (GR), and GSH transferase (GST). | (Abhilash et al., 2012) | Vitamin C reduced liver levels of triglycerides, steatosis, and inflammation, and serum levels of ALT, AST, and gamma glutamyl transpeptidase (GGT). |  |
|  |  |  |  | Fish, in vivo (zebrafish, male and female, n = 14/group, 200 mg/kg diet, 12 weeks) | Negative/ reduction (higher basal steatosis susceptibility in male) | In both males and females, the Vitamin C free diet depleted hepatic glycogen and induced smooth ER. | (Phromkunthong et al., 1994) | On a vitamin C free diet, fish developed symptoms of scurvy, including hemorrhages, fin erosion, and darker pigmentation. Male but not female fish developed hepatic steatosis, while females did not show microvesicular lipid accumulation within the cisternae of the endoplasmic reticulum. | High confidence, moderate relevance.  Indication of sex differences in susceptibility to steatosis in absence of ascorbic acid. |
|  |  |  |  | Human, in vivo (cross-sectional epidemiology, n = 789) | Negative/ decrease |  | (Ivancovsky-Wajcman et al., 2019) | Steatosis was assessed by ultrasonography.  789 participants (age 58.83±6.58 years), of which 52.6% were male. 714 participants passed inclusion criteria for evaluation (reliable FibroMax value).  Higher vitamin C intake (upper tertile vitamin C/ 1000 kcal) was associated with lower odds of NAFLD and NASH (OR = 0.68, 0.47–0.99, P = 0.045; OR = 0.57, 0.38–0.84, P = 0.004, respectively). |  |
|  |  |  |  | Human, in vivo (prospective cross-sectional epidemiology. n = 149 children) | Negative |  | (Vos et al., 2012) | Participants were enrolled in the multi-center NASH Clinical Research Network.  Relatively low vitamin C intake (72.6 mg vs. 106.9-139.9 mg) was associated with increased hepatic ballooning (n=30). There was no association between Vitamin C intake and steatosis. |  |
|  |  |  |  | Review evaluating the effects of vitamin C on fatty liver in animal models and in humans. | Negative/ (decrease) |  | (Ipsen et al., 2014) | In animal models, vitamin C deficiency is associated with increased hepatic lipids, oxidative stress, fibrosis and inflammation, whereas supplementation with Vitamin C reduces hepatic steatosis, ballooning, oxidative stress and inflammation. Some human studies have shown beneficial effects of Vitamin C on NAFLD, but overall, the results of human studies are conflicting/inconclusive. |  |
|  |  |  |  | Review on vitamins in the treatment of NAFLD in rodents and humans | Negative/ decrease | Vitamin C inhibits hepatic steatosis via an increase in PPAR-α dependent fatty acid β-oxidation genes and attenuates cell stress by activating a FGF21/FGFR2/adiponectin pathway. | (Raza et al., 2021) | Vitamin C reduced lipid accumulation in the hepatocytes of guinea pigs, and improved oxidative stress, hepatocellular ballooning and inflammation in a rat model. In some clinical studies, low vitamin C levels has been associated with NAFLD. |  |
|  |  |  |  | Review (cross-sectional) of the effects of nutrients on NAFLD in humans | Negative |  | (Yki-Järvinen, 2010) | Intake of vitamin C and E was found to be lower in NAFLD in one study. |  |
|  |  |  |  | Review on the role of nutrients in NAFLD. | Negative |  | (Mouzaki and Allard, 2012) | In one clinical study, NASH was associated with low intake of vitamin C and E. |  |
|  |  |  |  |  |  |  |  | Other potentially supportive literature from human in vivo studies reviewed, but not satisfying criteria for detailed listing (e.g., lack of characterization of exposure, or exposure to a mixture only, or not reporting on hepatic lipid levels): (Rives et al., 2020, Ued and Weffort, 2013, Musso et al., 2010, Sawangjit et al., 2016, Harrison et al., 2003, Ersöz et al., 2005, Alberti et al., 2020, Foster et al., 2011, Singh et al., 2020, Depner et al., 2013b, Nobili et al., 2008) |  |
| **Niacin (nicotinic acid; vitamin B_3_)** | 59-67-6 | 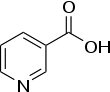 | Essential human nutrient; pharmaceutical (dyslipidaemia) | **SUMMARY** | **Negative/ reduction** |  |  | **Moderate-strong evidence** from in vitro (human and rodent), and rodent in vivo studies supporting **no induction of primary hepatic steatosis**. Few studies investigated niacin effects on lipid accumulation in “healthy lean” models, i.e., in animals not fed a high-fat diet or cell-culture models without additional supply of fatty acids, in some form. In models favoring lipid accumulation and/or obesity, niacin reduced body weight gain, improved serum lipid markers, and prevented hepatic lipid accumulation.  **Human in vivo evidence is less conclusive** (**moderate-weak weight of evidence**). On one hand, niacin is being prescribed for the treatment of some types of hyperlipidemia, on the other hand, adverse side effects of hepatotoxicity, including elevated blood liver enzyme markers, and (usually local) fatty infiltrations of the liver are described. Adverse hepatic side effects are more likely with prolonged intake of higher doses of niacin (≥3 g/d); a higher incidence of niacin-induced hepatotoxicity was reported with the no longer authorized form of “sustained release” niacin (currently, niacin is marketed as “extended release” formulation, which is between the “immediate release”/crystalline form and the “sustained exposure” formulation).  In some patients, nicotinic acid causes hepatotoxicity, including local fatty infiltration of the liver, (microvesicular) steatosis, increased serum levels of AST, ALT, and ALP, fibrosis, inflammation, and necrosis.  The detailed literature review of human in vivo studies summarized below might give the false impression of niacin acting as an inducer of hepatic steatosis in vivo, but the continued marketing authorization for the treatment of dyslipidemia since the 1950s, and the rather small number of adverse effect drug reports indicates that hepatotoxicity remains a side effect and is not a major mechanism of disease.  However, the potential of niacin inducing or contributing to the development of steatosis in vivo, which was not observed in rodents or in in vitro models, makes this chemical a less suitable candidate for proficiency testing of steatosis test methods.  It has also been noted that reports of induction of microvesicular steatosis in humans may be due to secondary effect and not by primary action on the liver. |  |
|  |  |  |  | Human, in vitro (Human primary hepatocytes or HepG2, pre-loaded with BSA-complexed 0.5 mM palmitate for 24 h, 0.25-0.5 mM, 24 h) | Negative/ decrease | Decreased ROS, NADPH oxidase activity, and IL-8 levels.  Gene expression  decreased: DGAT2,  unaltered: FAS, CPT1 | (Ganji et al., 2015) | Nicotinic acid at 0.25 and 0.5 mM decreased fat accumulation in the cells. |  |
|  |  |  |  | Human and rodent, in vitro (HuH-7 (male hepatoma) or primary mouse hepatocytes, 3 mM, 16-72 h)  Human, in vivo (randomized cross-over clinical study, n=20 non-diabetic dislipidaemic men; 2 g/d, 8 weeks) | Decrease |  | (Blond et al., 2014) | Nicotinic acid decreased intrahepatic triglyceride levels (alongside increase in diacylglycerols) in vitro, along with reduced insulin action independent of PKCε. (DGAT2 expression not changed)  In vivo, niacin improved the serum lipid profile (-28% triglycerides, +17 HDL-C, unchanged fasting non-esterified fatty acids). Niacin administration induced hepatic insulin resistance (reduced inhibition of endogenous glucose production by insulin, decrease in fasting hepatic insulin sensitivity index) |  |
|  |  |  |  | Rodent, in vivo (*APOA*-transgenic mice, n=6 female/group, 10 g niacin/kg chow diet, 2 weeks)  Human and rodent, in vitro (HepG2, primary human hepatocytes, primary *APOA*-transgenic mouse hepatocytes, (50-200 µM, 24-36 h) | Not assessed | Reduced hepatic cAMP; decreased *APOA* expression(50-200 µM) | (Chennamsetty et al., 2012) | Primary human hepatocytes were isolated from non-neoplastic tissue samples from patients undergoing partial hepatectomy for metastatic liver tumours of colorectal cancer.  The study did not include any liver hepatotoxicity endpoints. |  |
|  |  |  |  | Rodent, in vivo ( C57BL/6 WT and *Gpr109a^-/-^* male mice, n = 6-10/group, high-fat diet, 50 mM via drinking water, 8-9 weeks)  Human, in vitro (HepG2 pre-loaded with oleic acid, 300 µM, 24 h) | Negative/ reduction | *Gpr109a* ameliorated lipid accumulation | (Ye et al., 2020) | Prior to niacin exposure, mice were left on high-fat diet (60% of calories from fat) for 6 weeks, to induce obesity.  In WT mice, nicotinic acid reduced hepatic steatosis and TNF-α and serum AST and ALT (ALP unchanged).  In *Gpr109a^-/-^* mice, lipid accumulation in the liver was higher than in vehicle-treated WT control, and niacin did not decrease/change the lipid accumulation. While knock-out mice showed the highest gain on body weight over the experiment duration, independent on exposure to niacin, niacin significantly reduced bodyweight gain in WT mice (significant from week 2 post exposure). A comparable pattern was observed for total fat mass, and total liver weight, which were significantly decreased for niacin-treated WT mice.  Analysis of pro- and inti-inflammatory cytokines indicates a decrease of pro-inflammatory cytokines (non-significant, except for *Tnf-α*) and increase in anti-inflammatory cytokines (non-significant) in WT mice; no clear trent and no significant changes are reported for the knock-out model. |  |
|  |  |  |  | Literature review on the therapeutic and adverse effects of nicotinic acid | Negative/ decrease |  | (Capuzzi et al., 2000) | In humans, nicotinic acid is associated with hepatotoxicity (incl. raised plasma AST and ALT, cholestatic jaundice, and fulminant acute hepatitis). Therapeutic effects of nicotinic acid include a reduction in plasma cholesterol, triglycerides, and low-density lipoprotein (LDL), and increased high density lipoprotein (HDL).  Nicotinic acid reduces triacylglycerol formation in the liver, increases lipoprotein lipase activity, and inhibits the synthesis and secretion of apo B-100 and hepatic very low-density lipoprotein (VLDL). |  |
|  |  |  |  | Human, in vivo (randomized placebo-controlled clinical trial, n=25,673 patients at high risk of (cardio)vascular events; 2 g/d extended-release niacin + 40 mg/d laropiprant, ~1 month-4 years) | Not assessed, but negative/ reduction inferred (from niacin improving blood lipid profile and markers) |  | (HPS2-THRIVE Collaborative Group, 2013) | Niacin has potentially favourable effects on lipids, but its effect on cardiovascular outcomes is uncertain. HPS2THRIVE is a large, randomized trial assessing the effects of extended-release niacin in patients at high risk of vascular events.  Study sites were established in China (72 hospitals or clinics), UK (89), Denmark (22), Finland (10), Norway (21), and Sweden (31). A total of 51,698 patients attended the study screening clinics: 16,861 in China, 24,396 in the UK, and 10,441 in Scandinavia; and 97, 66, and 95%, respectively, entered the run-in trail phase. Of the 25,673 patients listed for randomization, 12,838 were allocated to the extended-release niacin/laropiprant group, 12,835 to placebo.  Niacin is prescribed for its anti-dyslipidaemic effects. It is/was authorised for medicinal use in the EU in combination with laropiprant, that has no effect on cholesterol levels, but ameliorates skin flushing, which is a frequently occurring side effect of niacin administration.  The risk of myopathy was increased by adding extended release niacin/laropiprant to simvastatin 40 mg daily (with or without ezetimibe), particularly in Chinese patients whose myopathy rates on simvastatin were higher. Despite the side effects of ERN/LRPT, among individuals who were able to tolerate it for ~1 month, three-quarters continued to take it for ~4years. | Very high confidence and relevance.  Large cohort |
|  |  |  |  | Human, in vivo (randomized placebo-controlled trail, n=27 (9 per group) obese patients with NAFLD, 2 g/d, 16 weeks) | Negative |  | (Fabbrini et al., 2010) | Nicotinic acid had no effect on levels of intrahepatic triglyceride.  Niacin decreased plasma triglyceride, very low-density lipoprotein-triglyceride (via decreased secretion), and very low-density lipoprotein-apolipoprotein b concentrations. |  |
|  |  |  |  | Human ,in vivo (clinical adverse event case report, n=4 cases of niacin-induced hepatotoxicity) | Inconclusive. Some indication for niacin-induced hepatotoxicity, including fatty infiltration of the liver |  | (Coppola et al., 1994) | In 4 patients, nicotinic acid was associated with hepatotoxicity, including local fatty infiltration (first report of such pathology ever described), and increased serum AST, ALT, and ALP levels. All patients reported current or recent past use of various other pharmaceuticals.  One patient presented with mild focal intracellular cholestasis and fatty liver a second patient presented with a 1-cm low-density lesion in the right lobe of the liver. Both cases were later attributed to niacin as fatty infiltrations of the liver induced by niacin, as they resolved after discontinuing niacin.  Overall, the article confirms and supports the prescription of niacin for certain types of dyslipidaemia but warrants that non-specific liver symptoms or elevated liver enzymes in the blood should trigger questioning about the use of niacin. | Moderate-low confidence, high relevance  Small cohort |
|  |  |  |  | Human, in vivo (clinical adverse event case report, n=1 52 year old male, 2 g/d, 5 months) | Induction |  | (Kristensen and Olcott, 1999) | In 1 patient with underlying health issueas and co-mediaction, nicotinic acid was associated with hepatotoxicity, including raised plasma ALT, AST, and ALP, and abnormal liver imaging results. The patient died on day 5 after admission to hospital.  While palpative abdominal anamnesis remained uncertain, biochemical indicators from blood initially indicated cholestasis and/or intrahepatic tumor (fatty infiltration of the liver), with no biliary obstruction.  After discontinuation of niacin, blood biochemistry returned to normal range within 4 days; hepatic lesions improved within 1 month and resolved within 9 months. | Moderate-low confidence, high relevance  Single case study |
|  |  |  |  | Human, in vivo (clinical adverse effect case report, n=1, 74 year old female, 500 mg niacin three times daily) | Induction |  | Leung et. Al. 2018 | In 1 patient, extended-release nicotinic acid caused hepatotoxicity, including raised serum ALP, AST, and ALT, and microvesicular steatosis. Also summarized findings from several other published reports in which nicotinic acid induced hepatotoxic effects, including fibrosis, inflammation, and necrosis. | Moderate-low confidence, moderate relevance  Single case study |
|  |  |  |  | Human, in vivo (clinical adverse event case report, n=1 37 year old female, 1-4 g/d, 7 months) | Induction |  | (Lawrence, 1993) | Symptom onset was 3 months after initiation of niacin therapy; 7 months after therapy began computed tomography was recorded, and identified two hypodense, well defined central defects in the right lobe of the liver. 2 months after discontinuation of niacin hepatic symptoms were improved and resolved within 6 months of discontinuation.  In 1 patient, nicotinic acid was associated with hepatotoxicity, including raised serum ALT, AST, ALP, and abnormal liver imaging results indicative of focal fatty infiltration.  It is mentioned that niacin-induced hepatic dysfunction was initially thought to occur at dosages of ≥3 g/d after several weeks of exposure. However, sustained-release formulations might induce such defects in a shorter time frame, i.e., within days of therapy onset, and at dosages as low as 500 mg/d. | Moderate-low confidence, high relevance  Single case study |
|  |  |  | Nicotin amide (amide of niacin) | Human and rodent, in vitro (HepG2 or AML-12 hepatocytes; 0.1 to 1 mM, 1 h; challenged post-exposure with BSA- conjugated 0.5 mM palmitate, 16 h) | Not assessed; inferred: negative/ non-inducer | SIRT1 | (Shen et al., 2017) | AML-12 (alpha mouse liver-12) hepatocyte culture was established from a mouse transgenic for human transforming growth factor α (TGFα). Both cell lines were transfected with Sirt1 siRNA to study RNA interference.  Nicotinamide increased autophagy, and SIRT1. Inhibition of autophagy or SIRT1 counteracted nicotinamide’s effects.  Did not include any measures of lipid accumulation. Nicotinamide at 0.1 to 1 mM protected vs. palmitate induced cell death. | Moderate-low relevance |
| **Acetaminophen (Paracetamol)** | 103-90-2 | 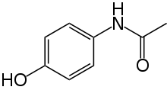 | Pharmaceutical (non-steroidal anti-inflammatory drug) | **SUMMARY** | **Negative** | **CYP2E1**, CYP3A4 |  | **Strong weight of evidence supporting acetaminophen not inducing primary hepatic steatosis, at non-cytotoxic levels**. This is supported by data from rodent in vivo, rodent and human in vitro, as well as human in vivo epidemiological studies.  In most studies in experimental models and humans, acetaminophen is not associated with steatosis. However, at high doses/concentrations acetaminophen does induce hepatotoxicity (drug-induced liver injury, DILI), including hepatic apoptosis, necrosis, neutrophil infiltration, and increased serum ALT and AST. In contrast to inducing steatosis, underlying steatosis/ fatty liver is a risk factor for developing DILI following acetaminophen treatment.  A possible explanation for increased risk of DILI in individuals with steatosis could be a higher activity of CYP2E1, and consequently a disbalance in acetaminophen metabolism into non-toxic metabolites or the reactive toxic metabolite NAPQI (via CYP2E1 and/or CYP3A4). |  |
|  |  |  |  | Human, in vitro (differentiated HepaRG cells, 72 h, 1.5×10^-8^ – 1×10^-3^ M) | (not concluded) | Oxidative stress | (Landesmann et al., 2012) | ROS in duction (60 µM) and decreased cell count (200 µM) |  |
|  |  |  |  | Human, in vitro (“Chang liver” (HeLa, see comment on right) cells, WT and FABP1-transfected, 0.5 to 20 mM, 3-24 h) | Not assessed | Increased ROS and Bax expression | (Gong et al., 2014) | Acetaminophen 0.5 to 20 mM decreased cell viability, and FABP1 overexpression reduced this effect. | Moderate-low confidence and relevance  Problematic cell line: Contaminated. Shown to be a HeLa derivative (Gao et al., 2011, Capes-Davis et al., 2010, Nelson-Rees and Flandermeyer, 1976, Gartler, 1968). Originally thought to originate from normal liver hepatic cells. |
|  |  |  |  | Human, in vitro (“HL-7702” (HeLa, see comment on right) cells, 20 mM, 6 h) | Not assessed |  | (Zhou et al., 2017) | Included no lipid measurements. Acetaminophen reduced cell viability. | Moderate-low confidence and relevance  Problematic cell line: Contaminated. Shown to be a HeLa derivative (Ye et al., 2015). Originally thought to originate from a normal fetal liver. |
|  |  |  |  | Human, in vitro (primary liver biopsy tissue, 0.025 to 10 mM, incubation time not specified) | Not assessed |  | (Chrøis et al., 2020) | Study focused on measurement of mitochondrial function in human primary liver biopsy tissue. Included no lipid measurements. Acetaminophen (2-10 mM) reduced mitochondrial state 3 respiration. |  |
|  |  |  |  | Human, in vitro (HepaRG, normal vs steatosis model (100 µM stearic or oleic acid, and/or 0.01-5 µg/mL insulin), 2.5-20 mM acetaminophen, 6 h - 7 d) | Not assessed/ reported (inferred: negative) | CYP2E1, CYP3A4, (oxidative) stress, mitochondrial impairment | (Michaut et al., 2016) | Acetaminophen reduced ATP and GSH levels, and increased mRNA of nuclear respiratory factor 2 (NRF2), heme oxygenase 1 (HMOX1), tribbles pseudokinase 3 (TRIB3), 70 kDa heat shock protein (HSP70), and acetaminophen protein adducts. AMPK activators prevented acetaminophen induced ATP depletion. The loss of ATP and GSH was greater in the presence of stearic acid.  The effect of acetaminophen on measures of steatosis or cell viability was not determined.  Incubation with 100 µM of either fatty acid significantly increased intracellular lipid accumulation. Insulin significantly reduced that accumulated lipid content and increased aboB levels in the medium. This suggests that the insulin-mediated decrease in intracellular lipids might be driven by increased VLDL triglyceride excretion.  CYP2E1 activity was increased by stearic, but not oleic acid. CYP3A4 expression was significantly decreased by stearic acid, to a lesser extent by oleate.  Acetaminophen depleted intracellular ATP stores; this was exacerbated in the steatosis models. | High confidence, moderate relevance |
|  |  |  |  | Human, in vitro (HepG2/C3A cells, 1 mM, 72 h) | Not assessed (inferred: negative) | VDR/RXR activation, CYP450 metabolism, hormone receptor-regulated cholesterol metabolism, p53 signalling, G2/M and G1/S cell cycle transition, AhR signalling | (Prot et al., 2011) | Cells were cultured on microfluidic biochips or 12-well plates. The study evaluated the transcriptomic and proteomic effects of acetaminophen, and included no lipid measurements. Acetaminophen inhibited cell growth.  Acetaminophen modulated pathways involved in cell morphology/movement, DNA repair, cell cycle, lipid metabolism, | High confidence, moderate-low relevance |
|  |  |  |  | Human, in vitro (HepG2/C3A cells, 1 mM, 72 h) | Not assessed (inferred: negative) | Lipid metabolism and peroxidation (p=9.9E-4; induction of the genes FAAH, PLA2G15, PPARD, DEGS1, FADS1, ACSBG1, ACSL1, AQP7, PASK, SMPD1, GPX2, GPX3, and the production of *lta4h* protein, calcium homeostasis via **VDR/RXR** activation pathway (p=8E-2; gene expression of NCOR2, HSD17B2, NC0A1, HES1, PPARD. Proteomics: *annexin A7, visinin, S100P*) DNA damage (p=4.4E-4), cell cycle arrest (p=1E-5), cell death (p=9.9E-4; apoptosis and necrosis), reorganisation of the cytoskeleton on protein level via *coronin, actin, keratin, tubulin* perturbation, and GSH metabolism. | (Prot et al., 2012) | Study focusing on transcriptomic, proteomic, and metabolomic (in medium) changes.  Acetaminophen modulated pathways involved in lipid metabolism and peroxidation, calcium homeostasis, DNA damage, cell cycle arrest, apoptosis, necrosis, reorganization of the cytoskeleton, and GSH metabolism.  Microfluidic culture conditions by themselves may induce stress, activating PXR/RXR, Nrf2, PPAR(gamma), and energy demand (INS, INSR) pathways.  The toxic acetaminophen metabolite N-acetyl-p-benzoquinone imine (NAPQI; detected as excreted GSH conjugate: GSH-APAP) was detected in the microfluidic culture medium, but not in the stationary plate culture. | High confidence, moderate-high relevance |
|  |  |  |  | Interspecies transcriptomics comparison:  Fish, in vivo (zebrafish embryo, 72 hpf, hatched, n=5/group, 73.3–660 µM, 48 h)  Rodent, in vivo (C57BL/6JIco, male mice; single dose of 168.8–300 mg/kg bw, 1-11 d)  Rodent, in vivo (from TG-GATE database; rat;  single dose of 300-1000 mg/kg bw, 3-24 h, OR: repeated dose 300-1000 mg/kg bw/d, 4-29 d)  Rodent, in vitro (from TG-TAGE database; rat primary hepatocytes, 1-10 mM, 2-24 h)  Human, in vitro (from TG-GATE database; primary human hepatocytes, 0.2-5 mM, 2-24 h) | Not assessed/ reported | **FXR**, CFTR, | (Driessen et al., 2015) | Acetaminophen induced many gene expression changes in the different models. There was concordance in the changes between the models at a pathway but not a single gene level. The pathway regulation of metabolism – bile acids regulation of glucose and lipid metabolism via FXR was affected across all models.  Acetaminophen exposure specifically upregulated CFTR in vivo. This transmembrane receptor is expressed in Kupffer cells, which explains the lack of alteration in vitro.  In summary, in vivo models shared 14 differentially expressed genes, in vitro models shared 11 differentially expressed genes.  The publication contains only transcriptomics data, and no steatosis or other hepatotoxicity results. |  |
|  |  |  |  | Rodent, in vitro (Wistar rat primary hepatocytes isolated from normal and steatotic livers, 1-5 mM, 24 h) | Not assessed | Oxidative stress, inflammation (IL-6) | (Kučera et al., 2012) | Prior to sacrifice and isolation of hepatocytes, Wistar rats were fed a normal (10% energy from fat) or high-fat (60% energy from fat) diet for 6 weeks.  Acetaminophen at 1 to 5 mM produced greater cytotoxicity in steatotic hepatocytes compared to normal hepatocytes.  Steatotic hepatocytes contained lower amounts of GSH compared to normal hepatocytes. In the steatotic hepatocytes, acetaminophen induced a more pronounced decrease in GSH and increase in ROS. Acetaminophen increased IL-6 to a similar degree in both steatotic and normal hepatocytes. |  |
|  |  |  |  | Rodent, in vitro (primary hepatocytes isolated from WT and transgenic mice (C57B1/6 background), 0.5-4 mM, 8-24 h) | Not assessed/ reported | Mitochondrial stress, oxidative stress | (Mezera et al., 2015) | Transgenic mice had the endogenous ApoE gene replaced by either human ApoE3 or human ApoE4.  Included no lipid measurements. In WT hepatocytes, acetaminophen at 1 and 4 mM induced cytotoxicity, and there were trends in measures of cytotoxicity being higher in the APOE3 and APOE4 hepatocytes compared to WT, but in most cases differences did not reach statistical significance.  Acetaminophen reduced mitochondrial membrane potential to a similar extent in the WT and APOE3 and 4 hepatocytes. Acetaminophen induced a significant increase in ROS production in APOE3 and APOE4 hepatocytes but not in WT hepatocytes. Acetaminophen significantly reduced GSH levels in the APOE4 but not in the WT and APOE3 hepatocytes. | Low relevance |
|  |  |  |  | Rodent, in vivo ( TAMH cells (“TGF-alpha overexpressing mouse hepatocytes”), 0.5-5 mM, 24-72 h) | Negative/ not assessed (inferred: induction of cell death/ necrosis) |  | (Pierce et al., 2002) | To initiate apoptosis, cells were pre-treated with 200 nM actinomycin D (30 min), followed by 20 ng-mL TNFα.  Acetaminophen increased multiorganellar collapse, DNA fragmentation, chromatin margination, ATF3, growth arrest and DNA damage-inducible gene 153 (GADD153), and mitochondrial proliferation, and decreased proteosomal activity and caspase-3 processing.  Included no lipid measurements. Acetaminophen at 0.5 to 5 mM reduced cell viability, and induced necrosis. |  |
|  |  |  |  | Rodent, in vivo (WT and *PDK4^-/-^*C57BL/6J, male and female mice, n = 3-6/group, 300 mg/kg bw, i.p., for males, 600 mg/kg bw, i.p., for females, 2-24 h)  Rodent, in vitro (primary hepatocytes isolated from animals used in this study, 5 mM, 3-6 h) | Hepatotoxicity. (Steatosis not specified and not assessed) | Acetaminophen increased hepatic p-JNK, Aif, metallothionein, Hmox-1, nitrotyrosine levels, superoxide, and decreased GSH and mitochondrial membrane potential; and the magnitude of most of the latter effects were reduced by PDK4 deficiency. | (Duan et al., 2020) | Included no lipid measurements. Acetaminophen induced hepatotoxicity (necrosis, apoptosis, increased serum ALT), and the toxic effects were reduced in PDK4 deficient mice. In the hepatocytes, acetaminophen reduced viability, and PDK4 deficiency had a protective effect. | Low relevance |
|  |  |  |  | Rodent, in vivo (WT and transgenic C57BL/6J, male mice, n = 4-16/group, 300 mg/kg bw, i.p., 2-24 h) | Not assessed | Acetaminophen decreased liver GSH. | (Torres et al., 2019) | Transgenic variants: liver specific steroidogenic acute regulatory protein 1 (STARD1), SH3 domain-binding protein 5 (SAB), or JNK1+2 KO mice.  Included no lipid measurements. Acetaminophen induced hepatotoxicity (necrosis, and increased serum ALT and AST) in WT mice. The STRARD1, SAB, and JNK1+2 knockout mice strains were resistant against acetaminophen toxicity.  Administration of valproic acid prior to acetaminophen increased hepatotoxicity. | Low relevance |
|  |  |  |  | Rodent, in vivo (WT and *Fgf21^-/-^-*C57BL/6, male mice, n = 3 -5/group, 300 mg/kg bw, i.p., 4-24 h) |  | Hepatotoxicity, but steatosis not specified | (Vispute et al., 2017) | Included no lipid measurements. Acetaminophen induced liver toxicity in mice (necrosis, and increased serum ALT and AST), and dexamethasone ameliorated acetaminophen toxicity in WT but not Fgf21-null mice. | Low relevance |
|  |  |  |  | Rodent, in vivo (WT and miR-155 KO C57BL/6J, male mice, n = 5-6/group,300 mg/kg bw i.p., 24 h) | Not assessed/ reported | miR-155, inflammation | (Yuan et al., 2016) | Acetaminophen increased hepatic miR-155 in WT mice. Acetaminophen also increased hepatic Mcp1, Tnfα, IL-1β, and IL-6, and the latter effects were enhanced in the miR155 KO mice. miR-155 agomir prevented acetaminophen induced hepatotoxicity.  Included no lipid measurements. Acetaminophen induced hepatoxicity (incl. apoptosis, necrosis, neutrophil infiltration, and increased serum ALT and AST) in mice, and the toxicity was enhanced in miR-155 KO mice. |  |
|  |  |  |  | Rodent, in vivo (WT or *β2sp^+-+^* C57BL/6, male mice, n not stated; 200-500 mg/kg bw i.p., 24 h - 9 d) | Induction (in transgenic mice) |  | (Baek et al., 2016) | Loss of β2-spectrin leads to developmental liver defects, its haploinsufficiency (this model) leads to chronic liver disease and eventual development of hepatocellular cancer.  In addition to transgenic mice, function of SPTBN1 was investigated in various cell lines: HepG2, U-2 OS, HeLa, COS7, 293T, HuH-7, SNU-761.  Acetaminophen induced hepatotoxicity (including necrosis and steatosis) in WT mice, and the toxicity was reduced in β2SP haplo-insufficient mice. In HepG2 cells, acetaminophen decreased cell viability, and β2SP knockdown counteracted this effect. | Moderate-high confidence, low relevance |
|  |  |  |  | Rodent, in vivo (Fisher 344, male rats, n = 3/group, 150-2000 mg/kg bw by gavage, 6-48 h) | Not assessed |  | (Powell et al., 2006) | Acetaminophen increased nitrotyrosine protein adducts, 8-hydroxy-deoxyguanosine, and base excision repair genes, and decreased GSH. |  |
|  |  |  |  | Rodent, in vivo (C57BL/6J, male mice, n = 6-8/group, on high-fat diet; 50, 100, 200 mg/kg bw by gavage, 24 h)Human, in vitro (“L02”/HeLa cell fatty liver model, 2-8 mM, 24 h. | Exacerbation of HFD/ fatty acid-induced hepatic lipid accumulation | increased: **mTOR**, Srebp-1c and  decreased: p-AMPK, LC3-II, Beclin1 | (Shi et al., 2019) | In mice, acetaminophen (100 and 200 mg/kg) accentuated HFD induced hepatic lipid accumulation, and increased serum ALT and AST, and in the cells, it accentuated oleic acid + alcohol induced lipid accumulation. | Moderate-low confidence, low relevance  Problematic cell line: Contaminated. Shown to be a HeLa derivative (Ye et al., 2015). Originally thought to originate from a normal fetal liver. |
|  |  |  |  | Rodent/human, in vivo ( uPA/SCID human hepatocyte-chimeric mice, male and female, n = 6/group, 1400 mg/kg bw orally, 4-24 h) | Not assessed/concluded |  | (Yamamoto et al., 2007) | In liver, acetaminophen modulated pathways involved in lipid/fatty acid metabolism (aldehyde dehydrogenase, long chain fatty acid CoA ligase, acetyl-CoA acetyl-transferase, hydroxymethylglutaryl-CoA synthase), glycolysis (glyceraldehyde 3-phosphate dehydrogenase and fructose-1,6-bisophosphate), energy metabolism/production (ATP synthase α chain), and oxidative stress (carbonic anhydrase II, peroxiredoxin 1, catalase, 78 kDa glucose-regulated protein, and glutathione S-transferase A2). No lipid measurements were included |  |
|  |  |  |  | Review | No steatosis anticipated at non-cytotoxic concentrations | CYP2E1, Oxidative stress, GSH depletion (primarily by alkylation). HIF-1α activation, Nrf2 activation  COX-1, COX-2, and COX-3 inhibition (mainly in the CNS), inhibition of enzymes in the prostaglandin biosynthesis pathway | (Jennings et al., 2014) | Acetaminophen was selected as a reference chemical for hepatotoxicity as a reactive chemical (thiol reagent oxidising NAD(P)H). Affected biological pathways are related to energy metabolism and cellular reduction potential, the anticipated primary hepatotoxic outcome is (non-specific) cytotoxicity.  Source of hepatotoxicity is mediated by the (CYP2E1-catalysis product) N-acetyl-p-benzoquinone imine, which has a high reduction potential and thus may disrupt the hepatic redox homeostasis.  Further data for this reference chemical are available online: <https://wiki.toxbank.net/wiki/Acetaminophen> (incl. curated information on toxicokinetics, in vivo and in vitro toxicogenomics, proteomics, metabolomics, physico-chemical properties) | High confidence, chemical selection report for SEURAT-1 project, accepted by relevant stakeholders, including co-authors from EFSA, EURL ECVAM/JRC, academia, industry and national public health authorities |
|  |  |  |  | Review on the role of the mitochondrial/lysosomal axis in acetaminophen hepatotoxicity. | Not assed or reported | Increased mitochondrial oxidative stress, mitochondrial protein adducts, ASK1, JNK mitochondrial transition pore opening, release of lysosomal proteases (cathepsin B and D), and decreased GSH. | (Moles et al., 2018) | No mention of steatosis. Conditions resulting in increased cholesterol accumulation in lysosomes potentiates acetaminophen hepatotoxicity. Changes in autophagy protects vs. acetaminophen toxicity. |  |
|  |  |  |  | Literature review with a focus on (impaired) mitochondrial fatty acid oxidation and its role in drug-induced liver injury/ steatosis | Induction (microvesicular steatosis in vivo: rodent, human, canine) | Mitochondrial fatty acid oxidation impairment  **CYP2E1, CYP3A4**, succinate dehydrogenase, oxidative stress (GSH depletion), PUMA;  Not involved: CPT1 | (Fromenty, 2019) | Severe impairment of mitochondrial fatty acid oxidation results in microvesicular steatosis (and hypoglycaemia and liver failure), while moderate inhibition of mitochondrial fatty acid oxidation in the liver can cause microvacuolar steatosis, which is benign/reversible in the short term.  Metabolism in the liver mainly into nontoxic sulphate and glucuronide conjugates, but also via CYP2E1 and CYP3A4 into reactive N-acetyl-*p*-benzoquinone (NAPQI), which is associated with adverse effects (mainly via adduct formation, GSH depletion/oxidative stress)  Inhibition of the mitochondrial respiratory complex: succinate dehydrogenase (complex II) and complex III are particularly sensitive to NAPQI-mediated inhibition/toxicity.  Acetaminophen intoxication, but also treatment, was associated with elevated long-chain fatty acids (long-chain acylcarnitines in serum and liver, such as palmitoylcarnitine and oleoylcarnitine) | High confidence, high relevance |
|  |  |  |  | Literature review on the hepatotoxicity of acetaminophen in NAFLD in experimental models and humans. | Hepatotoxicity, but steatosis not specified |  | (García-Román and Francés, 2020) | Acetaminophen produces greater hepatotoxicity in experimental models of NAFLD, and in some human studies greater toxicity was observed in NAFLD patients.  Steatosis/ fatty liver is a risk factor for drug-induced liver injury after acetaminophen treatment. |  |
|  |  |  |  | Review on acetaminophen hepatotoxicity. | Hepatotoxicity, but steatosis not specified | Oxidative stress, mitochondrial stress, inflammation | (Larson, 2007) | Contains no mention of steatosis. Acetaminophen overdose causes hepatotoxicity (incl. necrosis, increased serum ALT and AST, jaundice, acute liver failure) in humans.  Acetaminophen decreases GSH, mitochondrial function, and ATP, disrupts calcium homeostasis, and increases DNA damage, intracellular protein modification, interferon gamma (IFNγ), influx of natural killer cells, natural killer T cells, neutrophils, and macrophages. |  |
|  |  |  |  | Literature review on acetaminophen-induced liver injury in obesity and NAFLD. | Hepatotoxicity, but steatosis not specified | **CYP2E1** potentially involved in acetaminophen hepatotoxicity | (Michaut et al., 2014) | There is no mention of steatosis. Acetaminophen overdose induced hepatotoxicity is increased in patients with NAFLD or alcoholic liver disease, and also in some rodent models of NAFLD.  Acetaminophen increases JNK activation, ROS, peroxynitrate, and inflammation, and decreases mitochondrial function and ATP. | High confidence, moderate-low relevance |
|  |  |  |  | Review on acetaminophen hepatotoxicity. | Not assessed/ reported | CYP enzymes (esp. CYP2E1) | (Sumioka et al., 2004) | There is no mention of steatosis. Acetaminophen induces hepatotoxicity (incl. necrosis) in rodents and humans.  Acetaminophen increases hydrogen peroxide, superoxide, peroxynitrite, lipid peroxidation, plasma ALT, and decreases catalase, GPX, α-tocopherol, coenzyme Q9 and Q10, and GSH/GSSG ratio.  mechanisms underlying APAP toxicity are considered to be associated with i) covalent binding to cellular macromolecules of a reactive intermediate metabolite of APAP produced by cytochrome P450 (CYP), N-acetyl-p- benzoquinoneimine, and ii) oxidative stress. |  |
|  |  |  |  | Human, in vivo (epidemiology, n=461 incidences of drug-induced liver injury) | Not specified |  | (Andrade et al., 2005) | Incidences of drug-induced liver injury were submitted to the Spanish Registry over a period of 10 years (April 1994-August 2004). Of 570 initially submitted cases, 461 fulfilled formal criteria for drug-induced liver injury, and are included in this study, involving 505 pharmaceuticals. Drug-induced liver injury was classified as hepatocellular, cholestatic, or mixed (steatosis not differentiated/specified).  The most frequently associated group of pharmaceuticals were anti-infectives, with amoxicillin clavulanate accounting for 12.8% of cases; acetaminophen is not listed under the 19 main drugs suspected to induce drug-induced liver injury. |  |
|  |  |  |  | Human, in vivo (epidemiology; retrospective cohort, n=696 cases) | Not specified (hepatotoxicity reported) |  | (Beer et al., 2007) | Reports of all cases of paracetamol poisoning and severe liver dysfunction admitted to the liver unit in Newcastle (September 1996-March 2003) or Edinburgh (January 1992-June 2004) were included in this study.  Article evaluating the frequency of liver dysfunction after paracetamol overdoses that were below current UK thresholds for acetaminophen overdose (in non-risk patients: plasma paracetamol levels of 200 mg/L at 4 h post-ingestion).  Acetaminophen overdoses caused hepatic toxicity, including increased serum ALT.  In some patients, hepatotoxicity occurred below the overdose threshold, but these events were rare and, based on this study, do not warrant decreasing the overdose threshold. |  |
|  |  |  |  | Human, in vivo (nested case-control study, n = 198 pateints with acetaminophen-induced acute liver failure; 99 survivors, 99 non-survivors) | Not reported |  | (Karvellas et al., 2017) | Clinical study evaluating the associations between FABP7 serum levels and outcomes in acetaminophen-induced acute liver failure  Of 696 patients, 553 (79%) presented >15 h after exposure and 19 (2.7%) were excluded due to insufficient detail in documentation. Of the remaining 124 presenting within 15 h of exposure, 105 (81%) fulfilled criteria for paracetamol overdose.  Acetaminophen overdose induced hepatotoxicity (including increased serum ALT and liver failure). FABP7 may not discriminate between patients with or without acetaminophen induced intracranial complications. | Moderate confidence, low relevance |
|  |  |  |  | Human, in vivo (clinical adverse effect report , n=1, 9 year old child) | Induction |  | (Bouvet et al., 2020) | Acetaminophen induced necrosis and microvesicular steatosis, and increased serum levels of AST and ALT. | Moderate-low confidence, high relevance  Single case study |
|  |  |  |  |  |  |  |  | Other potentially supportive literature from human in vivo studies reviewed, but not satisfying criteria for detailed listing (e.g., no monotherapy group, or not reporting on hepatic lipid levels): (Zheng et al., 2015b, Jetten et al., 2012, Canet et al., 2015, Martin et al., 2006, Li et al., 2018) |  |
| **GW3965** | 405911-17-3 (hydrochloride) | 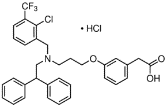 | Candidate pharmaceutical |  |  |  |  | LXR agonist (EC50 = 190 nM hLXRα and 30 nM hLXRβ)  Extensively reviewed and discussed in (Jennings et al., 2014).  Other LXR agonists of interest are oxysterols, which are the endogenous ligands for LXR. |  |
| **Chlorpyrifos** | 2921-88-2 | 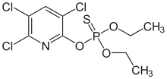 | Organophosphate pesticide | **SUMMARY** | **Negative/ inactive (tentative)** |  |  | **The weight of evidence for concluding on the activity of chlorpyrifos towards primary hepatic steatosis is weak/insufficient; the retrieved literature does not allow confident conclusion on its activity.**  While the mechanisms observed upon exposure to chlorpyrifos are possibly facilitating/contributing to the development of steatosis, it is not likely causative of (primary) steatosis at non-toxic levels.  Retrieved toxicological evaluations of chlorpyrifos by intergovernmental organizations (high confidence) do not support or indicate hepatotoxicity in general, including steatosis/lipid accumulation. Toxicological effects of concern and leading to withdrawal of marketing authorization in the EU from 2020, are indications of (non-genotoxic) carcinogenicity and neurodevelopmental effects, including in children (EFSA, 2019b).  Sex-specific effects (predominantly male) observed in rodents, predisposing animals for type 2 Diabetes and atherosclerosis in adulthood by early postnatal exposure; this could also be a contributory route to metabolic syndrome/ metabolic disruption in the wider sense, but the studies did not report adverse effects in the liver.  EU market approval for plant protection products containing chlorpyrifos (and chlorpyrifos-methyl) as an active ingredient was withdrawn on 6^th^ December 2019, and formally adopted by the European Commission on 10^th^ January 2020. (<https://food.ec.europa.eu/plants/pesticides/approval-active-substances/renewal-approval/chlorpyrifos-chlorpyrifos-methyl_en#modal>) |  |
|  |  |  |  | Human, in vitro (differentiated HepaRG cells, 24-72 h, 1-300 µM) | Inactive *in vitro* (HepaRG) |  | (Lichtenstein et al., 2020) |  | Very high confidence |
|  |  |  |  | Review | Inactive *in vivo* (rodent) |  | (Lichtenstein et al., 2020) | Data reviewed based on test guideline studies according to OECD TG 417. Data source: list of endpoints on residues, active substance. | Very high confidence; literature data based on guideline studies |
|  |  |  |  | Review | Not concluded | CYP enzymes, PON1  Oxidative stress (secondary effect; resulting from generation of reactive metabolites) | (Androutsopoulos et al., 2013) | Detoxified in the liver via CYP (especially CYP2B6, CYP2C19, CYP3A4), yielding “oxon” metabolites, that are further hydrolysed via phosphotriesterases, such as PON1. Chlorpyrifos can inactivate CYP enzymes by covalently binding the sulfur atom that arises during oxidative desulfuration in phase I metabolism. Oxon metabolites mediate neurotoxicity. | Low relevance, high confidence |
|  |  |  |  | Rodent, in vivo (neonatal exposure of Sprague-Dawley rats; 1 mg/kg/d on postnatal days 1-4 via s.c. injection. N=8 per sex per treatment group; samples taken on pnd 110 and 120) | Not concluded |  | (Slotkin et al., 2005) | Exposure of offspring was shown previously to produce developmental neurotoxicity without eliciting growth retardation or any other signs of systemic toxicity. Brain Acetylcholine esterase inhibition was 25% and, thus below the 70% threshold to elicit symptoms of cholinergic hyperstimulation (both, in rats and pregnant women)  **Sex-specific** effects on blood cholesterol and triglyceride levels: elevated (approx. by 35%) in males (non-significant in females). Effect persisted throughout fasting. No changes in plasma levels of non-esterified free fatty acids or glycerol were recorded in adulthood. No effects on blood glucose concentration, but insulin was elevated in non-fasted males (approx. 60% higher than in control); fasting restored insulin levels to normal. | High relevance, high confidence. One author is affiliated with pharmaceutical industry. |
|  |  |  |  | Rodent, in vivo (neonatal exposure of Sprague-Dawley rats; 1-40 mg/kg/d via s.c. injection on gestational days (GD) 9–12 or 17–20 [exposure of dams], postnatal days (PN) 1–4 or 11–14 [exposure of offspring]; effects observed until PN 60; tissues harvested on PN 21) | Not concluded | **Adenylyl cyclase** (male specific). Involved receptors: β-adrenoreceptor, glucagon receptor | (Meyer et al., 2004) | Developmental (neuro)toxicity becomes evident at doses about 5 mg/kg/d.  Reduced liver weight at prenatal doses > 5 mg/kg/d; no effect on liver weight by postnatal treatment. (Vulnerability to chlorpyrifos decreases after PN 4)  Direct action of organophosphate chlorpyrifos on neurotransmitter receptors and/or adenylyl cyclase is insufficient to explain the distinct sex-, tissue-, and age-selective actions observed in this study. Indeed, many effects observed here emerge only with a substantial delay (on PN 30-60), indicating **changes in the programming of cell development, rather than receptor-mediated acute toxicity**. Acute effects of chlorpyrifos on adenylyl cyclase signalling were poorly correlated with the subsequent emergence of major, sex-dependent, tissue-selective functional alterations.  Hyperresponsiveness (to glucose) of hepatic cell signalling mediated through **adenylyl cyclase** (male specific). Critical receptors: β-adrenoreceptor, glucagon receptor, but hyperresponsiveness of the adenylyl cyclase was observed with multiple stimuli (also with direct, receptor-independent stimulation by forskolin). | Moderate relevance, very high confidence |
|  |  |  |  | In silico model (PBPK/PD model) for (Sprague-Dawley) rats | Not concluded | CYP enzymes, PON1, B-esterase | (Timchalk et al., 2007) | Physiologically-based pharmacokinetic/pharmacodynamic model for rats was developed, incorporating age-dependent changes in CYP enzymes, PON1, and B-esterase (B-esterase is used as a cumulative proxy of other esterase activity, such as acetyl-/butyrylcholine esterase, carboxylesterase). (Model developed for choline esterase activity)  Model simulations suggest that pre-weanling rats are particularly sensitive to chlorpyrifos exposure. The age-dependent nonlinear increase in the chlorpyrifos-oxon metabolite concentration may potentially result from both the depletion of nontarget B-esterase and a lower PON1 metabolic capacity in younger animals. | Lower relevance, high confidence |
|  |  |  |  | Review of human health hazards for regulatory purposes | Not reported/ negative |  | (EFSA, 2019b) | Peer review of mammalian toxicity and human health by EFSA requested by the European Commission.  Based on this report, market approval in the EU was withdrawn in December 2019 (adopted by the European commission in January 2020).  Main toxicological concerns were the unclear genotoxic potential and neurodevelopmental effects. The latter was observed in rats in vivo, and supported by epidemiological evidence related to developmental neurological outcomes in children, and is most critical for non-renewal of market approval of chlorpyrifos (and the structurally similar chlorpyrifos-methyl) as the active ingredient of plant protection products.  There is no indication of hepatotoxicity, including steatosis. | High confidence and relevance |
|  |  |  |  | Review; intergovernmental organization | Not reported/ negative |  | (FAO/WHO Joint Meeting on Pesticide Specifications, 2009, Joint FAO/WHOA Meeting on Pesticide Residues, 2006) | There is no evidence suggesting hepatotoxicity in general, or steatosis/lipid accumulation specifically. | High confidence and relevance |
|  |  |  |  | Review for regulatory purposes (toxicological hazard for evaluation of chemicals listed under the Stockholm convention on POPs) | Not reported/ negative |  | (POPs Review Committee, 2022) | Adverse effects on human health are mainly related to neurological/neurodevelopmental effects: exposure to chlorpyrifos during pregnancy was associated with adverse neurodevelopmental outcomes in children, including changes in brain morphology and behavioural symptoms (in a matched mother-child pair epidemiological cohort). Acute poisoning events were dominated by neurotoxic effects and summarized as the cholinergic toxidrome with inhibition of (acetyl)cholinesterase as a central mechanism.  There is no evidence suggesting hepatotoxicity in general, or steatosis/lipid accumulation specifically. | High confidence and relevance |
|  |  |  | Chlorpyrifos-methyl | Review; intergovernmental organization | Not reported/ negative |  | (FAO/WHO Joint Meeting on Pesticide Residues, 2009) | Chlorpyrifos-methyl was concluded to be unlikely genotoxic, not carcinogenic, and not teratogenic. Developmental toxicity (in rats) was observed only at doses causing maternal toxicity; it was unlikely to produce delayed neuropathy in the absence of severe cholinergic toxicity.  In two studies in human volunteers exposed orally to chlorpyrifos-methyl for 21 or 28 days, there were no adverse findings concerning clinical signs, clinical chemistry or cholinesterase activity. The NOAEL was 0.3 mg/kg bw per day over 21 days, the highest dose tested. A single oral dose of (the closely related chemical) chlorpyrifos of up to 1 mg/kg bw did not significantly inhibit erythrocyte acetylcholinesterase activity in human volunteers. There were no reports of adverse effects in production-plant workers.  There is no evidence suggesting hepatotoxicity in general, or steatosis/lipid accumulation specifically. | High confidence and relevance |
| **Thiacloprid** | 111988-49-9 | 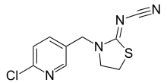 | Neonicotinoid insecticide | **SUMMARY** | **Uncertain: negative/ weak induction** | Hepatic aromatase, thyroid hormone signalling |  | **The weight of evidence for thiacloprid effects to the liver is moderate-weak** in general, and **weak/insufficient to conclude on induction of (primary) hepatic steatosis** specifically. There is no strong indication for thiacloprid causing substantial lipid accumulation, but some potential cannot be ruled out.  In rodents, increased liver weight was reported in one study (Alarcan et al., 2020), but not in GLP studies reviewed for the identification of Cumulative Assessment Groups by EFSA (Nielsen et al., 2012). No accumulation of lipids in hepatocytes or 3T3-L1 pre-adipocytes was detected.  However, in HepaRG cells in vitro, moderate lipid accumulation was observed at high concentrations (≥ 100 µM (Lichtenstein et al., 2020)). It is unclear if this is due to interspecies differences, or limited toxicokinetics in vitro. Human in vivo data were not retrieved.  Market authorisation in the EU withdrawn on 3 Feb 2020 based on an unacceptable level of risk for honeybees (EFSA, 2019a) (A potential structurally similar chemical to pursue could be acetamiprid, CASRN: 135410-20-7, with market approval in EU granted until 2033 (low risk for honeybees (EFSA, 2016))). |  |
|  |  |  |  | Human, in vitro (differentiated HepaRG cells, 24-72 h, 1-1000 µM) | Moderate induction *in vitro* (HepaRG) (LOEC = 100 µM) |  | (Lichtenstein et al., 2020) | EuroMix project deliverable with quantitative in vitro-in vivo extrapolation calculations for several pesticides, but thiacloprid as a model chemical. A human toxicokinetic model is provided. | Very high confidence |
|  |  |  |  | Review | Inactive *in vivo* (rodent) |  | (Lichtenstein et al., 2020, Nielsen et al., 2012) | Data reviewed based on test guideline studies according to OECD TG 417. Data source: list of endpoints on residues, active substance. | Very high confidence; literature data based on guideline studies |
|  |  |  |  | Rodent, in vitro (undifferentiated 3T3-L1 murine fibroblasts, 8 d differentiation protocol; 6 d exposure; 1-300 mg/L) | Not concluded (but no lipid accumulation in 3T3-L1 cells) | PXR | (Mesnage et al., 2018) | In vitro screening of neonicotinoid pesticide effects on estrogen and thyroid hormone receptor binding, and lipid accumulation in murine preadipocytes.  Thiacloprid did not show agonism towards estrogen (human MCF-7 cells) or thyroid hormone receptor (rat pituitary GH3 cells), and did not induce lipid accumulation in 3T3-L1 murine fibroblasts. However, the related pesticide **imidacloprid did induce triglyceride accumulation in 3T3-L1 murine fibroblasts at ≥50 mg/L** imidacloprid. This may be mediated by PXR activation. | Moderate relevance, moderate confidence |
|  |  |  |  | Chicken, in vivo (*Gallus gallus domesticus*, n=75 fertilised eggs, exposure by injection into albumen; 1-20 dpf, 0.92-92 µg/mL) | Yes. |  | (Salvaggio et al., 2018) | Liver steatosis was observed in chicks exposed to 0.92 and 9.2 µg/mL, and was accompanied by erythrocyte infiltrates into the liver tissue.  Teratogenicity, the primary endpoint of the study, was observed at all concentrations. | Low confidence, high relevance  Histological specimen and interpretation seem reliable, but quality of the publication is below average |
|  |  |  |  | Rodent, in vivo (female Wistar rats, 28-day oral toxicity study; 10-140 mg/kg bw/d; n=4 per group) | No steatosis |  | (Alarcan et al., 2020) | Liver weight increase, (potentially receptor-mediated) centrilobular hepatocellular hypertrophy, and cytoplasmic degeneration  Liver triglycerides showed a dose-dependent decreasing trend, but differences did not reach statistical significance. | High relevance, high confidence  Small sample size (4 animals per treatment group) |
|  |  |  |  | Rodent, in vivo (female Wistar rats, 28-day oral toxicity study; 10-140 mg/kg bw/d; n=4 per group) | No steatosis (inferred from (Alarcan et al., 2020)) | **PXR, PPARγ, xenobiotic metabolism** (especially ALDH1A1, CYP2B6, and CYP3A5)  Top ten differentially expressed genes  Upregulated: *Abcc3,* ***Aldh1a1****,* ***Cyp2b2****,* ***Cyp3a23/3a1****, Mme*  Downregulated: *Cyp7a1, Eln, Ky, Megf11, Slc6a1* | (Alarcan et al., 2021) | This study was a follow-up study of (Alarcan et al., 2020)  Total of differentially expressed genes upon thiacloprid exposure: 255  Among the differentially expressed genes, a subset of 64 genes was shared amongst two other pesticides (clothianidin, imazalil) with a similar mode of action. Shared pathways include CAR and PXR-mediated responses, with downstream activation of metabolism-associated functions (metabolism of xenobiotic, metabolism of terpenoid, conversion and oxidation of lipid). | High confidence, high relevance |
| **Acetamiprid** | 135410-20-7 | 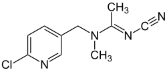 | Neonicotinoid insecticide |  | **Tentative negative** |  |  | Market approval in EU granted until 2033 (low risk for honeybees) (EFSA, 2016)  From JMPR (2011): no indication for steatosis induction, including evaluation of occupational exposure and human poisoning events. Critical effects in rodents were: decreased body weight gain and decreased food consumption; hepatocellular hypertrophy. (No new data were available for an update request to the JMPR in 2017) |  |
| **Thiamethoxam** | 153719-23-4 | 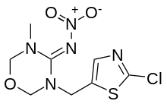 | Neonicotinoid insecticide | **SUMMARY** | **Uncertain**  possibly inactive in human in vitro (HepaRG), but (weak) positive in rodent in vivo |  | (Lichtenstein et al., 2020) | **The weight of evidence** for thiamethoxam effects to the liver is moderate-weak in general, but **weak/insufficient to conclude on induction of (primary) hepatic steatosis** specifically.  In rodents, GLP studies reviewed for the identification of Cumulative Assessment Groups by EFSA (Nielsen et al., 2012) indicate fatty changes in the liver, in vivo, however, other hepatic changes observed are more related to cytotoxicity and/or carcinogenicity than to steatosis. However, a recent study reported induction of dyslipidaemia and NAFLD, including histopathological evidence of steatosis in rat liver (Yang et al., 2021)  In HepaRG cells in vitro, no lipid accumulation was observed up to 1 mM (Lichtenstein et al., 2020). It is unclear if this is due to interspecies differences, or limited toxicokinetics in vitro. Human in vivo data were not retrieved.  Based on the larger discrepancy between (rodent) in vivo and (human) in vitro effects for thiamethoxam, inclusion of other neonicotinoid pesticides such as thiacloprid for testing in GOLIATH is preferred, despite the very limited amount of retrieved literature.  Market approval for EU expired on 30 April 2019 based on an unacceptable level of risk for honeybees. (EC, 2018) |  |
|  |  |  |  | Human, in vitro (differentiated HepaRG cells, 24-72 h, 1-1000 µM) | Inactive *in vitro* (HepaRG) |  | (Lichtenstein et al., 2020) |  | Very high confidence |
|  |  |  |  | Review | Fatty changes *in vivo* (rodent) (no cholestasis; steatosis not concluded) |  | (Lichtenstein et al., 2020, Nielsen et al., 2012) | Data reviewed based on test guideline studies according to OECD TG 417. Data source: list of endpoints on residues, active substance.  Toxic to the liver (CAG 1), hepatocellular hypertrophy (CAG 2a), hepatocellular fatty changes (CAG 2b), hepatocellular cell degeneration/ cell death (CAG 2c), inflammation in the liver (CAG 2d), Foci of cellular alteration in the liver (CAG 2e), Neoplasms (CAG 2f), Lesions of biliary epithelium (CAG 2g), Increase in phase 1 liver enzymes (CAG 3a), Foci of cellular alteration and/or liver neoplasms which may be caused by cytotoxicity (CAG 3c), Increase in CYP1A enzymes (CAG 4a1), Increase in CYP2B enzymes (CAG 4a3)  **No** cholestasis. | Very high confidence; literature data based on guideline studies |
|  |  |  |  | Rodent, in vitro (undifferentiated 3T3-L1 murine fibroblasts, 8 d differentiation protocol; 6 d exposure; 1-300 mg/L) | Not concluded | PXR | (Mesnage et al., 2018) | In vitro screening of neonicotinoid pesticide effects on oestrogen and thyroid hormone receptor binding, and lipid accumulation in murine preadipocytes.  Thiacloprid did not show agonism towards oestrogen (human MCF-7 cells) or thyroid hormone receptor (rat pituitary GH3 cells), and did not induce lipid accumulation in 3T3-L1 murine fibroblasts. However, the related pesticide imidacloprid did induce triglyceride accumulation in 3T3-L1 murine fibroblasts at ≥50 mg/L imidacloprid. This may be mediated by PXR activation. |  |
|  |  |  |  | Rodent, in vivo (male ICR mice, 6-8 weeks old, n=30; 12 weeks exposure by gavage, 4-20 mg/kg bw/d) | Yes (dyslipidaemia, NAFLD induction)  Steatosis in liver histopathological specimen at 4 and 20 mg/kg bw/d | **PPARα signalling** **(↓),** glycine N-methyl transferase (↓), **nicotinamide N-methyltransferase** **(↑),** oxidative stress (↑), PPARγ (↑), FASN (↑) | (Yang et al., 2021) | Inhibition of nicotinamide N-methyltransferase in mouse liver reversed the effects of thiamethoxam treatment.  NAFLD is induced through disturbed methionine metabolism and methyl donor imbalance. This leads to downregulation of PPARα signalling, which, in turn, results in increased lipogenesis and dyslipidaemia.  Increasing concentrations of thiamethoxam were associated with decreased superoxide dismutase activity and glutathione levels in the liver, indicating increased oxidative stress.  Plasma triglycerides were significantly increased in high-dose (20 mg/kg bw/d) group. | High relevance, high confidence |
| **Fructose** | 57-48-7 | 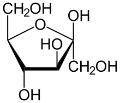 | Dietary monosaccharide; ketonic simple sugar | **SUMMARY** | Highly likely positive but not pursued for test method development reasons |  |  | Based on the interconnectedness of hepatic carbohydrate and lipid metabolism, responses to energy state, and with respect to the bigger picture of metabolic disruption, inclusion of a dietary sugar (glucose or fructose) was considered.  However, with many human in vitro steatosis assays under development, glucose is already a culture medium constituent at a substantial concentration (millimolar range), and a glucose-free media formulation is not available/compatible with the assay. This would make assessment of effects of dietary sugars like glucose or fructose difficult. Furthermore, it might result in a change of cell physiology that cannot be maintained for the longer duration of the assay necessary to detect changes in lipid accumulation.  A detailed literature review was not pursued further, despite indications of substantial available literature for the role of glucose homeostasis in NAFLD/metabolic disruption and syndrome. |  |
|  |  |  |  |  | Induction |  | (Pickens et al., 2009) | Dietary sucrose is essential to the development of liver injury in the methionine-choline-deficient model of steatohepatitis |  |
| **Glucose** | 50-99-7 | 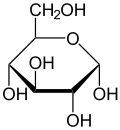 | Dietary monosaccharide; aldehyde simple sugar | **SUMMARY** | As above for fructose |  |  | (See comment on fructose; literature review was not pursued due to likely technical limitations of testing sugars in in vitro assays) |  |
| **Rifampicin** | 13292-46-1 | 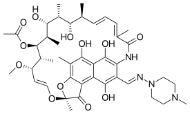 | Antibiotic | **SUMMARY** | **Uncertain/ tentative positive** | **PXR** |  | **The weight of reviewed evidence is conflicting** (limited full-text review conducted). While a reliable study, endorsed by the GOLIATH test method developers (Allard et al., 2020), concludes on rifampicin as a model steatogenic chemical, it was not listed or recognised as such by a literature review for proposing reference chemicals for (in vitro) steatosis test methods (Jennings et al., 2014).  Rifampicin is a positive control chemical for PXR activation, especially in humans. |  |
|  |  |  |  | Review | Not indicated as a steatotic chemical in human | **PXR**, CYP3A4, **bile acid export pump** (BSEP).  Target genes include: CYP3A4, CYP2B6, CYP2C9, (CYP7A1), **CD36**, ALDH1A1, UGT1A1, SULT1A1, ABCB1 | (Jennings et al., 2014) | Rifampicin was selected as a reference chemical as a model inducer of PXR, particularly in humans (rifampicin is a poor ligand for rat and mouse PXR). Biological pathways affected by rifampicin are cholesterol and fatty acid metabolism, and clearance of xenobiotics.  Rifampicin has a major adverse event frequency of 0.4 per 100 person-months of continuous dosing, with the main targets being the liver (hepatitis) and the gastrointestinal tract.  Rifampicin-induced cholestasis is probably mediated via competitive inhibition of the hepatic bile acid export pump, leading to accumulation of bile acids in hepatocytes. This is likely not linked to PXR activation, since PXR, complemented by FXR, is protective against cholestasis.  Further data for this reference chemical are available online: <https://wiki.toxbank.net/wiki/Rifampicin> (incl. curated information on toxicokinetics, (epi-)genomics, metabolomics, physico-chemical properties) | High confidence, chemical selection report for SEURAT-1 project, accepted by relevant stakeholders, including co-authors from EFSA, EURL ECVAM/JRC, academia, industry and national public health authorities |
|  |  |  |  | Human, in vitro (differentiated HepaRG, 96 h exposure, 10-300 µM) | Increase | VLDL assembly gene expression decreased at 150 µM: APOB, APOC3, MTTP, P4HB, ANGPTL3  ER stress gene expression increased at 300 µM: HSPA5 (BIP), DDIT3 (CHOP), ERN1 (IRE1α), ATF6. Unaltered: EIF2AK3 (PERK) | (Allard et al., 2020) | Positive control chemical for steatosis  **LOEC** in differentiated HepaRG cells: Mitochondrial fatty acid oxidation decreased: 100 µM; de novo lipogenesis increased: 10 µM; apoB secretion into medium: increased at 150 µM, decreased at 300 µM; neutral lipid accumulation increased: 100 µM. (steatosis induced also in primary human hepatocytes in vitro, but only in one out of six cell batches tested) | Very high confidence and relevance |
|  |  |  |  | Human, in vitro (undifferentiated HepaRG in 2D or 3D spheroid culture; exposure after 7 d pre-growth for 1-7 d; 9-step two-fold dilution from 100 µM) | Not assessed |  | (Ott et al., 2017) | High-throughput HepaRG CYP induction assay. CYP3A4 activity was assessed by P450-Glo Luciferin-IPA assay (Promega), CYP1A activity by EROD assay, cytotoxicity as ATP content by CTG assay (Promega).  Therapeutic maximum concentration in vivo: 12.2 µM.  Cytotoxicity: IC50 in 2D:0.04 µM (24 h), >100 µM (7 d) IC50 in 3D: n/a | High confidence, moderate relevance (high for cytotoxicity)  The authors declared a conflict of interest for the micromolds used to produce the HepaRG 3D spheroids |
| **Tetracycline** | 60-54-8  (64-75-5 hydrochloride) | 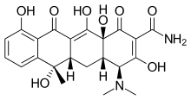 | Antibiotic | **SUMMARY** | **Positive**  **Induction** |  |  | **Strong weight of evidence supporting tetracycline inducing steatosis in vitro and in vivo; tetracycline is a model steatosis-inducing chemical.**  The literature summarised below is partial; due to the strong weight of evidence, including historic data more than 50 years old, not all retrieved studies were subjected to a full-text scrutinous review. Examples with a focus on in vitro studies are listed below.  **Tetracycline was selected as a reference chemical for hepatotoxicity as a disruptor of mitochondrial function and fatty acid metabolism, inducing steatosis in the EU funded project LIINTOP.** |  |
|  |  |  |  | Human, in vitro (undifferentiated HepaRG, passage 22-25. 1-14 d exposure, 0.2-20 µM) | Not assessed |  | (Limonciel et al., 2011) | Increased glycolysis with subsequent lactate production.  LOEC 14 d cytotoxicity: 5 µM (on intracellular ATP content and esterase activity by resazurin reduction; at 5 µM reduced to ≈50%) | Lower relevance towards steatosis, deviating culture medium used for HepaRG cultivation, HepaRG cells were not differentiated |
|  |  |  |  | Rodent, ex vivo (liver slices from C57BL/6 male mice (n = ≥4 mice), 24 weeks old. 0-100 µM (40 µM for gene expression profiling), 24 h) | Not assessed. Tetracycline was studied as a model steatogenic chemical, but fatty liver gene expression clusters were downregulated. | Inhibition of fatty acid catabolism in liver cells  Downregulated genes: Abcd2, Onecut1, Slc10a2, Acsm3, Bdh1, Nox2, Bbox1, Cxcl12, Slc16a7, Igfbp1, Fas, Maob, Elovl2, Hadh, Hsd17b4, Glyat, Crot, Slc25a20, Slc27a2 | (Szalowska et al., 2014) | 5 mm-diameter cylindrical liver cores were obtained by biopsy; after processing, slices were 0.2 mm thick and weighted ~6 mg per slice.  Genes significantly downregulated (fold-change ≤-1.5) in GSEA and proposed as biomarkers for tetracycline-like acting chemicals: Abcd2, Onecut1, Slc10a2, Acsm3, Bdh1, Nox2, Bbox1, Cxcl12, Slc16a7, Igfbp1, Fas, Maob, Elovl2, Hadh, Hsd17b4, Glyat, Crot, Slc25a20, Slc27a2  While amiodarone and valproate induced gene expression changes clustered together, tetracycline induced a distinctly different pattern. Main differences were found in gene sets related to **lipid metabolism, fatty liver, and peroxisomes**, which were upregulated by both AMI and VA, and **downregulated by TET**. Moreover, only TET-treatment downregulated gene sets related to other hepatic functions including bile acid metabolism, FA metabolism, ABC transporters, and cholesterol synthesis.  Tetracycline downregulated gene ontology clusters related to mitochondrion and processes localized in this organelle, such as electron carrier activity.  In contrast to AMI and VA, TET downregulated functional clusters related to lipid synthesis, β-oxidation, *Pparα* signalling, inflammation, apoptosis, and other clusters related to energy and bile acid homeostasis. |  |
|  |  |  |  | Human, in vitro (HepG2 and differentiated HepaRG, pre-loaded for 14 h with 62 µM oleate palmitate (2:1); 50-400 µM, 24 h) | Induction, very strong (up to >500% in HepaRG) | Oxidative stress (ROS; significant at ≥100 µM), decreased mitochondrial membrane potential (significant at 200-400 µM)  Gene expression changed: EHHADH, MTTP, IGFBP1, SREBP1C | (Tolosa et al., 2016) | Exposure of cells to test chemicals was conducted in fatty-acid-free medium.  Tetracycline is included as a model steatogenic chemical (Drug-induced liver Injury category: “mild”), also causing mitochondrial impairment – which is possibly also a contributory mechanism to intracellular lipid accumulation.  In HepaRG: Significant lipid accumulation at 100 µM and above (p<0.005 for ≥200 µM). No effect on cell viability.  Gene expression analysed (500 µM), but unchanged: CROT, ANGPTL3, FOXA1, PPARA, PGC1A | Very high confidence and relevance |
|  |  |  |  | Rodent, in vivo & ex vivo (Sprague-Dawley rats (male, female, pregnant: 16-20 d gestation), 20-200 mg/kg bw, i.v., 3 h) | Increase | Export of triglycerides from hepatocytes is impaired | (Breen et al., 1975) | co-exposure of ex vivo liver specimen with oleic acid: 663 µmol oleic acid in 70 mL perfusion solution.  Significantly increase accumulation of triglycerides at ≥100 mg/kg bw (without oleic acid supplementation in intact rat); with oleic acid and in perfused livers: ≥50 mg/kg bw in males, ≥75 mg/kg bw in females (lowest concentration tested in females), ≥100 mg/kg bw in pregnant females (lowest concentration tested in pregnant females). When pure tetracycline was added to perfusate, significant increase in triglyceride accumulation was observed in male liver specimen at all concentrations (2.5-20 mg, in 70 mL perfusate).  Decrease in lipid export from the liver/from hepatocytes was dose/concentration-dependent both, in vivo and ex vivo.  “In the intact male and female rat, no direct relationship was observed between dose of tetracycline and hepatic accumulation of triglyceride. Supplementation of ex vivo liver biopsy specimen with oleic acid resulted in observation of a direct relationship between dose of tetracycline and both accumulation of triglyceride in the liver and depression of output of triglyceride by livers from male and female rats.”  In female, and especially in pregnant female, rats lipid export from hepatocytes was markedly decreased; this was not related to altered disposition of tetracycline or altered oleic acid uptake (in ex vivo setting). However, increased intracellular lipids could only be accounted to 30-50% to the decreased export of triglycerides, therefore other mechanisms than decreased export of triglycerides are involved in hepatic lipid accumulation. | Very high relevance |
|  |  |  |  | Human, in vitro (differentiated HepaRG, 1-14 d exposure, 10-100 µM) | Induction of steatosis after repeated dose exposure | Gene expression significantly changed (in **bold**: at more than one concentration) after 24 h exposure: SLC27A4 (↑), PPARGC1A (↑), PPARG (↑), SREBP1 (↑), THRSP (↑), ADFP (↑), PLIN4 (↑), **CYP2E1 (↓)** after 14 d exposure: **FABP1 (↑),** THRSP (↑), LSS (↑), ADFP (↑), CYP2E1 (↓) | (Anthérieu et al., 2011) | Elevated triglycerides at 100 µM after 14 d exposure; TG accumulation was proportional to the increased expression of lipogenic genes.  Fatty acid oxidation impaired upon chronic exposure (14 d) to 50 µM only; no decrease after acute exposure (24 h).  Gene expression studies: acute 24-h exposure: 50 µM, 100 µM; chronic 14-d exposure: 10 µM, 50 µM | Very high confidence and relevance |
|  |  |  |  | Human, in vitro (undifferentiated HepaRG in 2D or 3D spheroid culture; exposure after 7 d pre-growth for 1-7 d; 9-step two-fold dilution from 3800 µM) | Not assessed |  | (Ott et al., 2017) | High-throughput HepaRG CYP induction assay. CYP3A4 activity was assessed by P450-Glo Luciferin-IPA assay (Promega), CYP1A activity by EROD assay, cytotoxicity as ATP content by CTG assay (Promega).  Therapeutic maximum concentration in vivo: 9 µM.  Cytotoxicity: IC50 in 2D: 3559.5 µM (24 h), 574.8 µM (7 d) IC50 in 3D: >3800 µM (24 h), 210.6 µM (7 d)  Interlaboratory IC50 comparison: 199-242 µM (8 d, 3D HepaRG), 210.6 µM (7 d, 3D HepaRG), 116-132 µM (8 d, 3D primary human hepatocytes) | High confidence, moderate relevance (high for cytotoxicity)  The authors declared a conflict of interest for the micromolds used to produce the HepaRG 3D spheroids |
|  |  |  |  | Human, in vitro (differentiated HepaRG, 10-50 µM, 24 h – 14 d) | Induction | Genes upregulated after 14 d treatment: FABP1 (only with one concentration: ADFP, LSS, THRSP, CYP2E1) | (Anthérieu et al., 2011) | Triglyceride accumulation was only significant upon treatment with 50 µM for 14 d (not with lower concentrations, or at the earlier timepoint/shorter exposure); this was accompanied by a significant reduction in fatty acid oxidation. | High confidence, very high relevance |
| Oleic acid | 112-80-1 | 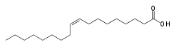 | Dietary constituent; (monounsaturated omega-9) fatty acid | **SUMMARY** | **Positive**  **Test method positive control** |  |  | **Very strong weight of evidence to support oleic acid inducing hepatic steatosis.** Oleic acid is used as a positive control/reference chemical both, in vitro and in vivo.  The literature displayed below is exemplary; oleic acid was included as a/the positive control in most reviewed in vitro studies (quantitatively) evaluating intracellular lipid accumulation (listed above). As a positive control chemical, it was often applied in equimolar mixture (1:1) with palmitic acid.  Unsaturated fatty acids are well established as having nutritional modulatory function in lipid metabolism. |  |
|  |  |  |  | Human, in vitro (HepaRG cells, 6 d, 225 µM) | Strong induction |  | (Knebel et al., 2019a) | Positive/sensitivity control: mixture of oleic acid and palmitic acid (1:1; 225 µM) induces approx. 2.8-fold induction. |  |
|  |  |  |  | Human, in vitro (differentiated HepaRG, 1 -14 d exposure, 500 µM) | Induction of steatosis | Gene expression significantly changed (in **bold**: at more than one concentration) after 24 h exposure: CPT1A (↑), **FASN (↑),** **ADFP (↑),** PLIN4 (↑), CYP3A4 (↓) after 14 d exposure: CPT1A (↑), **THRSP (↑),** **ADFP (↑),** **CYP2E1 (↑), ALDB (↓)** | (Anthérieu et al., 2011) | Positive control; TG accumulation was proportional to the increased expression of lipogenic genes.  Gene expression studies: acute 24-h exposure: 250 µM, 500 µM; chronic 14-d exposure: 250 µM, 500 µM | Very high confidence and relevance |
|  |  |  |  | Human, in vitro (HepG2 cells; 12-48 h, 0.1-2.0 mM | Strong induction | CYP1A1 | (Huang et al., 2018) | CYP1A1, expression is increased in oleic acid-stimulated HepG2 cells, catalysis (or is involved in the catalysis of) lipid peroxidation of lipids.  ≥1 mM reduced HepG2 proliferation. |  |
|  |  |  |  | Rodent, in vitro (RAW264.7 murine macrophage cell line, 65 µg/mL oleate vs. 65 µg/mL oleate + 1.5 µg/mL resveratrol; 24 h) | Not concluded, but model-inducer of intracellular lipid accumulation in murine macrophages | Gene expression: Oleate-only: Cd36, Fatp1, **Fatp2**, **Dgat1**, **Cpt1a**, and **Pnpla3** were significantly upregulated, while those of **Scara1**, **Fabp4**, **Fabp5**, and **Srebp1c** were significantly downregulated. [in bold: reversed by resveratrol co-exposure)  Resveratrol co-treatment: Cd36, Lipa, and Lpl were significantly increased and those of Fabp1 and Fatp1 were significantly decreased | (Ye et al., 2019) | Publication on the metabolomic effects of oleate treatment with and without resveratrol in macrophages.  Resveratrol co-exposure prevented accumulation of neutral lipids (by Nile Red staining).  “Most FFAs, such as palmitic acid, palmitoleic acid, stearic acid, linoleic acid, and eicosanoic acid, were all significantly decreased in oleate-treated macrophages. In contrast, oleic acid, 11,14-eicosadienoic acid and 5,8,11-eicosatrienoic acid were significantly increased in oleate-treated macrophages, and these effects were attenuated or abolished by RSV. Notably, glycerol, glycerol 3-phosphate, 1-monopalmitin, 2-monopalmitin, 1-monooleoylglycerol, and 2-monooleoylglycerol significantly accumulated in oleate-treated macrophages, but these accumulation events were alleviated by RSV.”  Oleate induces disturbances in glycerolipid metabolism and then leads to triglyceride accumulation in macrophages.  Gene expression data indicate disordered lipid transport, increased FFA import into mitochondria for oxidation, and enhanced TG synthesis and lipolysis during neutral lipid accumulation in oleate-treated macrophages. mRNA expression levels of Cd36, Lipa, and Lpl were significantly increased and those of Fabp1 and Fatp1 were significantly decreased in macrophages cotreated with oleate and RSV | Moderate relevance (no effects in hepatocytes) |
| **Palmitic acid** | 57-10-3 | 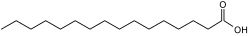 | Saturated fatty acid | **SUMMARY:** | **Positive**  Induction of steatosis might be subject to serum composition/ batch effects, particularly in vitro) |  |  | As opposed to oleic acid, palmitic acid alone does not always relibaly induce steatosis, particularly under (chemically) defined culture conditions and especially with different sera/serum batches. Therefore, **palmitic acid is currently not recommended as a reference or proficiency chemical for a human in vitro steatosis assay**.  The references listed below are selected examples; a complete literature review was not pursued due to time constraints and aforementioned reasonable technical limitations for the use of palmitic acid in the steatosis assay.  Exposure of hepatocytes to high levels of exogenous fatty acids is expected to activate PPARα and therefore modulate the baseline gene expression profile. (Jennings et al., 2014) |  |
|  |  |  |  | Human, in vitro (differentiated HepaRG cells, 6 d, 225 µM) | Active; **positive control** (in 1:1 mixture with oleic acid) |  | (Knebel et al., 2019a) | Positive control for triglyceride accumulation in differentiated HepaRG cells: 1:1 equimolar mixture of oleic acid and palmitic acid (225 µM) induces approx. 2.8-fold increase in triglyceride accumulation (assessed by adipored staining) after 6 d exposure | Operator overlap with (Lichtenstein et al., 2020) |
|  |  |  |  | Human, in vitro (differentiated HepaRG cells, 24-72 h, 250 µM) | Active; **positive control** (in 1:1 mixture with oleic acid0 |  | (Lichtenstein et al., 2020) | Positive control for triglyceride accumulation in differentiated HepaRG cells: 1:1 equimolar mixture of oleic acid and palmitic acid (250 µM) induces approx. 2.5-fold increase in triglyceride accumulation (assessed by adipored staining) after 24-72 h exposure |  |

# References

ABDULHAMEED, M. D. M., PANNALA, V. R. & WALLQVIST, A. 2019. Mining Public Toxicogenomic Data Reveals Insights and Challenges in Delineating Liver Steatosis Adverse Outcome Pathways. *Frontiers in Genetics,* 10.

ABHILASH, P. A., HARIKRISHNAN, R. & INDIRA, M. 2012. Ascorbic acid supplementation causes faster restoration of reduced glutathione content in the regression of alcohol-induced hepatotoxicity in male guinea pigs. *Redox Report,* 17**,** 72-79.

AGUAYO-OROZCO, A., BOIS, F. Y., BRUNAK, S. & TABOUREAU, O. 2018. Analysis of Time-Series Gene Expression Data to Explore Mechanisms of Chemical-Induced Hepatic Steatosis Toxicity. *Frontiers in Genetics,* 9.

AGUIRRE, L., PORTILLO, M. P., HIJONA, E. & BUJANDA, L. 2014. Effects of resveratrol and other polyphenols in hepatic steatosis. *World Journal of Gastroenterology,* 20**,** 7366-7380.

AIRES, C. C. P., IJLST, L., STET, F., PRIP-BUUS, C., DE ALMEIDA, I. T., DURAN, M., WANDERS, R. J. A. & SILVA, M. F. B. 2010. Inhibition of hepatic carnitine palmitoyl-transferase I (CPT IA) by valproyl-CoA as a possible mechanism of valproate-induced steatosis. *Biochemical Pharmacology,* 79**,** 792-799.

AJMO, J. M., LIANG, X., ROGERS, C. Q., PENNOCK, B. & YOU, M. 2008. Resveratrol alleviates alcoholic fatty liver in mice. *American Journal of Physiology - Gastrointestinal and Liver Physiology,* 295**,** G833-G842.

ALARCAN, J., SPRENGER, H., WAIZENEGGER, J., LICHTENSTEIN, D., LUCKERT, C., MARX-STOELTING, P., LAMPEN, A. & BRAEUNING, A. 2021. Transcriptomics analysis of hepatotoxicity induced by the pesticides imazalil, thiacloprid and clothianidin alone or in binary mixtures in a 28-day study in female Wistar rats. *Archives of Toxicology,* 95**,** 1039-1053.

ALARCAN, J., WAIZENEGGER, J., SOLANO, M. L. M., LICHTENSTEIN, D., LUCKERT, C., PEIJNENBURG, A., STOOPEN, G., SHARMA, R. P., KUMAR, V., MARX-STOELTING, P., LAMPEN, A. & BRAEUNING, A. 2020. Hepatotoxicity of the pesticides imazalil, thiacloprid and clothianidin - Individual and mixture effects in a 28-day study in female Wistar rats. *Food Chem Toxicol,* 140**,** 111306.

ALBERTI, G., GANA, J. C. & SANTOS, J. L. 2020. Fructose, omega 3 fatty acids, and vitamin E: Involvement in pediatric non-alcoholic fatty liver disease. *Nutrients,* 12**,** 1-19.

ALLARD, J., BUCHER, S., MASSART, J., FERRON, P.-J., LE GUILLOU, D., LOYANT, R., DANIEL, Y., LAUNAY, Y., BURON, N., BEGRICHE, K., BORGNE-SANCHEZ, A. & FROMENTY, B. 2020. Drug-induced hepatic steatosis in absence of severe mitochondrial dysfunction in HepaRG cells: proof of multiple mechanism-based toxicity. *Cell Biology and Toxicology*.

ALLMYR, M., PANAGIOTIDIS, G., SPARVE, E., DICZFALUSY, U. & SANDBORGH-ENGLUND, G. 2009. Human exposure to triclosan via toothpaste does not change CYP3A4 activity or plasma concentrations of thyroid hormones. *Basic Clin Pharmacol Toxicol,* 105**,** 339-44.

ANDRADE, R. J., LUCENA, M. I., FERNÁNDEZ, M. C., PELAEZ, G., PACHKORIA, K., GARCÍA-RUIZ, E., GARCÍA-MUÑOZ, B., GONZÁLEZ-GRANDE, R., PIZARRO, A., DURÁN, J. A., JIMÉNEZ, M., RODRIGO, L., ROMERO-GOMEZ, M., NAVARRO, J. M., PLANAS, R., COSTA, J., BORRAS, A., SOLER, A., SALMERÓN, J. & MARTIN-VIVALDI, R. 2005. Drug-induced liver injury: An analysis of 461 incidences submitted to the Spanish registry over a 10-year period. *Gastroenterology,* 129**,** 512-521.

ANDROUTSOPOULOS, V. P., HERNANDEZ, A. F., LIESIVUORI, J. & TSATSAKIS, A. M. 2013. A mechanistic overview of health associated effects of low levels of organochlorine and organophosphorous pesticides. *Toxicology,* 307**,** 89-94.

ANGLE, B. M., DO, R. P., PONZI, D., STAHLHUT, R. W., DRURY, B. E., NAGEL, S. C., WELSHONS, W. V., BESCH-WILLIFORD, C. L., PALANZA, P., PARMIGIANI, S., VOM SAAL, F. S. & TAYLOR, J. A. 2013. Metabolic disruption in male mice due to fetal exposure to low but not high doses of bisphenol A (BPA): evidence for effects on body weight, food intake, adipocytes, leptin, adiponectin, insulin and glucose regulation. *Reprod Toxicol,* 42**,** 256-68.

ANSES 2014. Opinion of the French Agency for Food, Environmental and Occupational Health & Safety (ANSES) in response to the consultation of the European Food Safety Authority on its draft Opinion regarding the assessment of risks to human health related to dietary exposure to Bisphenol A. ANSES – Request no. 2014-SA-0033.

ANTHÉRIEU, S., ROGUE, A., FROMENTY, B., GUILLOUZO, A. & ROBIN, M. A. 2011. Induction of vesicular steatosis by amiodarone and tetracycline is associated with up-regulation of lipogenic genes in HepaRG cells. *Hepatology,* 53**,** 1895-905.

APPLETON, R. E., FARRELL, K., APPLEGARTH, D. A., DIMMICK, J. E., WONG, L. T. K. & DAVIDSON, A. G. F. 1990. The High Incidence of Valproate Hepatotoxicity in Infants May Relate to Familial Metabolic Defects. *Canadian Journal of Neurological Sciences / Journal Canadien des Sciences Neurologiques,* 17**,** 145-148.

ARAI, H., YAMASHITA, S., YOKOTE, K., ARAKI, E., SUGANAMI, H., ISHIBASHI, S. & GROUP, K. S. 2018. Efficacy and safety of pemafibrate versus fenofibrate in patients with high triglyceride and low HDL cholesterol levels: A multicenter, placebo-controlled, double-blind, randomized trial. *Journal of Atherosclerosis and Thrombosis,* 25**,** 521-538.

ARDID-RUIZ, A., IBARS, M., MENA, P., DEL RIO, D., MUGUERZA, B., AROLA, L., ARAGONÈS, G. & SUÁREZ, M. 2019. Resveratrol treatment enhances the cellular response to leptin by increasing OBRB content in palmitate-induced steatotic hepg2 cells. *International Journal of Molecular Sciences,* 20.

AVERY, L. B. & BUMPUS, N. N. 2014. Valproic acid is a novel activator of amp-activated protein kinase and decreases liver mass, hepatic fat accumulation, and serum glucose in obese mice. *Molecular Pharmacology,* 85**,** 1-10.

BA, Q., HUANG, C., FU, Y., LI, J., LI, J., CHU, R., JIA, X. & WANG, H. 2015. Cumulative metabolic effects of low-dose benzo(a)pyrene exposure on human cells. *Toxicology Research,* 5**,** 107-115.

BAEK, H. J., LEE, Y. M., KIM, T. H., KIM, J. Y., PARK, E. J., IWABUCHI, K., MISHRA, L. & KIM, S. S. 2016. Caspase-3/7-mediated cleavage of β2-spectrin is required for acetaminophen-induced liver damage. *International Journal of Biological Sciences,* 12**,** 172-183.

BAI, J., HE, Z., LI, Y., JIANG, X., YU, H. & TAN, Q. 2019. Mono-2-ethylhexyl phthalate induces the expression of genes involved in fatty acid synthesis in HepG2 cells. *Environmental Toxicology and Pharmacology,* 69**,** 104-111.

BASSLER, J., DUCATMAN, A., ELLIOTT, M., WEN, S., WAHLANG, B., BARNETT, J. & CAVE, M. C. 2019. Environmental perfluoroalkyl acid exposures are associated with liver disease characterized by apoptosis and altered serum adipocytokines. *Environmental Pollution,* 247**,** 1055-1063.

BEER, C., PAKRAVAN, N., HUDSON, M., SMITH, L. T., SIMPSON, K., BATEMAN, D. N. & THOMAS, S. H. L. 2007. Liver unit admission following paracetamol overdose with concentrations below current UK treatment thresholds. *QJM,* 100**,** 93-96.

BEHR, A. C., KWIATKOWSKI, A., STÅHLMAN, M., SCHMIDT, F. F., LUCKERT, C., BRAEUNING, A. & BUHRKE, T. 2020. Impairment of bile acid metabolism by perfluorooctanoic acid (PFOA) and perfluorooctanesulfonic acid (PFOS) in human HepaRG hepatoma cells. *Archives of Toxicology,* 94**,** 1673-1686.

BIRERDINC, A., STEPANOVA, M., PAWLOSKI, L. & YOUNOSSI, Z. M. 2012. Caffeine is protective in patients with non-alcoholic fatty liver disease. *Alimentary Pharmacology and Therapeutics,* 35**,** 76-82.

BLOND, E., RIEUSSET, J., ALLIGIER, M., LAMBERT-PORCHERON, S., BENDRIDI, N., GABERT, L., CHETIVEAUX, M., DEBARD, C., CHAUVIN, M. A., NORMAND, S., ROTH, H., DE GOUVILLE, A. C., KREMPF, M., VIDAL, H., GOUDABLE, J. & LAVILLE, M. 2014. Nicotinic acid effects on insulin sensitivity and hepatic lipid metabolism: An in vivo to in vitro study. *Hormone and Metabolic Research,* 46**,** 390-396.

BODIN, K., BRETILLON, L., ADEN, Y., BERTILSSON, L., BROOMÉ, U., EINARSSON, C. & DICZFALUSY, U. 2001. Antiepileptic drugs increase plasma levels of 4β-hydroxycholesterol in humans. Evidence for involvement of cytochrome P450 3A4. *Journal of Biological Chemistry,* 276**,** 38685-38689.

BOETTCHER, E., CSAKO, G., PUCINO, F., WESLEY, R. & LOOMBA, R. 2012. Meta-analysis: Pioglitazone improves liver histology and fibrosis in patients with non-alcoholic steatohepatitis. *Alimentary Pharmacology and Therapeutics,* 35**,** 66-75.

BOTELHO, S. C., SAGHAFIAN, M., PAVLOVA, S., HASSAN, M., DEPIERRE, J. W. & ABEDI-VALUGERDI, M. 2015. Complement activation is involved in the hepatic injury caused by high-dose exposure of mice to perfluorooctanoic acid. *Chemosphere,* 129**,** 225-31.

BOUDABA, N., MARION, A., HUET, C., PIERRE, R., VIOLLET, B. & FORETZ, M. 2018. AMPK Re-Activation Suppresses Hepatic Steatosis but its Downregulation Does Not Promote Fatty Liver Development. *EBioMedicine,* 28**,** 194-209.

BOUVET, R., CAUCHOIS, A., BAERT, A., FROMENTY, B., MOREL, I., TURLIN, B. & GICQUEL, T. 2020. Fatal acetaminophen poisoning with hepatic microvesicular steatosis in a child after repeated administration of therapeutic doses. *Forensic Science International,* 310.

BREEN, K. J., SCHENKER, S. & HEIMBERG, M. 1975. Fatty liver induced by tetracycline in the rat. Dose response relationships and effect of sex. *Gastroenterology,* 69**,** 714-723.

BUCHER, S., LE GUILLOU, D., ALLARD, J., PINON, G., BEGRICHE, K., TÊTE, A., SERGENT, O., LAGADIC-GOSSMANN, D. & FROMENTY, B. 2018a. Possible Involvement of Mitochondrial Dysfunction and Oxidative Stress in a Cellular Model of NAFLD Progression Induced by Benzo[a]pyrene/Ethanol CoExposure. *Oxidative Medicine and Cellular Longevity,* 2018.

BUCHER, S., TÊTE, A., PODECHARD, N., LIAMIN, M., LE GUILLOU, D., CHEVANNE, M., COULOUARN, C., IMRAN, M., GALLAIS, I., FERNIER, M., HAMDAOUI, Q., ROBIN, M.-A., SERGENT, O., FROMENTY, B. & LAGADIC-GOSSMANN, D. 2018b. Co-exposure to benzo[a]pyrene and ethanol induces a pathological progression of liver steatosis in vitro and in vivo. *Scientific reports,* 8**,** 5963-5963.

BUCKLEY, J. P., ENGEL, S. M., MENDEZ, M. A., RICHARDSON, D. B., DANIELS, J. L., CALAFAT, A. M., WOLFF, M. S. & HERRING, A. H. 2016. Prenatal phthalate exposures and childhood fat mass in a New York city cohort. *Environmental Health Perspectives,* 124**,** 507-513.

BUHRKE, T., KRÜGER, E., PEVNY, S., RÖßLER, M., BITTER, K. & LAMPEN, A. 2015. Perfluorooctanoic acid (PFOA) affects distinct molecular signalling pathways in human primary hepatocytes. *Toxicology,* 333**,** 53-62.

BUTENHOFF, J. L., GAYLOR, D. W., MOORE, J. A., OLSEN, G. W., RODRICKS, J., MANDEL, J. H. & ZOBEL, L. R. 2004. Characterization of risk for general population exposure to perfluorooctanoate. *Regul Toxicol Pharmacol,* 39**,** 363-80.

CALDWELL, S. H., PATRIE, J. T., BRUNT, E. M., REDICK, J. A., DAVIS, C. A., PARK, S. H. & NEUSCHWANDER-TETRI, B. A. 2007. The effects of 48 weeks of rosiglitazone on hepatocyte mitochondria in human nonalcoholic steatohepatitis. *Hepatology,* 46**,** 1101-1107.

CANET, M. J., MERRELL, M. D., HARDWICK, R. N., BATAILLE, A. M., CAMPION, S. N., FERREIRA, D. W., XANTHAKOS, S. A., MANAUTOU, J. E., A-KADER, H. H., ERICKSON, R. P. & CHERRINGTON, N. J. 2015. Altered regulation of hepatic efflux transporters disrupts acetaminophen disposition in pediatric nonalcoholic steatohepatitis. *Drug Metabolism and Disposition,* 43**,** 829-835.

CANO-SANCHO, G., SALMON, A. G. & LA MERRILL, M. A. 2017. Association between Exposure to p,p'-DDT and Its Metabolite p,p'-DDE with Obesity: Integrated Systematic Review and Meta-Analysis. *Environ Health Perspect,* 125**,** 096002.

CAPES-DAVIS, A., THEODOSOPOULOS, G., ATKIN, I., DREXLER, H. G., KOHARA, A., MACLEOD, R. A., MASTERS, J. R., NAKAMURA, Y., REID, Y. A., REDDEL, R. R. & FRESHNEY, R. I. 2010. Check your cultures! A list of cross-contaminated or misidentified cell lines. *Int J Cancer,* 127**,** 1-8.

CAPUZZI, D. M., MORGAN, J. M., BRUSCO JR, O. A. & INTENZO, C. M. 2000. Niacin dosing: relationship to benefits and adverse effects. *Current atherosclerosis reports,* 2**,** 64-71.

CDC DEPARTMENT OF HEALTH AND HUMAN SERVICES 2009. Fourth National Report on Human Exposure to Environmental Chemicals.

CHACHAY, V. S., MACDONALD, G. A., MARTIN, J. H., WHITEHEAD, J. P., O'MOORE-SULLIVAN, T. M., LEE, P., FRANKLIN, M., KLEIN, K., TAYLOR, P. J., FERGUSON, M., COOMBES, J. S., THOMAS, G. P., COWIN, G. J., KIRKPATRICK, C. M. J., PRINS, J. B. & HICKMAN, I. J. 2014. Resveratrol Does Not Benefit Patients With Nonalcoholic Fatty Liver Disease. *Clinical Gastroenterology and Hepatology,* 12**,** 2092-2103.e6.

CHAMORRO-GARCÍA, R. & BLUMBERG, B. 2014. Transgenerational effects of obesogens and the obesity epidemic. *Current Opinion in Pharmacology,* 19**,** 153-158.

CHAMORRO-GARCÍA, R., SAHU, M., ABBEY, R. J., LAUDE, J., PHAM, N. & BLUMBERG, B. 2013. Transgenerational inheritance of increased fat depot size, stem cell reprogramming, and hepatic steatosis elicited by prenatal exposure to the obesogen tributyltin in mice. *Environ Health Perspect,* 121**,** 359-66.

CHAMORRO-GARCÍA, R., SHOUCRI, B. M., WILLNER, S., KÄCH, H., JANESICK, A. & BLUMBERG, B. 2018. Effects of perinatal exposure to dibutyltin chloride on fat and glucose metabolism in mice, and molecular mechanisms, in vitro. *Environmental Health Perspectives,* 126.

CHANG, R., CHOU, M. C., HUNG, L. Y., WANG, M. E., HSU, M. C. & CHIU, C. H. 2016. Study of valproic acid-enhanced hepatocyte steatosis. *BioMed Research International,* 2016.

CHEN, G., JIN, Y., WU, Y., LIU, L. & FU, Z. 2015. Exposure of male mice to two kinds of organophosphate flame retardants (OPFRs) induced oxidative stress and endocrine disruption. *Environ Toxicol Pharmacol,* 40**,** 310-8.

CHEN, L., SHU, Y., LIANG, X., CHEN, E. C., YEE, S. W., ZUR, A. A., LI, S., XU, L., KESHARI, K. R., LIN, M. J., CHIEN, H. C., ZHANG, Y., MORRISSEY, K. M., LIU, J., OSTREM, J., YOUNGER, N. S., KURHANEWICZ, J., SHOKAT, K. M., ASHRAFI, K. & GIACOMINI, K. M. 2014. OCT1 is a high-capacity thiamine transporter that regulates hepatic steatosis and is a target of metformin. *Proceedings of the National Academy of Sciences of the United States of America,* 111**,** 9983-9988.

CHEN, Q., WANG, E., MA, L. & ZHAI, P. 2012. Dietary resveratrol increases the expression of hepatic 7-hydroxylase and ameliorates hypercholesterolemia in high-fat fed C57BL/6J mice. *Lipids in Health and Disease,* 11.

CHEN, W. M., SHAW, L. H., CHANG, P. J., TUNG, S. Y., CHANG, T. S., SHEN, C. H., HSIEH, Y. Y. & WEI, K. L. 2016. Hepatoprotective effect of resveratrol against ethanol-induced oxidative stress through induction of superoxide dismutase in vivo and in vitro. *Experimental and Therapeutic Medicine,* 11**,** 1231-1238.

CHEN, Y. P., LU, F. B., HU, Y. B., XU, L. M., ZHENG, M. H. & HU, E. D. 2019. A systematic review and a dose–response meta-analysis of coffee dose and nonalcoholic fatty liver disease. *Clinical Nutrition,* 38**,** 2552-2557.

CHENNAMSETTY, I., KOSTNER, K. M., CLAUDEL, T., VINOD, M., FRANK, S., WEISS, T. S., TRAUNER, M. & KOSTNER, G. M. 2012. Nicotinic acid inhibits hepatic APOA gene expression: Studies in humans and in transgenic mice. *Journal of Lipid Research,* 53**,** 2405-2412.

CHRØIS, K. M., LARSEN, S., PEDERSEN, J. S., RYGG, M. O., BOILSEN, A. E. B., BENDTSEN, F. & DELA, F. 2020. Acetaminophen toxicity induces mitochondrial complex I inhibition in human liver tissue. *Basic and Clinical Pharmacology and Toxicology,* 126**,** 86-91.

CLARKSON, A., CHOONARA, I. & MARCOVITCH, H. 2002. Surveillance for fatal suspected adverse drug reactions in the UK. *Archives of Disease in Childhood,* 87**,** 462-467.

COPPOLA, A., BRADY, P. G. & NORD, H. J. 1994. Niacin-induced hepatotoxicity: Unusual presentations. *Southern Medical Journal,* 87**,** 30-32.

CULLINAN, M. P., PALMER, J. E., FADDY, M. J., WESTERMAN, B., CARLE, A. D., WEST, M. J. & SEYMOUR, G. J. 2015. The Influence of Triclosan on Biomarkers of Cardiovascular Risk in Patients in the Cardiovascular and Periodontal Study (CAPS): A Randomized Controlled Trial. *J Periodontol,* 86**,** 847-55.

CUYKX, M., CLAES, L., RODRIGUES, R. M., VANHAECKE, T. & COVACI, A. 2018. Metabolomics profiling of steatosis progression in HepaRG® cells using sodium valproate. *Toxicology Letters,* 286**,** 22-30.

DARROW, L. A., GROTH, A. C., WINQUIST, A., SHIN, H. M., BARTELL, S. M. & STEENLAND, K. 2016. Modeled perfluorooctanoic acid (PFOA) exposure and liver function in a Mid-Ohio Valley community. *Environmental Health Perspectives,* 124**,** 1227-1233.

DAS, K. P., WOOD, C. R., LIN, M. T., STARKOV, A. A., LAU, C., WALLACE, K. B., CORTON, J. C. & ABBOTT, B. D. 2017. Perfluoroalkyl acids-induced liver steatosis: Effects on genes controlling lipid homeostasis. *Toxicology,* 378**,** 37-52.

DASARATHY, S., DASARATHY, J., KHIYAMI, A., YERIAN, L., HAWKINS, C., SARGENT, R. & MCCULLOUGH, A. J. 2015. Double-blind randomized placebo-controlled clinical trial of omega 3 fatty acids for the treatment of diabetic patients with nonalcoholic steatohepatitis. *Journal of Clinical Gastroenterology,* 49**,** 137-144.

DASH, R. P., BABU, R. J. & SRINIVAS, N. R. 2018. Non-alcoholic steatohepatitis (NASH) drug discovery – building a consensus on ADME screening tools and clinical pharmacology strategies to aid candidate development. *Journal of Pharmacy and Pharmaceutical Sciences,* 21**,** 481-495.

DEPNER, C. M., PHILBRICK, K. A. & JUMP, D. B. 2013a. Docosahexaenoic acid attenuates hepatic inflammation, oxidative stress, and fibrosis without decreasing hepatosteatosis in a Ldlr-/- Mouse Model of Western Diet-Induced Nonalcoholic Steatohepatitis1-3. *Journal of Nutrition,* 143**,** 315-323.

DEPNER, C. M., TRABER, M. G., BOBE, G., KENSICKI, E., BOHREN, K. M., MILNE, G. & JUMP, D. B. 2013b. A metabolomic analysis of omega-3 fatty acid-mediated attenuation of western diet-induced nonalcoholic steatohepatitis in LDLR-/- mice. *PLoS ONE,* 8.

DESVERGNE, B., FEIGE, J. N. & CASALS-CASAS, C. 2009. PPAR-mediated activity of phthalates: A link to the obesity epidemic? *Molecular and Cellular Endocrinology,* 304**,** 43-48.

DI MINNO, M. N. D., RUSSOLILLO, A., LUPOLI, R., AMBROSINO, P., DI MINNO, A. & TARANTINO, G. 2012. Omega-3 fatty acids for the treatment of non-alcoholic fatty liver disease. *World Journal of Gastroenterology,* 18**,** 5839-5847.

DO, M. T., CHANG, V. C., MENDEZ, M. A. & DE GROH, M. 2017. Urinary bisphenol a and obesity in adults: Results from the canadian health measures survey. *Health Promotion and Chronic Disease Prevention in Canada,* 37**,** 403-412.

DONATO, M. T., TOLOSA, L., JIMÉNEZ, N., CASTELL, J. V. & GÓMEZ-LECHÓN, M. J. 2012. High-content imaging technology for the evaluation of drug-induced steatosis using a multiparametric cell-based assay. *Journal of Biomolecular Screening,* 17**,** 394-400.

DONG, X., ZHANG, Y., DONG, J., ZHAO, Y., GUO, J., WANG, Z., LIU, M., NA, X. & WANG, C. 2017. Urinary metabolomic profiling in rats exposed to dietary di(2-ethylhexyl) phthalate (DEHP) using ultra-performance liquid chromatography quadrupole time-of-flight tandem mass spectrometry (UPLC/Q-TOF-MS). *Environmental Science and Pollution Research,* 24**,** 16659-16672.

DRIESSEN, M., VITINS, A. P., PENNINGS, J. L. A., KIENHUIS, A. S., WATER, B. V. D. & VAN DER VEN, L. T. M. 2015. A transcriptomics-based hepatotoxicity comparison between the zebrafish embryo and established human and rodent in vitro and in vivo models using cyclosporine A, amiodarone and acetaminophen. *Toxicology Letters,* 232**,** 403-412.

DU, Z., ZHANG, Y., WANG, G., PENG, J., WANG, Z. & GAO, S. 2016. TPhP exposure disturbs carbohydrate metabolism, lipid metabolism, and the DNA damage repair system in zebrafish liver. *Scientific Reports,* 6.

DUAN, L., RAMACHANDRAN, A., AKAKPO, J. Y., WOOLBRIGHT, B. L., ZHANG, Y. & JAESCHKE, H. 2020. Mice deficient in pyruvate dehydrogenase kinase 4 are protected against acetaminophen-induced hepatotoxicity. *Toxicology and Applied Pharmacology,* 387.

EC 2018. Commission Implementing Regulation (EU) 2018/785 of 29 May 2018 amending Implementing Regulation (EU) No 540/2011 as regards the conditions of approval of the active substance thiamethoxam (Text with EEA relevance.) C/2018/3178. Document 32018R0785. Official Journal of the European Union.

ECHA & ANSES 2019. Analysis of the most appropriate risk management option (RMOA). Justification for the selection of a candidate CoRAP substance. ANSES on behalf of FR-MSCA.

EFSA 2009. Scientific Opinion on Risk Assessment for a Selected Group of Pesticides from the Triazole Group to Test Possible Methodologies to Assess Cumulative Effects from Exposure through Food from these Pesticides on Human Health. EFSA Panel on Plant Protection Products and their Residues. *EFSA Journal,* 7**,** 1167.

EFSA 2015. Scientific Opinion on the risks to public health related to the presence of bisphenol A (BPA) in foodstuffs. EFSA Panel on Food Contact Materials, Enzymes, Flavourings, Processing Aids and Panel on Dietetic Products, Nutrition. *EFSA Journal,* 13**,** 3978.

EFSA 2016. Peer review of the pesticide risk assessment of the active substance acetamiprid. *EFSA Journal,* 14.

EFSA 2019a. Peer review of the pesticide risk assessment of the active substance thiacloprid. EFSA-Q-2014-00849, EFSA-Q-2018-00136. *EFSA Journal,* 17.

EFSA 2019b. Statement on the available outcomes of the human health assessment in the context of the pesticides peer review of the active substance chlorpyrifos. EFSA-Q-2019-00414. *EFSA Journal,* 17**,** 23.

EHRLICH, A., TSYTKIN-KIRSCHENZWEIG, S., IOANNIDIS, K., AYYASH, M., RIU, A., NOTE, R., OUEDRAOGO, G., VANFLETEREN, J., COHEN, M. & NAHMIAS, Y. 2018. Microphysiological flux balance platform unravels the dynamics of drug induced steatosis. *Lab on a Chip,* 18**,** 2510-2522.

ELLINGROD, V. L., TAYLOR, S. F., DALACK, G., GROVE, T. B., BLY, M. J., BROOK, R. D., ZÖLLNER, S. K. & POP-BUSUI, R. 2012. Risk factors associated with metabolic syndrome in bipolar and schizophrenia subjects treated with antipsychotics: The role of folate pharmacogenetics. *Journal of Clinical Psychopharmacology,* 32**,** 261-265.

ELPHICK, L. M., PAWOLLECK, N., GUSCHINA, I. A., CHAIEB, L., EIKEL, D., NAU, H., HARWOOD, J. L., PLANT, N. J. & WILLIAMS, R. S. B. 2012. Conserved valproic-acid-induced lipid droplet formation in Dictyostelium and human hepatocytes identifies structurally active compounds. *DMM Disease Models and Mechanisms,* 5**,** 231-240.

EMA 2013. European Medicines Agency recommends suspension of marketing authorisations for oral ketoconazole. Benefit of oral ketoconazole does not outweigh risk of liver injury in fungal infections. *In:* AGENCY, E. M. (ed.).

ERIKSEN, K. T., RAASCHOU-NIELSEN, O., MCLAUGHLIN, J. K., LIPWORTH, L., TJØNNELAND, A., OVERVAD, K. & SØRENSEN, M. 2013. Association between plasma PFOA and PFOS levels and total cholesterol in a middle-aged Danish population. *PLoS One,* 8**,** e56969.

ERSÖZ, G., GÜNŞAR, F., KARASU, Z., AKAY, S., BATUR, Y. & AKARCA, U. S. 2005. Management of fatty liver disease with vitamin E and C compared to ursodeoxycholic acid treatment. *Turkish Journal of Gastroenterology,* 16**,** 124-128.

FABBRINI, E., MOHAMMED, B. S., KORENBLAT, K. M., MAGKOS, F., MCCREA, J., PATTERSON, B. W. & KLEIN, S. 2010. Effect of fenofibrate and niacin on intrahepatic triglyceride content, very low-density lipoprotein kinetics, and insulin action in obese subjects with nonalcoholic fatty liver disease. *Journal of Clinical Endocrinology and Metabolism,* 95**,** 2727-2735.

FAGHIHI, T., JAHED, A., MAHMOUDI-GHARAEI, J., SHARIFI, V., AKHONDZADEH, S. & GHAELI, P. 2012. Role of Omega-3 fatty acids in preventing metabolic disturbances in patients on olanzapine plus either sodium valproate or lithium: A randomized double-blind placebo-controlled trial. *DARU, Journal of Pharmaceutical Sciences,* 20.

FAO/WHO JOINT MEETING ON PESTICIDE RESIDUES 2009. Chlorpyrifos-methyl (090). Food and Agriculture Organization of the United Nations.

FAO/WHO JOINT MEETING ON PESTICIDE SPECIFICATIONS 2009. FAO Specifications and Evaluations for Agricultural Pesticides. Chlorpyrifos. O,O-diethyl O-3,5,6-trichloro-2-pyridyl phosphorothioate. Food and Agriculture Organization of the United Nations.

FAOUZI, M. A., DINE, T., GRESSIER, B., KAMBIA, K., LUYCKX, M., PAGNIEZ, D., BRUNET, C., CAZIN, M., BELABED, A. & CAZIN, J. C. 1999. Exposure of hemodialysis patients to di-2-ethylhexyl phthalate. *International Journal of Pharmaceutics,* 180**,** 113-121.

FEARS, R. 1978. The hypercholesterolaemic effect of caffeine in rats fed on diets with and without supplementary cholesterol. *British Journal of Nutrition,* 39**,** 363-374.

FEIGE, J. N., GELMAN, L., ROSSI, D., ZOETE, V., MÉTIVIER, R., TUDOR, C., ANGHEL, S. I., GROSDIDIER, A., LATHION, C., ENGELBORGHS, Y., MICHIELIN, O., WAHLI, W. & DESVERGNE, B. 2007. The endocrine disruptor monoethyl-hexyl-phthalate is a selective peroxisome proliferator-activated receptor gamma modulator that promotes adipogenesis. *J Biol Chem,* 282**,** 19152-66.

FEIGE, J. N., GERBER, A., CASALS-CASAS, C., YANG, Q., WINKLER, C., BEDU, E., BUENO, M., GELMAN, L., AUWERX, J., GONZALEZ, F. J. & DESVERGNE, B. 2010. The pollutant diethylhexyl phthalate regulates hepatic energy metabolism via species-specific PPARα-dependent mechanisms. *Environmental Health Perspectives,* 118**,** 234-241.

FOSTER, T., BUDOFF, M. J., SAAB, S., AHMADI, N., GORDON, C. & GUERCI, A. D. 2011. Atorvastatin and antioxidants for the treatment of nonalcoholic fatty liver disease: The st francis heart study randomized clinical trial. *American Journal of Gastroenterology,* 106**,** 71-77.

FRANCO, M. E., FERNANDEZ-LUNA, M. T., RAMIREZ, A. J. & LAVADO, R. 2020. Metabolomic-based assessment reveals dysregulation of lipid profiles in human liver cells exposed to environmental obesogens. *Toxicology and Applied Pharmacology,* 398.

FROMENTY, B. 2019. Inhibition of mitochondrial fatty acid oxidation in drug-induced hepatic steatosis. *Liver Research,* 3**,** 157-169.

FROMENTY, B., FISCH, C., BERSON, A., LETTERON, P., LARREY, D. & PESSAYRE, D. 1990. Dual effect of amiodarone on mitochondrial respiration. Initial protonophoric uncoupling effect followed by inhibition of the respiratory chain at the levels of complex I and complex II. *J Pharmacol Exp Ther,* 255**,** 1377-84.

FUSTINONI, S., MERCADANTE, R., POLLEDRI, E., RUBINO, F. M., MANDIC-RAJCEVIC, S., VIANELLO, G., COLOSIO, C. & MORETTO, A. 2014. Biological monitoring of exposure to tebuconazole in winegrowers. *J Expo Sci Environ Epidemiol,* 24**,** 643-9.

GANJI, S. H., KASHYAP, M. L. & KAMANNA, V. S. 2015. Niacin inhibits fat accumulation, oxidative stress, and inflammatory cytokine IL-8 in cultured hepatocytes: Impact on non-alcoholic fatty liver disease. *Metabolism: Clinical and Experimental,* 64**,** 982-990.

GAO, M., MA, Y., ALSAGGAR, M. & LIU, D. 2016. Dual Outcomes of Rosiglitazone Treatment on Fatty Liver. *Aaps j,* 18**,** 1023-31.

GAO, Q., WANG, X. Y., ZHOU, J. & FAN, J. 2011. Cell line misidentification: the case of the Chang liver cell line. *Hepatology,* 54**,** 1894-5.

GARCÍA-ROMÁN, R. & FRANCÉS, R. 2020. Acetaminophen-Induced Liver Damage in Hepatic Steatosis. *Clinical Pharmacology and Therapeutics,* 107**,** 1068-1081.

GARCÍA RODRÍGUEZ, L. A., DUQUE, A., CASTELLSAGUE, J., PÉREZ-GUTTHANN, S. & STRICKER, B. H. 1999. A cohort study on the risk of acute liver injury among users of ketoconazole and other antifungal drugs. *Br J Clin Pharmacol,* 48**,** 847-52.

GARINIS, G. A., FRUCI, B., MAZZA, A., DE SIENA, M., ABENAVOLI, S., GULLETTA, E., VENTURA, V., GRECO, M., ABENAVOLI, L. & BELFIORE, A. 2010. Metformin versus dietary treatment in nonalcoholic hepatic steatosis: A randomized study. *International Journal of Obesity,* 34**,** 1255-1264.

GAROCHE, C., BOULAHTOUF, A., GRIMALDI, M., CHIAVARINA, B., TOPOROVA, L., DEN BROEDER, M. J., LEGLER, J., BOURGUET, W. & BALAGUER, P. 2021. Interspecies Differences in Activation of Peroxisome Proliferator-Activated Receptor γ by Pharmaceutical and Environmental Chemicals. *Environ Sci Technol,* 55**,** 16489-16501.

GARTLER, S. M. 1968. Apparent Hela cell contamination of human heteroploid cell lines. *Nature,* 217**,** 750-1.

GODA, K., SAITO, K., MUTA, K., KOBAYASHI, A., SAITO, Y. & SUGAI, S. 2018. Ether-phosphatidylcholine characterized by consolidated plasma and liver lipidomics is a predictive biomarker for valproic acid-induced hepatic steatosis. *Journal of Toxicological Sciences,* 43**,** 395-405.

GÓMEZ-LECHÓN, M. J., TOLOSA, L., CASTELL, J. V. & DONATO, M. T. 2010. Mechanism-based selection of compounds for the development of innovative in vitro approaches to hepatotoxicity studies in the LIINTOP project. *Toxicology in Vitro,* 24**,** 1879-1889.

GONG, Y., WANG, G., GONG, Y., YAN, J., CHEN, Y. & BURCZYNSKI, F. J. 2014. Hepatoprotective role of liver fatty acid binding protein in acetaminophen induced toxicity. *BMC Gastroenterology,* 14.

GRAETER, T., NIEDERMAYER, P. C., MASON, R. A., OEZTUERK, S., HAENLE, M. M., KOENIG, W., BOEHM, B. O., KRATZER, W. & GROUP, E. M.-S. 2015. Coffee consumption and NAFLD: a community based study on 1223 subjects. *BMC Research Notes,* 8.

GREEN, C. J., PRAMFALK, C., CHARLTON, C. A., GUNN, P. J., CORNFIELD, T., PAVLIDES, M., KARPE, F. & HODSON, L. 2020. Hepatic de novo lipogenesis is suppressed and fat oxidation is increased by omega-3 fatty acids at the expense of glucose metabolism. *BMJ open diabetes research & care,* 8.

GRINBERG, M., STÖBER, R. M., EDLUND, K., REMPEL, E., GODOY, P., REIF, R., WIDERA, A., MADJAR, K., SCHMIDT-HECK, W., MARCHAN, R., SACHINIDIS, A., SPITKOVSKY, D., HESCHELER, J., CARMO, H., ARBO, M. D., VAN DE WATER, B., WINK, S., VINKEN, M., ROGIERS, V., ESCHER, S., HARDY, B., MITIC, D., MYATT, G., WALDMANN, T., MARDINOGLU, A., DAMM, G., SEEHOFER, D., NÜSSLER, A., WEISS, T. S., OBEREMM, A., LAMPEN, A., SCHAAP, M. M., LUIJTEN, M., VAN STEEG, H., THASLER, W. E., KLEINJANS, J. C. S., STIERUM, R. H., LEIST, M., RAHNENFÜHRER, J. & HENGSTLER, J. G. 2014. Toxicogenomics directory of chemically exposed human hepatocytes. *Archives of Toxicology,* 88**,** 2261-2287.

GRÜN, F., WATANABE, H., ZAMANIAN, Z., MAEDA, L., ARIMA, K., CUBACHA, R., GARDINER, D. M., KANNO, J., IGUCHI, T. & BLUMBERG, B. 2006. Endocrine-disrupting organotin compounds are potent inducers of adipogenesis in vertebrates. *Molecular Endocrinology,* 20**,** 2141-2155.

GRÜNIG, D., SZABO, L., MARBET, M. & KRÄHENBÜHL, S. 2020. Valproic acid affects fatty acid and triglyceride metabolism in HepaRG cells exposed to fatty acids by different mechanisms. *Biochemical Pharmacology,* 177**,** 113860.

GU, X., LUO, X., WANG, Y., HE, Z., LI, X., WU, K., ZHANG, Y., YANG, Y., JI, J. & LUO, X. 2019. Ascorbic acid attenuates cell stress by activating the fibroblast growth factor 21/fibroblast growth factor receptor 2/adiponectin pathway in HepG2 cells. *Molecular Medicine Reports,* 20**,** 2450-2458.

HAEGLER, P., JOERIN, L., KRÄHENBÜHL, S. & BOUITBIR, J. 2017. Hepatocellular Toxicity of Imidazole and Triazole Antimycotic Agents. *Toxicol Sci,* 157**,** 183-195.

HAJIGHASEM, A., FARZANEGI, P., MAZAHERI, Z., NAGHIZADEH, M. & SALEHI, G. 2018. Effects of resveratrol, exercises and their combination on Farnesoid X receptor, Liver X receptor and Sirtuin 1 gene expression and apoptosis in the liver of elderly rats with nonalcoholic fatty liver. *PeerJ,* 2018.

HALLE, W., HALDER, M., WORTH, A. & GENSCHOW, E. 2003. The Registry of Cytotoxicity: Toxicity Testing in Cell Cultures to Predict Acute Toxicity (LD50) and to Reduce Testing in Animals. *Alternatives to Laboratory Animals,* 31**,** 89-89.

HARRISON, S. A., TORGERSON, S., HAYASHI, P., WARD, J. & SCHENKER, S. 2003. Vitamin E and Vitamin C Treatment Improves Fibrosis in Patients with Nonalcoholic Steatohepatitis. *American Journal of Gastroenterology,* 98**,** 2485-2490.

HAYAT, U., SIDDIQUI, A. A., OKUT, H., AFROZ, S., TASLEEM, S. & HARIS, A. 2021. The effect of coffee consumption on the non-alcoholic fatty liver disease and liver fibrosis: A meta-analysis of 11 epidemiological studies. *Annals of Hepatology,* 20.

HPS2-THRIVE COLLABORATIVE GROUP 2013. HPS2-THRIVE randomized placebo-controlled trial in 25 673 high-risk patients of ER niacin/laropiprant: trial design, pre-specified muscle and liver outcomes, and reasons for stopping study treatment. *Eur Heart J,* 34**,** 1279-91.

HUANG, B., BAO, J., CAO, Y. R., GAO, H. F. & JIN, Y. 2018. Cytochrome P450 1A1 (CYP1A1) Catalyzes Lipid Peroxidation of Oleic Acid-Induced HepG2 Cells. *Biochemistry (Moscow),* 83**,** 595-602.

HUC, L., LEMARIÉ, A., GUÉRAUD, F. & HÉLIÈS-TOUSSAINT, C. 2012. Low concentrations of bisphenol A induce lipid accumulation mediated by the production of reactive oxygen species in the mitochondria of HepG2 cells. *Toxicology in Vitro,* 26**,** 709-717.

IDA, S., KANEKO, R. & MURATA, K. 2019. Efficacy and safety of pemafibrate administration in patients with dyslipidemia: A systematic review and meta-analysis. *Cardiovascular Diabetology,* 18.

IPSEN, D. H., TVEDEN-NYBORG, P. & LYKKESFELDT, J. 2014. Does vitamin C deficiency promote fatty liver disease development? *Nutrients,* 6**,** 5473-5499.

ISENBERG, J. S. & KLAUNIG, J. E. 2000. Role of the mitochondrial membrane permeability transition (MPT) in rotenone-induced apoptosis in liver cells. *Toxicol Sci,* 53**,** 340-51.

ISHIBASHI, S., ARAI, H., YOKOTE, K., ARAKI, E., SUGANAMI, H., YAMASHITA, S. & GROUP, K. S. 2018. Efficacy and safety of pemafibrate (K-877), a selective peroxisome proliferator-activated receptor α modulator, in patients with dyslipidemia: Results from a 24-week, randomized, double blind, active-controlled, phase 3 trial. *Journal of Clinical Lipidology,* 12**,** 173-184.

IVANCOVSKY-WAJCMAN, D., FLISS-ISAKOV, N., SALOMONE, F., WEBB, M., SHIBOLET, O., KARIV, R. & ZELBER-SAGI, S. 2019. Dietary vitamin E and C intake is inversely associated with the severity of nonalcoholic fatty liver disease. *Digestive and Liver Disease,* 51**,** 1698-1705.

IZDEBSKA, M., HERBET, M., GAWROŃSKA-GRZYWACZ, M., PIĄTKOWSKA-CHMIEL, I., KORGA, A., SYSA, M., IWAN, M., NATORSKA-CHOMICKA, D., POLESZAK, E., WRÓBEL, A., MANDZIUK, S. & DUDKA, J. 2018. Resveratrol limits lipogenesis and enhance mitochondrial activity in HepG2 cells. *Journal of Pharmacy and Pharmaceutical Sciences,* 21**,** 504-515.

JAKUBCZYK, K., SKONIECZNA-ŻYDECKA, K., KAŁDUŃSKA, J., STACHOWSKA, E., GUTOWSKA, I. & JANDA, K. 2020. Effects of resveratrol supplementation in patients with non-alcoholic fatty liver disease—A meta-analysis. *Nutrients,* 12**,** 1-15.

JENNINGS, P., SCHWARZ, M., LANDESMANN, B., MAGGIONI, S., GOUMENOU, M., BOWER, D., LEONARD, M. O. & WISEMAN, J. S. 2014. SEURAT-1 liver gold reference compounds: a mechanism-based review. *Archives of Toxicology,* 88**,** 2099-2133.

JETTEN, M. J. A., GAJ, S., RUIZ-ARACAMA, A., DE KOK, T. M., VAN DELFT, J. H. M., LOMMEN, A., VAN SOMEREN, E. P., JENNEN, D. G. J., CLAESSEN, S. M., PEIJNENBURG, A. A. C. M., STIERUM, R. H. & KLEINJANS, J. C. S. 2012. 'Omics analysis of low dose acetaminophen intake demonstrates novel response pathways in humans. *Toxicology and Applied Pharmacology,* 259**,** 320-328.

JOINT FAO/WHOA MEETING ON PESTICIDE RESIDUES 2006. Pesticide residues in food 2006. *FAO Plant Production and Protection Paper 187.* Rome: World Health Organization, Food and Agriculture Organization of the United Nations.

KARVELLAS, C. J., SPEISER, J. L., TREMBLAY, M., LEE, W. M., ROSE, C. F. & FOR THE, U. S. A. L. F. S. G. 2017. The association between FABP7 serum levels with survival and neurological complications in acetaminophen-induced acute liver failure: a nested case–control study. *Annals of Intensive Care,* 7.

KATSURA, A., MORISHITA, A., IWAMA, H., TANI, J., SAKAMOTO, T., TATSUTA, M., TOYOTA, Y., FUJITA, K., KATO, K., MAEDA, E., NOMURA, T., MIYOSHI, H., YONEYAMA, H., HIMOTO, T., FUJIWARA, S., KOBARA, H., MORI, H., NIKI, T., ONO, M., HIRASHIMA, M. & MASAKI, T. 2015. MicroRNA profiles following metformin treatment in a mouse model of non-alcoholic steatohepatitis. *International Journal of Molecular Medicine,* 35**,** 877-884.

KATZ, T. A., GRIMM, S. L., KAUSHAL, A., DONG, J., TREVIÑO, L. S., JANGID, R. K., GAITÁN, A. V., BERTOCCHIO, J. P., GUAN, Y., ROBERTSON, M. J., CABRERA, R. M., FINEGOLD, M. J., FOULDS, C. E., COARFA, C. & WALKER, C. L. 2020. Hepatic tumor formation in adult mice developmentally exposed to organotin. *Environmental Health Perspectives,* 128.

KENNEDY JR, G. L., BUTENHOFF, J. L., OLSEN, G. W., O'CONNOR, J. C., SEACAT, A. M., PERKINS, R. G., BIEGEL, L. B., MURPHY, S. R. & FARRAR, D. G. 2004. The toxicology of perfluorooctanoate. *Critical Reviews in Toxicology,* 34**,** 351-384.

KESSOKU, T., IMAJO, K., HONDA, Y., KATO, T., OGAWA, Y., TOMENO, W., KATO, S., MAWATARI, H., FUJITA, K., YONEDA, M., NAGASHIMA, Y., SAITO, S., WADA, K. & NAKAJIMA, A. 2016. Resveratrol ameliorates fibrosis and inflammation in a mouse model of nonalcoholic steatohepatitis. *Scientific Reports,* 6.

KHALDOUN OULARBI, H., RICHEVAL, C., LEBAILI, N., ZERROUKI-DAOUDI, N., BAHA, M., DJENNAS, N. & ALLORGE, D. 2017. Ameliorative effect of Vitamin C against hepatotoxicity induced by emamectin benzoate in rats. *Human and Experimental Toxicology,* 36**,** 709-717.

KILCHOER, B., VILS, A., MINDER, B., MUKA, T., GLISIC, M. & BALLY, L. 2020. Efficacy of dietary supplements to reduce liver fat. *Nutrients,* 12**,** 1-16.

KIM, A. J., SHI, Y., AUSTIN, R. C. & WERSTUCK, G. H. 2005. Valproate protects cells fom ER stress-induced lipid accumulation and apoptosis by inhibiting glycogen synthase kinase-3. *Journal of Cell Science,* 118**,** 89-99.

KIM, C., HARLOW, S. D., KARVONEN-GUTIERREZ, C. A., RANDOLPH, J. F., HELMUTH, M., KONG, S., NAN, B. & CARLOS, R. 2013. Racial/ethnic differences in hepatic steatosis in a population-based cohort of post-menopausal women: The Michigan Study of Women's Health Across the Nation. *Diabetic Medicine,* 30**,** 1433-1441.

KIM, D., YOO, E. R., LI, A. A., CHOLANKERIL, G., TIGHE, S. P., KIM, W., HARRISON, S. A. & AHMED, A. 2019. Elevated urinary bisphenol A levels are associated with non-alcoholic fatty liver disease among adults in the United States. *Liver International,* 39**,** 1335-1342.

KJÆR, T. N., ORNSTRUP, M. J., POULSEN, M. M., STØDKILDE-JØRGENSEN, H., JESSEN, N., JØRGENSEN, J. O. L., RICHELSEN, B. & PEDERSEN, S. B. 2017. No beneficial effects of resveratrol on the metabolic syndrome: A randomized placebo-controlled clinical trial. *Journal of Clinical Endocrinology and Metabolism,* 102**,** 1642-1651.

KNEBEL, C., BUHRKE, T., SÜSSMUTH, R., LAMPEN, A., MARX-STOELTING, P. & BRAEUNING, A. 2019a. Pregnane X receptor mediates steatotic effects of propiconazole and tebuconazole in human liver cell lines. *Archives of Toxicology,* 93**,** 1311-1322.

KNEBEL, C., HEISE, T., ZANGER, U. M., LAMPEN, A., MARX-STOELTING, P. & BRAEUNING, A. 2019b. The azole fungicide tebuconazole affects human CYP1A1 and CYP1A2 expression by an aryl hydrocarbon receptor-dependent pathway. *Food Chem Toxicol,* 123**,** 481-491.

KOLENA, B., PETROVICOVÁ, I., ŠIDLOVSKÁ, M., PILKA, T., NEUSCHLOVÁ, M., VALENTOVÁ, I., RYBANSKÝ, L. & TRNOVEC, T. 2017. Occupational phthalate exposure and health outcomes among hairdressing apprentices. *Human and Experimental Toxicology,* 36**,** 1100-1112.

KOZYRA, M., JOHANSSON, I., NORDLING, Å., ULLAH, S., LAUSCHKE, V. M. & INGELMAN-SUNDBERG, M. 2018. Human hepatic 3D spheroids as a model for steatosis and insulin resistance. *Scientific Reports,* 8.

KRATOCHVIL, I., HOFMANN, T., ROTHER, S., SCHLICHTING, R., MORETTI, R., SCHARNWEBER, D., HINTZE, V., ESCHER, B. I., MEILER, J., KALKHOF, S. & VON BERGEN, M. 2019. Mono(2-ethylhexyl) phthalate (MEHP) and mono(2-ethyl-5-oxohexyl) phthalate (MEOHP) but not di(2-ethylhexyl) phthalate (DEHP) bind productively to the peroxisome proliferator-activated receptor γ. *Rapid Communications in Mass Spectrometry,* 33**,** 75-85.

KRISTENSEN, T. & OLCOTT, E. W. 1999. Effects of niacin therapy that simulate neoplasia: Hepatic steatosis with concurrent hepatic dysfunction. *Journal of Computer Assisted Tomography,* 23**,** 314-317.

KUČERA, O., AL-DURY, S., LOTKOVÁ, H., ROǓAR, T., RYCHTRMOC, D. & ČERVINKOVÁ, Z. 2012. Steatotic rat hepatocytes in primary culture are more susceptible to the acute toxic effect of acetaminophen. *Physiological Research,* 61**,** S93-S101.

KUTTNER, C. S., MANCINA, R., WAGENPFEIL, G., LAMMERT, F. & STOKES, C. S. 2019. Four-Week Omega-3 Supplementation in Carriers of the Prosteatotic PNPLA3 p.I148M Genetic Variant: An Open-Label Study. *Lifestyle Genomics,* 12**,** 10-17.

LA MERRILL, M. A., JOHNSON, C. L., SMITH, M. T., KANDULA, N. R., MACHERONE, A., PENNELL, K. D. & KANAYA, A. M. 2019. Exposure to Persistent Organic Pollutants (POPs) and Their Relationship to Hepatic Fat and Insulin Insensitivity among Asian Indian Immigrants in the United States. *Environmental Science and Technology,* 53**,** 13906-13918.

LANÇON, A., HANET, N., JANNIN, B., DELMAS, D., HEYDEL, J. M., LIZARD, G., CHAGNON, M. C., ARTUR, Y. & LATRUFFE, N. 2007. Resveratrol in human hepatoma HepG2 cells: Metabolism and inducibility of detoxifying enzymes. *Drug Metabolism and Disposition,* 35**,** 699-703.

LANDESMANN, B., HARRIS, G., LISKA, R. & WHELAN, M. Hepatotoxicity Screening Taking a Mode-Of-Action Approach Using HepaRG Cells and HCA. 2012.

LAPINSKAS, P. J., BROWN, S., LEESNITZER, L. M., BLANCHARD, S., SWANSON, C., CATTLEY, R. C. & CORTON, J. C. 2005. Role of PPARα in mediating the effects of phthalates and metabolites in the liver. *Toxicology,* 207**,** 149-163.

LARSON, A. M. 2007. Acetaminophen Hepatotoxicity. *Clinics in Liver Disease,* 11**,** 525-548.

LASCH, A., ALARCAN, J., LAMPEN, A., BRAEUNING, A. & LICHTENSTEIN, D. 2020. Combinations of LXR and RXR agonists induce triglyceride accumulation in human HepaRG cells in a synergistic manner. *Arch Toxicol,* 94**,** 1303-1320.

LATORRE, J., ORTEGA, F. J., LIÑARES-POSE, L., MORENO-NAVARRETE, J. M., LLUCH, A., COMAS, F., OLIVERAS-CAÑELLAS, N., RICART, W., HÖRING, M., ZHOU, Y., LIEBISCH, G., NIDHINA HARIDAS, P. A., OLKKONEN, V. M., LÓPEZ, M. & FERNÁNDEZ-REAL, J. M. 2020. Compounds that modulate AMPK activity and hepatic steatosis impact the biosynthesis of microRNAs required to maintain lipid homeostasis in hepatocytes: Hepatic microRNAs and lipid homeostasis. *EBioMedicine,* 53.

LAWRENCE, S. P. 1993. Transient focal hepatic defects related to sustained-release niacin. *Journal of Clinical Gastroenterology,* 16**,** 234-236.

LE MAGUERESSE-BATTISTONI, B., MULTIGNER, L., BEAUSOLEIL, C. & ROUSSELLE, C. 2018. Effects of bisphenol A on metabolism and evidences of a mode of action mediated through endocrine disruption. *Molecular and Cellular Endocrinology,* 475**,** 74-91.

LEE, H., AHN, J., SHIN, S. S. & YOON, M. 2019a. Ascorbic acid inhibits visceral obesity and nonalcoholic fatty liver disease by activating peroxisome proliferator-activated receptor α in high-fat-diet-fed C57BL/6J mice. *International Journal of Obesity,* 43**,** 1620-1630.

LEE, J., HONG, S. W., KWON, H., PARK, S. E., RHEE, E. J., PARK, C. Y., OH, K. W., PARK, S. W. & LEE, W. Y. 2019b. Resveratrol, an activator of SIRT1, improves ER stress by increasing clusterin expression in HepG2 cells. *Cell Stress and Chaperones,* 24**,** 825-833.

LEE, S. W., BAEK, S. M., KANG, K. K., LEE, A. R., KIM, T. U., CHOI, S. K., ROH, Y. S., HONG, I. H., PARK, S. J., KIM, T. H., JEONG, K. S. & PARK, J. K. 2021. Vitamin C Deficiency Inhibits Nonalcoholic Fatty Liver Disease Progression through Impaired de Novo Lipogenesis. *American Journal of Pathology,* 191**,** 1550-1563.

LI, F., XIANG, B., JIN, Y., LI, C., REN, S., WU, Y., LI, J. & LUO, Q. 2020. Hepatotoxic effects of inhalation exposure to polycyclic aromatic hydrocarbons on lipid metabolism of C57BL/6 mice. *Environment International,* 134.

LI, H., TOTH, E. & CHERRINGTON, N. J. 2018. Asking the right questions with animal models: Methionine- and choline-deficient model in predicting adverse drug reactions in human NASH. *Toxicological Sciences,* 161**,** 23-33.

LI, R., QIN, X., LIANG, X., LIU, M. & ZHANG, X. 2019. Lipidomic characteristics and clinical findings of epileptic patients treated with valproic acid. *Journal of Cellular and Molecular Medicine,* 23**,** 6017-6023.

LI, S., GUO, J., YING, Z., CHEN, S., YANG, L., CHEN, K., LONG, Q., QIN, D., PEI, D. & LIU, X. 2015a. Valproic acid-induced hepatotoxicity in alpers syndrome is associated with mitochondrial permeability transition pore opening-dependent apoptotic sensitivity in an induced pluripotent stem cell model. *Hepatology,* 61**,** 1730-1739.

LI, Y., WONG, K., GILES, A., JIANG, J., LEE, J. W., ADAMS, A. C., KHARITONENKOV, A., YANG, Q., GAO, B., GUARENTE, L. & ZANG, M. 2014. Hepatic SIRT1 attenuates hepatic steatosis and controls energy balance in mice by inducing fibroblast growth factor 21. *Gastroenterology,* 146**,** 539-549.e7.

LI, Y. H., YANG, L. H., SHA, K. H., LIU, T. G., ZHANG, L. G. & LIU, X. X. 2015b. Efficacy of poly-unsaturated fatty acid therapy on patients with nonalcoholic steatohepatitis. *World Journal of Gastroenterology,* 21**,** 7008-7013.

LICHTENSTEIN, D., MENTZ, A., SCHMIDT, F. F., LUCKERT, C., BUHRKE, T., MARX-STOELTING, P., KALINOWSKI, J., ALBAUM, S. P., JOOS, T. O., POETZ, O. & BRAEUNING, A. 2020. Transcript and protein marker patterns for the identification of steatotic compounds in human HepaRG cells. *Food and Chemical Toxicology,* 145**,** 111690.

LIM, K., HAN, C., DAI, Y., SHEN, M. & WU, T. 2009. Omega-3 polyunsaturated fatty acids inhibit hepatocellular carcinoma cell growth through blocking β-catenin and cyclooxygenase-2. *Molecular Cancer Therapeutics,* 8**,** 3046-3055.

LIMONCIEL, A., ASCHAUER, L., WILMES, A., PRAJCZER, S., LEONARD, M. O., PFALLER, W. & JENNINGS, P. 2011. Lactate is an ideal non-invasive marker for evaluating temporal alterations in cell stress and toxicity in repeat dose testing regimes. *Toxicology in Vitro,* 25**,** 1855-1862.

LINGVAY, I., RASKIN, P. & SZCZEPANIAK, L. S. 2007. Effect of insulin-metformin combination on hepatic steatosis in patients with type 2 diabetes. *Journal of Diabetes and its Complications,* 21**,** 137-142.

LOOMBA, R., LUTCHMAN, G., KLEINER, D. E., RICKS, M., FELD, J. J., BORG, B. B., MODI, A., NAGABHYRU, P., SUMNER, A. E., LIANG, T. J. & HOOFNAGLE, J. H. 2009. Clinical trial: Pilot study of metformin for the treatment of non-alcoholic steatohepatitis. *Alimentary Pharmacology and Therapeutics,* 29**,** 172-182.

LÓPEZ-RIERA, M., CONDE, I., TOLOSA, L., ZARAGOZA, Á., CASTELL, J. V., GÓMEZ-LECHÓN, M. J. & JOVER, R. 2017. New microRNA biomarkers for drug-induced steatosis and their potential to predict the contribution of drugs to non-alcoholic fatty liver disease. *Frontiers in Pharmacology,* 8.

LUCKERT, C., BRAEUNING, A., DE SOUSA, G., DURINCK, S., KATSANOU, E. S., KONSTANTINIDOU, P., MACHERA, K., MILANI, E. S., PEIJNENBURG, A., RAHMANI, R., RAJKOVIC, A., RIJKERS, D., SPYROPOULOU, A., STAMOU, M., STOOPEN, G., STURLA, S., WOLLSCHEID, B., ZUCCHINI-PASCAL, N. & LAMPEN, A. 2018. Adverse Outcome Pathway-Driven Analysis of Liver Steatosis in Vitro: A Case Study with Cyproconazole. *Chem Res Toxicol,* 31**,** 784-798.

LUO, X., HE, Z., SUN, X., GU, X., ZHANG, W., GAO, J., LI, X., JIA, R., WEI, J., YU, Y. & LUO, X. 2020. DHA protects against hepatic steatosis by activating sirt1 in a high fat diet-induced nonalcoholic fatty liver disease mouse model. *Diabetes, Metabolic Syndrome and Obesity: Targets and Therapy,* 13**,** 185-196.

LV, Z., CHENG, J., HUANG, S., ZHANG, Y., WU, S., QIU, Y., GENG, Y., ZHANG, Q., HUANG, G., MA, Q., XIE, X., ZHOU, S., WU, T. & KE, Y. 2016. DEHP induces obesity and hypothyroidism through both central and peripheral pathways in C3H/He mice. *Obesity,* 24**,** 368-378.

MALINEN, M. M., ALI, I., BEZENÇON, J., BEAUDOIN, J. J. & BROUWER, K. L. R. 2018. Organic solute transporter OSTα/β is overexpressed in nonalcoholic steatohepatitis and modulated by drugs associated with liver injury. *American Journal of Physiology - Gastrointestinal and Liver Physiology,* 314**,** G597-G609.

MANOUSOPOULOU, A., SCORLETTI, E., SMITH, D. E., TENG, J., FOTOPOULOS, M., ROUMELIOTIS, T. I., CLOUGH, G. F., CALDER, P. C., BYRNE, C. D. & GARBIS, S. D. 2019. Marine omega-3 fatty acid supplementation in non-alcoholic fatty liver disease: Plasma proteomics in the randomized WELCOME* trial. *Clinical Nutrition,* 38**,** 1952-1955.

MANSOUR, A., MOHAJERI-TEHRANI, M. R., KARIMI, S., SANGINABADI, M., POUSTCHI, H., ENAYATI, S., ASGARBEIK, S., NASROLLAHZADEH, J. & HEKMATDOOST, A. 2020. Short term effects of coffee components consumption on gut microbiota in patients with non-alcoholic fatty liver and diabetes: A pilot randomized placebo-controlled, clinical trial. *EXCLI Journal,* 19**,** 241-250.

MANTEIGA, S. & LEE, K. 2017. Monoethylhexyl phthalate elicits an inflammatory response in adipocytes characterized by alterations in lipid and cytokine pathways. *Environmental Health Perspectives,* 125**,** 615-622.

MARMUGI, A., DUCHEIX, S., LASSERRE, F., POLIZZI, A., PARIS, A., PRIYMENKO, N., BERTRAND-MICHEL, J., PINEAU, T., GUILLOU, H., MARTIN, P. G. & MSELLI-LAKHAL, L. 2012. Low doses of bisphenol A induce gene expression related to lipid synthesis and trigger triglyceride accumulation in adult mouse liver. *Hepatology,* 55**,** 395-407.

MARTIN, R., ROSE, D., YU, K. & BARROS, S. 2006. Toxicogenomics strategies for predicting drug toxicity. *Pharmacogenomics,* 7**,** 1003-1016.

MARVENTANO, S., SALOMONE, F., GODOS, J., PLUCHINOTTA, F., DEL RIO, D., MISTRETTA, A. & GROSSO, G. 2016. Coffee and tea consumption in relation with non-alcoholic fatty liver and metabolic syndrome: A systematic review and meta-analysis of observational studies. *Clinical Nutrition,* 35**,** 1269-1281.

MELNICK, R. L. 2001. Is peroxisome proliferation an obligatory precursor step in the carcinogenicity of Di(2-ethylhexyl)phthalate (DEHP)? *Environmental Health Perspectives,* 109**,** 437-442.

MERAL, C., CEKMEZ, F., VURUCU, S., TASCILAR, E., PIRGON, O., CANPOLAT, F. E., IPCIOGLU, O. M., AYDEMIR, G. & AYDINOZ, S. 2011. New adipocytokines (vaspin, apelin, visfatin, adiponectin) levels in children treated with valproic acid. *European Cytokine Network,* 22**,** 118-122.

MESNAGE, R., BISERNI, M., GENKOVA, D., WESOLOWSKI, L. & ANTONIOU, M. N. 2018. Evaluation of neonicotinoid insecticides for oestrogenic, thyroidogenic and adipogenic activity reveals imidacloprid causes lipid accumulation. *Journal of Applied Toxicology,* 38**,** 1483-1491.

MEYER, A., SEIDLER, F. J. & SLOTKIN, T. A. 2004. Developmental effects of chlorpyrifos extend beyond neurotoxicity: critical periods for immediate and delayed-onset effects on cardiac and hepatic cell signaling. *Environ Health Perspect,* 112**,** 170-8.

MEZERA, V., KUCERA, O., MORAVCOVA, A., PETEROVA, E., ROUSAR, T., RYCHTRMOC, D., SOBOTKA, O. & CERVINKOVA, Z. 2015. Comparison of acetaminophen toxicity in primary hepatocytes isolated from transgenic mice with different apolipoprotein e alleles. *Journal of Physiology and Pharmacology,* 66**,** 863-873.

MICHAUT, A., LE GUILLOU, D., MOREAU, C., BUCHER, S., MCGILL, M. R., MARTINAIS, S., GICQUEL, T., MOREL, I., ROBIN, M. A., JAESCHKE, H. & FROMENTY, B. 2016. A cellular model to study drug-induced liver injury in nonalcoholic fatty liver disease: Application to acetaminophen. *Toxicology and Applied Pharmacology,* 292**,** 40-55.

MICHAUT, A., MOREAU, C., ROBIN, M. A. & FROMENTY, B. 2014. Acetaminophen-induced liver injury in obesity and nonalcoholic fatty liver disease. *Liver International,* 34**,** e171-e179.

MIGLIACCIO, V., SCUDIERO, R., SICA, R., LIONETTI, L. & PUTTI, R. 2019. Oxidative stress and mitochondrial uncoupling protein 2 expression in hepatic steatosis induced by exposure to xenobiotic DDE and high fat diet in male Wistar rats. *PLoS ONE,* 14.

MILOŠEVIĆ, N., MILIĆ, N., ŽIVANOVIĆ BOSIĆ, D., BAJKIN, I., PERČIĆ, I., ABENAVOLI, L. & MEDIĆ STOJANOSKA, M. 2018. Potential influence of the phthalates on normal liver function and cardiometabolic risk in males. *Environmental Monitoring and Assessment,* 190.

MITANI, T., NAGANO, T., HARADA, K., YAMASHITA, Y. & ASHIDA, H. 2017. Caffeine-stimulated intestinal epithelial cells suppress lipid accumulation in adipocytes. *Journal of Nutritional Science and Vitaminology,* 63**,** 331-338.

MOLES, A., TORRES, S., BAULIES, A., GARCIA-RUIZ, C. & FERNANDEZ-CHECA, J. C. 2018. Mitochondrial-lysosomal axis in acetaminophen hepatotoxicity. *Frontiers in Pharmacology,* 9.

MOLLOY, J. W., CALCAGNO, C. J., WILLIAMS, C. D., JONES, F. J., TORRES, D. M. & HARRISON, S. A. 2012. Association of coffee and caffeine consumption with fatty liver disease, nonalcoholic steatohepatitis, and degree of hepatic fibrosis. *Hepatology,* 55**,** 429-436.

MOUZAKI, M. & ALLARD, J. P. 2012. The role of nutrients in the development, progression, and treatment of nonalcoholic fatty liver disease. *Journal of Clinical Gastroenterology,* 46**,** 457-467.

MUSSO, G., GAMBINO, R., CASSADER, M. & PAGANO, G. 2010. A meta-analysis of randomized trials for the treatment of nonalcoholic fatty liver disease. *Hepatology,* 52**,** 79-104.

NAGAO, K., JINNOUCHI, T., KAI, S. & YANAGITA, T. 2013. Effect of dietary resveratrol on the metabolic profile of nutrients in obese OLETF rats. *Lipids in Health and Disease,* 12.

NAIR, S., DIEHL, A. M., WISEMAN, M., FARR JR, G. H. & PERRILLO, R. P. 2004. Metformin in the treatment of non-alcoholic steatohepatitis: A pilot open label trial. *Alimentary Pharmacology and Therapeutics,* 20**,** 23-28.

NAKAJIMA, A., EGUCHI, Y., YONEDA, M., IMAJO, K., TAMAKI, N., SUGANAMI, H., NOJIMA, T., TANIGAWA, R., IIZUKA, M., IIDA, Y. & LOOMBA, R. 2021. Randomised clinical trial: Pemafibrate, a novel selective peroxisome proliferator-activated receptor α modulator (SPPARMα), versus placebo in patients with non-alcoholic fatty liver disease. *Aliment Pharmacol Ther,* 54**,** 1263-1277.

NELSON-REES, W. A. & FLANDERMEYER, R. R. 1976. HeLa cultures defined. *Science,* 191**,** 96-8.

NEUSCHÄFER-RUBE, F., SCHRAPLAU, A., SCHEWE, B., LIESKE, S., KRÜTZFELDT, J. M., RINGEL, S., HENKEL, J., BIRKENFELD, A. L. & PÜSCHEL, G. P. 2015. Arylhydrocarbon receptor-dependent mIndy (Slc13a5) induction as possible contributor to benzo[a]pyrene-induced lipid accumulation in hepatocytes. *Toxicology,* 337**,** 1-9.

NEUSCHWANDER-TETRI, B. A., BRUNT, E. M., WEHMEIER, K. R., OLIVER, D. & BACON, B. R. 2003. Improved nonalcoholic steatohepatitis after 48 weeks of treatment with the PPAR-γ ligand rosiglitazone. *Hepatology,* 38**,** 1008-1017.

NIELSEN, E., NØRHEDE, P., BOBERG, J., KRAG ISLING, L., KROGHSBO, S., HADRUP, N., BREDSDORFF, L., MORTENSEN, A. & CHRISTIAN LARSEN, J. 2012. Identification of Cumulative Assessment Groups of Pesticides. *EFSA Supporting Publications,* 9**,** 269E.

NOBILI, V., MANCO, M., DEVITO, R., DI CIOMMO, V., COMPARCOLA, D., SARTORELLI, M. R., PIEMONTE, F., MARCELLINI, M. & ANGULO, P. 2008. Lifestyle intervention and antioxidant therapy in children with nonalcoholic fatty liver disease: A randomized, controlled trial. *Hepatology,* 48**,** 119-128.

NORDING, M. L., YANG, J., GEORGI, K., HEGEDUS KARBOWSKI, C., GERMAN, J. B., WEISS, R. H., HOGG, R. J., TRYGG, J., HAMMOCK, B. D. & ZIVKOVIC, A. M. 2013. Individual Variation in Lipidomic Profiles of Healthy Subjects in Response to Omega-3 Fatty Acids. *PLoS ONE,* 8.

OERLEMANS, A., VERSCHEIJDEN, L. F. M., MOL, J. G. J., VERMEULEN, R. C. H., WESTERHOUT, J., ROELEVELD, N., RUSSEL, F. G. M. & SCHEEPERS, P. T. J. 2019. Toxicokinetics of a urinary metabolite of tebuconazole following controlled oral and dermal administration in human volunteers. *Arch Toxicol,* 93**,** 2545-2553.

OLIVEIRA, C. P. M. S., DA COSTA GAYOTTO, L. C., TATAI, C., DELLA NINA, B. I., LIMA, E. S., ABDALLA, D. S. P., LOPASSO, F. P., LAURINDO, F. R. M. & CARRILHO, F. J. 2003. Vitamin C and vitamin E in prevention of Nonalcoholic Fatty Liver Disease (NAFLD) in choline deficient diet fed rats. *Nutrition Journal,* 2**,** 1-5.

OLSEN, G. W., EHRESMAN, D. J., BUEHRER, B. D., GIBSON, B. A., BUTENHOFF, J. L. & ZOBEL, L. R. 2012. Longitudinal assessment of lipid and hepatic clinical parameters in workers involved with the demolition of perfluoroalkyl manufacturing facilities. *J Occup Environ Med,* 54**,** 974-83.

OSCARSSON, J., ÖNNERHAG, K., RISÉRUS, U., SUNDÉN, M., JOHANSSON, L., JANSSON, P. A., MORIS, L., NILSSON, P. M., ERIKSSON, J. W. & LIND, L. 2018. Effects of free omega-3 carboxylic acids and fenofibrate on liver fat content in patients with hypertriglyceridemia and non-alcoholic fatty liver disease: A double-blind, randomized, placebo-controlled study. *Journal of Clinical Lipidology,* 12**,** 1390-1403.e4.

OTT, L. M., RAMACHANDRAN, K. & STEHNO-BITTEL, L. 2017. An Automated Multiplexed Hepatotoxicity and CYP Induction Assay Using HepaRG Cells in 2D and 3D. *SLAS Discov,* 22**,** 614-625.

PALM, R., SILSETH, C. & ALVAN, G. 1984. Phenytoin intoxication as the first symptom of fatal liver damage induced by sodium valproate. *British Journal of Clinical Pharmacology,* 17**,** 597-599.

PANCHAL, S. K., POUDYAL, H., WAANDERS, J. & BROWN, L. 2012. Coffee extract attenuates changes in cardiovascular and hepatic structure and function without decreasing obesity in high-carbohydrate, high-fat diet-fed male rats. *Journal of Nutrition,* 142**,** 690-697.

PAPANDREOU, C., HERNÁNDEZ-ALONSO, P., BULLÓ, M., RUIZ-CANELA, M., YU, E., GUASCH-FERRÉ, M., TOLEDO, E., DENNIS, C., DEIK, A., CLISH, C., RAZQUIN, C., CORELLA, D., ESTRUCH, R., ROS, E., FITÓ, M., ARÓS, F., FIOL, M., LAPETRA, J., RUANO, C., LIANG, L., MARTÍNEZ-GONZÁLEZ, M. A., HU, F. B. & SALAS-SALVADÓ, J. 2019. Plasma metabolites associated with coffee consumption: A metabolomic approach within the PREDIMED study. *Nutrients,* 11.

PARK, S., CHOI, Y. J. & LEE, B. H. 2012. In vitro validation of drug-induced phospholipidosis. *Journal of Toxicological Sciences,* 37**,** 261-267.

PARK, S. H., HAN, A. L., KIM, N. H. & SHIN, S. R. 2018. Liver histological improvement after administration of high-dose Vitamin C in guinea pig with nonalcoholic steatohepatitis. *International Journal for Vitamin and Nutrition Research,* 88**,** 263-269.

PARK, Y. & HARRIS, W. S. 2003. Omega-3 fatty acid supplementation accelerates chylomicron triglyceride clearance. *Journal of Lipid Research,* 44**,** 455-463.

PETIT, J., WAKX, A., GIL, S., FOURNIER, T., AUZEIL, N., RAT, P. & LAPRÉVOTE, O. 2018. Lipidome-wide disturbances of human placental JEG-3 cells by the presence of MEHP. *Biochimie,* 149**,** 1-8.

PHROMKUNTHONG, W., STORCH, V. & BRAUNBECKW, T. 1994. Sexual dimorphism in the reaction of zebrafish (Brachydanio rerio) to ascorbic acid deficiency: Induction of steatosis in hepatocytes of male fish. *Journal of Applied Ichthyology,* 10**,** 146-153.

PICKENS, M. K., YAN, J. S., NG, R. K., OGATA, H., GRENERT, J. P., BEYSEN, C., TURNER, S. M. & MAHER, J. J. 2009. Dietary sucrose is essential to the development of liver injury in the methionine-choline-deficient model of steatohepatitis. *Journal of Lipid Research,* 50**,** 2072-2082.

PIERCE, R. H., FRANKLIN, C. C., CAMPBELL, J. S., TONGE, R. P., CHEN, W., FAUSTO, N., NELSON, S. D. & BRUSCHI, S. A. 2002. Cell culture model for acetaminophen-induced hepatocyte death in vivo. *Biochemical Pharmacology,* 64**,** 413-424.

POPS REVIEW COMMITTEE 2022. Chlorpyrifos - draft risk profile. UNEP/POPS/POPRC.18/xxx.

POTRATZ, S., JUNGNICKEL, H., GRABIGER, S., TARNOW, P., OTTO, W., FRITSCHE, E., VON BERGEN, M. & LUCH, A. 2016. Differential cellular metabolite alterations in HaCaT cells caused by exposure to the aryl hydrocarbon receptor-binding polycyclic aromatic hydrocarbons chrysene, benzo[a]pyrene and dibenzo[a,l]pyrene. *Toxicology Reports,* 3**,** 763-773.

POWELL, C. L., KOSYK, O., ROSS, P. K., SCHOONHOVEN, R., BOYSEN, G., SWENBERG, J. A., HEINLOTH, A. N., BOORMAN, G. A., CUNNINGHAM, M. L., PAULES, R. S. & RUSYN, I. 2006. Phenotypic anchoring of acetaminophen-induced oxidative stress with gene expression profiles in rat liver. *Toxicological Sciences,* 93**,** 213-222.

PROT, J. M., BRIFFAUT, A. S., LETOURNEUR, F., CHAFEY, P., MERLIER, F., GRANDVALET, Y., LEGALLAIS, C. & LECLERC, E. 2011. Integrated proteomic and transcriptomic investigation of the acetaminophen toxicity in liver microfluidic biochip. *PLoS ONE,* 6.

PROT, J. M., BUNESCU, A., ELENA-HERRMANN, B., ANINAT, C., SNOUBER, L. C., GRISCOM, L., RAZAN, F., BOIS, F. Y., LEGALLAIS, C., BROCHOT, C., CORLU, A., DUMAS, M. E. & LECLERC, E. 2012. Predictive toxicology using systemic biology and liver microfluidic "on chip" approaches: Application to acetaminophen injury. *Toxicology and Applied Pharmacology,* 259**,** 270-280.

PURI, P., WIEST, M. M., CHEUNG, O., MIRSHAHI, F., SARGEANT, C., MIN, H. K., CONTOS, M. J., STERLING, R. K., FUCHS, M., ZHOU, H., WATKINS, S. M. & SANYAL, A. J. 2009. The plasma lipidomic signature of nonalcoholic steatohepatitis. *Hepatology,* 50**,** 1827-1838.

QIAO, X., LI, Y., MAI, J., JI, X. & LI, Q. 2018. Effect of dibutyltin dilaurate on triglyceride metabolism through the inhibition of the mTOR pathway in human HL7702 liver cells. *Molecules,* 23**,** 1-15.

QUAN, H. Y., KIM, D. Y. & CHUNG, S. H. 2013. Caffeine attenuates lipid accumulation via activation of AMP-activated protein kinase signaling pathway in HepG2 cells. *BMB Reports,* 46**,** 207-212.

RAFIEI, H., OMIDIAN, K. & BANDY, B. 2019. Dietary polyphenols protect against oleic acid-induced steatosis in an in vitro model of NAFLD by modulating lipid metabolism and improving mitochondrial function. *Nutrients,* 11.

RAKOSKI, M. O., SINGAL, A. G., ROGERS, M. A. M. & CONJEEVARAM, H. 2010. Meta-analysis: Insulin sensitizers for the treatment of non-alcoholic steatohepatitis. *Alimentary Pharmacology and Therapeutics,* 32**,** 1211-1221.

RAUCHENZAUNER, M., HABERLANDT, E., SCHOLL-BÜRGI, S., ERNST, B., HOPPICHLER, F., KARALL, D., EBENBICHLER, C. F., ROSTASY, K. & LUEF, G. 2008. Adiponectin and visfatin concentrations in children treated with valproic acid. *Epilepsia,* 49**,** 353-357.

RAZA, S., TEWARI, A., RAJAK, S. & SINHA, R. A. 2021. Vitamins and non-alcoholic fatty liver disease: A molecular insight. *Liver Research,* 5**,** 62-71.

REBOUISSOU, S., ZUCMAN-ROSSI, J., MOREAU, R., QIU, Z. & HUI, L. 2017. Note of caution: Contaminations of hepatocellular cell lines. *J Hepatol,* 67**,** 896-897.

REGNAULT, C., USAL, M., VEYRENC, S., COUTURIER, K., BATANDIER, C., BULTEAU, A. L., LEJON, D., SAPIN, A., COMBOURIEU, B., CHETIVEAUX, M., LE MAY, C., LAFOND, T., RAVETON, M. & REYNAUD, S. 2018. Unexpected metabolic disorders induced by endocrine disruptors in Xenopus tropicalis provide new lead for understanding amphibian decline. *Proc Natl Acad Sci U S A,* 115**,** E4416-e4425.

RIEDEL, A., PIGNITTER, M., HOCHKOGLER, C. M., ROHM, B., WALKER, J., BYTOF, G., LANTZ, I. & SOMOZA, V. 2012. Caffeine dose-dependently induces thermogenesis but restores ATP in HepG2 cells in culture. *Food and Function,* 3**,** 955-964.

RIVES, C., FOUGERAT, A., ELLERO-SIMATOS, S., LOISEAU, N., GUILLOU, H., GAMET-PAYRASTRE, L. & WAHLI, W. 2020. Oxidative stress in NAFLD: Role of nutrients and food contaminants. *Biomolecules,* 10**,** 1-69.

ROBERTS, R. A. 1999. Peroxisome proliferators: Mechanisms of adverse effects in rodents and molecular basis for species differences. *Archives of Toxicology,* 73**,** 413-418.

RODRÍGUEZ-ALCALÁ, L. M., SÁ, C., PIMENTEL, L. L., PESTANA, D., TEIXEIRA, D., FARIA, A., CALHAU, C. & GOMES, A. 2015. Endocrine Disruptor DDE Associated with a High-Fat Diet Enhances the Impairment of Liver Fatty Acid Composition in Rats. *Journal of Agricultural and Food Chemistry,* 63**,** 9341-9348.

RODRIGUEZ-ECHEVARRIA, R., MACIAS-BARRAGAN, J., PARRA-VARGAS, M., DAVILA-RODRIGUEZ, J. R., AMEZCUA-GALVEZ, E. & ARMENDARIZ-BORUNDA, J. 2018. Diet switch and omega-3 hydroxy-fatty acids display differential hepatoprotective effects in an obesity/ nonalcoholic fatty liver disease model in mice. *World Journal of Gastroenterology,* 24**,** 461-474.

ROGUE, A., ANTHÉRIEU, S., VLUGGENS, A., UMBDENSTOCK, T., CLAUDE, N., DE LA MOUREYRE-SPIRE, C., WEAVER, R. J. & GUILLOUZO, A. 2014. PPAR agonists reduce steatosis in oleic acid-overloaded HepaRG cells. *Toxicology and Applied Pharmacology,* 276**,** 73-81.

ROGUE, A., LAMBERT, C., JOSSÉ, R., ANTHERIEU, S., SPIRE, C., CLAUDE, N. & GUILLOUZO, A. 2011. Comparative gene expression profiles induced by PPARγ and PPARα/γ agonists in human hepatocytes. *PLoS ONE,* 6.

ROSE, M., VEYSEY, M., LUCOCK, M., NIBLETT, S., KING, K., BAINES, S. & GARG, M. L. 2016. Association between erythrocyte omega-3 polyunsaturated fatty acid levels and fatty liver index in older people is sex dependent. *Journal of Nutrition and Intermediary Metabolism,* 5**,** 78-85.

SAAB, S., MALLAM, D., COX, G. A. & TONG, M. J. 2014. Impact of coffee on liver diseases: A systematic review. *Liver International,* 34**,** 495-504.

SAID, A. & AKHTER, A. 2017. Meta-analysis of randomized controlled trials of pharmacologic agents in non-alcoholic steatohepatitis. *Annals of Hepatology,* 16**,** 538-547.

SALEH, D. A. A., ISMAIL, M. A. & IBRAHIM, A. M. 2012. Non alcoholic fatty liver disease, insulin resistance, dyslipidemia and atherogenic ratios in epileptic children and adolescents on long term antiepileptic drug therapy. *Pakistan Journal of Biological Sciences,* 15**,** 68-77.

SALOMONE, F., GALVANO, F. & VOLTI, G. L. 2017. Molecular bases underlying the hepatoprotective effects of coffee. *Nutrients,* 9.

SALVAGGIO, A., ANTOCI, F., MESSINA, A., FERRANTE, M., COPAT, C., RUBERTO, C., SCALISI, E. M., PECORARO, R. & BRUNDO, M. V. 2018. Teratogenic effects of the neonicotinoid thiacloprid on chick embryos (Gallus gallus domesticus). *Food and Chemical Toxicology,* 118**,** 812-820.

SANT, K. E., MOREAU, H. M., WILLIAMS, L. M., JACOBS, H. M., BOWSHER, A. M., BOISVERT, J. D., SMOLOWITZ, R. M., PANTAZIS, J., ANNUNZIATO, K., NGUYEN, M. & TIMME-LARAGY, A. 2021. Embryonic exposures to mono-2-ethylhexyl phthalate induce larval steatosis in zebrafish independent of Nrf2a signaling. *Journal of Developmental Origins of Health and Disease,* 12**,** 132-140.

SASAKI, Y., ASAHIYAMA, M., TANAKA, T., YAMAMOTO, S., MURAKAMI, K., KAMIYA, W., MATSUMURA, Y., OSAWA, T., ANAI, M., FRUCHART, J. C., ABURATANI, H., SAKAI, J. & KODAMA, T. 2020. Pemafibrate, a selective PPARα modulator, prevents non-alcoholic steatohepatitis development without reducing the hepatic triglyceride content. *Scientific Reports,* 10.

SATAKE, S., NAKAMURA, C., MINAMIDE, Y., KUDO, S., MAEDA, H., CHIHAYA, Y., KAMIMURA, Y., MIYAJIMA, H., SASAKI, J., GORYO, M. & OKADA, O. 2010. Effect of a large dose of Di (2-ethylhexyl) phthalate (DEHP) on hepatic peroxisome in cynomolgus monkeys (Macaca Fascicularis). *Journal of Toxicologic Pathology,* 23**,** 75-83.

SAVARY, C. C., JIANG, X., AUBRY, M., JOSSÉ, R., KOPP-SCHNEIDER, A., HEWITT, P. & GUILLOUZO, A. 2015. Transcriptomic analysis of untreated and drug-treated differentiated HepaRG cells over a 2-week period. *Toxicology in Vitro,* 30**,** 27-35.

SAVASTANO, S., TARANTINO, G., D'ESPOSITO, V., PASSARETTI, F., CABARO, S., LIOTTI, A., LIGUORO, D., PERRUOLO, G., ARIEMMA, F., FINELLI, C., BEGUINOT, F., FORMISANO, P. & VALENTINO, R. 2015. Bisphenol-A plasma levels are related to inflammatory markers, visceral obesity and insulin-resistance: A cross-sectional study on adult male population. *Journal of Translational Medicine,* 13.

SAWANGJIT, R., CHONGMELAXME, B., PHISALPRAPA, P., SAOKAEW, S., THAKKINSTIAN, A., KOWDLEY, K. V. & CHAIYAKUNAPRUK, N. 2016. Comparative efficacy of interventions on nonalcoholic fatty liver disease (NAFLD): A PRISMA-compliant systematic review and network meta-analysis. *Medicine (United States),* 95.

SCCP 2009. Opinion on triclosan. SCCP/1192/08. *(Scientific Committee on Consumer Products)***,** 136.

SCHAEDLICH, K., GEBAUER, S., HUNGER, L., BEIER, L. S., KOCH, H. M., WABITSCH, M., FISCHER, B. & ERNST, J. 2018. DEHP deregulates adipokine levels and impairs fatty acid storage in human SGBS-adipocytes. *Scientific Reports,* 8.

SCHMID, M. M., FREUDENMANN, R. W., KELLER, F., CONNEMANN, B. J., HIEMKE, C., GAHR, M., KRATZER, W., FUCHS, M. & SCHÖNFELDT-LECUONA, C. 2013. Non-fatal and fatal liver failure associated with valproic acid. *Pharmacopsychiatry,* 46**,** 63-68.

SCHMIDT, F., MARX-STOELTING, P., HAIDER, W., HEISE, T., KNEUER, C., LADWIG, M., BANNEKE, S., RIEKE, S. & NIEMANN, L. 2016. Combination effects of azole fungicides in male rats in a broad dose range. *Toxicology,* 355-356**,** 54-63.

SCHWIMMER, J. B., MIDDLETON, M. S., DEUTSCH, R. & LAVINE, J. E. 2005. A phase 2 clinical trial of metformin as a treatment for non-diabetic paediatric non-alcoholic steatohepatitis. *Alimentary Pharmacology and Therapeutics,* 21**,** 871-879.

SELMI-RUBY, S., MARÍN-SÁEZ, J., FILDIER, A., BULETÉ, A., ABDALLAH, M., GARCIA, J., DEVERCHÈRE, J., SPINNER, L., GIROUD, B., IBANEZ, S., GRANJON, T., BARDEL, C., PUISIEUX, A., FERVERS, B., VULLIET, E., PAYEN, L. & VIGNERON, A. M. 2020. In vivo characterization of the toxicological properties of DPhP, one of the main degradation products of aryl phosphate esters. *Environmental Health Perspectives,* 128**,** 1-18.

SHANG, J., CHEN, L. L., XIAO, F. X., SUN, H., DING, H. C. & XIAO, H. 2008. Resveratrol improves non-alcoholic fatty liver disease by activating AMP-activated protein kinase. *Acta Pharmacologica Sinica,* 29**,** 698-706.

SHARGORODSKY, M., OMELCHENKO, E., MATAS, Z., BOAZ, M. & GAVISH, D. 2012. Relation between augmentation index and adiponectin during one-year metformin treatment for nonalcoholic steatohepatosis: effects beyond glucose lowering? *Cardiovascular Diabetology,* 11.

SHEN, C., DOU, X., MA, Y., MA, W., LI, S. & SONG, Z. 2017. Nicotinamide protects hepatocytes against palmitate-induced lipotoxicity via SIRT1-dependent autophagy induction. *Nutrition Research,* 40**,** 40-47.

SHEN, H., RODRIGUEZ, A. C., SHIANI, A., LIPKA, S., SHAHZAD, G., MUSTACCHIA, P. & KUMAR, A. 2016. Association between caffeine consumption and nonalcoholic fatty liver disease: A systemic review and meta-analysis. *Therapeutic Advances in Gastroenterology,* 9**,** 113-120.

SHERLOCK, S. 1983. Acute fatty liver of pregnancy and the microvesicular fat diseases. *Gut,* 24**,** 265-269.

SHI, C., XUE, W., HAN, B., YANG, F., YIN, Y. & HU, C. 2019. Acetaminophen aggravates fat accumulation in NAFLD by inhibiting autophagy via the AMPK/mTOR pathway. *European Journal of Pharmacology,* 850**,** 15-22.

SHIELDS, W. W., THOMPSON, K. E., GRICE, G. A. & COYLE, W. J. 2009. The effect of metformin and standard therapy versus standard therapy alone in nondiabetic patients with insulin resistance and nonalcoholic steatohepatitis (NASH): A pilot trial. *Therapeutic Advances in Gastroenterology,* 2**,** 157-163.

SHOAITO, H., PETIT, J., CHISSEY, A., AUZEIL, N., GUIBOURDENCHE, J., GIL, S., LAPRÉVOTE, O., FOURNIER, T. & DEGRELLE, S. A. 2019. The Role of Peroxisome Proliferator–Activated Receptor Gamma (PPARγ) in Mono(2-ethylhexyl) Phthalate (MEHP)-Mediated Cytotrophoblast Differentiation. *Environmental health perspectives,* 127**,** 27003.

SILVA, M. F. B., AIRES, C. C. P., LUIS, P. B. M., RUITER, J. P. N., IJLST, L., DURAN, M., WANDERS, R. J. A. & TAVARES DE ALMEIDA, I. 2008. Valproic acid metabolism and its effects on mitochondrial fatty acid oxidation: A review. *Journal of Inherited Metabolic Disease,* 31**,** 205-216.

SINGH, N., AHUJA, V., SACHDEV, V., UPADHYAY, A. D., GOSWAMI, R., RAMAKRISHNAN, L., DWIVEDI, S. & SARAYA, A. 2020. Antioxidants for Pancreatic Functions in Chronic Pancreatitis: A Double-blind Randomized Placebo-controlled Pilot Study. *Journal of Clinical Gastroenterology,* 54**,** 284-293.

SINGH, S., KHERA, R., ALLEN, A. M., MURAD, M. H. & LOOMBA, R. 2015. Comparative effectiveness of pharmacological interventions for nonalcoholic steatohepatitis: A systematic review and network meta-analysis. *Hepatology,* 62**,** 1417-1432.

SINHA, R. A., FARAH, B. L., SINGH, B. K., SIDDIQUE, M. M., LI, Y., WU, Y., ILKAYEVA, O. R., GOODING, J., CHING, J., ZHOU, J., MARTINEZ, L., XIE, S., BAY, B. H., SUMMERS, S. A., NEWGARD, C. B. & YEN, P. M. 2014. Caffeine stimulates hepatic lipid metabolism by the autophagy-lysosomal pathway in mice. *Hepatology,* 59**,** 1366-1380.

SLOTKIN, T. A., BROWN, K. K. & SEIDLER, F. J. 2005. Developmental exposures of rats to chlorpyrifos elicits sex-selective hyperlipidemia and hyperinsulinemia in adulthood. *Environmental Health Perspectives,* 113**,** 1291-1294.

SOL, C. M., SANTOS, S., DUIJTS, L., ASIMAKOPOULOS, A. G., MARTINEZ-MORAL, M. P., KANNAN, K., JADDOE, V. W. V. & TRASANDE, L. 2020a. Fetal phthalates and bisphenols and childhood lipid and glucose metabolism. A population-based prospective cohort study. *Environment International,* 144.

SOL, C. M., SANTOS, S., DUIJTS, L., ASIMAKOPOULOS, A. G., MARTINEZ-MORAL, M. P., KANNAN, K., PHILIPS, E. M., TRASANDE, L. & JADDOE, V. W. V. 2020b. Fetal exposure to phthalates and bisphenols and childhood general and organ fat. A population-based prospective cohort study. *International Journal of Obesity,* 44**,** 2225-2235.

SONG, Y. M., LEE, Y. H., KIM, J. W., HAM, D. S., KANG, E. S., CHA, B. S., LEE, H. C. & LEE, B. W. 2015. Metformin alleviates hepatosteatosis by restoring SIRT1-mediated autophagy induction via an AMP-activated protein kinase-independent pathway. *Autophagy,* 11**,** 46-59.

SPRATLEN, M. J., PERERA, F. P., LEDERMAN, S. A., ROBINSON, M., KANNAN, K., HERBSTMAN, J. & TRASANDE, L. 2020. The Association Between Perfluoroalkyl Substances and Lipids in Cord Blood. *J Clin Endocrinol Metab,* 105**,** 43-54.

STEWART, J. D., HORVATH, R., BARUFFINI, E., FERRERO, I., BULST, S., WATKINS, P. B., FONTANA, R. J., DAY, C. P. & CHINNERY, P. F. 2010. Polymerase γ Gene POLG determines the risk of sodium valproate-induced liver toxicity. *Hepatology,* 52**,** 1791-1796.

STOSSI, F., DANDEKAR, R. D., JOHNSON, H., LAVERE, P., FOULDS, C. E., MANCINI, M. G. & MANCINI, M. A. 2019. Tributyltin chloride (TBT) induces RXRA down-regulation and lipid accumulation in human liver cells. *PLOS ONE,* 14**,** e0224405.

STRICKER, B. H., BLOK, A. P., BRONKHORST, F. B., VAN PARYS, G. E. & DESMET, V. J. 1986. Ketoconazole-associated hepatic injury. A clinicopathological study of 55 cases. *J Hepatol,* 3**,** 399-406.

SUGENG, E. J., SYMEONIDES, C., O'HELY, M., VUILLERMIN, P., SLY, P. D., VIJAYASARATHY, S., THOMPSON, K., PEZIC, A., MUELLER, J. F., PONSONBY, A. L. & THE BARWON INFANT STUDY INVESTIGATOR, G. 2020. Predictors with regard to ingestion, inhalation and dermal absorption of estimated phthalate daily intakes in pregnant women: The Barwon infant study. *Environment International,* 139.

SUMIOKA, I., MATSURA, T. & YAMADA, K. 2004. Acetaminophen-induced hepatotoxicity: Still an important issue. *Yonago Acta Medica,* 47**,** 17-28.

SVENSSON, L. T., ENGBERG, S. T., AOYAMA, T., USUDA, N., ALEXSON, S. E. H. & HASHIMOTO, T. 1998. Molecular cloning and characterization of a mitochondrial peroxisome proliferator-induced acyl-CoA thioesterase from rat liver. *Biochemical Journal,* 329**,** 601-608.

SZALOWSKA, E., VAN DER BURG, B., MAN, H. Y., HENDRIKSEN, P. J. M. & PEIJNENBURG, A. A. C. M. 2014. Model steatogenic compounds (amiodarone, valproic acid, and tetracycline) alter lipid metabolism by different mechanisms in mouse liver slices. *PLoS ONE,* 9.

TAKAYAMA, S., SIEBER, S. M., DALGARD, D. W., THORGEIRSSON, U. P. & ADAMSON, R. H. 1999. Effects of long-term oral administration of DDT on nonhuman primates. *J Cancer Res Clin Oncol,* 125**,** 219-25.

TANG, X., LI, J., XIANG, W., CUI, Y., XIE, B., WANG, X., XU, Z. & GAN, L. 2016. Metformin increases hepatic leptin receptor and decreases steatosis in mice. *Journal of Endocrinology,* 230**,** 227-237.

TÊTE, A., GALLAIS, I., IMRAN, M., CHEVANNE, M., LIAMIN, M., SPARFEL, L., BUCHER, S., BUREL, A., PODECHARD, N., APPENZELLER, B. M. R., FROMENTY, B., GROVA, N., SERGENT, O. & LAGADIC-GOSSMANN, D. 2018. Mechanisms involved in the death of steatotic WIF-B9 hepatocytes co-exposed to benzo[a]pyrene and ethanol: a possible key role for xenobiotic metabolism and nitric oxide. *Free Radic Biol Med,* 129**,** 323-337.

TÊTE, A., GALLAIS, I., IMRAN, M., LEGOFF, L., MARTIN-CHOULY, C., SPARFEL, L., BESCHER, M., SERGENT, O., PODECHARD, N. & LAGADIC-GOSSMANN, D. 2020. MEHP/ethanol co-exposure favors the death of steatotic hepatocytes, possibly through CYP4A and ADH involvement. *Food and Chemical Toxicology,* 146.

TILLMAN, E. M., GUAN, P., HOWZE, T. J., HELMS, R. A. & BLACK, D. D. 2016. Role of PPARα in the attenuation of bile acid-induced apoptosis by omega-3 long-chain polyunsaturated fatty acids in cultured hepatocytes. *Pediatric Research,* 79**,** 754-758.

TIMCHALK, C., KOUSBA, A. A. & POET, T. S. 2007. An age-dependent physiologically based pharmacokinetic/pharmacodynamic model for the organophosphorus insecticide chlorpyrifos in the preweanling rat. *Toxicological Sciences,* 98**,** 348-365.

TOLOSA, L., GÓMEZ-LECHÓN, M. J., JIMÉNEZ, N., HERVÁS, D., JOVER, R. & DONATO, M. T. 2016. Advantageous use of HepaRG cells for the screening and mechanistic study of drug-induced steatosis. *Toxicology and Applied Pharmacology,* 302**,** 1-9.

TORRES, D. M. & HARRISON, S. A. 2013. Is it time to write a prescription for coffee? Coffee and liver d. *Gastroenterology,* 144**,** 670-672.

TORRES, S., BAULIES, A., INSAUSTI-URKIA, N., ALARCÓN-VILA, C., FUCHO, R., SOLSONA-VILARRASA, E., NÚÑEZ, S., ROBLES, D., RIBAS, V., WAKEFIELD, L., GROMPE, M., LUCENA, M. I., ANDRADE, R. J., WIN, S., AUNG, T. A., KAPLOWITZ, N., GARCÍA-RUIZ, C. & FERNÁNDEZ-CHECA, J. C. 2019. Endoplasmic Reticulum Stress-Induced Upregulation of STARD1 Promotes Acetaminophen-Induced Acute Liver Failure. *Gastroenterology,* 157**,** 552-568.

TRASANDE, L., ATTINA, T. M., SATHYANARAYANA, S., SPANIER, A. J. & BLUSTEIN, J. 2013. Race/ethnicity-specific associations of urinary phthalates with childhood body mass in a nationally representative sample. *Environmental Health Perspectives,* 121**,** 501-506.

TROVATO, G. M., MARTINES, G. F., TROVATO, F. M. & CATALANO, D. 2013. Regular coffee: A magic bullet or a naked gun? Regular coffee but not espresso drinking is protective against fibrosis in NAFLD. *Journal of Hepatology,* 58**,** 1264-1265.

TUNG, E. W. Y., PESHDARY, V., GAGNÉ, R., ROWAN-CARROLL, A., YAUK, C. L., BOUDREAU, A. & ATLAS, E. 2017. Adipogenic effects and gene expression profiling of firemaster ® 550 components in human primary preadipocytes. *Environmental Health Perspectives,* 125.

TURNBULL, D. M., DICK, D. J., WILSON, L., SHERRATT, H. S. & ALBERTI, K. G. 1986. Valproate causes metabolic disturbance in normal man. *Journal of Neurology Neurosurgery and Psychiatry,* 49**,** 405-410.

UED, F. V. & WEFFORT, V. R. S. 2013. Antioxidant vitamins in the context of nonalcoholic fatty liver disease in obese children and adolescents. *Revista Paulista de Pediatria,* 31**,** 523-530.

UNEP 2019. Stockholm Convention on Persistent Organic Pollutants (POPs) (2019 revised version in press). *Texts and Annexes, revised in 2017.*

US FDA. *Cordarone (amiodarone HCl) Tablets, medicinal product print label. Reference ID: 2876651* [Online]. Available: <https://www.accessdata.fda.gov/drugsatfda_docs/label/2010/018972s042lbl.pdf> [Accessed 27/04 2022].
[truncated: 16,429 more chars]
